# Finding the undiscovered roles of genes: an approach using mutual ranking of coexpressed genes and promoter architecture-case study: dual roles of thaumatin like proteins in biotic and abiotic stresses

**DOI:** 10.1186/2193-1801-1-30

**Published:** 2012-10-05

**Authors:** Tahereh Deihimi, Ali Niazi, Mansour Ebrahimi, Kimia Kajbaf, Somaye Fanaee, Mohammad Reza Bakhtiarizadeh, Esmaeile Ebrahimie

**Affiliations:** 1 Institute of Biotechnology, Shiraz University, Shiraz, Iran; 2 Department of Biology & Bioinformatics Research Group, University of Qom, Qom, Iran; 3 Department of Animal Science, University of Tehran, Karaj, Iran; 4 Department of Crop Production & Plant Breeding, College of Agriculture, Shiraz University, 71441 Shiraz, Iran; 5 School of Molecular & Biomedical Science, The University of Adelaide, Adelaide, SA Australia

**Keywords:** Promoter analysis, Domain and prosite analysis, Gene expression, Multivariate analysis, Thaumatin like proteins, Stress

## Abstract

Regarding the possible multiple functions of a specific gene, finding the alternative roles of genes is a major challenge. Huge amount of available expression data and the central role of the promoter and its regulatory elements provide unique opportunely to address this issue. The question is that how the expression data and promoter analysis can be applied to uncover the different functions of a gene. A computational approach has been presented here by analysis of promoter regulatory elements, coexpressed gene as well as protein domain and prosite analysis. We applied our approach on Thaumatin like protein (TLP) as example. TLP is of group 5 of pathogenesis related proteins which their antifungal role has been proved previously. In contrast, Osmotin like proteins (OLPs) are basic form of TLPs with proved role only in abiotic stresses. We demonstrated the possible outstanding homolouges involving in both biotic and abiotic stresses by analyzing 300 coexpressed genes for each Arabidopsis TLP and OLP in biotic, abiotic, hormone, and light microarray experiments based on mutual ranking. In addition, promoter analysis was employed to detect transcription factor binding sites (TFBs) and their differences between OLPs and TLPs. A specific combination of five TFBs was found in all TLPs presenting the key structure in functional response of TLP to fungal stress. Interestingly, we found the fungal response TFBs in some of salt responsive OLPs, indicating the possible role of OLPs in biotic stresses. Thirteen TFBS were unique for all OLPs and some found in TLPs, proposing the possible role of these TLPs in abiotic stresses. Multivariate analysis showed the possibility of estimating models for distinguishing biotic and abiotic functions of TIPs based on promoter regulatory elements. This is the first report in identifying multiple roles of TLPs and OLPs in biotic and abiotic stresses. This study provides valuable clues for screening and discovering new genes with possible roles in tolerance against both biotic and abiotic stresses. Interestingly, principle component analysis showed that promoter regulatory elements of TLPs and OLPs are more variable than protein properties reinforcing the prominent role of promoter architecture in determining gene function alteration.

**Electronic supplementary material:**

The online version of this article (doi:10.1186/2193-1801-1-30) contains supplementary material, which is available to authorized users.

## Introduction

Although non-coding sequences play a key role in transcriptional regulation, most of the studies have focused on identifying the genes and predicting their function based on coding sequences. However, gene function is the outcome of upstream non-coding promoter region and downstream coding sequence. Transcription factor binding sites (TFBs or cis-regulatory elements) which identify the specific timing and location of transcriptional activity are placed in the long non-coding sequence upstream of a gene. Diverse cis-regulatory modules are required for a specific expression pattern (Su et al. [2010]). Consequently, the identification of regulatory motifs and their organization modules is an important step to improve understanding of gene expression and regulation. Consequently, promoter analysis can open a new avenue in the field of genes with unknown function.

As many phenotypes are the result of complex gene-gene interactions, there is an increased interest identifying gene sets underlying the expression of a given phenotype ( [Fichlin and FaFFA 2010]). Interaction relationships among genes have not been allocated by the individual gene. Sharing the genes between different networks (cross talk) is common in system biology; as a result, one gene can play different functions. For instance, a gene can play bifunctional roles in biotic and abiotic stresses. Huge amount of available expression data and recent advances in sequencing of promoter regions provide the valuable opportunity for prediction of gene functions. However, a defined reliable approach is highly required here.

Thus, expression data and computational analysis might reveal the coexpressed gene subsets which are described to be highly correlated under one condition but uncorrelated under another condition ( [Varadan and Anastassiou 2006]). The coexpressed genes should be analyzed by gene subsets rather than individual genes. Identification of stress specific coexpressed gene subsets is very useful for finding unfamiliar gene role ( [Zhang et al. 2009]). In this study, we defined a subset of coexpressed genes based on Mutual Rank (MR) index. For any given pair, gene A and gene B, the MR is calculated as an average of the rank of gene B in the coexpressed genes to gene A and the average of the rank of gene A to gene B. It has been documented that MR is the better measure of similarity than the correlation value in order to determine related genes ( [Obayashi et al. 2009]). This is partly because even the gene pair with low expression similarities can work together if no other genes are highly coexpressed, as in some examples where one gene is highly coexpressed according to the MRs, although expression similarities are low ([Obayashi et al. 2007]).

In addition to promoter and coexpressed gene analysis, to reveal the function of proteins the use of protein sequence patterns, especially discovery of prosite signature, is becoming one of the vital tools of sequence analysis. Short well-conserved regions of proteins are adapted as prosite ( [Hulo et al. 2008]). They are typically enzyme catalytic sites, prosthetic group-attachment sites (haem, pyridoxal phosphate, biotin, etc.), metal ion-binding amino acids, cysteines involved in disulfide bonds or regions involved in binding a molecule ( [Hulo et al. 2008]). In our previous study, we employed motif and domain analysis to predict different subcellular locations of glutathione reductase proteins ( [Tahmasebi et al. 2012]).

As example, we analyzed a family of plant defense genes in plants. Defense mechanisms of plants are induced by multiple genes during different stresses. Manipulation of multiple genes is needed to artificially confer resistance to plants which is a time-consuming and labor-intensive task. As a result, finding the genes which their transformation can up-regulate some resistant genes simultaneously is of a great interest. Except transcription factors, Thaumatin like proteins (TLPs) are one of the best candidates for this purpose ( [Breiteneder 2000]). TLPs have been categorized as a family 5 of Pathogenesis Related Proteins (PRs) ( [Zhong and Shen 2004]). The induction of TLPs in plants resistance mechanism during pathogen infection has been proved ( [Petre et al. 2011]). For decades, TLPs switching on by pathogens such as bacteria, virus and fungi has been defined in many higher plants ( [Liu and Ekramoddoullah 2010]; [Mukherjee et al. 2010]). Although TLPs mechanisms remain unclear ( [Petre et al. 2011]), membrane permeability ( [Vigers and Selitrennikoff 1991]), b-glucan binding and degradation ( [Sakamoto et al. 2006]), inhibition of enzymes such as xylanases ( [Fierens et al. 2007]), a-amylase, or trypsin (Schimoler-O’ [Rourke and Selitrennikoff 2001]), possessed to some TLPs antifungal activity. Moreover participation of TLPs in pathogen defense mechanism, [Rajam et al. 2007] have reported other functional properties for protection against abiotic stresses ( [Rajam et al. 2007]).

TLPs basic isoform, Osmotin like proteins (OLPs), with a molecular weight of 24 KDa have reported as osmoprotectant in the tobacco cells ( [Abada et al. 1996]; [Yun et al. 1997]). OLP protein and genomic sequence hasve been isolated from treated tobacco with high NaCl concentration ( [Singh et al. 1985]). Proline accumulation happens by upregulation of osmotin conferring tolerance to osmotic stress in transgenic tobacco ( [Barthakur SBVB 2001]). Besides induction of OLPs during salt stress, evidences show that a broad range of fungal pathogens can activate these proteins ( [Abada et al. 1996]; [Yun et al. 1997]).

Regarding the valuable role of TLPs in resistance to both biotic and abiotic stresses, deciphering the complex mechanism and function of these protein homologs is interesting. Bioinformatics provide valuable tools in elucidating the function of mysterious gene. In this research, promoter analysis, analysis of coexpressed genes, and prosite study were employed to shed light on diverse functions of TLPs. The nature of specific cis-elements as activators, repressors, enhancers and chromatin modifiers is detectors of gene activities and combinatorial transcriptional regulation in plants ( [Yu et al. 2003]). However, the differences between the function of TLP and OLP promoters are remained unknown. This study discovers the key elements responsible for dual role of TLPs in both biotic and abiotic stresses by *in silico* TLP and OLP comparative model analysis based on promoter characteristics.

In this study, a variety of bioinformatics tools including coexpressed genes determination, *in silico* promoter analysis, as well as *in silico* domains and prosite discovery were used to provide clues for better understanding and prediction of these diverse functions of TLPs and OLPs in Arabidopsis (*Arabidopsis thaliana)* and Rice (*Oryza sativa*). Furthermore, a statistical approach has been developed for prediction and distinguishing different functions of genes based on Mutual Ranking of coexpressed genes and multivariate analysis of regulatory elements on promoter regions.

## Result and discussion

### Promoter analysis

Analysis of 1500 bp promoter sequence of Arabidopsis and Rice in both TLPs and OLPs groups predicted 34 fundamental specific transcription factor binding sites (TFBs) in all promoters. Thirteen TFBs were detected by TLPs promoter analyses. In contrast, only 5 TFBS were shared between all TLP genes (Table 1).

**Table 1:** **Transcription factor binding sites on the promoter region of Thaumatin like proteins (TLPs) and Osmotin like proteins (OLPs)**

Thirteen cis-acting regulatory elements which are shared between all OLPs		Five cis-acting regulatory elements which are shared between all TLPs	
Name	Function	Name	Function
ABRE	ABA inducible transcriptional activator	ASRC	Pathogen defense
CAAT	CAAT box	CCAF	Circadian clock associated
CARM	CA-rich element	L1BX	Homeodomain protein
CNAC	Calmodulin binding NAC protein	NCS1	Nodulin consensus sequence
GAGA	(GA)n/(CT)n binding proteins	WBXF	Pathogen defense
IDDF	Intermediate zinc figure protein		
LEGB	Iron-deficiency-responsive element		
MIIG	Activator of flavonoid biosynthesis gene		
NACF	Transcription factor binding to the iron deficiency-responsive element		
OPAQ	Transcriptional activator		
PSPE	SA induction of secreted gene		
SPF1	DNA binding protein that binds to beta amylase		
WNAC	NAC domain DNA binding factor		

Regarding the proved role of TLPs in fungal/biotic resistance, these 5 elements can be assumed as biotic-defense elements for TLPs function. Interestingly, these 5 biotic-defense TFBs were found on some of OLPs (Table 2). As a result, theses OLPs can be expressed during salt abiotic stresses and biotic fungal stress making them as super resistance genes. It should be noted that identification of these genes by common laboratory techniques is a time-consuming and expensive method, while this rapid bioinformatics approach can provide a short list of potential outstanding homologs with dual resistance properties for further laboratory tests.

**Table 2:** **Screening the Thaumatin like proteins which can perform dual function against fungal (biotic) and salt (abiotic) stresses through presented promoter regulatory element model (TFBs) in this research for biotic and abiotic stresses**

Organism	Locus	Primary resistance function	Extra regulatory elements related to another type of stress (biotic/abiotic)	Secondary predicted resistance function
TLP				
Arabidopsis	AT1G75030 (TLP)	Fungal resistance	ABRE/CAAT/CARM/IDDF/OPAQ/PSPE/SPF1/WNAC	Salt resistance
Arabidopsis	AT1G18250 (TLP)	Fungal resistance	ABRE/CARM/CNAC/GAGA/IDDF/LEGB/MIIG/NACF/OPAQ/SPF1/WNAC	Salt resistance
Arabidopsis	AT1G73620 (TLP)	Fungal resistance	CAAT/CARM/CNAC/GAGA/IDDF/NACF/OPAQ/PSPE/SPF1/WNAC	Salt resistance
Arabidopsis	AT1G77700 (TLP)	Fungal resistance	ABRE/CAAT/CARM/CNAC/GAGA/IDDF/MIIG/OPAQ/PSPE/SPF1	Salt resistance
Arabidopsis	AT4G36010 (TLP)	Fungal resistance	ABRE/CAAT/CARM/CNAC/GAGA/IDDF/LEGB/MIIG/NACF/OPAQ/PSPE/SPF1/WNAC	Salt resistance
Arabidopsis	AT4G38660.1 (TLP)	Fungal resistance	CAAT/CNAC/GAGA/IDDF/MIIG/NACF/OPAQ/PSPE/SPF1	Salt resistance
Arabidopsis	AT5G02140 (TLP)	Fungal resistance	ABRE/CARM/CNAC/MIIG/NACF/OPAQ/PSPE/WNAC	Salt resistance
Arabidopsis	AT5G40020 (TLP)	Fungal resistance	ABRE/CAAT/CARM/IDDF/NACF/OPAQ/SPF1/WNAC	Salt resistance
Rice	Os04G0689900 (TLP)	Fungal resistance	ABRE/CAAT/CARM/CNAC/IDDF/LEGB/MIIG/NACF/OPAQ/PSPE/SPF1	Salt resistance
Rice	Os10G0412700 (TLP)	Fungal resistance	ABRE/CAAT/CARM/CNAC/GAGA/LEGB/MIIG/NACF/OPAQ/PSPE/SPF1/WNAC	Salt resistance
OLP				
Arabidopsis	AT2G28790 (OLP)	salt resistance	ASRC/CCAF/L1BX/NCS1/WBXF	Fungal resistance
Arabidopsis	AT4G11650 (OLP)	salt resistance	ASRC/CCAF/NCS1/WBXF	Fungal resistance
Arabidopsis	AT1G75800 (OLP)	salt resistance	ASRC/CCAF/L1BX/NCS1/WBXF	Fungal resistance

**Figure 1:**
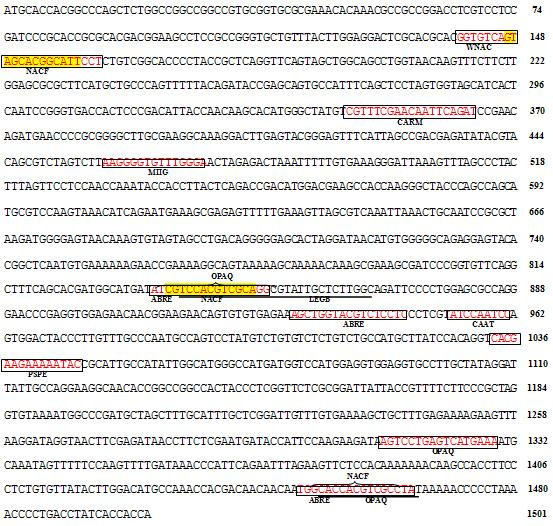
**The sequence and the predicted cis-elements of the positive strand of putative promoter region of OLP in Rice (Os01g0839900).** This homolog solely contains abiotics elements on promoter.

**Figure 2:**
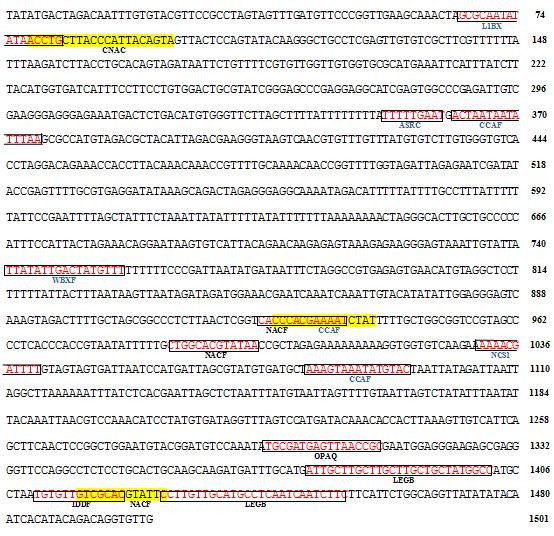
**The sequence and the predicted cis-elements of the positive strand of putative promoter region of TLP in Rice (Os04g0689900).** This homolog contains both abiotics and biotic elements on promoter.

Rice OLP isoform (Os01g0839900) does not carry the shared elements of TLPs. In contrast, the majority of OLPs in Arabidopsis contain the shared biotic responsible elements of TLPs (Table 2). Consequently, these OLPs homologes may upregulate in both biotic and abiotic stresses. The sequences and the predicted cis-elements of Rice OLP (Os01g0839900) and Rice TLP (Os04g0689900) have been presented in Figure 1 and Figure 2.

*In silico* promoter analysis of OLPs detected 21 TFBS which 13 of them were shared between all OLPs (Table 1). The function of these 13 TFBS mainly was related to salt stress. Some TLPs carry this structure similar to OLPs showing possible roles in salt/abiotic resistance as well as fungal/biotic resistance (Table 2). Some TLPs in Rice had the OLP-salt resistance elements except 3 of them showing the role in fungal stress (Table 2).

With regard to the central role of the promoter and its regulatory elements, it seems that the most researchers have missed the outstanding advantages of promoter analysis in prediction of gene function and discovering the genes with similar function. Here, for the first time, we found the conserved combination model of regulatory elements on the promoter of TLP fungal resistance genes (ASRC/CCAF/L1BX/NCS1/WBXF) which can efficiently be used for screening the genes with unknown function and finding the new effective genes in fungal and biotic resistance. In the same road, a unique complex regulatory element combination (ABRE/CARM/CNAC/GAGA/IDDF/LEGB/MIIG/NACF/OPAQ/SPF1/WNAC) was found for screening the effective genes involved in abiotic salt stress (Table 1).

The results revealed the dramatic differences between OLPs in rice with Arabidopsis. While most of Arabidopsis OLPs promoters carry the additional fungal response TFBs, Rice OLPs does not have this structure. In other words, opposite to Arabidopsis, Rice OLPs are mainly involved in salt stress. This finding highlights the crucial role of considering homolog source of gene and promoter at the time of gene isolation and transferring.

### Coexpressed gene analysis

Another *in silico* analysis tool, which can provide valuable clues about different functions of a gene, is analysis of coexpressed genes with gene of interest using available transcriptomics data in databases. The analysis of coexpressed gene using deposited microarray data indicated the role of some Arabidopsis’s TLPs in abiotic stresses and some OLPs in biotic stresses (Table 3, Additional file 1). We analyzed 300 coexpressed genes and selected some genes with MR < 10 for each TLPs and OLPs in biotic, abiotic, hormone and light microarray experiments by ATTED-II ( http://atted.jp). Based on the function of each coexpressed gene in each experiment, we could suggest the outstanding role of some TLPs and OLPs in response to both biotic and abiotic stresses. As presented in Table 3, among 21 TLPs, just 2 of them (AT1G19320/AT4G36000) has no coexpressed gene with MR < 10 in abiotic experiments revealing that these two isoforms upregulate specificly in biotic experiments. This result identified that AT1G19320 and AT4G36000 can be activated solely in response to biotic stresses in plants. In contrast, other 19 isoforms of TLPs have coexpressed gene with MR < 10 in both biotic and abiotic stresses. This result suggests the bifunctional role of some TLPs homologs in response to biotic and abiotic stresses (Table 3, Additional file 1). In OLP group, AT2G28790 does not activate by biotic stresses because there is no coexpressed gene by MR < 10 in biotic experiments by this OLP homolog. In contrast, there are 7 genes (At3g12500/At1g02220/At3g01420/At3g60140/At1g55020/At2g14620/At3g21500) in biotic microarray experiments which coexpressed by another isoform of OLP (At4g11650).

**Table 3:** **Coexpressed genes with TLPs and OLPs loci in different biotic, abiotic, hormone and light microarray experiments**

lllkk	Type	Coexpressed genes in abiotic microarray experiments	Coexpressed genes in biotic microarray experiments	Coexpressed genes in hormone microarray experiments	Coexpressed genes in light Microarray experiments	Fnction prediction
At1g73620	TLP	At3g03130/260118_s_at	-	At2g20515/At5g50375/At3g20015/At5g08640	-	abiotic
At1G75030	TLP	At2g03200/At2g24140/ At3g06390/At2g22510	-	-	254338_s_at/At3g06390/247765_at	abiotic
At1G18250	TLP	At3g53190/At4g15830/ At1g21880/At2g25060/ At1g44110/At2g36200/ At1g29980/At5g48360/ At2g27970/At2g28790/ At4g03100/At5g62550/ At4g39630/At1g33040/	At4g34160/At1g02730/ At1g76540/At1g30600/ At4g31840/At2g13820/	At4g34160/At3g02640/ At3g15680/At5g16250/ At2g36570/At4g31840/ At1g47670/At1g72670/	-	Abiotic/biotic
At5G24620	TLP	At5g43830/At1g24120/ At1g03160/At3g21060/ At5g24610/At2g01130/	-	At5g47500/At3g57470/ At3g33530/265974_at	At3g55020	abiotic
At5G02140	TLP	At1g64920/At2g42250/	-	-	-	abiotic
AT1G19320	TLP	-	-	-	-	biotic
AT1G20030	TLP	At4g23040/At1g22770/ At4g18270/At3g53990/ At4g18530	At2g31360/At4g18270At4g25480/ At3g24515At1g48330/At2g45560	-	-	Abiotic/biotic
AT1G75040	TLP	At5g60950/At5g55450/ At2g32680	At5g24530/At2g18660	At3g57240/At2g14560/ At5g55450/At2g18660/ At2g14610/254265_s_at/ At5g10760	-	Abiotic/biotic
AT1G75050	TLP	At3g06100/At3g23770/ At5g53190	-	-	-	abiotic
AT1G75800	TLP	At3g05120/At2g15890/ At4g05150/At1g28330	At3g60530/At1g22740	-	At1g74840	Abiotic
AT1G77700	TLP	At5g20870/At5g56720/ At1g73370/At3g15800/ At5g25370/250853_s_at	-	-	-	abiotic
AT2G17860	TLP	At1g04625	-	-	-	abiotic
AT2G28790	TLP	At2g37910/At2g10340/ At2g15810/At1g28160/ At2g02550/At3g15860/ At1g67220	At1g33220/At2g11010At1g32980/ At1g30473/At1g63540	-	-	Abiotic/biotic
AT4G18250	TLP	At1g67800/At2g38290/ At1g08050/At1g79680/ At3g09010/At4g29050/ At3g09405/At4g11850/ 246927_s_at	At3g19010/At3g59660At3g28450/ At4g23150 At4g23280/ At4g03450 At1g51890/ At1g51920/At1g26420	At4g26120/At5g26920/ At2g38290/At2g20142/ At2g37910/At1g64250/ 246927_s_at/At1g57630 At1g01340/ At1g43680/At2g23680/ At1g18570/At4g11850	-	Abiotic/biotic
AT4G24180	TLP	At5g03310/At3g25190/ At2g47560/At3g13760	-	-	-	abiotic
AT4G36000	TLP	-	-	At2g03360/At4g01890	-	Hormone response/biotic
AT4G36010	TLP	At3g50260/At3g04010/ At5g63370/At4g35985/ At3g59350/At4g18280/ 246178_s_at/At5g17850/ At1g11960/At1g09950/ At1g02270/At2g23340/ At2g17840/At3g10300/ At1g20450/At1g75860	At1g01470/At1g16850At1g20450/ At1g51090	At5g23850/	-	Abiotic/biotic
AT4G38660	TLP	At1g64450/At3g49670/ At1g70710/At2g27810/ At1g18650/At1g68400/ At1g74690/At5g67200/ At5g65700/At2g05790/ At3g08680/At3g17840/ At3g56370/At5g51560/ At3g53190	At3g15680/At3g56370/At5g51560	At2g05920/At3g49670/ At5g58480/At3g19820/ At1g70710/At5g55730/ At4g29360/At1g77630/ At1g74690/At5g65700/ At5g51560	-	Abiotic/biotic
AT4G38670	TLP	At3g05100/At3g54720/ At2g24150	-	At3g20070	-	abiotic
AT5G40020	TLP	At1g20850/At3g62020/ At3g16920/At5g19870/ At1g43790/At1g32100/ At3g59690/At4g08160	At2g38080/At3g16920265174_s_at/ At4g35350	-	At1g24030/At3g62160/At5g60720/ At1g43790	Abiotic/biotic
AT4G11650.1	OLP	At1g73260/At5g43580/ At3g01420/At5g17330/ At1g76930/At5g63600/ At5g44380/At1g70850/ At1g18980/267053_s_at/ 256994_s_at/At2g18370/At2g01520	At3g12500/At1g02220/ At3g01420/At3g60140/ At1g55020 At2g14620/At3g21500	At3g12500/At4g16260/At3g04720	At3g12500/At1g73260At5g43580/ At4g16260 265920_s_at/ At3g09220/At4g23700/ At3g04720/At2g45220/ At4g05200/At2g43510	Abiotic/biotic
AT2G28790.1	OLP	At3g08770/At1g18250/ At3g06030/At1g75640/	-	At5g28640	-	abiotic

Coexpressed genes were selected based on Mutual Rank (MR) < 10.

Interestingly, to some extent, the results of coexpressed analysis were confirmed by the results of promoter analysis. As example, we found fungal and salt response elements on At4g11650 promoter, and in the same line, coexpressed analysis proved the dual expressions of At4g11650 and its associated genes in both biotic and abiotic microarray experiments. This finding suggests that coexpressed gene selected by MR index can be used to justify the activation of in *silico* discovered promoter regulatory elements (TFBs) and uncovering the different functions of genes.

### Domains and prosite analysis

**Figure 3:**
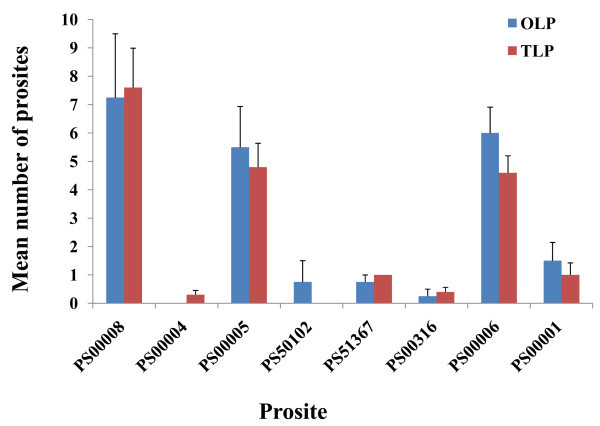
**Prosite comparison between TLP and OLP protein sequences.** PS00008: MYRISTYL N-myristoylation site, PS00004: CAMP_PHOSPHO_SITE cAMP- and cGMP-dependent protein kinase phosphorylation site, PS50102: RRM Eukaryotic RNA Recognition Motif (RRM) profile, PS51367: THAUMATIN_2 Thaumatin family profile, PS00316: THAUMATIN_1 Thaumatin family signatur, PS00006: CK2_PHOSPHO_SITE Casein kinase II phosphorylation site, PS00001: ASN_GLYCOSYLATION N-glycosylation site, PS00009: AMIDATION Amidation site, PS51257: PROKAR_LIPOPROTEIN Prokaryotic membrane lipoprotein lipid attachment site profil.

Difference in the function of genes can be tracked in their coding sequences (which results in different protein structures) or in the promoter region (which results in different protein structure). In this part of study, domains and prosite of OLPs and TLPs homologs were extracted and compared. Domain analysis did not result in distinct differences between TLPs and OLPs as domain did not found in the majority of sequences ( Additional file 2). Interestingly, prosite assay resulted in distinct differences between salt and fungal homologs (Figure 3, Additional file 3). Figure 3 shows that some prosites have different distributions between TLP and OLP. CK2_PHOSPHO_SITE Casein kinase II phosphorylation site (PS00006), PKC_PHOSPHO_SITE Protein kinase C phosphorylation site (PS00005), and ASN_GLYCOSYLATION N-glycosylation site (PS00001) are more abundant in OLP compared to TLP homologs (Figure 3).In contrast, THAUMATIN_2 Thaumatin family profile (PS51367) and CAMP_PHOSPHO_SITE cAMP- and cGMP-dependent protein kinase phosphorylation site (PS00004) are more frequent in TLP homologs (Figure 3). It can be concluded that differences in gene function in protein level can be traced in prosites which are biologically significant short sequences in comparison to domains. It should be noted that changing or adding domains (larger organization) needs more energy than prosite alteration.

### Comparative multivariate analysis of promoter regulatory elements and prosite elements of TLP and OLP homologs

**Figure 4:**
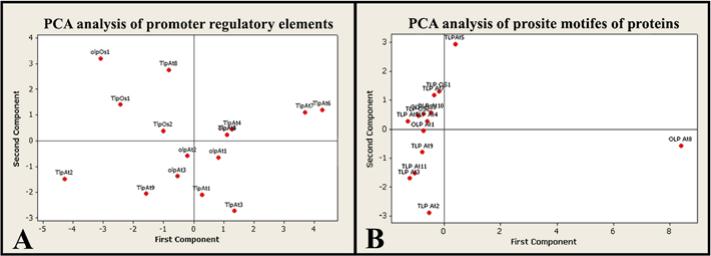
**Principle component analysis of TLP and OLP genes based on promoter regulatory elements and prosite signature of protein sequences.** TLPAt1: (TLP) (AT1G75030), TLPAt2: (TLP) (AT1G18250), TLPAt3: (TLP) AT1G73620, TLPAt4: (TLP) AT1G77700, TLPAt5: (TLP) AT4G36010, TLPAt6: (TLP) AT4G38660.1, TLPAt7: (TLP) AT4G38660.2, TLPAt8: (TLP) AT5G02140, TLPAt9: (TLP) AT5G40020, TLPOs1: (TLP) Os10g0412700, TLPOs2: (TLP) Os04g0689900, OLPAt1: (OLP) AT2G28790, OLPAt2: (OLP) (AT1G11650), OLPAt3: (OLP) AT1G75800, OLPOs1: (OLP) (Os01g0839900).

Using TFBs as variables, principle component analysis (PCA) carried out to find underling dimensions of promoter regulatory elements of TLP and OLP homologs. The first two principle components accounted for the 44.7% of variation in data. The formula of the first and second components are presented here:



At first component, the abiotic TFBs do not have significant coefficients, while in the second component, abiotic TFBs have significant coefficients. As a result, it can be concluded that the first component is presenting the biotic regulatory elements, and the second component is presenting the abiotic components. As example, Os01g0839900 which does not carry biotic TFBs has low value of first component and high value of the second component (Figure 4). On the other hand, AT5g40020 which has acceptable level of the first and second components (Figure 4) has both biotic and abiotic response elements (Table 2) and has the best promoter architecture for biotic and abiotic defense mechanisms. Interestingly, the result of our coexpression analysis based on MR index confirmed the expression of AT5g40020 in both biotic and abiotic stresses (Table 3). We suggest that principle component analysis can efficiently be used for promoter-based gene selection in future studies.

In the next part of study, Discriminant Function Analysis (DFA) carried out to estimate models for separation of TLPs from OLPs based on TFBs of promoter regions. The following models were developed based on biotic promoter regulatory elements (Table 1). As it can be inferred from the following formula, TLPs and OLPs have apparent different coefficients in WBXF and L1BX elements. In other words, WBXF and L1BX are main TFBs distinguishing specific TLPs from specific OLPs.



The mean value for discriminant value for TLP was -53.2, while this value was -28.6 for OLP homologs. Similar to PCA, Discriminant Function Analysis is a valuable technique, since the genes with intermediate values can pe proposed as genes with dual functional roles.

Figure 4 compares classification of TLPs and OLPs based on both promoter regulatory elements and prosite motifs of proteins. As it can be inferred from Figure 4, promoter elements are more variable than prosite elements. It can be concluded that promoter elements play more key role in differentiation of TLPs from OLPs and assigning gene functions to a gene.

### Importance of promoter elements in the success of genetic transformation

Commonly, in genetic transformation procedure, after cloning the gene, general promoters such as 35 S are used. However, regarding the key role of promoter for proper function, a special attention should be paid to cloning and transformation of outstanding promoter as well as gene to obtain satisfactory result we suggest that in new transformation activities a. As example, ( [Kim et al., 2008]) observed that seed-specific promoter is prerequisite for proper function of fatty acid desaturase genes in altering the unsaturated fatty acid content of oilseeds by genetic manipulation expression ( [Kim et al. 2008]).

Up to now, the majority of researchers just considered individual gene to predict gene function. The approach employed in this research considering coexpressed gene with gene of interest and promoter analysis, as well as illustrating prosite structure can result in reveal valuable findings about protein function in different pathway. In particular, the unique regulatory elements (responding to different sorts of stresses) open a new avenue in genetic engineering trough manipulating of cis-acting regulatory elements on promoter region.

## Conclusion

Here, for the first time, we demonstrated that promoter analysis of TLPs and OLPs can explain multiple roles of TLPs and OLPs in biotic and abiotic stresses. In addition, we showed that analysis of coexpressed genes with gene of interest analysis can provide valuable insight in dtertmination of diverse role of genes. In conclusion, our results revealed that, new computational tools such as coexpressed gene analysis, cis regulatory analysis and *in silico* protein analysis can identify the outstanding TLPs and OLPs homologue involving in response to biotic and abiotic stresses. Discovering the genes with dual resistance functions in biotic and abiotic stresses is a major advance in genetic transformation. Furthermore, the present methods can be efficiently employed in discovering the unknown function of genes.

## Material and methods

### Promoter analysis

Genome-wide collection of all genes encoding OLPs (acting against salt stress) (AT1G75800, AT2G28790, AT4G36010, ATOSM34 or AT4G11650.1, Os01g0839900) and TLPs (acting against fungal stress) (AT1G73620, AT1G77700, AT4G36010, AT4G38660.1, AT4G38660.2, AT5G02140, AT5G40020, AT1G18250, AT1G75030, OS04G0689900, Os10g0412700) in the Arabidopsis and Rice genomes carried out using Genomatix ( http://www.genomatix.de/en/index.html) and TAIR ( http://www.arabidopsis.org/) databases.

Cis-acting regulatory elements of each group of TLPs and OLPs were recognized by *in silico* promoter analysis using Genomatix ( http://www.genomatix.de/en/index.html) and PlantCARE ( http://bioinformatics.psb.ugent.be/webtools/plantcare/html/) databases.

To highlight the roles of specific TFBS in promoter activity, the general core promoter elements (such as TATA-box) were disregarded. The number and position of promoter regulatory elements, particularly hormonal, biotic and abiotic ones were compared between TLPs and OLPs.

### Coexpressed genes analysis

All TLPs and OLPs locuses of *Arabidopsis thaliana* has been selected from TAIR database ( http://www.arabidopsis.org). In order to analyze the coexpressed gene we used ATTED-II ( http://atted.jp) was used. This database collects gene expression data in Arabidopsis from a wide range of microarray experiments. Three hundered coexpressed genes by each TLP and OLP locus were extracted from abiotic, biotic, hormone and light experiments in this database. To avoid discarding potentially important coexpressed gene pairs having low Pearson’s correlation coefficient (PCCs), ATTED-II employs a new measure of gene coexpression, Mutual Rank (MR). Correlation rank is asymmetric, namely the rank of gene B from gene A is not the same as the rank of gene A from gene B. And thus, those two ranks are geometrically averaged, which we call Mutual Rank (MR). MR(AB) = √ (Rank(A → B) x Rank(B → A)).

For any given pair, gene A and gene B, the MR is calculated as an average of the rank of gene B in the coexpressed genes to gene A and the average of the rank of gene A to gene B. We selected the coexpressed gene in each experiment by MR < 10 ( Additional file 1, Table 3).

### Domains and prosites

In order to investigate all TLPs and OLPs protein structure, domains and prosites identification were applied. All 14 protein sequences of TLPs and OLPs (10 TLPs, and 4 OLPs) extracted from NCBI ( http://www.ncbi.nlm.nih.gov/). Protein domains have been extracted from pfam database ( http://pfam.sanger.ac.uk/) and prosites from NPS (PROSCAN) database ( http://npsa-pbil.ibcp.fr/cgi-bin/npsa_automat.pl?page=/NPSA/npsa_proscan.html).

### Multivariate analysis

Principle Component Analysis and Discriminant Function Analysis by Minitab 16 package ( http://www.minitab.com/). For performing the above mentioned analysis, different promoter regulatory elements and prosite motifs were used as variables (Table 1 and Additional file 3).

## Electronic supplementary material

Additional file 1: **Top 300 coexpressed genes to different isoforms of TLPs and OLPs in*Arabidopsis thaliana*through different experiment such as tissue, abiotic, biotic, hormone and light.** Lower Mutual Rank (MR) between genes shows higher correlation in expression. (DOCX 1 MB)

Additional file 2: **Domain analysis of TLPs and OLPs homologs result.** (XLSX 10 KB)

Additional file 3: **Prosite analysis of TLP and OLP homologs.** (XLSX 10 KB)

## Abbreviations

TLP: Thaumatin like protein
PR proteins: Pathogenesis related proteins
OLP: Osmotin like protein
TFBS: Transcription factor binding site
MR: Mutual rank.
