# Supplementary material for: Finding the undiscovered roles of genes: an approach using mutual ranking of coexpressed genes and promoter architecture-case study: dual roles of thaumatin like proteins in biotic and abiotic stresses
Source: Springerplus. 2012 Oct 5;1:30. doi: 10.1186/2193-1801-1-30 (PMC3725900; doi:10.1186/2193-1801-1-30)
Supplement: Supplementary file 1 — Additional file 1: Top 300 coexpressed genes to different isoforms of TLPs and OLPs inArabidopsis thalianathrough different experiment such as tissue, abiotic, biotic, hormone and light. Lower Mutual Rank (MR) between genes shows higher correlation in expression. (DOCX 1 MB) [file 40064_2012_20_MOESM1_ESM.docx]

Additional file 1

Top 300 coexpressed genes to different isoforms of TLPs and OLPs in *Arabidopsis thaliana* through different experiment such as tissue, abiotic, biotic, hormone and light. Lower Mutual Rank (MR) between genes shows higher correlation in expression.

| **300 coexpressed gene with At1g73620** | | | | | | | | | | | |
| --- | --- | --- | --- | --- | --- | --- | --- | --- | --- | --- | --- |
|  | | **locus** | **Short description** | | | **MR^**^**  **(all)** | **MR**  **(tissue)** | **MR**  **(abiotic)** | **MR**  **(biotic)** | **MR**  **(hormone)** | **MR**  **(light)** |
| 1 | | [At5g13520](http://atted.jp/cgi-bin/coex_Ath.cgi?gene=At5g13520&sort=all) | peptidase M1 | | | **5.0** | 12.5 | 292.0 | 394.1 | 1380.2 | 1869.5 |
| 2 | | [At2g20515](http://atted.jp/cgi-bin/coex_Ath.cgi?gene=At2g20515&sort=all) |  | | | **6.9** | 355.5 | 20.5 | 8583.5 | 7.1 | 10182.0 |
| 3 | | [At3g43960](http://atted.jp/cgi-bin/coex_Ath.cgi?gene=At3g43960&sort=all) | cysteinease | | | **8.4** | 8.5 | 400.9 | 3283.9 | 47.7 | 19384.0 |
| 4 | | [At5g43020](http://atted.jp/cgi-bin/coex_Ath.cgi?gene=At5g43020&sort=all) | kinase | | | **10.1** | 154.0 | 21.4 | 1260.3 | 729.2 | 9925.4 |
| 5 | | [At4g26760](http://atted.jp/cgi-bin/coex_Ath.cgi?gene=At4g26760&sort=all) | MAP65-2 | | | **12.4** | 128.0 | 60.9 | 5796.7 | 23.2 | 14424.0 |
| 6 | | [At5g01870](http://atted.jp/cgi-bin/coex_Ath.cgi?gene=At5g01870&sort=all) | lipid transfer | | | **12.8** | 73.7 | 1229.7 | 1284.6 | 777.7 | 14775.9 |
| 7 | | [At1g63650](http://atted.jp/cgi-bin/coex_Ath.cgi?gene=At1g63650&sort=all) | EGL3 | | | **13.2** | 145.0 | 67.3 | 2323.3 | 551.5 | 8974.9 |
| 8 | | [At5g48360](http://atted.jp/cgi-bin/coex_Ath.cgi?gene=At5g48360&sort=all) | FH2 | | | **18.1** | 215.8 | 71.2 | 1022.9 | 5931.5 | 6968.1 |
| 9 | | [At4g39860](http://atted.jp/cgi-bin/coex_Ath.cgi?gene=At4g39860&sort=all) |  | | | **23.8** | 360.9 | 146.5 | 4451.1 | 1942.5 | 20954.3 |
| 10 | | [At1g07790](http://atted.jp/cgi-bin/coex_Ath.cgi?gene=At1g07790&sort=all) | HTB1 | | | **24.2** | 72.3 | 81.1 | 3403.9 | 350.7 | 9016.2 |
| 11 | | [At4g36180](http://atted.jp/cgi-bin/coex_Ath.cgi?gene=At4g36180&sort=all) | LRR | | | **24.7** | 222.7 | 205.6 | 3079.3 | 60.4 | 9084.8 |
| 12 | | [At2g20480](http://atted.jp/cgi-bin/coex_Ath.cgi?gene=At2g20480&sort=all) |  | | | **26.3** | 45.5 | 750.8 | 10637.3 | 640.5 | 1668.0 |
| 13 | | [At3g19590](http://atted.jp/cgi-bin/coex_Ath.cgi?gene=At3g19590&sort=all) | WD-40 repeat | | | **28.1** | 152.7 | 41.0 | 408.5 | 2343.7 | 16143.8 |
| 14 | | [At3g14190](http://atted.jp/cgi-bin/coex_Ath.cgi?gene=At3g14190&sort=all) |  | | | **30.4** | 67.1 | 45.2 | 43.8 | 781.2 | 9512.0 |
| 15 | | [At2g37080](http://atted.jp/cgi-bin/coex_Ath.cgi?gene=At2g37080&sort=all) | RIP3 | | | **32.0** | 273.1 | 302.6 | 6039.0 | 1412.7 | 6645.1 |
| 16 | | [At3g20260](http://atted.jp/cgi-bin/coex_Ath.cgi?gene=At3g20260&sort=all) | ribosome | | | **33.8** | 311.8 | 444.2 | 4487.1 | 1141.2 | 21390.8 |
| 17 | | [At2g28790](http://atted.jp/cgi-bin/coex_Ath.cgi?gene=At2g28790&sort=all) | osmotin-like | | | **34.2** | 453.5 | 123.9 | 4733.6 | 1210.9 | 11439.6 |
| 18 | | [At1g10850](http://atted.jp/cgi-bin/coex_Ath.cgi?gene=At1g10850&sort=all) | kinase | | | **34.8** | 140.8 | 542.0 | 712.7 | 1645.7 | 6194.1 |
| 19 | | [At4g22250](http://atted.jp/cgi-bin/coex_Ath.cgi?gene=At4g22250&sort=all) | zinc finger | | | **34.8** | 137.7 | 656.3 | 4760.3 | 8605.4 | 11882.0 |
| 20 | | [At3g54250](http://atted.jp/cgi-bin/coex_Ath.cgi?gene=At3g54250&sort=all) | decarboxylase | | | **35.5** | 142.6 | 587.1 | 2951.8 | 1715.3 | 17321.2 |
| 21 | | [At3g17160](http://atted.jp/cgi-bin/coex_Ath.cgi?gene=At3g17160&sort=all) |  | | | **37.1** | 225.6 | 125.7 | 13541.8 | 1058.8 | 19582.2 |
| 22 | | [At3g22790](http://atted.jp/cgi-bin/coex_Ath.cgi?gene=At3g22790&sort=all) | kinase interacting | | | **38.8** | 546.1 | 76.0 | 2519.6 | 2518.9 | 17199.4 |
| 23 | | [At5g67200](http://atted.jp/cgi-bin/coex_Ath.cgi?gene=At5g67200&sort=all) | kinase | | | **40.4** | 200.6 | 43.8 | 5689.7 | 490.3 | 5728.3 |
| 24 | | [At5g48230](http://atted.jp/cgi-bin/coex_Ath.cgi?gene=At5g48230&sort=all) | ACAT2 | | | **43.0** | 86.3 | 78.2 | 9253.9 | 2833.2 | 14984.0 |
| 25 | | [At2g34190](http://atted.jp/cgi-bin/coex_Ath.cgi?gene=At2g34190&sort=all) | permease | | | **44.6** | 566.5 | 29.7 | 113.8 | 804.8 | 7983.2 |
| 26 | | [At5g51750](http://atted.jp/cgi-bin/coex_Ath.cgi?gene=At5g51750&sort=all) | SBT1.3 | | | **45.2** | 268.6 | 223.4 | 2570.4 | 768.5 | 9562.3 |
| 27 | | [At1g14900](http://atted.jp/cgi-bin/coex_Ath.cgi?gene=At1g14900&sort=all) | HMGA | | | **47.0** | 45.1 | 2429.7 | 8933.7 | 872.4 | 9841.9 |
| 28 | | [At2g17560](http://atted.jp/cgi-bin/coex_Ath.cgi?gene=At2g17560&sort=all) | HMGB4 | | | **47.8** | 97.7 | 170.4 | 18656.8 | 5728.7 | 22018.1 |
| 29 | | [At1g18250](http://atted.jp/cgi-bin/coex_Ath.cgi?gene=At1g18250&sort=all) | LP-1 | | | **49.6** | 773.0 | 13.6 | 1300.7 | 218.1 | 11489.4 |
| 30 | | 253340_s_at |  | | | **50.0** | 309.7 | 85.5 | 2819.1 | 3255.3 | 11156.7 |
| 31 | | [At3g08770](http://atted.jp/cgi-bin/coex_Ath.cgi?gene=At3g08770&sort=all) | LTP6 | | | **50.8** | 329.9 | 195.0 | 2344.7 | 682.7 | 6216.6 |
| 32 | | [At1g21090](http://atted.jp/cgi-bin/coex_Ath.cgi?gene=At1g21090&sort=all) | glycoprotein | | | **55.9** | 429.6 | 202.6 | 3322.7 | 33.1 | 14023.5 |
| 33 | | [At4g31360](http://atted.jp/cgi-bin/coex_Ath.cgi?gene=At4g31360&sort=all) | selenium binding | | | **58.9** | 313.8 | 214.3 | 6461.7 | 6966.7 | 21337.3 |
| 34 | | [At1g72730](http://atted.jp/cgi-bin/coex_Ath.cgi?gene=At1g72730&sort=all) | translation | | | **60.6** | 142.5 | 52.6 | 10218.2 | 857.6 | 14213.7 |
| 35 | | [At3g23890](http://atted.jp/cgi-bin/coex_Ath.cgi?gene=At3g23890&sort=all) | TOPII | | | **62.4** | 314.9 | 93.1 | 2910.2 | 538.5 | 9871.5 |
| 36 | | [At3g13674](http://atted.jp/cgi-bin/coex_Ath.cgi?gene=At3g13674&sort=all) |  | | | **63.6** | 356.0 | 77.5 | 6073.2 | 302.0 | 14454.7 |
| 37 | | [At3g08680](http://atted.jp/cgi-bin/coex_Ath.cgi?gene=At3g08680&sort=all) | kinase | | | **64.0** | 209.3 | 162.5 | 5989.8 | 1261.8 | 5380.9 |
| 38 | | [At1g10780](http://atted.jp/cgi-bin/coex_Ath.cgi?gene=At1g10780&sort=all) | F-box | | | **64.2** | 549.6 | 71.7 | 855.1 | 982.8 | 15628.5 |
| 39 | | [At2g42110](http://atted.jp/cgi-bin/coex_Ath.cgi?gene=At2g42110&sort=all) |  | | | **64.6** | 431.0 | 69.2 | 2054.5 | 514.9 | 7625.4 |
| 40 | | [At3g50070](http://atted.jp/cgi-bin/coex_Ath.cgi?gene=At3g50070&sort=all) | CYCD3;3 | | | **65.0** | 340.9 | 492.7 | 3785.9 | 18.4 | 13365.1 |
| 41 | | [At4g15830](http://atted.jp/cgi-bin/coex_Ath.cgi?gene=At4g15830&sort=all) | binding | | | **66.5** | 538.6 | 20.9 | 2084.4 | 606.2 | 13305.3 |
| 42 | | [At1g07370](http://atted.jp/cgi-bin/coex_Ath.cgi?gene=At1g07370&sort=all) | PCNA1 | | | **71.5** | 342.4 | 41.3 | 6255.7 | 1532.9 | 7889.4 |
| 43 | | [At4g17190](http://atted.jp/cgi-bin/coex_Ath.cgi?gene=At4g17190&sort=all) | FPS2 | | | **72.6** | 251.9 | 392.5 | 9034.0 | 1954.8 | 21313.3 |
| 44 | | [At5g50375](http://atted.jp/cgi-bin/coex_Ath.cgi?gene=At5g50375&sort=all) | CPI1 | | | **73.2** | 318.9 | 115.5 | 6722.9 | 4.0 | 15147.6 |
| 45 | | [At3g29280](http://atted.jp/cgi-bin/coex_Ath.cgi?gene=At3g29280&sort=all) |  | | | **74.1** | 124.1 | 86.4 | 2599.9 | 1206.2 | 5447.5 |
| 46 | | [At1g75710](http://atted.jp/cgi-bin/coex_Ath.cgi?gene=At1g75710&sort=all) | zinc finger | | | **75.6** | 89.9 | 1489.9 | 5431.6 | 998.5 | 2371.6 |
| 47 | | [At4g25760](http://atted.jp/cgi-bin/coex_Ath.cgi?gene=At4g25760&sort=all) | GDU2 | | | **75.7** | 50.8 | 2430.2 | 5580.2 | 919.7 | 5242.4 |
| 48 | | [At2g42570](http://atted.jp/cgi-bin/coex_Ath.cgi?gene=At2g42570&sort=all) |  | | | **75.8** | 315.5 | 744.8 | 4856.9 | 50.0 | 12740.6 |
| 49 | | [At3g03130](http://atted.jp/cgi-bin/coex_Ath.cgi?gene=At3g03130&sort=all) |  | | | **77.0** | 712.0 | 9.2 | 3376.7 | 59.1 | 9128.6 |
| 50 | | [At3g61490](http://atted.jp/cgi-bin/coex_Ath.cgi?gene=At3g61490&sort=all) | pectinase | | | **77.3** | 88.4 | 455.2 | 16836.3 | 189.1 | 7948.4 |
| 51 | | [At5g45670](http://atted.jp/cgi-bin/coex_Ath.cgi?gene=At5g45670&sort=all) | hydrolase | | | **78.0** | 270.0 | 247.4 | 337.5 | 9359.5 | 17931.1 |
| 52 | | [At1g17560](http://atted.jp/cgi-bin/coex_Ath.cgi?gene=At1g17560&sort=all) | HLL | | | **78.5** | 119.6 | 291.8 | 5351.3 | 3435.2 | 11939.1 |
| 53 | | [At4g28310](http://atted.jp/cgi-bin/coex_Ath.cgi?gene=At4g28310&sort=all) |  | | | **78.9** | 463.7 | 45.9 | 6751.1 | 2119.8 | 2502.9 |
| 54 | | [At2g38810](http://atted.jp/cgi-bin/coex_Ath.cgi?gene=At2g38810&sort=all) | HTA8 | | | **80.7** | 189.5 | 330.8 | 8267.3 | 2052.0 | 17716.7 |
| 55 | | [At1g25510](http://atted.jp/cgi-bin/coex_Ath.cgi?gene=At1g25510&sort=all) | protease | | | **81.3** | 404.4 | 122.0 | 6881.3 | 895.9 | 4808.6 |
| 56 | | [At5g26850](http://atted.jp/cgi-bin/coex_Ath.cgi?gene=At5g26850&sort=all) |  | | | **82.8** | 133.9 | 519.3 | 3700.0 | 11597.5 | 18614.4 |
| 57 | | [At3g25220](http://atted.jp/cgi-bin/coex_Ath.cgi?gene=At3g25220&sort=all) | FKBP15-1 | | | **85.0** | 91.0 | 464.9 | 19129.8 | 5067.4 | 22245.7 |
| 58 | | [At5g02550](http://atted.jp/cgi-bin/coex_Ath.cgi?gene=At5g02550&sort=all) |  | | | **85.1** | 79.5 | 1273.3 | 6779.0 | 15353.8 | 18612.5 |
| 59 | | [At1g18370](http://atted.jp/cgi-bin/coex_Ath.cgi?gene=At1g18370&sort=all) | HIK | | | **85.2** | 631.5 | 54.0 | 3501.6 | 1563.6 | 11724.2 |
| 60 | | [At2g19170](http://atted.jp/cgi-bin/coex_Ath.cgi?gene=At2g19170&sort=all) | SLP3 | | | **86.4** | 340.4 | 510.0 | 918.5 | 1962.5 | 10857.8 |
| 61 | | [At4g38660](http://atted.jp/cgi-bin/coex_Ath.cgi?gene=At4g38660&sort=all) | thaumatin | | | **87.5** | 465.6 | 112.1 | 4177.4 | 1355.1 | 7976.9 |
| 62 | | [At4g33400](http://atted.jp/cgi-bin/coex_Ath.cgi?gene=At4g33400&sort=all) | dem-related | | | **88.2** | 236.9 | 117.4 | 5209.8 | 1613.4 | 19502.9 |
| 63 | | [At1g44110](http://atted.jp/cgi-bin/coex_Ath.cgi?gene=At1g44110&sort=all) | CYCA1;1 | | | **88.5** | 432.5 | 88.7 | 1951.8 | 47.6 | 2087.7 |
| 64 | | [At3g20015](http://atted.jp/cgi-bin/coex_Ath.cgi?gene=At3g20015&sort=all) | endopeptidase | | | **92.0** | 378.9 | 672.5 | 3368.9 | 4.2 | 7046.4 |
| 65 | | 258121_s_at |  | | | **92.0** | 173.0 | 627.4 | 10717.1 | 6175.7 | 1664.8 |
| 66 | | [At4g23800](http://atted.jp/cgi-bin/coex_Ath.cgi?gene=At4g23800&sort=all) | HMG1/2 | | | **93.5** | 388.4 | 98.2 | 1986.1 | 1251.8 | 13497.2 |
| 67 | | [At3g25980](http://atted.jp/cgi-bin/coex_Ath.cgi?gene=At3g25980&sort=all) | MAD2 | | | **95.5** | 468.8 | 104.4 | 505.7 | 239.3 | 6341.4 |
| 68 | | [At4g00820](http://atted.jp/cgi-bin/coex_Ath.cgi?gene=At4g00820&sort=all) | iqd17 | | | **96.2** | 500.1 | 295.1 | 1764.3 | 4450.2 | 21212.7 |
| 69 | | [At5g62210](http://atted.jp/cgi-bin/coex_Ath.cgi?gene=At5g62210&sort=all) | embryo-specific | | | **96.5** | 210.5 | 691.0 | 4041.3 | 36.8 | 3425.3 |
| 70 | | [At5g08580](http://atted.jp/cgi-bin/coex_Ath.cgi?gene=At5g08580&sort=all) | calcium-binding EF hand | | | **97.5** | 251.8 | 332.5 | 6491.1 | 1044.0 | 19091.2 |
| 71 | | [At2g07690](http://atted.jp/cgi-bin/coex_Ath.cgi?gene=At2g07690&sort=all) | MCM5 | | | **99.1** | 333.7 | 113.3 | 6332.3 | 8718.6 | 5876.6 |
| 72 | | [At5g50740](http://atted.jp/cgi-bin/coex_Ath.cgi?gene=At5g50740&sort=all) | metal ion binding | | | **100.4** | 663.2 | 118.4 | 488.6 | 3272.4 | 1972.8 |
| 73 | | [At5g25090](http://atted.jp/cgi-bin/coex_Ath.cgi?gene=At5g25090&sort=all) | plastocyanin-like | | | **101.0** | 789.0 | 42.2 | 132.7 | 174.8 | 12303.4 |
| 74 | | [At1g05210](http://atted.jp/cgi-bin/coex_Ath.cgi?gene=At1g05210&sort=all) |  | | | **108.0** | 402.2 | 193.7 | 3503.9 | 369.3 | 12635.2 |
| 75 | | [At3g53190](http://atted.jp/cgi-bin/coex_Ath.cgi?gene=At3g53190&sort=all) | lyase | | | **108.5** | 815.5 | 115.2 | 2769.1 | 843.5 | 3990.5 |
| 76 | | [At5g13840](http://atted.jp/cgi-bin/coex_Ath.cgi?gene=At5g13840&sort=all) | FZR3 | | | **109.1** | 439.6 | 113.0 | 8298.7 | 6635.5 | 10828.1 |
| 77 | | [At1g35780](http://atted.jp/cgi-bin/coex_Ath.cgi?gene=At1g35780&sort=all) |  | | | **109.2** | 254.6 | 344.5 | 3438.5 | 5846.8 | 14872.1 |
| 78 | | [At1g74030](http://atted.jp/cgi-bin/coex_Ath.cgi?gene=At1g74030&sort=all) | ENO1 | | | **109.4** | 458.9 | 94.2 | 11922.1 | 546.5 | 12713.3 |
| 79 | | [At5g17160](http://atted.jp/cgi-bin/coex_Ath.cgi?gene=At5g17160&sort=all) |  | | | **109.6** | 499.1 | 52.3 | 1481.5 | 111.8 | 11859.6 |
| 80 | | [At2g16440](http://atted.jp/cgi-bin/coex_Ath.cgi?gene=At2g16440&sort=all) | MCM4 | | | **110.2** | 331.4 | 281.7 | 5759.1 | 4575.1 | 1731.5 |
| 81 | | 262939_s_at |  | | | **112.9** | 233.2 | 1896.4 | 16200.2 | 7813.4 | 19804.1 |
| 82 | | 266401_s_at |  | | | **115.2** | 571.3 | 149.5 | 2712.4 | 2779.1 | 8096.7 |
| 83 | | [At1g53140](http://atted.jp/cgi-bin/coex_Ath.cgi?gene=At1g53140&sort=all) | DRP5A | | | **115.2** | 584.1 | 15.6 | 310.7 | 7859.0 | 5624.2 |
| 84 | | [At3g56370](http://atted.jp/cgi-bin/coex_Ath.cgi?gene=At3g56370&sort=all) | kinase | | | **115.6** | 254.3 | 120.2 | 5022.6 | 978.6 | 3429.8 |
| 85 | | [At3g06030](http://atted.jp/cgi-bin/coex_Ath.cgi?gene=At3g06030&sort=all) | ANP3 | | | **118.3** | 707.4 | 80.5 | 4797.4 | 5050.4 | 10910.4 |
| 86 | | [At3g20150](http://atted.jp/cgi-bin/coex_Ath.cgi?gene=At3g20150&sort=all) | kinesin motor | | | **121.9** | 787.0 | 39.3 | 213.2 | 5803.9 | 17946.5 |
| 87 | | [At5g46280](http://atted.jp/cgi-bin/coex_Ath.cgi?gene=At5g46280&sort=all) | MCM3 | | | **122.0** | 376.0 | 95.4 | 7208.3 | 3807.7 | 18564.6 |
| 88 | | [At1g09200](http://atted.jp/cgi-bin/coex_Ath.cgi?gene=At1g09200&sort=all) | histone H3 | | | **122.2** | 290.9 | 130.2 | 6388.2 | 1517.7 | 18010.3 |
| 89 | | 251331_s_at |  | | | **124.3** | 205.5 | 192.2 | 7130.1 | 2311.9 | 18266.0 |
| 90 | | [At5g06050](http://atted.jp/cgi-bin/coex_Ath.cgi?gene=At5g06050&sort=all) | dehydration-responsive | | | **124.6** | 164.4 | 181.0 | 4337.1 | 2386.8 | 21329.1 |
| 91 | | [At3g55660](http://atted.jp/cgi-bin/coex_Ath.cgi?gene=At3g55660&sort=all) | ROPGEF6 | | | **125.0** | 986.2 | 33.8 | 509.1 | 8010.9 | 2639.3 |
| 92 | | [At3g48410](http://atted.jp/cgi-bin/coex_Ath.cgi?gene=At3g48410&sort=all) | hydrolase | | | **125.5** | 572.9 | 2334.3 | 5075.0 | 16.4 | 8424.8 |
| 93 | | [At3g14740](http://atted.jp/cgi-bin/coex_Ath.cgi?gene=At3g14740&sort=all) | PHD finger | | | **126.1** | 676.7 | 34.3 | 9666.9 | 5100.4 | 18615.0 |
| 94 | | [At3g51290](http://atted.jp/cgi-bin/coex_Ath.cgi?gene=At3g51290&sort=all) | proline-rich | | | **126.9** | 641.1 | 119.3 | 914.4 | 2338.8 | 20049.7 |
| 95 | | [At2g43360](http://atted.jp/cgi-bin/coex_Ath.cgi?gene=At2g43360&sort=all) | BIO2 | | | **127.3** | 495.7 | 36.2 | 1863.6 | 799.0 | 18422.6 |
| 96 | | [At1g72250](http://atted.jp/cgi-bin/coex_Ath.cgi?gene=At1g72250&sort=all) | kinesin motor | | | **127.3** | 726.8 | 90.0 | 5130.8 | 729.4 | 4428.6 |
| 97 | | [At4g11820](http://atted.jp/cgi-bin/coex_Ath.cgi?gene=At4g11820&sort=all) | MVA1 | | | **127.9** | 209.0 | 333.6 | 13408.7 | 1101.4 | 17771.4 |
| 98 | | [At1g49580](http://atted.jp/cgi-bin/coex_Ath.cgi?gene=At1g49580&sort=all) | CDPK | | | **128.1** | 149.9 | 1337.6 | 802.9 | 1152.2 | 20630.1 |
| 99 | | [At4g37490](http://atted.jp/cgi-bin/coex_Ath.cgi?gene=At4g37490&sort=all) | CYCB1;1 | | | **128.4** | 154.9 | 544.0 | 7776.7 | 306.1 | 16874.9 |
| 100 | | 260118_s_at |  | | | **129.1** | 1018.0 | 5.9 | 1486.6 | 4038.0 | 8277.7 |
| 101 | | [At5g17070](http://atted.jp/cgi-bin/coex_Ath.cgi?gene=At5g17070&sort=all) |  | | | **129.6** | 110.7 | 276.4 | 5214.5 | 6201.0 | 11475.7 |
| 102 | | [At1g32930](http://atted.jp/cgi-bin/coex_Ath.cgi?gene=At1g32930&sort=all) | transferase | | | **129.7** | 406.0 | 1014.7 | 3484.7 | 2397.1 | 18304.4 |
| 103 | | [At1g63470](http://atted.jp/cgi-bin/coex_Ath.cgi?gene=At1g63470&sort=all) | DNA-binding | | | **130.2** | 324.9 | 526.1 | 7717.1 | 5210.4 | 1728.5 |
| 104 | | [At3g11250](http://atted.jp/cgi-bin/coex_Ath.cgi?gene=At3g11250&sort=all) | RPP0C | | | **131.0** | 76.9 | 148.4 | 11779.3 | 1123.7 | 20268.0 |
| 105 | | [At3g02640](http://atted.jp/cgi-bin/coex_Ath.cgi?gene=At3g02640&sort=all) |  | | | **131.4** | 674.0 | 22.6 | 1288.0 | 637.5 | 7253.3 |
| 106 | | [At1g01370](http://atted.jp/cgi-bin/coex_Ath.cgi?gene=At1g01370&sort=all) | HTR12 | | | **131.6** | 668.4 | 129.0 | 4347.4 | 12067.0 | 3642.3 |
| 107 | | [At1g70710](http://atted.jp/cgi-bin/coex_Ath.cgi?gene=At1g70710&sort=all) | GH9B1 | | | **131.7** | 526.2 | 206.8 | 2844.4 | 4776.5 | 15253.5 |
| 108 | | [At4g02060](http://atted.jp/cgi-bin/coex_Ath.cgi?gene=At4g02060&sort=all) | PRL | | | **134.2** | 447.1 | 180.9 | 5796.7 | 4488.0 | 15551.5 |
| 109 | | [At1g69770](http://atted.jp/cgi-bin/coex_Ath.cgi?gene=At1g69770&sort=all) | CMT3 | | | **136.0** | 346.7 | 114.8 | 13137.7 | 8275.5 | 7599.4 |
| 110 | | [At3g54560](http://atted.jp/cgi-bin/coex_Ath.cgi?gene=At3g54560&sort=all) | HTA11 | | | **140.4** | 337.7 | 111.9 | 12525.7 | 2836.5 | 15105.5 |
| 111 | | [At2g25060](http://atted.jp/cgi-bin/coex_Ath.cgi?gene=At2g25060&sort=all) | plastocyanin-like | | | **140.4** | 942.8 | 38.0 | 1602.0 | 138.9 | 6645.2 |
| 112 | | [At3g25040](http://atted.jp/cgi-bin/coex_Ath.cgi?gene=At3g25040&sort=all) | ERD2B | | | **141.6** | 75.8 | 445.1 | 11141.8 | 8353.1 | 18109.9 |
| 113 | | [At2g26760](http://atted.jp/cgi-bin/coex_Ath.cgi?gene=At2g26760&sort=all) | CYCB1;4 | | | **142.4** | 360.4 | 74.5 | 1576.3 | 2436.5 | 8929.4 |
| 114 | | [At5g15530](http://atted.jp/cgi-bin/coex_Ath.cgi?gene=At5g15530&sort=all) | BCCP2 | | | **144.6** | 527.4 | 257.9 | 961.8 | 4766.8 | 12962.4 |
| 115 | | [At3g56100](http://atted.jp/cgi-bin/coex_Ath.cgi?gene=At3g56100&sort=all) | MRLK | | | **145.9** | 805.3 | 95.7 | 13520.7 | 2167.8 | 18148.4 |
| 116 | | [At4g02800](http://atted.jp/cgi-bin/coex_Ath.cgi?gene=At4g02800&sort=all) |  | | | **146.0** | 738.7 | 62.9 | 11026.1 | 1945.6 | 16402.0 |
| 117 | | [At3g24495](http://atted.jp/cgi-bin/coex_Ath.cgi?gene=At3g24495&sort=all) | MSH7 | | | **146.1** | 326.2 | 143.4 | 7724.2 | 7407.9 | 1490.0 |
| 118 | | [At2g31270](http://atted.jp/cgi-bin/coex_Ath.cgi?gene=At2g31270&sort=all) | CDT1A | | | **146.5** | 789.0 | 62.8 | 1652.3 | 6531.2 | 9513.5 |
| 119 | | [At3g22880](http://atted.jp/cgi-bin/coex_Ath.cgi?gene=At3g22880&sort=all) | DMC1 | | | **148.0** | 409.1 | 235.5 | 2573.1 | 15302.3 | 16365.8 |
| 120 | | [At1g51060](http://atted.jp/cgi-bin/coex_Ath.cgi?gene=At1g51060&sort=all) | HTA10 | | | **148.1** | 190.1 | 285.5 | 10666.6 | 619.9 | 7919.1 |
| 121 | | [At5g67270](http://atted.jp/cgi-bin/coex_Ath.cgi?gene=At5g67270&sort=all) | EB1C | | | **149.2** | 587.2 | 124.7 | 1745.8 | 980.4 | 9391.6 |
| 122 | | [At5g19340](http://atted.jp/cgi-bin/coex_Ath.cgi?gene=At5g19340&sort=all) |  | | | **150.9** | 45.3 | 9447.9 | 938.7 | 2673.5 | 12299.9 |
| 123 | | [At1g60860](http://atted.jp/cgi-bin/coex_Ath.cgi?gene=At1g60860&sort=all) | AGD2 | | | **151.7** | 265.2 | 1511.4 | 2042.4 | 1111.3 | 19916.6 |
| 124 | | [At5g55520](http://atted.jp/cgi-bin/coex_Ath.cgi?gene=At5g55520&sort=all) |  | | | **152.1** | 781.8 | 59.8 | 9483.3 | 2611.4 | 8504.4 |
| 125 | | [At1g21560](http://atted.jp/cgi-bin/coex_Ath.cgi?gene=At1g21560&sort=all) |  | | | **152.8** | 638.1 | 424.3 | 5448.9 | 370.5 | 18766.5 |
| 126 | | [At1g44900](http://atted.jp/cgi-bin/coex_Ath.cgi?gene=At1g44900&sort=all) | MCM2 | | | **154.0** | 340.2 | 178.1 | 9847.3 | 4076.6 | 17730.6 |
| 127 | | [At1g76310](http://atted.jp/cgi-bin/coex_Ath.cgi?gene=At1g76310&sort=all) | CYCB2;4 | | | **154.2** | 735.4 | 107.0 | 1965.5 | 1740.8 | 12804.4 |
| 128 | | [At2g32590](http://atted.jp/cgi-bin/coex_Ath.cgi?gene=At2g32590&sort=all) |  | | | **154.4** | 731.1 | 161.7 | 7765.6 | 1059.1 | 4466.5 |
| 129 | | [At3g12870](http://atted.jp/cgi-bin/coex_Ath.cgi?gene=At3g12870&sort=all) |  | | | **154.6** | 845.0 | 84.8 | 597.8 | 1910.1 | 3397.5 |
| 130 | | [At5g63140](http://atted.jp/cgi-bin/coex_Ath.cgi?gene=At5g63140&sort=all) | PAP29 | | | **155.7** | 345.1 | 582.2 | 960.1 | 1200.2 | 17972.6 |
| 131 | | [At1g48610](http://atted.jp/cgi-bin/coex_Ath.cgi?gene=At1g48610&sort=all) | AT hook motif | | | **155.8** | 527.2 | 139.9 | 2535.1 | 3453.2 | 5852.0 |
| 132 | | [At3g06840](http://atted.jp/cgi-bin/coex_Ath.cgi?gene=At3g06840&sort=all) |  | | | **156.2** | 468.6 | 247.6 | 1356.2 | 3904.8 | 13829.0 |
| 133 | | [At1g02690](http://atted.jp/cgi-bin/coex_Ath.cgi?gene=At1g02690&sort=all) | IMPA-6 | | | **156.2** | 428.2 | 103.0 | 10003.5 | 4181.6 | 4426.8 |
| 134 | | [At3g09730](http://atted.jp/cgi-bin/coex_Ath.cgi?gene=At3g09730&sort=all) |  | | | **156.8** | 368.6 | 795.2 | 1096.0 | 2714.3 | 16248.8 |
| 135 | | [At5g65420](http://atted.jp/cgi-bin/coex_Ath.cgi?gene=At5g65420&sort=all) | CYCD4;1 | | | **156.8** | 616.3 | 663.8 | 4636.5 | 1831.3 | 20517.9 |
| 136 | | [At2g25270](http://atted.jp/cgi-bin/coex_Ath.cgi?gene=At2g25270&sort=all) |  | | | **157.0** | 616.9 | 119.9 | 6925.5 | 956.5 | 13479.2 |
| 137 | | [At2g42840](http://atted.jp/cgi-bin/coex_Ath.cgi?gene=At2g42840&sort=all) | PDF1 | | | **157.6** | 1130.0 | 141.2 | 421.4 | 5198.5 | 11660.9 |
| 138 | | [At2g36010](http://atted.jp/cgi-bin/coex_Ath.cgi?gene=At2g36010&sort=all) | E2F3 | | | **158.7** | 304.9 | 411.7 | 4036.8 | 9950.2 | 1642.7 |
| 139 | | [At1g05440](http://atted.jp/cgi-bin/coex_Ath.cgi?gene=At1g05440&sort=all) |  | | | **159.2** | 845.0 | 64.9 | 17012.6 | 4749.5 | 3831.1 |
| 140 | | [At5g47500](http://atted.jp/cgi-bin/coex_Ath.cgi?gene=At5g47500&sort=all) | pectinesterase | | | **160.5** | 424.7 | 570.6 | 309.0 | 5155.0 | 11213.4 |
| 141 | | [At5g44560](http://atted.jp/cgi-bin/coex_Ath.cgi?gene=At5g44560&sort=all) | VPS2.2 | | | **161.0** | 585.1 | 276.1 | 3801.1 | 6676.6 | 17877.3 |
| 142 | | [At5g51590](http://atted.jp/cgi-bin/coex_Ath.cgi?gene=At5g51590&sort=all) | DNA-binding | | | **161.4** | 661.0 | 227.4 | 59.4 | 10421.6 | 1119.6 |
| 143 | | [At4g01730](http://atted.jp/cgi-bin/coex_Ath.cgi?gene=At4g01730&sort=all) | zinc ion binding | | | **161.7** | 826.1 | 120.0 | 197.3 | 794.4 | 4395.7 |
| 144 | | [At2g17630](http://atted.jp/cgi-bin/coex_Ath.cgi?gene=At2g17630&sort=all) | transferase | | | **161.9** | 643.1 | 21.8 | 8745.3 | 2090.1 | 15980.0 |
| 145 | | [At4g27230](http://atted.jp/cgi-bin/coex_Ath.cgi?gene=At4g27230&sort=all) | HTA2 | | | **162.4** | 352.3 | 216.8 | 7212.4 | 78.0 | 19877.7 |
| 146 | | [At2g22610](http://atted.jp/cgi-bin/coex_Ath.cgi?gene=At2g22610&sort=all) | kinesin motor | | | **162.6** | 820.2 | 51.3 | 2247.8 | 2685.9 | 1672.1 |
| 147 | | 247442_s_at |  | | | **163.1** | 259.3 | 435.4 | 8776.3 | 10730.7 | 19430.4 |
| 148 | | [At2g28620](http://atted.jp/cgi-bin/coex_Ath.cgi?gene=At2g28620&sort=all) | kinesin motor | | | **164.3** | 803.6 | 23.1 | 2885.0 | 2907.4 | 18594.4 |
| 149 | | [At1g59540](http://atted.jp/cgi-bin/coex_Ath.cgi?gene=At1g59540&sort=all) | ZCF125 | | | **165.2** | 617.5 | 29.1 | 15273.4 | 3011.2 | 8037.1 |
| 150 | | [At5g62390](http://atted.jp/cgi-bin/coex_Ath.cgi?gene=At5g62390&sort=all) | BAG7 | | | **165.4** | 449.8 | 1584.4 | 11691.8 | 8527.6 | 18618.6 |
| 151 | | [At2g01120](http://atted.jp/cgi-bin/coex_Ath.cgi?gene=At2g01120&sort=all) | ORC4 | | | **168.2** | 153.6 | 551.9 | 16755.7 | 5327.2 | 12560.9 |
| 152 | | [At1g08560](http://atted.jp/cgi-bin/coex_Ath.cgi?gene=At1g08560&sort=all) | SYP111 | | | **168.2** | 840.4 | 69.8 | 4105.1 | 3957.2 | 8390.8 |
| 153 | | [At5g05270](http://atted.jp/cgi-bin/coex_Ath.cgi?gene=At5g05270&sort=all) | isomerase | | | **169.0** | 85.2 | 1710.3 | 9172.9 | 46.7 | 7739.9 |
| 154 | | [At4g05190](http://atted.jp/cgi-bin/coex_Ath.cgi?gene=At4g05190&sort=all) | ATK5 | | | **169.6** | 522.5 | 57.7 | 2531.4 | 4912.8 | 8709.3 |
| 155 | | [At5g03870](http://atted.jp/cgi-bin/coex_Ath.cgi?gene=At5g03870&sort=all) | glutaredoxin | | | **170.2** | 822.8 | 86.2 | 9209.7 | 12896.9 | 15055.1 |
| 156 | | [At1g50240](http://atted.jp/cgi-bin/coex_Ath.cgi?gene=At1g50240&sort=all) | FU | | | **170.7** | 780.0 | 180.1 | 84.1 | 6965.5 | 4302.8 |
| 157 | | [At5g01910](http://atted.jp/cgi-bin/coex_Ath.cgi?gene=At5g01910&sort=all) |  | | | **170.8** | 1023.9 | 46.6 | 4016.3 | 3596.8 | 600.8 |
| 158 | | [At2g27970](http://atted.jp/cgi-bin/coex_Ath.cgi?gene=At2g27970&sort=all) | CKS2 | | | **171.0** | 586.0 | 88.5 | 6431.4 | 73.5 | 13596.1 |
| 159 | | [At3g51280](http://atted.jp/cgi-bin/coex_Ath.cgi?gene=At3g51280&sort=all) | MS5 | | | **173.1** | 573.7 | 98.0 | 8314.8 | 368.1 | 7117.1 |
| 160 | | [At1g03780](http://atted.jp/cgi-bin/coex_Ath.cgi?gene=At1g03780&sort=all) | TPX2 | | | **173.2** | 771.3 | 91.0 | 1219.5 | 10836.2 | 11002.3 |
| 161 | | [At3g60900](http://atted.jp/cgi-bin/coex_Ath.cgi?gene=At3g60900&sort=all) | FLA10 | | | **173.2** | 808.2 | 29.8 | 5409.3 | 5893.5 | 3875.2 |
| 162 | | [At4g21820](http://atted.jp/cgi-bin/coex_Ath.cgi?gene=At4g21820&sort=all) | binding | | | **173.9** | 1193.5 | 88.3 | 755.4 | 6524.5 | 16896.2 |
| 163 | | [At1g78770](http://atted.jp/cgi-bin/coex_Ath.cgi?gene=At1g78770&sort=all) | APC6 | | | **174.0** | 310.9 | 734.5 | 8466.5 | 8736.0 | 9653.3 |
| 164 | | [At1g23410](http://atted.jp/cgi-bin/coex_Ath.cgi?gene=At1g23410&sort=all) | RPS27aA | | | **174.6** | 86.0 | 841.9 | 16616.7 | 670.2 | 17269.5 |
| 165 | | [At1g61450](http://atted.jp/cgi-bin/coex_Ath.cgi?gene=At1g61450&sort=all) |  | | | **175.0** | 656.0 | 265.8 | 6214.5 | 15038.6 | 15895.9 |
| 166 | | [At5g10390](http://atted.jp/cgi-bin/coex_Ath.cgi?gene=At5g10390&sort=all) | histone H3 | | | **177.6** | 415.5 | 68.3 | 8441.6 | 2427.3 | 12208.1 |
| 167 | | [At1g02730](http://atted.jp/cgi-bin/coex_Ath.cgi?gene=At1g02730&sort=all) | CSLD5 | | | **178.0** | 1006.9 | 47.0 | 1524.3 | 3242.1 | 3614.3 |
| 168 | | [At5g60150](http://atted.jp/cgi-bin/coex_Ath.cgi?gene=At5g60150&sort=all) |  | | | **178.4** | 996.7 | 114.3 | 1503.2 | 6502.4 | 1600.2 |
| 169 | | [At1g80370](http://atted.jp/cgi-bin/coex_Ath.cgi?gene=At1g80370&sort=all) | CYCA2;4 | | | **180.2** | 565.0 | 298.9 | 2474.9 | 10949.1 | 3729.8 |
| 170 | | [At2g14860](http://atted.jp/cgi-bin/coex_Ath.cgi?gene=At2g14860&sort=all) | peroxisomal membrane 22 kDa | | | **182.0** | 201.0 | 1263.1 | 6189.1 | 5139.9 | 4357.0 |
| 171 | | [At3g11520](http://atted.jp/cgi-bin/coex_Ath.cgi?gene=At3g11520&sort=all) | CYCB1;3 | | | **184.4** | 583.0 | 50.4 | 657.2 | 73.8 | 6179.4 |
| 172 | | 249659_s_at |  | | | **184.7** | 1002.6 | 34.3 | 1397.9 | 799.6 | 1181.3 |
| 173 | | [At3g46940](http://atted.jp/cgi-bin/coex_Ath.cgi?gene=At3g46940&sort=all) | DUT1 | | | **186.2** | 643.5 | 30.2 | 6728.7 | 3162.4 | 5196.3 |
| 174 | | [At1g63100](http://atted.jp/cgi-bin/coex_Ath.cgi?gene=At1g63100&sort=all) | transcription | | | **189.0** | 673.7 | 74.2 | 3048.4 | 8034.4 | 7695.6 |
| 175 | | [At5g05010](http://atted.jp/cgi-bin/coex_Ath.cgi?gene=At5g05010&sort=all) | clathrin adaptor complexes | | | **189.7** | 496.7 | 496.8 | 11934.8 | 6259.9 | 15229.0 |
| 176 | | [At4g39630](http://atted.jp/cgi-bin/coex_Ath.cgi?gene=At4g39630&sort=all) |  | | | **190.1** | 764.4 | 106.4 | 719.8 | 5976.4 | 17136.9 |
| 177 | | [At1g80280](http://atted.jp/cgi-bin/coex_Ath.cgi?gene=At1g80280&sort=all) | hydrolase | | | **191.0** | 755.7 | 20.5 | 11060.8 | 6835.9 | 18171.2 |
| 178 | | [At4g29360](http://atted.jp/cgi-bin/coex_Ath.cgi?gene=At4g29360&sort=all) | hydrolase | | | **191.4** | 384.4 | 2146.4 | 243.3 | 2515.2 | 15361.2 |
| 179 | | [At5g55730](http://atted.jp/cgi-bin/coex_Ath.cgi?gene=At5g55730&sort=all) | FLA1 | | | **191.8** | 488.2 | 104.0 | 2288.1 | 2050.3 | 11478.9 |
| 180 | | [At1g04520](http://atted.jp/cgi-bin/coex_Ath.cgi?gene=At1g04520&sort=all) | PDLP2 | | | **192.3** | 719.0 | 334.2 | 687.5 | 3332.9 | 6864.2 |
| 181 | | [At3g17680](http://atted.jp/cgi-bin/coex_Ath.cgi?gene=At3g17680&sort=all) |  | | | **192.9** | 816.5 | 310.2 | 731.0 | 12147.6 | 9767.6 |
| 182 | | [At4g25240](http://atted.jp/cgi-bin/coex_Ath.cgi?gene=At4g25240&sort=all) | SKS1 | | | **193.4** | 1052.0 | 71.2 | 2728.7 | 3591.3 | 6316.6 |
| 183 | | [At3g10310](http://atted.jp/cgi-bin/coex_Ath.cgi?gene=At3g10310&sort=all) | ATP binding | | | **193.7** | 1034.9 | 68.2 | 5679.4 | 6488.6 | 6532.9 |
| 184 | | [At5g54670](http://atted.jp/cgi-bin/coex_Ath.cgi?gene=At5g54670&sort=all) | ATK3 | | | **196.2** | 1088.7 | 241.6 | 1475.4 | 75.1 | 11100.1 |
| 185 | | [At5g57590](http://atted.jp/cgi-bin/coex_Ath.cgi?gene=At5g57590&sort=all) | BIO1 | | | **196.3** | 367.8 | 809.6 | 54.9 | 7133.7 | 3715.6 |
| 186 | | [At5g22880](http://atted.jp/cgi-bin/coex_Ath.cgi?gene=At5g22880&sort=all) | HTB2 | | | **197.3** | 432.4 | 166.1 | 8618.4 | 2172.2 | 17922.3 |
| 187 | | [At5g08020](http://atted.jp/cgi-bin/coex_Ath.cgi?gene=At5g08020&sort=all) | RPA70B | | | **199.0** | 733.9 | 80.5 | 3968.5 | 3923.4 | 13186.9 |
| 188 | | [At5g63440](http://atted.jp/cgi-bin/coex_Ath.cgi?gene=At5g63440&sort=all) |  | | | **199.3** | 449.9 | 175.5 | 4737.9 | 5990.3 | 13232.1 |
| 189 | | [At5g24330](http://atted.jp/cgi-bin/coex_Ath.cgi?gene=At5g24330&sort=all) | ATXR6 | | | **200.2** | 812.3 | 219.8 | 14.5 | 13929.4 | 4670.7 |
| 190 | | [At5g17620](http://atted.jp/cgi-bin/coex_Ath.cgi?gene=At5g17620&sort=all) |  | | | **200.2** | 427.3 | 295.3 | 4075.0 | 5293.9 | 9764.1 |
| 191 | | [At5g66750](http://atted.jp/cgi-bin/coex_Ath.cgi?gene=At5g66750&sort=all) | CHR1 | | | **201.2** | 923.6 | 184.5 | 7545.3 | 3828.8 | 4431.0 |
| 192 | | [At1g55130](http://atted.jp/cgi-bin/coex_Ath.cgi?gene=At1g55130&sort=all) | endomembrane 70 | | | **201.2** | 423.7 | 454.4 | 5105.1 | 359.5 | 21024.3 |
| 193 | | [At5g06270](http://atted.jp/cgi-bin/coex_Ath.cgi?gene=At5g06270&sort=all) |  | | | **202.9** | 247.3 | 829.2 | 3530.3 | 2211.9 | 3471.4 |
| 194 | | [At3g02120](http://atted.jp/cgi-bin/coex_Ath.cgi?gene=At3g02120&sort=all) | glycoprotein | | | **203.5** | 1119.1 | 72.1 | 4301.1 | 2226.2 | 9856.2 |
| 195 | | [At1g80860](http://atted.jp/cgi-bin/coex_Ath.cgi?gene=At1g80860&sort=all) | PLMT | | | **204.3** | 45.5 | 1037.9 | 9906.8 | 1365.3 | 17591.9 |
| 196 | | [At4g13710](http://atted.jp/cgi-bin/coex_Ath.cgi?gene=At4g13710&sort=all) | lyase | | | **204.8** | 523.2 | 129.5 | 6036.7 | 19090.7 | 13145.4 |
| 197 | | [At3g23670](http://atted.jp/cgi-bin/coex_Ath.cgi?gene=At3g23670&sort=all) | KINESIN-12B | | | **205.5** | 995.5 | 114.0 | 4886.9 | 15388.8 | 493.7 |
| 198 | | [At5g27450](http://atted.jp/cgi-bin/coex_Ath.cgi?gene=At5g27450&sort=all) | MK | | | **205.6** | 345.4 | 1417.0 | 1358.4 | 1074.2 | 9718.3 |
| 199 | | [At5g23530](http://atted.jp/cgi-bin/coex_Ath.cgi?gene=At5g23530&sort=all) | CXE18 | | | **206.1** | 632.1 | 5506.9 | 13555.0 | 1564.4 | 15140.4 |
| 200 | | [At2g05990](http://atted.jp/cgi-bin/coex_Ath.cgi?gene=At2g05990&sort=all) | MOD1 | | | **206.4** | 636.3 | 34.4 | 2009.2 | 5045.3 | 13003.4 |
| 201 | | [At1g76540](http://atted.jp/cgi-bin/coex_Ath.cgi?gene=At1g76540&sort=all) | CDKB2;1 | | | **210.2** | 731.6 | 161.5 | 2660.5 | 2713.6 | 9405.0 |
| 202 | | [At3g51720](http://atted.jp/cgi-bin/coex_Ath.cgi?gene=At3g51720&sort=all) |  | | | **210.4** | 945.5 | 125.7 | 3698.6 | 568.5 | 17684.0 |
| 203 | | [At5g10400](http://atted.jp/cgi-bin/coex_Ath.cgi?gene=At5g10400&sort=all) | histone H3 | | | **212.0** | 181.2 | 226.5 | 10784.2 | 5771.9 | 15262.8 |
| 204 | | [At3g01410](http://atted.jp/cgi-bin/coex_Ath.cgi?gene=At3g01410&sort=all) | RNase H | | | **212.1** | 917.9 | 84.0 | 1520.7 | 11758.0 | 15514.1 |
| 205 | | [At5g61000](http://atted.jp/cgi-bin/coex_Ath.cgi?gene=At5g61000&sort=all) | RPA70D | | | **212.1** | 675.5 | 44.5 | 4863.2 | 4145.6 | 18102.3 |
| 206 | | [At5g14150](http://atted.jp/cgi-bin/coex_Ath.cgi?gene=At5g14150&sort=all) |  | | | **213.0** | 935.7 | 236.7 | 1422.4 | 477.7 | 10302.0 |
| 207 | | [At1g67750](http://atted.jp/cgi-bin/coex_Ath.cgi?gene=At1g67750&sort=all) | lyase | | | **214.0** | 885.8 | 211.0 | 2655.8 | 631.0 | 15176.1 |
| 208 | | [At4g24670](http://atted.jp/cgi-bin/coex_Ath.cgi?gene=At4g24670&sort=all) | TAR2 | | | **214.2** | 386.6 | 1485.6 | 1759.2 | 305.8 | 3993.2 |
| 209 | | [At3g20670](http://atted.jp/cgi-bin/coex_Ath.cgi?gene=At3g20670&sort=all) | HTA13 | | | **214.9** | 161.8 | 451.0 | 10001.8 | 9360.0 | 8393.2 |
| 210 | | [At1g24360](http://atted.jp/cgi-bin/coex_Ath.cgi?gene=At1g24360&sort=all) | reductase | | | **216.1** | 543.4 | 45.9 | 16915.3 | 6942.0 | 21189.8 |
| 211 | | [At5g59870](http://atted.jp/cgi-bin/coex_Ath.cgi?gene=At5g59870&sort=all) | HTA6 | | | **216.7** | 343.8 | 138.0 | 5831.1 | 1158.9 | 8680.5 |
| 212 | | [At2g29570](http://atted.jp/cgi-bin/coex_Ath.cgi?gene=At2g29570&sort=all) | PCNA2 | | | **217.1** | 469.7 | 115.1 | 13716.3 | 3722.1 | 17097.5 |
| 213 | | [At1g06420](http://atted.jp/cgi-bin/coex_Ath.cgi?gene=At1g06420&sort=all) |  | | | **217.3** | 649.7 | 397.5 | 14278.8 | 6581.9 | 17677.2 |
| 214 | | [At1g71830](http://atted.jp/cgi-bin/coex_Ath.cgi?gene=At1g71830&sort=all) | SERK1 | | | **219.8** | 800.3 | 246.6 | 4718.9 | 1570.8 | 16429.3 |
| 215 | | [At2g36200](http://atted.jp/cgi-bin/coex_Ath.cgi?gene=At2g36200&sort=all) | kinesin motor | | | **220.6** | 944.9 | 88.7 | 4818.1 | 8894.7 | 3922.4 |
| 216 | | [At2g22900](http://atted.jp/cgi-bin/coex_Ath.cgi?gene=At2g22900&sort=all) | GMA12/MNN10 | | | **221.2** | 496.7 | 2426.5 | 9240.7 | 51.4 | 14678.4 |
| 217 | | [At4g03100](http://atted.jp/cgi-bin/coex_Ath.cgi?gene=At4g03100&sort=all) | rac GTPase activating | | | **224.1** | 1349.2 | 39.1 | 1818.0 | 3410.1 | 6456.0 |
| 218 | | [At2g36620](http://atted.jp/cgi-bin/coex_Ath.cgi?gene=At2g36620&sort=all) | RPL24A | | | **225.0** | 154.0 | 205.8 | 9719.2 | 1581.3 | 16287.4 |
| 219 | | [At5g13960](http://atted.jp/cgi-bin/coex_Ath.cgi?gene=At5g13960&sort=all) | SUVH4 | | | **225.9** | 799.4 | 573.4 | 11415.4 | 6308.4 | 5253.7 |
| 220 | | [At5g16250](http://atted.jp/cgi-bin/coex_Ath.cgi?gene=At5g16250&sort=all) |  | | | **226.0** | 914.2 | 78.8 | 1558.2 | 1558.9 | 3025.5 |
| 221 | | [At2g42120](http://atted.jp/cgi-bin/coex_Ath.cgi?gene=At2g42120&sort=all) | POLD2 | | | **226.6** | 347.8 | 948.2 | 8038.3 | 3079.9 | 769.2 |
| 222 | | [At4g29680](http://atted.jp/cgi-bin/coex_Ath.cgi?gene=At4g29680&sort=all) | pyrophosphatase | | | **227.1** | 333.9 | 702.8 | 14133.8 | 340.0 | 17619.8 |
| 223 | | [At5g06940](http://atted.jp/cgi-bin/coex_Ath.cgi?gene=At5g06940&sort=all) | LRR | | | **227.7** | 973.7 | 141.9 | 39.5 | 13131.2 | 8141.0 |
| 224 | | [At5g15510](http://atted.jp/cgi-bin/coex_Ath.cgi?gene=At5g15510&sort=all) |  | | | **227.9** | 459.8 | 147.3 | 13417.2 | 6312.0 | 10429.8 |
| 225 | | [At3g63250](http://atted.jp/cgi-bin/coex_Ath.cgi?gene=At3g63250&sort=all) | HMT2 | | | **228.0** | 389.5 | 1187.5 | 6333.9 | 1708.2 | 7579.0 |
| 226 | | [At5g63920](http://atted.jp/cgi-bin/coex_Ath.cgi?gene=At5g63920&sort=all) | TOP3A | | | **228.7** | 982.3 | 138.5 | 53.7 | 4610.7 | 7131.4 |
| 227 | | [At1g15660](http://atted.jp/cgi-bin/coex_Ath.cgi?gene=At1g15660&sort=all) | CENP-C | | | **228.8** | 330.7 | 157.8 | 15355.6 | 9073.9 | 19501.4 |
| 228 | | [At1g12000](http://atted.jp/cgi-bin/coex_Ath.cgi?gene=At1g12000&sort=all) | kinase | | | **229.7** | 416.8 | 228.4 | 17410.6 | 3125.6 | 8751.6 |
| 229 | | [At5g23940](http://atted.jp/cgi-bin/coex_Ath.cgi?gene=At5g23940&sort=all) | EMB3009 | | | **230.1** | 431.0 | 2029.6 | 10045.6 | 7968.3 | 7616.9 |
| 230 | | [At4g33130](http://atted.jp/cgi-bin/coex_Ath.cgi?gene=At4g33130&sort=all) |  | | | **231.0** | 504.7 | 1238.2 | 11187.0 | 1394.4 | 20907.1 |
| 231 | | [At3g51740](http://atted.jp/cgi-bin/coex_Ath.cgi?gene=At3g51740&sort=all) | IMK2 | | | **231.7** | 986.3 | 109.1 | 6492.6 | 6334.2 | 15010.9 |
| 232 | | [At1g50490](http://atted.jp/cgi-bin/coex_Ath.cgi?gene=At1g50490&sort=all) | UBC20 | | | **232.4** | 828.9 | 51.8 | 6859.8 | 2184.8 | 2624.0 |
| 233 | | [At1g16920](http://atted.jp/cgi-bin/coex_Ath.cgi?gene=At1g16920&sort=all) | RABA1B | | | **232.4** | 676.7 | 247.4 | 4150.9 | 2174.2 | 12358.2 |
| 234 | | [At5g62410](http://atted.jp/cgi-bin/coex_Ath.cgi?gene=At5g62410&sort=all) | SMC2 | | | **232.7** | 530.5 | 438.0 | 1915.7 | 4218.4 | 12835.0 |
| 235 | | [At1g60810](http://atted.jp/cgi-bin/coex_Ath.cgi?gene=At1g60810&sort=all) | ACLA-2 | | | **233.3** | 827.8 | 117.6 | 8274.8 | 430.4 | 5371.6 |
| 236 | | [At4g21590](http://atted.jp/cgi-bin/coex_Ath.cgi?gene=At4g21590&sort=all) | ENDO3 | | | **233.6** | 529.7 | 463.9 | 4345.7 | 1836.5 | 10409.3 |
| 237 | | [At4g26660](http://atted.jp/cgi-bin/coex_Ath.cgi?gene=At4g26660&sort=all) |  | | | **233.7** | 1238.2 | 65.4 | 6617.0 | 4000.6 | 17509.8 |
| 238 | | [264552_at](http://atted.jp/cgi-bin/coex_Ath.cgi?gene=264552_at&sort=all) |  | | | **235.4** | 1027.8 | 65.0 | 149.8 | 8590.3 | 5676.3 |
| 239 | | [At1g64650](http://atted.jp/cgi-bin/coex_Ath.cgi?gene=At1g64650&sort=all) |  | | | **235.5** | 853.5 | 90.6 | 6993.9 | 2136.2 | 16477.6 |
| 240 | | [At3g62300](http://atted.jp/cgi-bin/coex_Ath.cgi?gene=At3g62300&sort=all) | DUF7 | | | **235.6** | 182.4 | 843.9 | 7491.6 | 3497.9 | 17887.3 |
| 241 | | [At3g63300](http://atted.jp/cgi-bin/coex_Ath.cgi?gene=At3g63300&sort=all) | phosphoinositide binding | | | **236.7** | 857.4 | 465.1 | 346.2 | 566.6 | 3281.7 |
| 242 | | [At4g02290](http://atted.jp/cgi-bin/coex_Ath.cgi?gene=At4g02290&sort=all) | GH9B13 | | | **236.8** | 796.4 | 317.2 | 133.4 | 8806.2 | 19595.8 |
| 243 | | [At1g04020](http://atted.jp/cgi-bin/coex_Ath.cgi?gene=At1g04020&sort=all) | BARD1 | | | **237.2** | 629.4 | 86.0 | 11984.2 | 3733.2 | 9224.0 |
| 244 | | [At1g64450](http://atted.jp/cgi-bin/coex_Ath.cgi?gene=At1g64450&sort=all) | proline-rich | | | **237.7** | 869.0 | 489.2 | 7062.8 | 3085.4 | 2151.0 |
| 245 | | [At5g37010](http://atted.jp/cgi-bin/coex_Ath.cgi?gene=At5g37010&sort=all) |  | | | **238.5** | 746.6 | 105.6 | 930.9 | 8042.6 | 18011.5 |
| 246 | | [At3g56110](http://atted.jp/cgi-bin/coex_Ath.cgi?gene=At3g56110&sort=all) | PRA1.B1 | | | **238.6** | 229.9 | 1317.4 | 20161.9 | 4721.6 | 10975.5 |
| 247 | | [At1g28290](http://atted.jp/cgi-bin/coex_Ath.cgi?gene=At1g28290&sort=all) | AGP31 | | | **241.3** | 906.8 | 704.0 | 3334.2 | 1676.7 | 16762.5 |
| 248 | | [At3g17840](http://atted.jp/cgi-bin/coex_Ath.cgi?gene=At3g17840&sort=all) | RLK902 | | | **242.1** | 664.1 | 115.2 | 5483.3 | 316.8 | 4746.3 |
| 249 | | 259563_s_at |  | | | **242.4** | 320.2 | 263.5 | 1932.0 | 872.2 | 16298.1 |
| 250 | | [At2g18400](http://atted.jp/cgi-bin/coex_Ath.cgi?gene=At2g18400&sort=all) | ribosome | | | **242.6** | 120.2 | 261.7 | 9930.5 | 2176.4 | 21140.3 |
| 251 | | [At1g72670](http://atted.jp/cgi-bin/coex_Ath.cgi?gene=At1g72670&sort=all) | iqd8 | | | **243.8** | 1226.2 | 162.4 | 10812.2 | 1900.1 | 1416.3 |
| 252 | | [At3g15550](http://atted.jp/cgi-bin/coex_Ath.cgi?gene=At3g15550&sort=all) |  | | | **244.1** | 1096.5 | 123.5 | 2575.1 | 4985.9 | 13515.0 |
| 253 | | [At4g29700](http://atted.jp/cgi-bin/coex_Ath.cgi?gene=At4g29700&sort=all) | pyrophosphatase | | | **245.2** | 644.1 | 1931.3 | 14820.9 | 5338.9 | 17255.6 |
| 254 | | [At3g49250](http://atted.jp/cgi-bin/coex_Ath.cgi?gene=At3g49250&sort=all) | DMS3 | | | **245.3** | 1114.1 | 145.2 | 6256.8 | 80.3 | 11198.2 |
| 255 | | [At5g48310](http://atted.jp/cgi-bin/coex_Ath.cgi?gene=At5g48310&sort=all) |  | | | **249.5** | 1066.3 | 97.8 | 4082.5 | 7023.9 | 9445.1 |
| 256 | | [At3g22780](http://atted.jp/cgi-bin/coex_Ath.cgi?gene=At3g22780&sort=all) | TSO1 | | | **250.3** | 299.0 | 462.2 | 5339.6 | 5957.1 | 16060.3 |
| 257 | | [At2g24490](http://atted.jp/cgi-bin/coex_Ath.cgi?gene=At2g24490&sort=all) | RPA2 | | | **251.7** | 205.8 | 506.7 | 6938.6 | 6540.6 | 11244.3 |
| 258 | | [At3g44590](http://atted.jp/cgi-bin/coex_Ath.cgi?gene=At3g44590&sort=all) | RPP2D | | | **252.2** | 111.9 | 158.4 | 11966.3 | 1426.2 | 12695.6 |
| 259 | | [At1g14180](http://atted.jp/cgi-bin/coex_Ath.cgi?gene=At1g14180&sort=all) | protein binding | | | **252.3** | 564.5 | 307.4 | 43.1 | 14253.7 | 6009.9 |
| 260 | | [At5g59740](http://atted.jp/cgi-bin/coex_Ath.cgi?gene=At5g59740&sort=all) | transporter | | | **253.3** | 747.0 | 1469.3 | 6640.6 | 7031.9 | 16327.7 |
| 261 | | [At5g27670](http://atted.jp/cgi-bin/coex_Ath.cgi?gene=At5g27670&sort=all) | HTA7 | | | **253.8** | 225.6 | 1006.1 | 12308.2 | 3190.7 | 19873.2 |
| 262 | | [247311_at](http://atted.jp/cgi-bin/coex_Ath.cgi?gene=247311_at&sort=all) |  | | | **254.3** | 284.1 | 547.9 | 2887.3 | 5975.1 | 20791.0 |
| 263 | | [At2g16270](http://atted.jp/cgi-bin/coex_Ath.cgi?gene=At2g16270&sort=all) |  | | | **255.5** | 851.8 | 62.6 | 1704.4 | 7518.6 | 7534.8 |
| 264 | | [At2g20710](http://atted.jp/cgi-bin/coex_Ath.cgi?gene=At2g20710&sort=all) | PPR | | | **256.7** | 21.8 | 1070.8 | 14648.2 | 14020.7 | 3967.2 |
| 265 | | [At5g48600](http://atted.jp/cgi-bin/coex_Ath.cgi?gene=At5g48600&sort=all) | SMC3 | | | **257.3** | 785.4 | 237.4 | 8769.5 | 7683.3 | 1602.1 |
| 266 | | [At3g44050](http://atted.jp/cgi-bin/coex_Ath.cgi?gene=At3g44050&sort=all) | kinesin motor | | | **258.3** | 822.0 | 105.7 | 13671.5 | 2130.0 | 13618.4 |
| 267 | | [At1g74055](http://atted.jp/cgi-bin/coex_Ath.cgi?gene=At1g74055&sort=all) |  | | | **259.4** | 511.0 | 1854.2 | 2228.7 | 21729.0 | 17357.0 |
| 268 | | [At1g50110](http://atted.jp/cgi-bin/coex_Ath.cgi?gene=At1g50110&sort=all) | BCAT6 | | | **261.9** | 362.4 | 348.9 | 1940.5 | 6163.6 | 18746.4 |
| 269 | | [At2g40550](http://atted.jp/cgi-bin/coex_Ath.cgi?gene=At2g40550&sort=all) | ETG1 | | | **262.0** | 468.8 | 263.9 | 7239.9 | 10278.7 | 18925.0 |
| 270 | | [At5g23420](http://atted.jp/cgi-bin/coex_Ath.cgi?gene=At5g23420&sort=all) | HMGB6 | | | **262.6** | 707.2 | 80.6 | 11611.0 | 5521.1 | 22339.4 |
| 271 | | [At5g15520](http://atted.jp/cgi-bin/coex_Ath.cgi?gene=At5g15520&sort=all) | RPS19B | | | **262.9** | 25.7 | 401.0 | 9653.0 | 5072.4 | 10556.2 |
| 272 | | [At4g15790](http://atted.jp/cgi-bin/coex_Ath.cgi?gene=At4g15790&sort=all) |  | | | **263.3** | 652.9 | 887.4 | 7151.5 | 1143.8 | 6356.4 |
| 273 | | [At5g45700](http://atted.jp/cgi-bin/coex_Ath.cgi?gene=At5g45700&sort=all) | NIF | | | **263.6** | 1369.9 | 25.6 | 15703.3 | 2181.2 | 14932.8 |
| 274 | | [At5g51600](http://atted.jp/cgi-bin/coex_Ath.cgi?gene=At5g51600&sort=all) | PLE | | | **264.0** | 1059.5 | 181.2 | 8642.9 | 1191.3 | 5197.9 |
| 275 | | [At3g03960](http://atted.jp/cgi-bin/coex_Ath.cgi?gene=At3g03960&sort=all) | chaperonin | | | **266.2** | 201.8 | 353.5 | 12139.9 | 6137.7 | 16536.3 |
| 276 | | [At2g32765](http://atted.jp/cgi-bin/coex_Ath.cgi?gene=At2g32765&sort=all) | SUMO5 | | | **266.6** | 822.8 | 1383.0 | 9075.8 | 381.1 | 17149.7 |
| 277 | | [At2g22370](http://atted.jp/cgi-bin/coex_Ath.cgi?gene=At2g22370&sort=all) |  | | | **268.8** | 173.8 | 1783.5 | 12123.5 | 9501.5 | 8544.1 |
| 278 | | [At2g17620](http://atted.jp/cgi-bin/coex_Ath.cgi?gene=At2g17620&sort=all) | CYCB2;1 | | | **270.1** | 828.2 | 149.9 | 682.9 | 12247.0 | 16543.7 |
| 279 | | [At5g67100](http://atted.jp/cgi-bin/coex_Ath.cgi?gene=At5g67100&sort=all) | ICU2 | | | **271.1** | 873.5 | 174.1 | 4197.3 | 6195.1 | 2041.8 |
| 280 | | [At1g22170](http://atted.jp/cgi-bin/coex_Ath.cgi?gene=At1g22170&sort=all) | mutase | | | **272.2** | 1217.2 | 255.6 | 587.5 | 285.9 | 12715.5 |
| 281 | | [At4g35905](http://atted.jp/cgi-bin/coex_Ath.cgi?gene=At4g35905&sort=all) |  | | | **273.5** | 338.4 | 1427.1 | 6626.5 | 654.1 | 20545.6 |
| 282 | | [At2g37380](http://atted.jp/cgi-bin/coex_Ath.cgi?gene=At2g37380&sort=all) |  | | | **273.6** | 713.5 | 189.9 | 1601.5 | 5677.8 | 6936.6 |
| 283 | | [At5g09980](http://atted.jp/cgi-bin/coex_Ath.cgi?gene=At5g09980&sort=all) | PROPEP4 | | | **274.3** | 126.1 | 7787.5 | 9392.3 | 18088.5 | 18044.1 |
| 284 | | [At1g26640](http://atted.jp/cgi-bin/coex_Ath.cgi?gene=At1g26640&sort=all) | kinase | | | **276.8** | 712.6 | 427.9 | 12381.3 | 618.7 | 17183.1 |
| 285 | | [At1g65060](http://atted.jp/cgi-bin/coex_Ath.cgi?gene=At1g65060&sort=all) | 4CL3 | | | **277.1** | 140.4 | 3552.0 | 6559.9 | 38.1 | 5632.4 |
| 286 | | [At5g55820](http://atted.jp/cgi-bin/coex_Ath.cgi?gene=At5g55820&sort=all) |  | | | **277.2** | 896.4 | 365.4 | 7250.7 | 7066.8 | 7461.2 |
| 287 | | [At4g30130](http://atted.jp/cgi-bin/coex_Ath.cgi?gene=At4g30130&sort=all) |  | | | **277.8** | 649.6 | 376.8 | 4186.3 | 15757.9 | 11295.3 |
| 288 | | [At1g16520](http://atted.jp/cgi-bin/coex_Ath.cgi?gene=At1g16520&sort=all) |  | | | **278.4** | 548.5 | 805.1 | 10443.1 | 7663.4 | 6739.7 |
| 289 | | [At1g47200](http://atted.jp/cgi-bin/coex_Ath.cgi?gene=At1g47200&sort=all) | WPP2 | | | **279.4** | 579.0 | 390.3 | 3107.5 | 3943.2 | 12272.2 |
| 290 | | [At1g20930](http://atted.jp/cgi-bin/coex_Ath.cgi?gene=At1g20930&sort=all) | CDKB2;2 | | | **279.6** | 872.6 | 253.7 | 2911.1 | 3853.1 | 12161.2 |
| 291 | | [At5g66230](http://atted.jp/cgi-bin/coex_Ath.cgi?gene=At5g66230&sort=all) |  | | | **279.8** | 1054.1 | 117.4 | 211.0 | 527.0 | 10971.8 |
| 292 | | [At2g37300](http://atted.jp/cgi-bin/coex_Ath.cgi?gene=At2g37300&sort=all) |  | | | **283.0** | 730.9 | 1169.2 | 3788.2 | 215.5 | 11769.6 |
| 293 | | [At5g65360](http://atted.jp/cgi-bin/coex_Ath.cgi?gene=At5g65360&sort=all) | histone H3 | | | **283.5** | 433.7 | 187.7 | 9089.9 | 3902.6 | 11784.7 |
| 294 | | [At3g07320](http://atted.jp/cgi-bin/coex_Ath.cgi?gene=At3g07320&sort=all) | hydrolase | | | **284.6** | 716.6 | 1273.0 | 5815.0 | 7188.2 | 21958.5 |
| 295 | | [At1g75640](http://atted.jp/cgi-bin/coex_Ath.cgi?gene=At1g75640&sort=all) | kinase | | | **286.0** | 951.6 | 223.6 | 6188.4 | 9439.8 | 12433.9 |
| 296 | | [At3g06740](http://atted.jp/cgi-bin/coex_Ath.cgi?gene=At3g06740&sort=all) | GATA15 | | | **286.3** | 1158.2 | 461.7 | 5358.6 | 3783.2 | 19460.9 |
| 297 | | [At5g27330](http://atted.jp/cgi-bin/coex_Ath.cgi?gene=At5g27330&sort=all) |  | | | **287.6** | 293.9 | 280.0 | 10063.4 | 7146.6 | 14922.0 |
| 298 | | [At3g53730](http://atted.jp/cgi-bin/coex_Ath.cgi?gene=At3g53730&sort=all) | histone H4 | | | **287.7** | 725.6 | 406.8 | 11461.5 | 4277.5 | 16362.4 |
| 299 | | [254282_at](http://atted.jp/cgi-bin/coex_Ath.cgi?gene=254282_at&sort=all) |  | | | **287.7** | 570.7 | 489.0 | 2421.6 | 10945.4 | 2344.5 |
| 300 | | [At5g08640](http://atted.jp/cgi-bin/coex_Ath.cgi?gene=At5g08640&sort=all) | FLS | | | **287.9** | 285.3 | 3958.8 | 8397.6 | 4.5 | 7220.2 |
|  | | | | | | | | | | | |
| **300 coexpressed gene with At1g75030** | | | | | | | | | | | |
|  | **locus** | | | **Short description** | | **MR**  **(all)** | **MR**  **(tissue)** | **MR**  **(abiotic)** | **MR**  **(biotic)** | **MR**  **(hormone)** | **MR**  **(light)** |
| 1 | [At1g01280](http://atted.jp/cgi-bin/coex_Ath.cgi?gene=At1g01280&sort=all) | | | CYP703A2 | | **9.8** | 22.1 | 137.5 | 9660.0 | 9336.8 | 7949.5 |
| 2 | [At3g42960](http://atted.jp/cgi-bin/coex_Ath.cgi?gene=At3g42960&sort=all) | | | ATA1 | | **13.3** | 16.2 | 223.5 | 21512.7 | 5144.9 | 12778.4 |
| 3 | [At1g61070](http://atted.jp/cgi-bin/coex_Ath.cgi?gene=At1g61070&sort=all) | | | LCR66 | | **17.5** | 29.7 | 218.2 | 6249.6 | 10593.0 | 8190.4 |
| 4 | [At4g35420](http://atted.jp/cgi-bin/coex_Ath.cgi?gene=At4g35420&sort=all) | | | DRL1 | | **18.0** | 10.3 | 724.3 | 8735.6 | 54.8 | 10968.3 |
| 5 | [At3g11980](http://atted.jp/cgi-bin/coex_Ath.cgi?gene=At3g11980&sort=all) | | | MS2 | | **20.1** | 19.7 | 246.0 | 19584.9 | 13828.4 | 13870.5 |
| 6 | [At3g23770](http://atted.jp/cgi-bin/coex_Ath.cgi?gene=At3g23770&sort=all) | | | hydrolase | | **22.1** | 43.8 | 126.2 | 12964.0 | 7384.6 | 8501.7 |
| 7 | [At1g02050](http://atted.jp/cgi-bin/coex_Ath.cgi?gene=At1g02050&sort=all) | | | synthase | | **22.3** | 21.1 | 221.4 | 1601.3 | 1275.5 | 15162.9 |
| 8 | [At5g07230](http://atted.jp/cgi-bin/coex_Ath.cgi?gene=At5g07230&sort=all) | | | LTP | | **24.6** | 35.5 | 214.1 | 16183.5 | 9428.2 | 7905.7 |
| 9 | [At5g13380](http://atted.jp/cgi-bin/coex_Ath.cgi?gene=At5g13380&sort=all) | | | GH3 | | **27.8** | 8.9 | 3343.9 | 11239.0 | 10835.0 | 22247.8 |
| 10 | [At4g14080](http://atted.jp/cgi-bin/coex_Ath.cgi?gene=At4g14080&sort=all) | | | MEE48 | | **30.9** | 36.7 | 279.9 | 20094.3 | 16553.4 | 17666.9 |
| 11 | [At4g34850](http://atted.jp/cgi-bin/coex_Ath.cgi?gene=At4g34850&sort=all) | | | synthase | | **31.9** | 31.9 | 258.2 | 21210.5 | 10794.7 | 13226.8 |
| 12 | [At1g02813](http://atted.jp/cgi-bin/coex_Ath.cgi?gene=At1g02813&sort=all) | | |  | | **33.8** | 28.7 | 535.3 | 16886.0 | 15776.8 | 9948.5 |
| 13 | [At1g62940](http://atted.jp/cgi-bin/coex_Ath.cgi?gene=At1g62940&sort=all) | | | ACOS5 | | **36.7** | 26.3 | 413.0 | 5352.6 | 7905.1 | 5796.6 |
| 14 | [At1g69500](http://atted.jp/cgi-bin/coex_Ath.cgi?gene=At1g69500&sort=all) | | | CYP704B1 | | **37.5** | 63.6 | 231.9 | 3636.8 | 5562.7 | 16942.2 |
| 15 | [At5g16920](http://atted.jp/cgi-bin/coex_Ath.cgi?gene=At5g16920&sort=all) | | |  | | **39.5** | 44.2 | 855.5 | 11234.0 | 10451.7 | 21328.4 |
| 16 | [At5g55590](http://atted.jp/cgi-bin/coex_Ath.cgi?gene=At5g55590&sort=all) | | | QRT1 | | **39.7** | 90.8 | 235.6 | 15451.2 | 5215.4 | 1789.1 |
| 17 | [At3g07450](http://atted.jp/cgi-bin/coex_Ath.cgi?gene=At3g07450&sort=all) | | | LTP | | **40.4** | 40.9 | 593.2 | 9756.1 | 9829.6 | 16935.7 |
| 18 | [At3g13220](http://atted.jp/cgi-bin/coex_Ath.cgi?gene=At3g13220&sort=all) | | | WBC27 | | **42.0** | 33.8 | 774.1 | 18973.9 | 6366.2 | 10733.2 |
| 19 | [At5g47635](http://atted.jp/cgi-bin/coex_Ath.cgi?gene=At5g47635&sort=all) | | |  | | **44.1** | 144.0 | 39.9 | 16645.5 | 95.1 | 16143.0 |
| 20 | [At3g57620](http://atted.jp/cgi-bin/coex_Ath.cgi?gene=At3g57620&sort=all) | | | oxidase | | **48.3** | 54.8 | 2492.3 | 2107.2 | 3558.8 | 6812.8 |
| 21 | [At3g52130](http://atted.jp/cgi-bin/coex_Ath.cgi?gene=At3g52130&sort=all) | | | LTP | | **50.0** | 66.0 | 824.1 | 12999.4 | 8377.4 | 8011.0 |
| 22 | [At3g52160](http://atted.jp/cgi-bin/coex_Ath.cgi?gene=At3g52160&sort=all) | | | KCS15 | | **52.5** | 51.4 | 7961.8 | 4112.9 | 11986.6 | 12723.0 |
| 23 | [At4g20420](http://atted.jp/cgi-bin/coex_Ath.cgi?gene=At4g20420&sort=all) | | | tapetum-specific | | **54.8** | 46.9 | 699.1 | 13652.2 | 11513.1 | 21995.2 |
| 24 | [At1g20150](http://atted.jp/cgi-bin/coex_Ath.cgi?gene=At1g20150&sort=all) | | | subtilase | | **57.1** | 35.7 | 11931.1 | 12581.1 | 9202.8 | 21295.8 |
| 25 | [At4g29980](http://atted.jp/cgi-bin/coex_Ath.cgi?gene=At4g29980&sort=all) | | |  | | **57.2** | 65.7 | 644.5 | 8146.5 | 9707.5 | 10631.6 |
| 26 | [At5g62080](http://atted.jp/cgi-bin/coex_Ath.cgi?gene=At5g62080&sort=all) | | | LTP | | **59.3** | 67.7 | 442.8 | 7146.2 | 9028.6 | 21763.5 |
| 27 | [At1g03390](http://atted.jp/cgi-bin/coex_Ath.cgi?gene=At1g03390&sort=all) | | | transferase | | **59.6** | 52.2 | 4446.4 | 2028.7 | 5769.7 | 5827.0 |
| 28 | [At5g52160](http://atted.jp/cgi-bin/coex_Ath.cgi?gene=At5g52160&sort=all) | | | LTP | | **60.8** | 59.1 | 4848.2 | 14058.1 | 7603.5 | 4393.6 |
| 29 | [At1g67990](http://atted.jp/cgi-bin/coex_Ath.cgi?gene=At1g67990&sort=all) | | | TSM1 | | **61.0** | 79.1 | 1317.8 | 961.7 | 6426.0 | 19218.1 |
| 30 | [At1g30020](http://atted.jp/cgi-bin/coex_Ath.cgi?gene=At1g30020&sort=all) | | |  | | **62.5** | 54.8 | 11215.8 | 12598.9 | 6520.6 | 16089.2 |
| 31 | [At4g20050](http://atted.jp/cgi-bin/coex_Ath.cgi?gene=At4g20050&sort=all) | | | QRT3 | | **66.1** | 132.1 | 41.0 | 1852.2 | 170.4 | 10800.6 |
| 32 | [At2g16910](http://atted.jp/cgi-bin/coex_Ath.cgi?gene=At2g16910&sort=all) | | | AMS | | **68.1** | 88.5 | 688.6 | 11452.4 | 5023.7 | 1679.4 |
| 33 | [At5g61110](http://atted.jp/cgi-bin/coex_Ath.cgi?gene=At5g61110&sort=all) | | | protein binding | | **71.8** | 53.2 | 1353.2 | 9618.7 | 11132.1 | 4573.9 |
| 34 | [At4g28395](http://atted.jp/cgi-bin/coex_Ath.cgi?gene=At4g28395&sort=all) | | | ATA7 | | **71.8** | 55.9 | 7176.8 | 18467.7 | 11703.0 | 13111.2 |
| 35 | [At5g48210](http://atted.jp/cgi-bin/coex_Ath.cgi?gene=At5g48210&sort=all) | | |  | | **71.8** | 73.0 | 8649.0 | 14567.8 | 9710.5 | 18689.3 |
| 36 | [At5g24820](http://atted.jp/cgi-bin/coex_Ath.cgi?gene=At5g24820&sort=all) | | | protease | | **75.9** | 95.7 | 979.2 | 16867.5 | 11802.5 | 7403.9 |
| 37 | 257400_s_at | | |  | | **79.9** | 104.2 | 1091.9 | 9344.4 | 4501.8 | 6787.7 |
| 38 | 247639_s_at | | |  | | **80.0** | 78.6 | 13315.6 | 19602.7 | 8713.8 | 4012.7 |
| 39 | [At1g71160](http://atted.jp/cgi-bin/coex_Ath.cgi?gene=At1g71160&sort=all) | | | KCS7 | | **82.0** | 79.7 | 11314.7 | 9476.2 | 6776.0 | 6950.6 |
| 40 | [At2g18420](http://atted.jp/cgi-bin/coex_Ath.cgi?gene=At2g18420&sort=all) | | |  | | **82.2** | 93.0 | 2230.9 | 8832.7 | 8233.4 | 4125.9 |
| 41 | [At2g42940](http://atted.jp/cgi-bin/coex_Ath.cgi?gene=At2g42940&sort=all) | | | DNA-binding | | **83.7** | 62.3 | 2009.4 | 18057.0 | 13274.5 | 14708.5 |
| 42 | [At2g31210](http://atted.jp/cgi-bin/coex_Ath.cgi?gene=At2g31210&sort=all) | | | bHLH | | **84.3** | 65.5 | 4727.0 | 8091.1 | 3377.5 | 16711.6 |
| 43 | [At1g23330](http://atted.jp/cgi-bin/coex_Ath.cgi?gene=At1g23330&sort=all) | | |  | | **87.0** | 647.5 | 397.2 | 7492.2 | 60.8 | 3264.7 |
| 44 | [At2g19070](http://atted.jp/cgi-bin/coex_Ath.cgi?gene=At2g19070&sort=all) | | | SHT | | **89.2** | 86.0 | 11625.0 | 21545.1 | 6687.2 | 3869.1 |
| 45 | [At3g06100](http://atted.jp/cgi-bin/coex_Ath.cgi?gene=At3g06100&sort=all) | | | NIP7;1 | | **91.0** | 74.4 | 1195.6 | 3706.7 | 6078.6 | 6161.7 |
| 46 | 254338_s_at | | |  | | **91.1** | 28.5 | 10360.7 | 11494.3 | 11629.9 | 6.9 |
| 47 | [At5g60090](http://atted.jp/cgi-bin/coex_Ath.cgi?gene=At5g60090&sort=all) | | | kinase | | **91.2** | 57.3 | 8594.2 | 1598.1 | 10757.5 | 1366.8 |
| 48 | [At1g06170](http://atted.jp/cgi-bin/coex_Ath.cgi?gene=At1g06170&sort=all) | | | bHLH | | **91.4** | 96.2 | 5647.6 | 2569.5 | 9062.5 | 5843.1 |
| 49 | [At4g12920](http://atted.jp/cgi-bin/coex_Ath.cgi?gene=At4g12920&sort=all) | | | protease | | **91.5** | 83.0 | 5880.1 | 11189.1 | 4644.9 | 17998.4 |
| 50 | [At5g62320](http://atted.jp/cgi-bin/coex_Ath.cgi?gene=At5g62320&sort=all) | | | MYB99 | | **92.8** | 65.7 | 9195.8 | 15047.7 | 7248.5 | 7444.1 |
| 51 | [At5g61260](http://atted.jp/cgi-bin/coex_Ath.cgi?gene=At5g61260&sort=all) | | | chromosome scaffold | | **98.5** | 275.9 | 385.6 | 7521.1 | 9377.5 | 12091.4 |
| 52 | [At5g16960](http://atted.jp/cgi-bin/coex_Ath.cgi?gene=At5g16960&sort=all) | | | oxidoreductase | | **99.1** | 55.5 | 15347.6 | 7309.6 | 11411.1 | 11021.5 |
| 53 | [At5g28520](http://atted.jp/cgi-bin/coex_Ath.cgi?gene=At5g28520&sort=all) | | |  | | **100.9** | 15280.5 | 16.7 | 5618.7 | 57.5 | 11074.4 |
| 54 | [At1g75790](http://atted.jp/cgi-bin/coex_Ath.cgi?gene=At1g75790&sort=all) | | | sks18 | | **103.5** | 120.5 | 1573.0 | 14224.9 | 8387.9 | 17479.2 |
| 55 | [At4g30040](http://atted.jp/cgi-bin/coex_Ath.cgi?gene=At4g30040&sort=all) | | | protease | | **107.7** | 82.0 | 5150.3 | 9583.5 | 11585.2 | 19325.5 |
| 56 | [At1g33430](http://atted.jp/cgi-bin/coex_Ath.cgi?gene=At1g33430&sort=all) | | | transferase | | **108.1** | 136.5 | 596.7 | 2345.9 | 13519.9 | 21956.2 |
| 57 | [At4g14815](http://atted.jp/cgi-bin/coex_Ath.cgi?gene=At4g14815&sort=all) | | | LTP | | **108.3** | 108.0 | 7451.4 | 4818.9 | 8794.9 | 14146.0 |
| 58 | [At5g49070](http://atted.jp/cgi-bin/coex_Ath.cgi?gene=At5g49070&sort=all) | | | KCS21 | | **108.8** | 78.8 | 19542.0 | 416.4 | 12656.2 | 19007.4 |
| 59 | [At3g20450](http://atted.jp/cgi-bin/coex_Ath.cgi?gene=At3g20450&sort=all) | | |  | | **109.2** | 61.8 | 11347.9 | 8069.7 | 8930.3 | 13458.0 |
| 60 | [At5g40940](http://atted.jp/cgi-bin/coex_Ath.cgi?gene=At5g40940&sort=all) | | | FLA20 | | **116.0** | 86.8 | 10666.1 | 12388.0 | 6420.9 | 19246.4 |
| 61 | [At1g19230](http://atted.jp/cgi-bin/coex_Ath.cgi?gene=At1g19230&sort=all) | | | RbohE | | **118.2** | 285.9 | 560.7 | 5218.3 | 477.9 | 111.0 |
| 62 | 264273_s_at | | |  | | **128.0** | 86.1 | 3997.4 | 16515.8 | 18850.3 | 14288.1 |
| 63 | [At1g74540](http://atted.jp/cgi-bin/coex_Ath.cgi?gene=At1g74540&sort=all) | | | CYP98A8 | | **128.7** | 119.7 | 12354.2 | 4542.4 | 11113.9 | 7453.7 |
| 64 | [At1g26710](http://atted.jp/cgi-bin/coex_Ath.cgi?gene=At1g26710&sort=all) | | |  | | **136.5** | 135.3 | 9980.9 | 13772.6 | 4592.9 | 19551.7 |
| 65 | [At4g35690](http://atted.jp/cgi-bin/coex_Ath.cgi?gene=At4g35690&sort=all) | | |  | | **142.9** | 9788.5 | 72.8 | 8132.3 | 21.8 | 3231.9 |
| 66 | [At1g13140](http://atted.jp/cgi-bin/coex_Ath.cgi?gene=At1g13140&sort=all) | | | CYP86C3 | | **143.0** | 137.7 | 6846.1 | 17398.8 | 13796.9 | 16394.8 |
| 67 | [At4g29250](http://atted.jp/cgi-bin/coex_Ath.cgi?gene=At4g29250&sort=all) | | | transferase | | **144.6** | 80.7 | 16919.9 | 2136.5 | 9332.7 | 8591.3 |
| 68 | [At4g04760](http://atted.jp/cgi-bin/coex_Ath.cgi?gene=At4g04760&sort=all) | | | transporter | | **144.9** | 55.5 | 19669.7 | 2587.9 | 14063.4 | 13160.4 |
| 69 | [At2g46130](http://atted.jp/cgi-bin/coex_Ath.cgi?gene=At2g46130&sort=all) | | | WRKY43 | | **148.2** | 469.6 | 83.8 | 15952.2 | 346.8 | 12151.5 |
| 70 | [At4g24140](http://atted.jp/cgi-bin/coex_Ath.cgi?gene=At4g24140&sort=all) | | | hydrolase | | **149.5** | 960.1 | 57.0 | 3573.1 | 147.2 | 2158.7 |
| 71 | [At1g22015](http://atted.jp/cgi-bin/coex_Ath.cgi?gene=At1g22015&sort=all) | | | DD46 | | **150.0** | 120.5 | 9325.3 | 20270.4 | 8779.3 | 7797.3 |
| 72 | [At1g17950](http://atted.jp/cgi-bin/coex_Ath.cgi?gene=At1g17950&sort=all) | | | MYB52 | | **152.3** | 804.9 | 140.5 | 8520.6 | 301.4 | 10796.7 |
| 73 | [At1g79780](http://atted.jp/cgi-bin/coex_Ath.cgi?gene=At1g79780&sort=all) | | |  | | **152.9** | 140.0 | 5068.4 | 20337.8 | 4733.7 | 3050.9 |
| 74 | [At3g51750](http://atted.jp/cgi-bin/coex_Ath.cgi?gene=At3g51750&sort=all) | | |  | | **157.4** | 716.3 | 394.5 | 4328.5 | 75.4 | 18038.5 |
| 75 | [At1g23560](http://atted.jp/cgi-bin/coex_Ath.cgi?gene=At1g23560&sort=all) | | |  | | **159.1** | 137.3 | 2427.4 | 17458.0 | 5862.8 | 3469.3 |
| 76 | [At1g24600](http://atted.jp/cgi-bin/coex_Ath.cgi?gene=At1g24600&sort=all) | | |  | | **164.7** | 342.8 | 733.0 | 7000.9 | 42.2 | 3268.6 |
| 77 | [At3g16280](http://atted.jp/cgi-bin/coex_Ath.cgi?gene=At3g16280&sort=all) | | | transcription | | **171.2** | 408.7 | 606.3 | 10444.2 | 8114.4 | 2489.3 |
| 78 | [At5g65205](http://atted.jp/cgi-bin/coex_Ath.cgi?gene=At5g65205&sort=all) | | | SDR | | **171.3** | 87.9 | 8891.8 | 7892.5 | 11531.5 | 7464.0 |
| 79 | [At3g23840](http://atted.jp/cgi-bin/coex_Ath.cgi?gene=At3g23840&sort=all) | | | transferase | | **171.5** | 170.3 | 5691.7 | 6739.0 | 4960.3 | 1664.8 |
| 80 | [At5g17200](http://atted.jp/cgi-bin/coex_Ath.cgi?gene=At5g17200&sort=all) | | | pectinase | | **171.6** | 92.9 | 5203.0 | 4593.1 | 17984.1 | 11691.6 |
| 81 | [At3g51590](http://atted.jp/cgi-bin/coex_Ath.cgi?gene=At3g51590&sort=all) | | | LTP12 | | **173.0** | 168.4 | 4568.2 | 15403.0 | 10598.6 | 8353.0 |
| 82 | [At4g37900](http://atted.jp/cgi-bin/coex_Ath.cgi?gene=At4g37900&sort=all) | | | glycine-rich | | **179.2** | 286.8 | 3250.4 | 12324.0 | 14844.6 | 7459.8 |
| 83 | [At1g68875](http://atted.jp/cgi-bin/coex_Ath.cgi?gene=At1g68875&sort=all) | | |  | | **180.9** | 176.1 | 11247.3 | 5597.2 | 17405.3 | 142.0 |
| 84 | [At1g47980](http://atted.jp/cgi-bin/coex_Ath.cgi?gene=At1g47980&sort=all) | | |  | | **184.8** | 212.4 | 391.1 | 16671.3 | 13464.0 | 3532.9 |
| 85 | [At1g64000](http://atted.jp/cgi-bin/coex_Ath.cgi?gene=At1g64000&sort=all) | | | WRKY56 | | **185.3** | 1300.4 | 31.4 | 5617.6 | 284.5 | 9063.3 |
| 86 | [At2g03200](http://atted.jp/cgi-bin/coex_Ath.cgi?gene=At2g03200&sort=all) | | | protease | | **190.6** | 621.4 | 7.3 | 19227.2 | 91.8 | 46.1 |
| 87 | [At3g28470](http://atted.jp/cgi-bin/coex_Ath.cgi?gene=At3g28470&sort=all) | | | TDF1 | | **196.8** | 141.3 | 6945.7 | 15362.7 | 8843.9 | 17743.9 |
| 88 | [At3g15400](http://atted.jp/cgi-bin/coex_Ath.cgi?gene=At3g15400&sort=all) | | | ATA20 | | **199.3** | 306.2 | 71.5 | 20804.4 | 13664.4 | 9715.3 |
| 89 | 259905_s_at | | |  | | **209.7** | 132.8 | 7561.3 | 2399.4 | 7672.4 | 10775.9 |
| 90 | [At2g34610](http://atted.jp/cgi-bin/coex_Ath.cgi?gene=At2g34610&sort=all) | | |  | | **216.4** | 3157.1 | 250.5 | 18406.0 | 78.5 | 1022.3 |
| 91 | [At1g75940](http://atted.jp/cgi-bin/coex_Ath.cgi?gene=At1g75940&sort=all) | | | ATA27 | | **221.3** | 220.2 | 15086.1 | 4835.1 | 12067.7 | 5293.2 |
| 92 | [At5g41890](http://atted.jp/cgi-bin/coex_Ath.cgi?gene=At5g41890&sort=all) | | | hydrolase | | **225.0** | 134.1 | 2802.5 | 10735.2 | 6435.3 | 9643.8 |
| 93 | [At5g43340](http://atted.jp/cgi-bin/coex_Ath.cgi?gene=At5g43340&sort=all) | | | PHT6 | | **228.3** | 180.7 | 7900.6 | 6016.8 | 9196.4 | 3604.9 |
| 94 | [At1g54540](http://atted.jp/cgi-bin/coex_Ath.cgi?gene=At1g54540&sort=all) | | |  | | **229.7** | 689.3 | 265.5 | 8901.9 | 114.0 | 7406.9 |
| 95 | [At2g23945](http://atted.jp/cgi-bin/coex_Ath.cgi?gene=At2g23945&sort=all) | | | DNA-binding | | **238.6** | 101.8 | 5193.6 | 9738.5 | 3998.2 | 3164.8 |
| 96 | [At2g31035](http://atted.jp/cgi-bin/coex_Ath.cgi?gene=At2g31035&sort=all) | | |  | | **240.5** | 248.1 | 8406.4 | 2049.4 | 2977.9 | 10767.4 |
| 97 | [At1g28375](http://atted.jp/cgi-bin/coex_Ath.cgi?gene=At1g28375&sort=all) | | |  | | **241.0** | 257.6 | 6514.9 | 6259.3 | 14550.1 | 17327.8 |
| 98 | [At5g53190](http://atted.jp/cgi-bin/coex_Ath.cgi?gene=At5g53190&sort=all) | | | MtN3 | | **241.9** | 308.9 | 837.7 | 6880.2 | 6974.3 | 4307.8 |
| 99 | [At2g22240](http://atted.jp/cgi-bin/coex_Ath.cgi?gene=At2g22240&sort=all) | | | MIPS2 | | **243.7** | 1566.4 | 272.8 | 17414.6 | 23.4 | 13107.5 |
| 100 | [At5g23190](http://atted.jp/cgi-bin/coex_Ath.cgi?gene=At5g23190&sort=all) | | | CYP86B1 | | **245.8** | 707.0 | 148.1 | 15141.1 | 146.5 | 2620.5 |
| 101 | [At2g03740](http://atted.jp/cgi-bin/coex_Ath.cgi?gene=At2g03740&sort=all) | | | LEA | | **245.9** | 211.4 | 11346.0 | 12606.3 | 16433.0 | 6372.2 |
| 102 | [At5g28510](http://atted.jp/cgi-bin/coex_Ath.cgi?gene=At5g28510&sort=all) | | | BGLU24 | | **246.0** | 9461.6 | 144.1 | 22224.7 | 57.1 | 839.6 |
| 103 | [At1g54940](http://atted.jp/cgi-bin/coex_Ath.cgi?gene=At1g54940&sort=all) | | | PGSIP4 | | **246.8** | 108.8 | 6132.2 | 10026.8 | 15802.3 | 4863.1 |
| 104 | [At2g24140](http://atted.jp/cgi-bin/coex_Ath.cgi?gene=At2g24140&sort=all) | | |  | | **246.9** | 13606.2 | 9.5 | 2929.7 | 1514.6 | 4432.1 |
| 105 | [At5g17830](http://atted.jp/cgi-bin/coex_Ath.cgi?gene=At5g17830&sort=all) | | |  | | **248.4** | 117.9 | 13729.4 | 13661.1 | 10860.4 | 12649.5 |
| 106 | [At2g23510](http://atted.jp/cgi-bin/coex_Ath.cgi?gene=At2g23510&sort=all) | | | SDT | | **253.3** | 362.3 | 1425.5 | 16766.8 | 12198.6 | 6416.7 |
| 107 | [At4g24480](http://atted.jp/cgi-bin/coex_Ath.cgi?gene=At4g24480&sort=all) | | | kinase | | **254.2** | 2282.4 | 357.8 | 21493.8 | 244.4 | 1538.8 |
| 108 | [252088_at](http://atted.jp/cgi-bin/coex_Ath.cgi?gene=252088_at&sort=all) | | |  | | **254.6** | 1041.1 | 216.4 | 3238.2 | 956.4 | 4025.3 |
| 109 | [At5g63560](http://atted.jp/cgi-bin/coex_Ath.cgi?gene=At5g63560&sort=all) | | | transferase | | **257.2** | 882.9 | 79.1 | 2175.1 | 455.5 | 687.4 |
| 110 | [At3g50980](http://atted.jp/cgi-bin/coex_Ath.cgi?gene=At3g50980&sort=all) | | | XERO1 | | **262.0** | 1205.8 | 33.0 | 15752.0 | 887.8 | 971.6 |
| 111 | [At1g44224](http://atted.jp/cgi-bin/coex_Ath.cgi?gene=At1g44224&sort=all) | | |  | | **265.7** | 171.3 | 15110.7 | 18028.9 | 6741.2 | 12273.6 |
| 112 | 254567_s_at | | |  | | **269.1** | 129.2 | 5006.0 | 1271.7 | 13727.3 | 17649.5 |
| 113 | [At5g25950](http://atted.jp/cgi-bin/coex_Ath.cgi?gene=At5g25950&sort=all) | | |  | | **271.7** | 415.3 | 1169.4 | 21794.0 | 7120.8 | 6366.4 |
| 114 | [At3g52820](http://atted.jp/cgi-bin/coex_Ath.cgi?gene=At3g52820&sort=all) | | | PAP22 | | **274.3** | 671.1 | 296.2 | 7871.4 | 70.3 | 1196.1 |
| 115 | [At1g13150](http://atted.jp/cgi-bin/coex_Ath.cgi?gene=At1g13150&sort=all) | | | CYP86C4 | | **277.4** | 241.6 | 8477.0 | 16017.0 | 10148.9 | 14729.2 |
| 116 | [At2g29380](http://atted.jp/cgi-bin/coex_Ath.cgi?gene=At2g29380&sort=all) | | | HAI3 | | **278.1** | 2895.0 | 255.7 | 10203.8 | 44.5 | 756.5 |
| 117 | [At2g41290](http://atted.jp/cgi-bin/coex_Ath.cgi?gene=At2g41290&sort=all) | | | SSL2 | | **279.1** | 257.8 | 5634.3 | 1729.5 | 16168.7 | 7148.3 |
| 118 | [At3g06390](http://atted.jp/cgi-bin/coex_Ath.cgi?gene=At3g06390&sort=all) | | | integral membrane | | **279.1** | 1852.0 | 4.0 | 11734.9 | 2048.4 | 6.2 |
| 119 | [At5g04000](http://atted.jp/cgi-bin/coex_Ath.cgi?gene=At5g04000&sort=all) | | |  | | **280.3** | 528.0 | 983.2 | 21270.0 | 358.1 | 8669.3 |
| 120 | [At4g27420](http://atted.jp/cgi-bin/coex_Ath.cgi?gene=At4g27420&sort=all) | | | transporter | | **285.3** | 315.2 | 5056.5 | 14018.2 | 8021.2 | 10668.1 |
| 121 | [At1g73410](http://atted.jp/cgi-bin/coex_Ath.cgi?gene=At1g73410&sort=all) | | | MYB54 | | **289.1** | 1492.1 | 274.9 | 15877.5 | 101.3 | 17005.6 |
| 122 | [At3g50580](http://atted.jp/cgi-bin/coex_Ath.cgi?gene=At3g50580&sort=all) | | |  | | **289.5** | 316.4 | 1608.7 | 18345.5 | 6349.7 | 9670.4 |
| 123 | [At1g06260](http://atted.jp/cgi-bin/coex_Ath.cgi?gene=At1g06260&sort=all) | | | cysteinease | | **293.3** | 262.7 | 19230.0 | 4818.4 | 8403.8 | 10412.9 |
| 124 | [At5g07330](http://atted.jp/cgi-bin/coex_Ath.cgi?gene=At5g07330&sort=all) | | |  | | **295.1** | 1086.7 | 572.0 | 12049.5 | 44.4 | 2906.0 |
| 125 | [At1g79330](http://atted.jp/cgi-bin/coex_Ath.cgi?gene=At1g79330&sort=all) | | | ATMC5 | | **301.0** | 884.8 | 75.0 | 21265.6 | 747.0 | 17086.8 |
| 126 | [At2g22510](http://atted.jp/cgi-bin/coex_Ath.cgi?gene=At2g22510&sort=all) | | | glycoprotein | | **301.0** | 1931.7 | 4.0 | 17431.8 | 164.5 | 15.5 |
| 127 | [At4g23660](http://atted.jp/cgi-bin/coex_Ath.cgi?gene=At4g23660&sort=all) | | | PPT1 | | **305.1** | 342.9 | 2644.8 | 12387.2 | 4357.1 | 1805.0 |
| 128 | [At5g41090](http://atted.jp/cgi-bin/coex_Ath.cgi?gene=At5g41090&sort=all) | | | anac095 | | **309.0** | 417.2 | 1175.0 | 2603.1 | 13108.3 | 17732.6 |
| 129 | [At2g43390](http://atted.jp/cgi-bin/coex_Ath.cgi?gene=At2g43390&sort=all) | | |  | | **313.1** | 1566.3 | 263.6 | 22426.0 | 883.7 | 100.1 |
| 130 | [At1g75920](http://atted.jp/cgi-bin/coex_Ath.cgi?gene=At1g75920&sort=all) | | | EXL5 | | **314.8** | 263.2 | 15165.9 | 9911.1 | 11290.9 | 4594.4 |
| 131 | [At1g53270](http://atted.jp/cgi-bin/coex_Ath.cgi?gene=At1g53270&sort=all) | | | transporter | | **317.8** | 1084.1 | 224.1 | 12045.0 | 172.9 | 7919.1 |
| 132 | [At5g49350](http://atted.jp/cgi-bin/coex_Ath.cgi?gene=At5g49350&sort=all) | | |  | | **317.8** | 1686.2 | 88.2 | 18042.2 | 1649.9 | 441.1 |
| 133 | [At1g14100](http://atted.jp/cgi-bin/coex_Ath.cgi?gene=At1g14100&sort=all) | | | FUT8 | | **320.3** | 74.2 | 7763.6 | 7611.0 | 9655.8 | 9011.6 |
| 134 | [At2g19900](http://atted.jp/cgi-bin/coex_Ath.cgi?gene=At2g19900&sort=all) | | | NADP-ME1 | | **320.9** | 1208.3 | 575.8 | 7276.8 | 213.1 | 1825.8 |
| 135 | [At1g17310](http://atted.jp/cgi-bin/coex_Ath.cgi?gene=At1g17310&sort=all) | | | AGL100 | | **328.1** | 2592.0 | 10.1 | 19594.7 | 2149.8 | 677.7 |
| 136 | [At1g66460](http://atted.jp/cgi-bin/coex_Ath.cgi?gene=At1g66460&sort=all) | | | kinase | | **329.2** | 409.3 | 10731.8 | 21530.9 | 1350.5 | 3176.1 |
| 137 | [At5g06839](http://atted.jp/cgi-bin/coex_Ath.cgi?gene=At5g06839&sort=all) | | | transcription | | **330.4** | 372.2 | 5913.1 | 4819.8 | 5028.3 | 738.9 |
| 138 | [At1g75910](http://atted.jp/cgi-bin/coex_Ath.cgi?gene=At1g75910&sort=all) | | | EXL4 | | **330.7** | 327.9 | 12648.2 | 17569.2 | 6849.3 | 14632.9 |
| 139 | [At2g37360](http://atted.jp/cgi-bin/coex_Ath.cgi?gene=At2g37360&sort=all) | | | transporter | | **333.7** | 1133.4 | 521.4 | 13788.4 | 177.4 | 3626.5 |
| 140 | [At5g66780](http://atted.jp/cgi-bin/coex_Ath.cgi?gene=At5g66780&sort=all) | | |  | | **340.1** | 1379.5 | 595.9 | 15984.6 | 65.9 | 18149.0 |
| 141 | [At1g72230](http://atted.jp/cgi-bin/coex_Ath.cgi?gene=At1g72230&sort=all) | | | plastocyanin-like | | **341.2** | 298.7 | 9884.1 | 7677.5 | 666.3 | 7139.8 |
| 142 | [At3g55090](http://atted.jp/cgi-bin/coex_Ath.cgi?gene=At3g55090&sort=all) | | | ATPase | | **345.8** | 898.9 | 1025.2 | 801.7 | 358.8 | 4247.4 |
| 143 | [At1g66850](http://atted.jp/cgi-bin/coex_Ath.cgi?gene=At1g66850&sort=all) | | | LTP | | **349.4** | 428.9 | 2179.9 | 8429.6 | 17410.2 | 8414.5 |
| 144 | [At2g43670](http://atted.jp/cgi-bin/coex_Ath.cgi?gene=At2g43670&sort=all) | | | hydrolase | | **349.8** | 1693.7 | 272.9 | 1749.5 | 360.6 | 63.8 |
| 145 | [At4g37050](http://atted.jp/cgi-bin/coex_Ath.cgi?gene=At4g37050&sort=all) | | | PLP4 | | **351.8** | 2003.0 | 32.0 | 11396.6 | 2790.1 | 1035.7 |
| 146 | [At1g74550](http://atted.jp/cgi-bin/coex_Ath.cgi?gene=At1g74550&sort=all) | | | CYP98A9 | | **351.9** | 229.9 | 14128.8 | 10074.8 | 11149.3 | 1101.7 |
| 147 | [At4g03140](http://atted.jp/cgi-bin/coex_Ath.cgi?gene=At4g03140&sort=all) | | | oxidoreductase | | **352.1** | 2746.2 | 101.5 | 8191.3 | 149.3 | 21849.8 |
| 148 | [At4g09600](http://atted.jp/cgi-bin/coex_Ath.cgi?gene=At4g09600&sort=all) | | | GASA3 | | **352.2** | 799.3 | 1451.8 | 17030.7 | 224.7 | 11043.9 |
| 149 | [At3g26125](http://atted.jp/cgi-bin/coex_Ath.cgi?gene=At3g26125&sort=all) | | | CYP86C2 | | **356.4** | 300.9 | 18412.5 | 4046.0 | 5915.5 | 20961.0 |
| 150 | [At3g60220](http://atted.jp/cgi-bin/coex_Ath.cgi?gene=At3g60220&sort=all) | | | ATL4 | | **360.9** | 646.8 | 2734.8 | 8269.3 | 87.8 | 3025.8 |
| 151 | [At5g19410](http://atted.jp/cgi-bin/coex_Ath.cgi?gene=At5g19410&sort=all) | | | transporter | | **361.5** | 2221.9 | 104.0 | 15226.4 | 100.2 | 552.6 |
| 152 | [At1g08320](http://atted.jp/cgi-bin/coex_Ath.cgi?gene=At1g08320&sort=all) | | | transcription | | **365.2** | 423.6 | 4387.5 | 17180.7 | 4905.4 | 2032.6 |
| 153 | [At1g04360](http://atted.jp/cgi-bin/coex_Ath.cgi?gene=At1g04360&sort=all) | | | zinc finger | | **366.9** | 2000.4 | 416.1 | 9433.5 | 12.4 | 1685.5 |
| 154 | [At5g04370](http://atted.jp/cgi-bin/coex_Ath.cgi?gene=At5g04370&sort=all) | | | NAMT1 | | **370.9** | 1480.8 | 915.0 | 6679.3 | 33.5 | 16130.9 |
| 155 | [At3g30460](http://atted.jp/cgi-bin/coex_Ath.cgi?gene=At3g30460&sort=all) | | | zinc finger | | **371.7** | 425.9 | 3684.2 | 6483.6 | 2107.2 | 11705.5 |
| 156 | [At2g46750](http://atted.jp/cgi-bin/coex_Ath.cgi?gene=At2g46750&sort=all) | | | FAD-binding | | **373.6** | 961.6 | 340.2 | 18619.9 | 13802.0 | 733.2 |
| 157 | [At2g01430](http://atted.jp/cgi-bin/coex_Ath.cgi?gene=At2g01430&sort=all) | | | ATHB17 | | **377.7** | 832.5 | 1108.0 | 20411.5 | 388.0 | 8222.3 |
| 158 | 249684_s_at | | |  | | **379.4** | 1726.9 | 172.9 | 18922.3 | 17850.5 | 194.7 |
| 159 | [At4g09300](http://atted.jp/cgi-bin/coex_Ath.cgi?gene=At4g09300&sort=all) | | |  | | **379.7** | 458.4 | 801.1 | 12557.4 | 8672.2 | 11484.1 |
| 160 | [At3g27270](http://atted.jp/cgi-bin/coex_Ath.cgi?gene=At3g27270&sort=all) | | |  | | **384.4** | 2890.5 | 390.3 | 10690.8 | 346.6 | 2103.6 |
| 161 | [At3g17520](http://atted.jp/cgi-bin/coex_Ath.cgi?gene=At3g17520&sort=all) | | | LEA | | **385.2** | 1796.7 | 823.8 | 11228.2 | 12.5 | 3883.7 |
| 162 | [At1g68850](http://atted.jp/cgi-bin/coex_Ath.cgi?gene=At1g68850&sort=all) | | | peroxidase | | **387.5** | 1568.3 | 110.0 | 17612.3 | 125.7 | 517.8 |
| 163 | 259221_s_at | | |  | | **389.0** | 672.1 | 841.5 | 16448.2 | 825.2 | 12128.8 |
| 164 | [At1g33700](http://atted.jp/cgi-bin/coex_Ath.cgi?gene=At1g33700&sort=all) | | | glucosylceramidase | | **391.2** | 526.6 | 1570.6 | 11171.4 | 11672.3 | 1414.6 |
| 165 | [At5g17300](http://atted.jp/cgi-bin/coex_Ath.cgi?gene=At5g17300&sort=all) | | | RVE1 | | **392.3** | 1819.0 | 550.1 | 72.6 | 3238.8 | 13843.0 |
| 166 | [At1g03790](http://atted.jp/cgi-bin/coex_Ath.cgi?gene=At1g03790&sort=all) | | | SOM | | **398.9** | 1856.1 | 157.8 | 12388.6 | 68.6 | 5139.9 |
| 167 | [At5g52420](http://atted.jp/cgi-bin/coex_Ath.cgi?gene=At5g52420&sort=all) | | |  | | **399.0** | 692.3 | 680.7 | 2988.3 | 728.4 | 14426.7 |
| 168 | [At2g03850](http://atted.jp/cgi-bin/coex_Ath.cgi?gene=At2g03850&sort=all) | | | LEA | | **399.9** | 898.6 | 125.5 | 14444.5 | 9705.4 | 6259.3 |
| 169 | [At3g24310](http://atted.jp/cgi-bin/coex_Ath.cgi?gene=At3g24310&sort=all) | | | MYB305 | | **400.6** | 9038.3 | 231.3 | 13190.1 | 237.4 | 10581.2 |
| 170 | [At5g47740](http://atted.jp/cgi-bin/coex_Ath.cgi?gene=At5g47740&sort=all) | | |  | | **406.1** | 2394.5 | 113.0 | 4699.3 | 6443.0 | 1945.7 |
| 171 | [At2g21820](http://atted.jp/cgi-bin/coex_Ath.cgi?gene=At2g21820&sort=all) | | |  | | **407.0** | 1609.1 | 537.6 | 13106.2 | 90.9 | 6313.3 |
| 172 | [At3g12720](http://atted.jp/cgi-bin/coex_Ath.cgi?gene=At3g12720&sort=all) | | | MYB67 | | **413.0** | 1384.7 | 394.4 | 19536.8 | 5433.5 | 18549.7 |
| 173 | [At5g59845](http://atted.jp/cgi-bin/coex_Ath.cgi?gene=At5g59845&sort=all) | | | gibberellin-regulated | | **413.9** | 1044.5 | 493.0 | 7589.5 | 29.5 | 22504.2 |
| 174 | [At5g62165](http://atted.jp/cgi-bin/coex_Ath.cgi?gene=At5g62165&sort=all) | | | AGL42 | | **421.2** | 1795.5 | 364.7 | 14975.1 | 58.0 | 22112.9 |
| 175 | [At3g50400](http://atted.jp/cgi-bin/coex_Ath.cgi?gene=At3g50400&sort=all) | | | hydrolase | | **422.0** | 3606.8 | 139.6 | 19797.8 | 44.5 | 7601.0 |
| 176 | [At1g06250](http://atted.jp/cgi-bin/coex_Ath.cgi?gene=At1g06250&sort=all) | | | lipase | | **423.3** | 472.4 | 4402.3 | 18968.0 | 11220.0 | 20293.3 |
| 177 | [At4g17215](http://atted.jp/cgi-bin/coex_Ath.cgi?gene=At4g17215&sort=all) | | |  | | **423.3** | 1794.3 | 102.5 | 17133.1 | 90.9 | 1386.3 |
| 178 | [At1g28470](http://atted.jp/cgi-bin/coex_Ath.cgi?gene=At1g28470&sort=all) | | | ANAC010 | | **425.8** | 1038.3 | 801.9 | 13225.2 | 3506.1 | 5123.1 |
| 179 | [At1g56320](http://atted.jp/cgi-bin/coex_Ath.cgi?gene=At1g56320&sort=all) | | |  | | **427.0** | 2417.9 | 96.7 | 1720.5 | 106.6 | 629.5 |
| 180 | [At1g17090](http://atted.jp/cgi-bin/coex_Ath.cgi?gene=At1g17090&sort=all) | | |  | | **439.4** | 183.8 | 9309.1 | 174.8 | 16957.4 | 11742.3 |
| 181 | [At1g03700](http://atted.jp/cgi-bin/coex_Ath.cgi?gene=At1g03700&sort=all) | | |  | | **440.2** | 1364.4 | 1571.2 | 2662.4 | 66.4 | 530.6 |
| 182 | [At2g01580](http://atted.jp/cgi-bin/coex_Ath.cgi?gene=At2g01580&sort=all) | | |  | | **440.8** | 2449.3 | 15.0 | 2074.0 | 1380.2 | 5449.0 |
| 183 | [At5g37680](http://atted.jp/cgi-bin/coex_Ath.cgi?gene=At5g37680&sort=all) | | | ARLA1A | | **443.4** | 665.4 | 1564.1 | 3218.8 | 8436.8 | 6443.2 |
| 184 | [At3g59530](http://atted.jp/cgi-bin/coex_Ath.cgi?gene=At3g59530&sort=all) | | | synthase | | **448.9** | 1115.7 | 1207.2 | 16702.3 | 9404.9 | 10472.5 |
| 185 | [At5g44700](http://atted.jp/cgi-bin/coex_Ath.cgi?gene=At5g44700&sort=all) | | | GSO2 | | **453.0** | 508.5 | 2835.2 | 18661.9 | 6929.6 | 6837.9 |
| 186 | [At1g23570](http://atted.jp/cgi-bin/coex_Ath.cgi?gene=At1g23570&sort=all) | | |  | | **454.5** | 526.3 | 5509.2 | 22237.5 | 8558.2 | 3930.5 |
| 187 | [At5g20860](http://atted.jp/cgi-bin/coex_Ath.cgi?gene=At5g20860&sort=all) | | | pectinesterase | | **456.9** | 1420.7 | 212.2 | 15457.3 | 613.1 | 4144.0 |
| 188 | [At5g07550](http://atted.jp/cgi-bin/coex_Ath.cgi?gene=At5g07550&sort=all) | | | GRP19 | | **458.6** | 455.1 | 9140.9 | 17951.4 | 15636.7 | 12697.6 |
| 189 | [At1g76230](http://atted.jp/cgi-bin/coex_Ath.cgi?gene=At1g76230&sort=all) | | |  | | **461.1** | 745.8 | 3294.7 | 5356.7 | 4529.8 | 7946.8 |
| 190 | [At2g23110](http://atted.jp/cgi-bin/coex_Ath.cgi?gene=At2g23110&sort=all) | | |  | | **461.2** | 1232.6 | 595.8 | 15877.6 | 637.9 | 14946.9 |
| 191 | [At3g15670](http://atted.jp/cgi-bin/coex_Ath.cgi?gene=At3g15670&sort=all) | | | LEA | | **463.0** | 1261.5 | 1337.9 | 19772.7 | 75.8 | 15817.4 |
| 192 | [253349_at](http://atted.jp/cgi-bin/coex_Ath.cgi?gene=253349_at&sort=all) | | |  | | **463.9** | 1606.4 | 686.8 | 15525.0 | 1437.5 | 20451.7 |
| 193 | [At2g47780](http://atted.jp/cgi-bin/coex_Ath.cgi?gene=At2g47780&sort=all) | | | REF | | **464.1** | 475.3 | 3148.2 | 8622.4 | 252.9 | 18323.1 |
| 194 | [At5g08430](http://atted.jp/cgi-bin/coex_Ath.cgi?gene=At5g08430&sort=all) | | | GYF | | **465.9** | 418.8 | 2450.7 | 6820.9 | 16680.5 | 3331.4 |
| 195 | [At4g27330](http://atted.jp/cgi-bin/coex_Ath.cgi?gene=At4g27330&sort=all) | | | SPL | | **469.5** | 389.9 | 11126.3 | 9631.7 | 9501.9 | 17634.2 |
| 196 | [At5g52300](http://atted.jp/cgi-bin/coex_Ath.cgi?gene=At5g52300&sort=all) | | | LTI65 | | **471.4** | 1597.5 | 1828.9 | 17937.6 | 23.5 | 181.5 |
| 197 | [At5g01670](http://atted.jp/cgi-bin/coex_Ath.cgi?gene=At5g01670&sort=all) | | | reductase | | **471.6** | 1195.0 | 1463.9 | 16520.3 | 81.5 | 15064.0 |
| 198 | [At5g07530](http://atted.jp/cgi-bin/coex_Ath.cgi?gene=At5g07530&sort=all) | | | GRP17 | | **473.7** | 479.7 | 6803.2 | 21341.2 | 12138.8 | 2249.6 |
| 199 | [At3g18400](http://atted.jp/cgi-bin/coex_Ath.cgi?gene=At3g18400&sort=all) | | | anac058 | | **479.8** | 1298.7 | 924.8 | 12027.6 | 309.5 | 2583.9 |
| 200 | [At1g68540](http://atted.jp/cgi-bin/coex_Ath.cgi?gene=At1g68540&sort=all) | | | oxidoreductase | | **479.9** | 121.5 | 4780.8 | 3564.1 | 5704.0 | 18710.5 |
| 201 | [At3g12955](http://atted.jp/cgi-bin/coex_Ath.cgi?gene=At3g12955&sort=all) | | | auxin-responsive | | **481.1** | 640.0 | 4477.7 | 4959.6 | 197.7 | 2902.1 |
| 202 | [At5g62490](http://atted.jp/cgi-bin/coex_Ath.cgi?gene=At5g62490&sort=all) | | | HVA22B | | **482.9** | 1090.0 | 2663.0 | 9151.9 | 51.9 | 11817.4 |
| 203 | [At2g19810](http://atted.jp/cgi-bin/coex_Ath.cgi?gene=At2g19810&sort=all) | | | CCCH-type | | **490.4** | 1526.3 | 3095.7 | 21419.5 | 89.6 | 10409.8 |
| 204 | [At1g71340](http://atted.jp/cgi-bin/coex_Ath.cgi?gene=At1g71340&sort=all) | | | phosphodiesterase | | **491.8** | 1215.5 | 1365.2 | 11854.6 | 2719.9 | 714.8 |
| 205 | [At1g79320](http://atted.jp/cgi-bin/coex_Ath.cgi?gene=At1g79320&sort=all) | | | MC6 | | **493.2** | 1975.0 | 315.8 | 7722.3 | 3346.6 | 1468.1 |
| 206 | [At5g49990](http://atted.jp/cgi-bin/coex_Ath.cgi?gene=At5g49990&sort=all) | | | permease | | **493.3** | 1201.7 | 800.8 | 19013.3 | 2428.5 | 591.5 |
| 207 | [At5g15720](http://atted.jp/cgi-bin/coex_Ath.cgi?gene=At5g15720&sort=all) | | | GLIP7 | | **493.8** | 644.2 | 4795.9 | 13971.4 | 12581.9 | 22595.5 |
| 208 | [At5g14980](http://atted.jp/cgi-bin/coex_Ath.cgi?gene=At5g14980&sort=all) | | | thioesterase | | **494.5** | 225.0 | 10121.1 | 15138.5 | 12664.4 | 9018.1 |
| 209 | [At1g55240](http://atted.jp/cgi-bin/coex_Ath.cgi?gene=At1g55240&sort=all) | | |  | | **494.8** | 2994.5 | 222.5 | 15300.7 | 347.9 | 4006.3 |
| 210 | [At4g01410](http://atted.jp/cgi-bin/coex_Ath.cgi?gene=At4g01410&sort=all) | | | HIN1 | | **495.6** | 361.2 | 7772.3 | 12145.3 | 20569.9 | 13578.3 |
| 211 | [At3g25050](http://atted.jp/cgi-bin/coex_Ath.cgi?gene=At3g25050&sort=all) | | | XTH3 | | **496.7** | 499.2 | 16442.7 | 19063.8 | 9220.5 | 20204.8 |
| 212 | [At1g76470](http://atted.jp/cgi-bin/coex_Ath.cgi?gene=At1g76470&sort=all) | | | reductase | | **497.1** | 256.0 | 2469.8 | 9084.9 | 18726.0 | 18607.5 |
| 213 | [At3g03310](http://atted.jp/cgi-bin/coex_Ath.cgi?gene=At3g03310&sort=all) | | | LCAT3 | | **500.6** | 950.6 | 776.5 | 8022.8 | 468.3 | 8473.9 |
| 214 | [At3g58550](http://atted.jp/cgi-bin/coex_Ath.cgi?gene=At3g58550&sort=all) | | | LTP | | **505.0** | 1492.5 | 312.8 | 19180.7 | 2974.2 | 236.2 |
| 215 | [At5g03180](http://atted.jp/cgi-bin/coex_Ath.cgi?gene=At5g03180&sort=all) | | | zinc finger | | **506.7** | 1024.9 | 577.7 | 8412.1 | 3617.5 | 3448.4 |
| 216 | [At5g07130](http://atted.jp/cgi-bin/coex_Ath.cgi?gene=At5g07130&sort=all) | | | LAC13 | | **511.7** | 1573.5 | 968.9 | 15560.7 | 122.8 | 6137.8 |
| 217 | [At2g31980](http://atted.jp/cgi-bin/coex_Ath.cgi?gene=At2g31980&sort=all) | | | inhibitor | | **511.8** | 764.3 | 1512.6 | 1015.6 | 197.0 | 17352.0 |
| 218 | [At5g12840](http://atted.jp/cgi-bin/coex_Ath.cgi?gene=At5g12840&sort=all) | | | NF-YA1 | | **513.2** | 2761.8 | 532.7 | 19419.2 | 220.6 | 4890.2 |
| 219 | [At5g07510](http://atted.jp/cgi-bin/coex_Ath.cgi?gene=At5g07510&sort=all) | | | GRP14 | | **513.6** | 526.1 | 5089.8 | 22273.7 | 7724.0 | 8039.5 |
| 220 | [At4g04460](http://atted.jp/cgi-bin/coex_Ath.cgi?gene=At4g04460&sort=all) | | | protease | | **518.4** | 854.6 | 1433.8 | 6541.5 | 3650.3 | 233.1 |
| 221 | [At2g33310](http://atted.jp/cgi-bin/coex_Ath.cgi?gene=At2g33310&sort=all) | | | IAA13 | | **518.4** | 1285.3 | 707.0 | 7765.2 | 2390.0 | 2015.6 |
| 222 | [At5g42290](http://atted.jp/cgi-bin/coex_Ath.cgi?gene=At5g42290&sort=all) | | | transcription | | **518.5** | 1247.3 | 2194.4 | 17411.1 | 52.7 | 8455.3 |
| 223 | [At5g37860](http://atted.jp/cgi-bin/coex_Ath.cgi?gene=At5g37860&sort=all) | | | copper-binding | | **518.8** | 154.4 | 12140.3 | 10617.6 | 11026.4 | 18398.7 |
| 224 | [At1g75930](http://atted.jp/cgi-bin/coex_Ath.cgi?gene=At1g75930&sort=all) | | | EXL6 | | **522.9** | 608.9 | 7918.7 | 15506.0 | 13725.7 | 9184.1 |
| 225 | [At2g44260](http://atted.jp/cgi-bin/coex_Ath.cgi?gene=At2g44260&sort=all) | | |  | | **528.7** | 1618.9 | 435.4 | 8949.2 | 4212.4 | 4906.8 |
| 226 | [At1g48940](http://atted.jp/cgi-bin/coex_Ath.cgi?gene=At1g48940&sort=all) | | | plastocyanin-like | | **533.2** | 640.8 | 4493.4 | 741.7 | 10327.5 | 12684.9 |
| 227 | [At4g17280](http://atted.jp/cgi-bin/coex_Ath.cgi?gene=At4g17280&sort=all) | | |  | | **533.3** | 1350.1 | 1612.2 | 14100.2 | 169.0 | 73.6 |
| 228 | [At2g48140](http://atted.jp/cgi-bin/coex_Ath.cgi?gene=At2g48140&sort=all) | | | EDA4 | | **533.8** | 1972.0 | 74.9 | 16345.7 | 155.2 | 1239.9 |
| 229 | [At3g16340](http://atted.jp/cgi-bin/coex_Ath.cgi?gene=At3g16340&sort=all) | | | PDR1 | | **538.0** | 737.5 | 3260.7 | 3357.5 | 270.9 | 6387.9 |
| 230 | [At5g57390](http://atted.jp/cgi-bin/coex_Ath.cgi?gene=At5g57390&sort=all) | | | AIL5 | | **543.2** | 779.4 | 4031.2 | 6531.0 | 3600.5 | 12868.8 |
| 231 | [At3g58000](http://atted.jp/cgi-bin/coex_Ath.cgi?gene=At3g58000&sort=all) | | | VQ motif | | **543.7** | 565.9 | 11240.2 | 10362.5 | 14044.1 | 4137.8 |
| 232 | [At3g62090](http://atted.jp/cgi-bin/coex_Ath.cgi?gene=At3g62090&sort=all) | | | PIL2 | | **544.6** | 1145.6 | 1420.1 | 18962.3 | 170.8 | 9976.5 |
| 233 | [At5g07520](http://atted.jp/cgi-bin/coex_Ath.cgi?gene=At5g07520&sort=all) | | | GRP18 | | **545.6** | 675.0 | 6073.6 | 10794.6 | 7531.5 | 7973.9 |
| 234 | [At3g57020](http://atted.jp/cgi-bin/coex_Ath.cgi?gene=At3g57020&sort=all) | | | synthase | | **547.9** | 2687.4 | 297.0 | 975.2 | 71.0 | 8916.7 |
| 235 | [At2g21100](http://atted.jp/cgi-bin/coex_Ath.cgi?gene=At2g21100&sort=all) | | | dirigent | | **549.3** | 1435.6 | 1179.8 | 20056.5 | 63.2 | 3913.4 |
| 236 | [At4g31830](http://atted.jp/cgi-bin/coex_Ath.cgi?gene=At4g31830&sort=all) | | |  | | **550.2** | 1945.3 | 462.7 | 14597.0 | 118.6 | 5557.3 |
| 237 | [At1g06990](http://atted.jp/cgi-bin/coex_Ath.cgi?gene=At1g06990&sort=all) | | | hydrolase | | **553.2** | 508.7 | 15554.5 | 14767.8 | 7068.8 | 13359.1 |
| 238 | [At3g12960](http://atted.jp/cgi-bin/coex_Ath.cgi?gene=At3g12960&sort=all) | | |  | | **555.4** | 774.7 | 6884.1 | 13237.0 | 531.5 | 3768.2 |
| 239 | [At5g14130](http://atted.jp/cgi-bin/coex_Ath.cgi?gene=At5g14130&sort=all) | | | peroxidase | | **558.5** | 2047.3 | 537.8 | 12640.1 | 28.8 | 5220.7 |
| 240 | [At3g57810](http://atted.jp/cgi-bin/coex_Ath.cgi?gene=At3g57810&sort=all) | | | protease | | **563.4** | 598.5 | 2511.3 | 9655.0 | 18622.0 | 16673.4 |
| 241 | [At1g49960](http://atted.jp/cgi-bin/coex_Ath.cgi?gene=At1g49960&sort=all) | | | permease | | **564.4** | 2622.6 | 165.5 | 5992.9 | 1944.6 | 169.5 |
| 242 | [At2g41040](http://atted.jp/cgi-bin/coex_Ath.cgi?gene=At2g41040&sort=all) | | | transferase | | **565.6** | 854.5 | 1953.4 | 10994.3 | 5771.3 | 17082.8 |
| 243 | [At3g51810](http://atted.jp/cgi-bin/coex_Ath.cgi?gene=At3g51810&sort=all) | | | EM1 | | **569.3** | 824.5 | 3104.4 | 20945.3 | 9803.1 | 1811.2 |
| 244 | [At4g10850](http://atted.jp/cgi-bin/coex_Ath.cgi?gene=At4g10850&sort=all) | | | MtN3 | | **571.9** | 679.2 | 4189.9 | 15059.0 | 7352.2 | 18985.6 |
| 245 | [At1g75050](http://atted.jp/cgi-bin/coex_Ath.cgi?gene=At1g75050&sort=all) | | |  | | **572.7** | 1161.8 | 376.0 | 2127.2 | 7748.6 | 4915.9 |
| 246 | [At3g15790](http://atted.jp/cgi-bin/coex_Ath.cgi?gene=At3g15790&sort=all) | | | MBD11 | | **575.6** | 1998.4 | 1011.0 | 11930.2 | 375.9 | 8538.8 |
| 247 | [At3g03170](http://atted.jp/cgi-bin/coex_Ath.cgi?gene=At3g03170&sort=all) | | |  | | **578.5** | 1481.2 | 2052.8 | 16102.8 | 440.2 | 3911.6 |
| 248 | [At2g18370](http://atted.jp/cgi-bin/coex_Ath.cgi?gene=At2g18370&sort=all) | | | LTP | | **584.7** | 1894.9 | 305.0 | 14036.4 | 1459.8 | 42.9 |
| 249 | [At5g51760](http://atted.jp/cgi-bin/coex_Ath.cgi?gene=At5g51760&sort=all) | | | AHG1 | | **587.5** | 1137.7 | 1022.0 | 7277.6 | 8667.6 | 7727.1 |
| 250 | [At5g07560](http://atted.jp/cgi-bin/coex_Ath.cgi?gene=At5g07560&sort=all) | | | GRP20 | | **589.1** | 699.2 | 12284.9 | 9957.8 | 11841.6 | 4910.5 |
| 251 | [At5g54570](http://atted.jp/cgi-bin/coex_Ath.cgi?gene=At5g54570&sort=all) | | | BGLU41 | | **591.0** | 1278.7 | 256.8 | 9220.5 | 3740.2 | 10909.5 |
| 252 | [At3g29630](http://atted.jp/cgi-bin/coex_Ath.cgi?gene=At3g29630&sort=all) | | | transferase | | **593.9** | 2506.2 | 329.1 | 1058.1 | 69.1 | 4672.7 |
| 253 | [At5g57400](http://atted.jp/cgi-bin/coex_Ath.cgi?gene=At5g57400&sort=all) | | |  | | **598.6** | 762.6 | 6163.3 | 4108.6 | 553.4 | 7859.6 |
| 254 | [At1g14930](http://atted.jp/cgi-bin/coex_Ath.cgi?gene=At1g14930&sort=all) | | | MLP | | **603.9** | 875.7 | 2701.2 | 18769.7 | 6989.1 | 15928.6 |
| 255 | [At2g47200](http://atted.jp/cgi-bin/coex_Ath.cgi?gene=At2g47200&sort=all) | | |  | | **607.9** | 2096.3 | 570.2 | 11111.4 | 619.9 | 30.0 |
| 256 | [At1g15330](http://atted.jp/cgi-bin/coex_Ath.cgi?gene=At1g15330&sort=all) | | | CBS | | **608.6** | 1681.1 | 952.8 | 14964.6 | 2592.3 | 14581.6 |
| 257 | [At1g04220](http://atted.jp/cgi-bin/coex_Ath.cgi?gene=At1g04220&sort=all) | | | KCS2 | | **609.4** | 2631.7 | 677.1 | 9122.5 | 119.5 | 2575.2 |
| 258 | [At3g58290](http://atted.jp/cgi-bin/coex_Ath.cgi?gene=At3g58290&sort=all) | | | MATH | | **615.3** | 483.5 | 8983.3 | 3032.6 | 12154.8 | 8493.0 |
| 259 | [At3g30210](http://atted.jp/cgi-bin/coex_Ath.cgi?gene=At3g30210&sort=all) | | | MYB121 | | **616.0** | 9194.1 | 276.8 | 19419.9 | 77.5 | 11103.1 |
| 260 | [At1g31470](http://atted.jp/cgi-bin/coex_Ath.cgi?gene=At1g31470&sort=all) | | | NFD4 | | **620.9** | 864.1 | 3469.9 | 7801.3 | 141.1 | 15980.0 |
| 261 | [At4g16270](http://atted.jp/cgi-bin/coex_Ath.cgi?gene=At4g16270&sort=all) | | | P40 | | **628.6** | 557.8 | 14666.7 | 19681.2 | 8890.5 | 15183.1 |
| 262 | [At3g44540](http://atted.jp/cgi-bin/coex_Ath.cgi?gene=At3g44540&sort=all) | | | FAR4 | | **629.1** | 2856.7 | 192.7 | 13879.7 | 63.2 | 246.6 |
| 263 | [At3g27200](http://atted.jp/cgi-bin/coex_Ath.cgi?gene=At3g27200&sort=all) | | | plastocyanin-like | | **629.6** | 524.4 | 9439.9 | 698.6 | 734.6 | 21276.2 |
| 264 | [At3g03880](http://atted.jp/cgi-bin/coex_Ath.cgi?gene=At3g03880&sort=all) | | |  | | **633.4** | 1402.4 | 718.2 | 18393.6 | 17235.4 | 18322.1 |
| 265 | [At5g66110](http://atted.jp/cgi-bin/coex_Ath.cgi?gene=At5g66110&sort=all) | | | metal ion binding | | **636.1** | 1656.4 | 1071.8 | 20891.8 | 363.2 | 10823.4 |
| 266 | [At2g41280](http://atted.jp/cgi-bin/coex_Ath.cgi?gene=At2g41280&sort=all) | | | M10 | | **637.2** | 910.8 | 6726.5 | 13728.7 | 4202.9 | 9175.1 |
| 267 | [At4g27530](http://atted.jp/cgi-bin/coex_Ath.cgi?gene=At4g27530&sort=all) | | |  | | **638.5** | 1053.8 | 4975.9 | 4197.7 | 558.4 | 2712.8 |
| 268 | [At5g60080](http://atted.jp/cgi-bin/coex_Ath.cgi?gene=At5g60080&sort=all) | | | kinase | | **639.1** | 192.3 | 9955.8 | 10825.0 | 2678.9 | 1183.1 |
| 269 | [At2g25900](http://atted.jp/cgi-bin/coex_Ath.cgi?gene=At2g25900&sort=all) | | | CTH | | **639.5** | 449.1 | 2288.9 | 9646.3 | 1967.5 | 10008.5 |
| 270 | [At2g21490](http://atted.jp/cgi-bin/coex_Ath.cgi?gene=At2g21490&sort=all) | | | LEA | | **642.2** | 697.6 | 9016.7 | 18670.9 | 7058.3 | 1926.3 |
| 271 | [At1g23700](http://atted.jp/cgi-bin/coex_Ath.cgi?gene=At1g23700&sort=all) | | | kinase | | **642.8** | 108.2 | 13864.2 | 5166.2 | 11684.0 | 13417.8 |
| 272 | [At5g44670](http://atted.jp/cgi-bin/coex_Ath.cgi?gene=At5g44670&sort=all) | | |  | | **647.4** | 1888.2 | 2391.8 | 290.0 | 1044.5 | 3070.5 |
| 273 | [At3g07970](http://atted.jp/cgi-bin/coex_Ath.cgi?gene=At3g07970&sort=all) | | | QRT2 | | **647.8** | 2716.7 | 552.8 | 4379.4 | 2088.9 | 4383.3 |
| 274 | [At3g10340](http://atted.jp/cgi-bin/coex_Ath.cgi?gene=At3g10340&sort=all) | | | PAL4 | | **649.2** | 2940.5 | 78.2 | 8738.6 | 47.6 | 1252.6 |
| 275 | [At5g57790](http://atted.jp/cgi-bin/coex_Ath.cgi?gene=At5g57790&sort=all) | | |  | | **653.3** | 549.7 | 9708.3 | 17832.5 | 2158.5 | 13848.9 |
| 276 | [At3g12920](http://atted.jp/cgi-bin/coex_Ath.cgi?gene=At3g12920&sort=all) | | | protein binding | | **653.5** | 532.1 | 6027.5 | 4191.0 | 2479.6 | 8294.0 |
| 277 | [At1g64030](http://atted.jp/cgi-bin/coex_Ath.cgi?gene=At1g64030&sort=all) | | | SRP3 | | **659.7** | 52.5 | 17936.0 | 12016.2 | 9195.7 | 16868.0 |
| 278 | 258579_s_at | | |  | | **663.5** | 1016.1 | 5601.6 | 1949.7 | 7735.0 | 18793.3 |
| 279 | [247765_at](http://atted.jp/cgi-bin/coex_Ath.cgi?gene=247765_at&sort=all) | | |  | | **663.6** | 2526.2 | 135.6 | 13088.6 | 719.3 | 4.0 |
| 280 | [At1g01120](http://atted.jp/cgi-bin/coex_Ath.cgi?gene=At1g01120&sort=all) | | | KCS1 | | **665.7** | 1801.3 | 808.4 | 2548.5 | 702.5 | 1492.0 |
| 281 | [At1g07150](http://atted.jp/cgi-bin/coex_Ath.cgi?gene=At1g07150&sort=all) | | | MAPKKK13 | | **666.5** | 2191.0 | 79.1 | 18857.0 | 5887.5 | 18559.5 |
| 282 | [At5g45310](http://atted.jp/cgi-bin/coex_Ath.cgi?gene=At5g45310&sort=all) | | |  | | **668.1** | 2020.0 | 1292.4 | 18456.0 | 111.1 | 4998.4 |
| 283 | [At3g60730](http://atted.jp/cgi-bin/coex_Ath.cgi?gene=At3g60730&sort=all) | | | pectinesterase | | **671.4** | 981.7 | 8784.0 | 13802.4 | 10072.1 | 16078.6 |
| 284 | [At1g04560](http://atted.jp/cgi-bin/coex_Ath.cgi?gene=At1g04560&sort=all) | | | AWPM-19-like membrane | | **677.1** | 2192.3 | 365.4 | 17692.0 | 73.6 | 8321.3 |
| 285 | [At1g61110](http://atted.jp/cgi-bin/coex_Ath.cgi?gene=At1g61110&sort=all) | | | anac025 | | **680.1** | 778.5 | 9024.9 | 8154.1 | 12431.6 | 11410.6 |
| 286 | [At1g56360](http://atted.jp/cgi-bin/coex_Ath.cgi?gene=At1g56360&sort=all) | | | PAP6 | | **681.5** | 751.5 | 6120.2 | 15945.1 | 9005.6 | 13197.9 |
| 287 | [At1g69800](http://atted.jp/cgi-bin/coex_Ath.cgi?gene=At1g69800&sort=all) | | | CBS | | **682.4** | 1528.3 | 469.8 | 3119.9 | 3175.6 | 10239.9 |
| 288 | [At1g29160](http://atted.jp/cgi-bin/coex_Ath.cgi?gene=At1g29160&sort=all) | | | zinc finger | | **682.5** | 620.6 | 6275.3 | 3344.9 | 18999.8 | 2645.4 |
| 289 | [At1g64110](http://atted.jp/cgi-bin/coex_Ath.cgi?gene=At1g64110&sort=all) | | | ATPase | | **683.4** | 3504.1 | 622.6 | 14881.3 | 313.4 | 20689.7 |
| 290 | [At5g56620](http://atted.jp/cgi-bin/coex_Ath.cgi?gene=At5g56620&sort=all) | | | anac099 | | **683.5** | 3855.3 | 108.0 | 17308.1 | 6080.2 | 2880.6 |
| 291 | [At1g61065](http://atted.jp/cgi-bin/coex_Ath.cgi?gene=At1g61065&sort=all) | | |  | | **687.2** | 407.7 | 4528.0 | 5188.6 | 8522.9 | 13801.8 |
| 292 | [At4g09490](http://atted.jp/cgi-bin/coex_Ath.cgi?gene=At4g09490&sort=all) | | | RNase H | | **687.4** | 750.2 | 4169.9 | 7561.8 | 1239.3 | 16385.5 |
| 293 | [At1g48840](http://atted.jp/cgi-bin/coex_Ath.cgi?gene=At1g48840&sort=all) | | |  | | **689.6** | 1329.8 | 1348.0 | 13276.3 | 269.3 | 15513.4 |
| 294 | [At4g18550](http://atted.jp/cgi-bin/coex_Ath.cgi?gene=At4g18550&sort=all) | | | lipase | | **690.5** | 4077.4 | 74.0 | 19114.2 | 162.6 | 1368.7 |
| 295 | [At5g39720](http://atted.jp/cgi-bin/coex_Ath.cgi?gene=At5g39720&sort=all) | | | AIG2L | | **694.8** | 1464.1 | 427.5 | 5861.5 | 221.4 | 3107.5 |
| 296 | [At1g44970](http://atted.jp/cgi-bin/coex_Ath.cgi?gene=At1g44970&sort=all) | | | peroxidase | | **695.3** | 1522.5 | 3400.2 | 8462.6 | 12560.7 | 2418.7 |
| 297 | [At2g22800](http://atted.jp/cgi-bin/coex_Ath.cgi?gene=At2g22800&sort=all) | | | HAT9 | | **696.3** | 1428.8 | 1995.2 | 17199.3 | 6732.1 | 8609.0 |
| 298 | [At2g40170](http://atted.jp/cgi-bin/coex_Ath.cgi?gene=At2g40170&sort=all) | | | GEA6 | | **700.9** | 1113.2 | 2024.6 | 17779.7 | 3926.9 | 3618.4 |
| 299 | [At1g07500](http://atted.jp/cgi-bin/coex_Ath.cgi?gene=At1g07500&sort=all) | | |  | | **701.5** | 1469.4 | 1859.5 | 16205.8 | 633.0 | 16739.7 |
| 300 | [At1g65670](http://atted.jp/cgi-bin/coex_Ath.cgi?gene=At1g65670&sort=all) | | | CYP702A1 | | **705.5** | 1043.4 | 14549.6 | 4224.2 | 2088.5 | 12115.0 |
|  | | | | | | | | | | | |
| **300 coexpressed gene with main gene At1g18250** | | | | | | | | | | | |
|  | **locus** | | **Short description** | | | **MR**  **(all)** | **MR**  **(tissue)** | **MR**  **(abiotic)** | **MR**  **(biotic)** | **MR**  **(hormone)** | **MR**  **(light)** |
| 1 | [At3g53190](http://atted.jp/cgi-bin/coex_Ath.cgi?gene=At3g53190&sort=all) | | lyase | | | **1.4** | 1.0 | 3.3 | 710.4 | 13.4 | 521.0 |
| 2 | [At4g38660](http://atted.jp/cgi-bin/coex_Ath.cgi?gene=At4g38660&sort=all) | | thaumatin | | | **3.9** | 3.5 | 44.5 | 405.6 | 12.0 | 2009.8 |
| 3 | [At1g28290](http://atted.jp/cgi-bin/coex_Ath.cgi?gene=At1g28290&sort=all) | | AGP31 | | | **5.0** | 27.6 | 87.5 | 31.7 | 13.0 | 19239.5 |
| 4 | [At4g34160](http://atted.jp/cgi-bin/coex_Ath.cgi?gene=At4g34160&sort=all) | | CYCD3;1 | | | **5.7** | 7.8 | 32.6 | 4.6 | 1.4 | 23.0 |
| 5 | [At5g44560](http://atted.jp/cgi-bin/coex_Ath.cgi?gene=At5g44560&sort=all) | | VPS2.2 | | | **6.2** | 14.9 | 16.8 | 371.0 | 275.9 | 8266.7 |
| 6 | [At2g43800](http://atted.jp/cgi-bin/coex_Ath.cgi?gene=At2g43800&sort=all) | | FH2 | | | **7.0** | 6.9 | 116.8 | 3850.2 | 77.6 | 3050.7 |
| 7 | [At5g62710](http://atted.jp/cgi-bin/coex_Ath.cgi?gene=At5g62710&sort=all) | | kinase | | | **7.6** | 48.1 | 14.3 | 71.9 | 56.7 | 10012.6 |
| 8 | [At4g15830](http://atted.jp/cgi-bin/coex_Ath.cgi?gene=At4g15830&sort=all) | | binding | | | **8.0** | 11.3 | 8.7 | 46.0 | 50.2 | 17076.8 |
| 9 | [At2g21050](http://atted.jp/cgi-bin/coex_Ath.cgi?gene=At2g21050&sort=all) | | LAX2 | | | **8.4** | 31.9 | 27.3 | 1139.9 | 441.4 | 11037.4 |
| 10 | [At3g23890](http://atted.jp/cgi-bin/coex_Ath.cgi?gene=At3g23890&sort=all) | | TOPII | | | **9.2** | 13.1 | 20.1 | 67.8 | 102.0 | 15219.0 |
| 11 | [At5g67200](http://atted.jp/cgi-bin/coex_Ath.cgi?gene=At5g67200&sort=all) | | kinase | | | **9.7** | 132.5 | 30.1 | 6423.5 | 3839.0 | 2924.1 |
| 12 | [At1g70710](http://atted.jp/cgi-bin/coex_Ath.cgi?gene=At1g70710&sort=all) | | GH9B1 | | | **10.1** | 49.6 | 26.6 | 62.5 | 224.1 | 17776.8 |
| 13 | [At4g26760](http://atted.jp/cgi-bin/coex_Ath.cgi?gene=At4g26760&sort=all) | | MAP65-2 | | | **10.1** | 26.4 | 24.8 | 2995.1 | 20.9 | 1345.5 |
| 14 | [At4g37750](http://atted.jp/cgi-bin/coex_Ath.cgi?gene=At4g37750&sort=all) | | ANT | | | **10.5** | 28.0 | 93.0 | 53.0 | 268.7 | 876.3 |
| 15 | [At1g02730](http://atted.jp/cgi-bin/coex_Ath.cgi?gene=At1g02730&sort=all) | | CSLD5 | | | **10.6** | 3.5 | 12.3 | 6.9 | 22.1 | 17859.2 |
| 16 | [At3g51720](http://atted.jp/cgi-bin/coex_Ath.cgi?gene=At3g51720&sort=all) | |  | | | **11.2** | 3.5 | 65.4 | 83.0 | 582.4 | 12225.0 |
| 17 | [At3g22790](http://atted.jp/cgi-bin/coex_Ath.cgi?gene=At3g22790&sort=all) | | kinase interacting | | | **12.3** | 20.7 | 34.4 | 1593.5 | 548.5 | 8344.7 |
| 18 | [At2g25270](http://atted.jp/cgi-bin/coex_Ath.cgi?gene=At2g25270&sort=all) | |  | | | **12.7** | 2.2 | 48.1 | 4977.4 | 1230.5 | 12446.5 |
| 19 | [At5g01890](http://atted.jp/cgi-bin/coex_Ath.cgi?gene=At5g01890&sort=all) | | kinase | | | **12.7** | 26.8 | 242.7 | 1120.4 | 85.0 | 3528.8 |
| 20 | [At1g21880](http://atted.jp/cgi-bin/coex_Ath.cgi?gene=At1g21880&sort=all) | | LYM1 | | | **13.4** | 13.3 | 7.4 | 505.4 | 240.2 | 948.4 |
| 21 | [At2g25060](http://atted.jp/cgi-bin/coex_Ath.cgi?gene=At2g25060&sort=all) | | plastocyanin-like | | | **13.4** | 17.4 | 4.2 | 353.5 | 91.4 | 7869.8 |
| 22 | [At5g51560](http://atted.jp/cgi-bin/coex_Ath.cgi?gene=At5g51560&sort=all) | | kinase | | | **13.6** | 31.1 | 87.0 | 1153.3 | 66.7 | 1563.0 |
| 23 | [At3g62060](http://atted.jp/cgi-bin/coex_Ath.cgi?gene=At3g62060&sort=all) | | pectinacetylesterase | | | **13.8** | 39.1 | 163.2 | 306.8 | 121.0 | 5493.7 |
| 24 | [At4g36180](http://atted.jp/cgi-bin/coex_Ath.cgi?gene=At4g36180&sort=all) | | LRR | | | **14.0** | 39.2 | 231.4 | 778.8 | 84.4 | 275.8 |
| 25 | [At3g02640](http://atted.jp/cgi-bin/coex_Ath.cgi?gene=At3g02640&sort=all) | |  | | | **14.2** | 2.8 | 19.2 | 14.8 | 2.5 | 8472.0 |
| 26 | [At1g17560](http://atted.jp/cgi-bin/coex_Ath.cgi?gene=At1g17560&sort=all) | | HLL | | | **14.5** | 71.3 | 74.9 | 2403.1 | 2348.3 | 3508.4 |
| 27 | [At4g23800](http://atted.jp/cgi-bin/coex_Ath.cgi?gene=At4g23800&sort=all) | | HMG1/2 | | | **16.5** | 4.9 | 45.7 | 18.6 | 109.1 | 7708.3 |
| 28 | [At3g07320](http://atted.jp/cgi-bin/coex_Ath.cgi?gene=At3g07320&sort=all) | | hydrolase | | | **16.7** | 52.8 | 1872.2 | 1882.2 | 331.1 | 6456.5 |
| 29 | [At5g13520](http://atted.jp/cgi-bin/coex_Ath.cgi?gene=At5g13520&sort=all) | | peptidase M1 | | | **18.6** | 62.7 | 187.8 | 117.2 | 196.3 | 13303.9 |
| 30 | [At1g48610](http://atted.jp/cgi-bin/coex_Ath.cgi?gene=At1g48610&sort=all) | | AT hook motif | | | **19.8** | 98.5 | 292.7 | 1992.0 | 495.5 | 1753.8 |
| 31 | [At3g15680](http://atted.jp/cgi-bin/coex_Ath.cgi?gene=At3g15680&sort=all) | | Ran-binding | | | **20.1** | 7.1 | 149.4 | 72.4 | 5.9 | 811.4 |
| 32 | [At1g04520](http://atted.jp/cgi-bin/coex_Ath.cgi?gene=At1g04520&sort=all) | | PDLP2 | | | **20.3** | 51.0 | 292.7 | 167.5 | 761.3 | 9753.3 |
| 33 | [At1g44110](http://atted.jp/cgi-bin/coex_Ath.cgi?gene=At1g44110&sort=all) | | CYCA1;1 | | | **21.2** | 52.5 | 2.8 | 433.4 | 100.7 | 8916.0 |
| 34 | [At1g11130](http://atted.jp/cgi-bin/coex_Ath.cgi?gene=At1g11130&sort=all) | | SUB | | | **21.6** | 51.9 | 227.1 | 854.8 | 183.7 | 682.8 |
| 35 | 260118_s_at | |  | | | **22.1** | 117.8 | 39.4 | 826.4 | 3721.6 | 4829.5 |
| 36 | [At2g44830](http://atted.jp/cgi-bin/coex_Ath.cgi?gene=At2g44830&sort=all) | | kinase | | | **22.4** | 21.4 | 65.4 | 660.0 | 3243.9 | 4891.0 |
| 37 | [At3g19590](http://atted.jp/cgi-bin/coex_Ath.cgi?gene=At3g19590&sort=all) | | WD-40 repeat | | | **22.6** | 27.0 | 36.0 | 1137.3 | 754.5 | 9887.8 |
| 38 | [At1g76540](http://atted.jp/cgi-bin/coex_Ath.cgi?gene=At1g76540&sort=all) | | CDKB2;1 | | | **24.2** | 11.8 | 39.6 | 6.9 | 462.3 | 16204.6 |
| 39 | [At4g02800](http://atted.jp/cgi-bin/coex_Ath.cgi?gene=At4g02800&sort=all) | |  | | | **24.7** | 16.9 | 19.9 | 2057.0 | 2312.1 | 12223.4 |
| 40 | [At1g18370](http://atted.jp/cgi-bin/coex_Ath.cgi?gene=At1g18370&sort=all) | | HIK | | | **25.5** | 27.9 | 31.4 | 226.6 | 74.8 | 12492.0 |
| 41 | [At4g20430](http://atted.jp/cgi-bin/coex_Ath.cgi?gene=At4g20430&sort=all) | | subtilase | | | **26.0** | 24.3 | 471.9 | 792.1 | 1700.8 | 528.8 |
| 42 | [At1g69770](http://atted.jp/cgi-bin/coex_Ath.cgi?gene=At1g69770&sort=all) | | CMT3 | | | **26.7** | 62.5 | 81.6 | 10210.8 | 1559.0 | 5120.7 |
| 43 | [At3g20260](http://atted.jp/cgi-bin/coex_Ath.cgi?gene=At3g20260&sort=all) | | ribosome | | | **26.7** | 78.7 | 86.1 | 1627.3 | 111.5 | 12953.6 |
| 44 | [At1g07370](http://atted.jp/cgi-bin/coex_Ath.cgi?gene=At1g07370&sort=all) | | PCNA1 | | | **26.8** | 17.8 | 117.3 | 1107.1 | 767.3 | 523.2 |
| 45 | [At5g25090](http://atted.jp/cgi-bin/coex_Ath.cgi?gene=At5g25090&sort=all) | | plastocyanin-like | | | **26.8** | 38.8 | 22.8 | 1058.7 | 215.7 | 7106.6 |
| 46 | [At4g15790](http://atted.jp/cgi-bin/coex_Ath.cgi?gene=At4g15790&sort=all) | |  | | | **27.2** | 282.6 | 295.2 | 3011.1 | 690.2 | 2903.8 |
| 47 | [At1g08560](http://atted.jp/cgi-bin/coex_Ath.cgi?gene=At1g08560&sort=all) | | SYP111 | | | **27.5** | 26.1 | 40.3 | 78.0 | 276.6 | 19841.7 |
| 48 | [At1g63470](http://atted.jp/cgi-bin/coex_Ath.cgi?gene=At1g63470&sort=all) | | DNA-binding | | | **28.4** | 68.9 | 73.9 | 4810.9 | 1731.0 | 5018.5 |
| 49 | [At2g36200](http://atted.jp/cgi-bin/coex_Ath.cgi?gene=At2g36200&sort=all) | | kinesin motor | | | **28.8** | 177.4 | 4.2 | 406.4 | 491.5 | 3758.7 |
| 50 | [At1g78430](http://atted.jp/cgi-bin/coex_Ath.cgi?gene=At1g78430&sort=all) | | RIP2 | | | **29.3** | 64.5 | 97.7 | 4895.8 | 1881.4 | 9464.9 |
| 51 | [At2g01630](http://atted.jp/cgi-bin/coex_Ath.cgi?gene=At2g01630&sort=all) | | glucanase | | | **30.6** | 25.0 | 534.0 | 426.8 | 11.8 | 2431.8 |
| 52 | 249659_s_at | |  | | | **30.7** | 7.5 | 30.7 | 21.7 | 194.5 | 6224.5 |
| 53 | [At1g30600](http://atted.jp/cgi-bin/coex_Ath.cgi?gene=At1g30600&sort=all) | | subtilase | | | **30.8** | 266.1 | 92.9 | 6.6 | 1636.6 | 5289.2 |
| 54 | [At3g58100](http://atted.jp/cgi-bin/coex_Ath.cgi?gene=At3g58100&sort=all) | | PDCB5 | | | **31.1** | 32.3 | 546.4 | 2923.6 | 1330.9 | 16871.3 |
| 55 | [At5g06050](http://atted.jp/cgi-bin/coex_Ath.cgi?gene=At5g06050&sort=all) | | dehydration-responsive | | | **31.3** | 50.8 | 240.6 | 1820.6 | 219.1 | 10448.0 |
| 56 | [At1g29980](http://atted.jp/cgi-bin/coex_Ath.cgi?gene=At1g29980&sort=all) | |  | | | **31.8** | 274.7 | 5.8 | 3732.2 | 2670.3 | 670.5 |
| 57 | [At3g56810](http://atted.jp/cgi-bin/coex_Ath.cgi?gene=At3g56810&sort=all) | |  | | | **32.0** | 55.3 | 83.7 | 170.8 | 255.3 | 11004.1 |
| 58 | [At3g29280](http://atted.jp/cgi-bin/coex_Ath.cgi?gene=At3g29280&sort=all) | |  | | | **32.1** | 196.5 | 31.0 | 257.1 | 925.3 | 4693.0 |
| 59 | [At4g29360](http://atted.jp/cgi-bin/coex_Ath.cgi?gene=At4g29360&sort=all) | | hydrolase | | | **33.2** | 43.9 | 1472.8 | 1244.0 | 49.0 | 5339.8 |
| 60 | [At2g38810](http://atted.jp/cgi-bin/coex_Ath.cgi?gene=At2g38810&sort=all) | | HTA8 | | | **33.4** | 100.3 | 16.2 | 9402.0 | 518.0 | 17963.4 |
| 61 | [At1g53140](http://atted.jp/cgi-bin/coex_Ath.cgi?gene=At1g53140&sort=all) | | DRP5A | | | **33.5** | 76.7 | 16.4 | 2852.9 | 12939.9 | 18848.7 |
| 62 | [At3g25980](http://atted.jp/cgi-bin/coex_Ath.cgi?gene=At3g25980&sort=all) | | MAD2 | | | **33.9** | 35.2 | 62.3 | 55.1 | 88.6 | 17685.0 |
| 63 | [At1g32930](http://atted.jp/cgi-bin/coex_Ath.cgi?gene=At1g32930&sort=all) | | transferase | | | **34.1** | 140.5 | 245.8 | 12698.2 | 10563.5 | 11126.6 |
| 64 | [At2g29550](http://atted.jp/cgi-bin/coex_Ath.cgi?gene=At2g29550&sort=all) | | TUB7 | | | **34.3** | 47.5 | 13.3 | 1453.4 | 144.0 | 20510.6 |
| 65 | [At5g16250](http://atted.jp/cgi-bin/coex_Ath.cgi?gene=At5g16250&sort=all) | |  | | | **34.9** | 11.8 | 77.3 | 26.9 | 8.4 | 13831.2 |
| 66 | [At1g10780](http://atted.jp/cgi-bin/coex_Ath.cgi?gene=At1g10780&sort=all) | | F-box | | | **35.5** | 196.8 | 56.2 | 200.1 | 15.2 | 17627.1 |
| 67 | [At3g13674](http://atted.jp/cgi-bin/coex_Ath.cgi?gene=At3g13674&sort=all) | |  | | | **36.2** | 158.0 | 395.9 | 1061.3 | 724.3 | 1382.5 |
| 68 | [At3g54560](http://atted.jp/cgi-bin/coex_Ath.cgi?gene=At3g54560&sort=all) | | HTA11 | | | **36.2** | 17.4 | 29.4 | 6836.2 | 135.5 | 19126.2 |
| 69 | [At1g59540](http://atted.jp/cgi-bin/coex_Ath.cgi?gene=At1g59540&sort=all) | | ZCF125 | | | **36.8** | 88.4 | 44.7 | 3553.8 | 1773.2 | 10467.6 |
| 70 | [At4g33400](http://atted.jp/cgi-bin/coex_Ath.cgi?gene=At4g33400&sort=all) | | dem-related | | | **37.4** | 114.0 | 56.8 | 6768.1 | 13.0 | 11683.9 |
| 71 | [At5g46700](http://atted.jp/cgi-bin/coex_Ath.cgi?gene=At5g46700&sort=all) | | TRN2 | | | **37.6** | 55.9 | 40.5 | 2104.5 | 55.6 | 5803.4 |
| 72 | [At2g36570](http://atted.jp/cgi-bin/coex_Ath.cgi?gene=At2g36570&sort=all) | | kinase | | | **37.7** | 158.4 | 133.8 | 22.8 | 7.1 | 4637.0 |
| 73 | 253340_s_at | |  | | | **37.8** | 26.0 | 29.8 | 162.8 | 93.0 | 17401.5 |
| 74 | [At2g07690](http://atted.jp/cgi-bin/coex_Ath.cgi?gene=At2g07690&sort=all) | | MCM5 | | | **38.4** | 74.9 | 121.0 | 3683.1 | 2707.6 | 1316.5 |
| 75 | [At2g33620](http://atted.jp/cgi-bin/coex_Ath.cgi?gene=At2g33620&sort=all) | | AHP1 | | | **38.6** | 120.6 | 89.7 | 12256.7 | 489.2 | 2426.8 |
| 76 | [At1g72250](http://atted.jp/cgi-bin/coex_Ath.cgi?gene=At1g72250&sort=all) | | kinesin motor | | | **38.7** | 91.5 | 24.5 | 412.4 | 632.1 | 1695.2 |
| 77 | [At5g55520](http://atted.jp/cgi-bin/coex_Ath.cgi?gene=At5g55520&sort=all) | |  | | | **38.9** | 74.5 | 38.7 | 2263.8 | 212.4 | 9242.7 |
| 78 | [At3g17840](http://atted.jp/cgi-bin/coex_Ath.cgi?gene=At3g17840&sort=all) | | RLK902 | | | **39.0** | 50.8 | 204.3 | 1751.0 | 32.2 | 2126.2 |
| 79 | [At3g24660](http://atted.jp/cgi-bin/coex_Ath.cgi?gene=At3g24660&sort=all) | | TMKL1 | | | **39.5** | 152.4 | 79.0 | 1547.4 | 95.7 | 15921.7 |
| 80 | [At5g17620](http://atted.jp/cgi-bin/coex_Ath.cgi?gene=At5g17620&sort=all) | |  | | | **39.8** | 127.6 | 184.8 | 422.5 | 6524.6 | 14468.7 |
| 81 | [At5g48360](http://atted.jp/cgi-bin/coex_Ath.cgi?gene=At5g48360&sort=all) | | FH2 | | | **40.0** | 332.8 | 6.8 | 683.7 | 1816.9 | 1203.9 |
| 82 | [At2g27970](http://atted.jp/cgi-bin/coex_Ath.cgi?gene=At2g27970&sort=all) | | CKS2 | | | **41.2** | 246.9 | 2.8 | 252.4 | 83.1 | 14470.6 |
| 83 | [At5g13960](http://atted.jp/cgi-bin/coex_Ath.cgi?gene=At5g13960&sort=all) | | SUVH4 | | | **42.1** | 42.0 | 95.0 | 8252.1 | 7540.6 | 11782.5 |
| 84 | [At2g16440](http://atted.jp/cgi-bin/coex_Ath.cgi?gene=At2g16440&sort=all) | | MCM4 | | | **42.4** | 128.4 | 91.2 | 1231.8 | 481.4 | 1724.1 |
| 85 | [At4g31840](http://atted.jp/cgi-bin/coex_Ath.cgi?gene=At4g31840&sort=all) | | plastocyanin-like | | | **42.7** | 9.9 | 21.0 | 8.1 | 7.0 | 1289.7 |
| 86 | [At1g44900](http://atted.jp/cgi-bin/coex_Ath.cgi?gene=At1g44900&sort=all) | | MCM2 | | | **42.8** | 58.0 | 114.9 | 3535.5 | 1361.5 | 2202.0 |
| 87 | [At2g28790](http://atted.jp/cgi-bin/coex_Ath.cgi?gene=At2g28790&sort=all) | | osmotin-like | | | **44.1** | 357.7 | 8.2 | 199.2 | 147.8 | 186.9 |
| 88 | [At3g17680](http://atted.jp/cgi-bin/coex_Ath.cgi?gene=At3g17680&sort=all) | |  | | | **44.2** | 66.1 | 181.2 | 128.4 | 3611.8 | 10892.7 |
| 89 | [At1g49580](http://atted.jp/cgi-bin/coex_Ath.cgi?gene=At1g49580&sort=all) | | CDPK | | | **44.5** | 356.9 | 367.0 | 844.6 | 911.9 | 13159.3 |
| 90 | [At4g05520](http://atted.jp/cgi-bin/coex_Ath.cgi?gene=At4g05520&sort=all) | | EHD2 | | | **44.9** | 51.1 | 161.8 | 767.6 | 346.1 | 12381.1 |
| 91 | [At3g22880](http://atted.jp/cgi-bin/coex_Ath.cgi?gene=At3g22880&sort=all) | | DMC1 | | | **45.5** | 84.0 | 33.0 | 1363.3 | 7943.6 | 12302.3 |
| 92 | [At1g26100](http://atted.jp/cgi-bin/coex_Ath.cgi?gene=At1g26100&sort=all) | | B561 | | | **46.3** | 48.1 | 273.7 | 2868.1 | 925.9 | 1388.4 |
| 93 | [At1g05210](http://atted.jp/cgi-bin/coex_Ath.cgi?gene=At1g05210&sort=all) | |  | | | **46.5** | 100.8 | 658.9 | 1663.5 | 987.3 | 9365.5 |
| 94 | [At5g01370](http://atted.jp/cgi-bin/coex_Ath.cgi?gene=At5g01370&sort=all) | | ACI1 | | | **47.0** | 27.4 | 99.1 | 57.2 | 6767.3 | 10765.9 |
| 95 | [At4g05190](http://atted.jp/cgi-bin/coex_Ath.cgi?gene=At4g05190&sort=all) | | ATK5 | | | **47.0** | 48.7 | 31.0 | 153.2 | 285.1 | 15394.7 |
| 96 | [At5g56580](http://atted.jp/cgi-bin/coex_Ath.cgi?gene=At5g56580&sort=all) | | MKK6 | | | **47.3** | 159.8 | 253.1 | 2161.8 | 2275.3 | 4104.8 |
| 97 | [At1g69700](http://atted.jp/cgi-bin/coex_Ath.cgi?gene=At1g69700&sort=all) | | HVA22C | | | **47.5** | 99.8 | 131.3 | 973.1 | 82.7 | 1896.4 |
| 98 | [At1g76310](http://atted.jp/cgi-bin/coex_Ath.cgi?gene=At1g76310&sort=all) | | CYCB2;4 | | | **48.5** | 66.1 | 72.8 | 910.4 | 2660.9 | 12818.4 |
| 99 | [At5g23400](http://atted.jp/cgi-bin/coex_Ath.cgi?gene=At5g23400&sort=all) | | LRR | | | **48.7** | 164.0 | 65.7 | 266.8 | 211.5 | 17822.2 |
| 100 | [At4g03100](http://atted.jp/cgi-bin/coex_Ath.cgi?gene=At4g03100&sort=all) | | rac GTPase activating | | | **48.8** | 172.9 | 4.6 | 122.3 | 876.9 | 11962.5 |
| 101 | [At5g67100](http://atted.jp/cgi-bin/coex_Ath.cgi?gene=At5g67100&sort=all) | | ICU2 | | | **49.0** | 55.5 | 79.2 | 2754.8 | 476.9 | 3740.3 |
| 102 | [At1g73620](http://atted.jp/cgi-bin/coex_Ath.cgi?gene=At1g73620&sort=all) | | pathogenesis | | | **49.6** | 773.0 | 13.6 | 1300.7 | 218.1 | 11489.4 |
| 103 | [At1g16920](http://atted.jp/cgi-bin/coex_Ath.cgi?gene=At1g16920&sort=all) | | RABA1B | | | **50.0** | 105.5 | 281.5 | 674.7 | 299.6 | 15242.2 |
| 104 | [At3g51280](http://atted.jp/cgi-bin/coex_Ath.cgi?gene=At3g51280&sort=all) | | MS5 | | | **50.5** | 49.3 | 60.0 | 1597.6 | 18.4 | 18445.7 |
| 105 | [At5g46280](http://atted.jp/cgi-bin/coex_Ath.cgi?gene=At5g46280&sort=all) | | MCM3 | | | **50.7** | 91.8 | 58.8 | 3087.4 | 766.6 | 4511.9 |
| 106 | [At3g02120](http://atted.jp/cgi-bin/coex_Ath.cgi?gene=At3g02120&sort=all) | | glycoprotein | | | **50.7** | 49.8 | 33.5 | 59.8 | 69.4 | 570.9 |
| 107 | [At2g17560](http://atted.jp/cgi-bin/coex_Ath.cgi?gene=At2g17560&sort=all) | | HMGB4 | | | **51.1** | 246.5 | 282.1 | 12396.8 | 1448.2 | 14685.8 |
| 108 | [At3g22780](http://atted.jp/cgi-bin/coex_Ath.cgi?gene=At3g22780&sort=all) | | TSO1 | | | **51.2** | 103.4 | 41.7 | 1929.5 | 2926.1 | 1262.6 |
| 109 | [At5g13840](http://atted.jp/cgi-bin/coex_Ath.cgi?gene=At5g13840&sort=all) | | FZR3 | | | **52.1** | 19.0 | 54.1 | 102.7 | 1292.0 | 12815.5 |
| 110 | [At5g48310](http://atted.jp/cgi-bin/coex_Ath.cgi?gene=At5g48310&sort=all) | |  | | | **52.4** | 55.2 | 67.9 | 1907.5 | 5738.3 | 16414.4 |
| 111 | [At2g16780](http://atted.jp/cgi-bin/coex_Ath.cgi?gene=At2g16780&sort=all) | | MSI2 | | | **52.4** | 38.2 | 196.5 | 8321.2 | 125.9 | 9232.3 |
| 112 | [At1g50240](http://atted.jp/cgi-bin/coex_Ath.cgi?gene=At1g50240&sort=all) | | FU | | | **52.6** | 57.2 | 53.6 | 474.8 | 5164.2 | 10738.3 |
| 113 | [At4g02060](http://atted.jp/cgi-bin/coex_Ath.cgi?gene=At4g02060&sort=all) | | PRL | | | **52.8** | 216.7 | 28.6 | 964.9 | 1165.5 | 3196.6 |
| 114 | [At3g20150](http://atted.jp/cgi-bin/coex_Ath.cgi?gene=At3g20150&sort=all) | | kinesin motor | | | **52.9** | 83.4 | 34.0 | 2173.1 | 2467.5 | 19678.2 |
| 115 | [At3g56370](http://atted.jp/cgi-bin/coex_Ath.cgi?gene=At3g56370&sort=all) | | kinase | | | **54.0** | 124.7 | 53.0 | 1939.9 | 838.3 | 4612.9 |
| 116 | [At1g67320](http://atted.jp/cgi-bin/coex_Ath.cgi?gene=At1g67320&sort=all) | | DNA primase, | | | **54.8** | 51.9 | 135.9 | 644.2 | 6668.1 | 831.7 |
| 117 | [At5g01910](http://atted.jp/cgi-bin/coex_Ath.cgi?gene=At5g01910&sort=all) | |  | | | **55.3** | 78.8 | 30.7 | 1585.7 | 2195.3 | 11305.4 |
| 118 | [At3g06740](http://atted.jp/cgi-bin/coex_Ath.cgi?gene=At3g06740&sort=all) | | GATA15 | | | **55.6** | 17.6 | 624.2 | 3872.5 | 1505.5 | 19492.9 |
| 119 | [At1g74690](http://atted.jp/cgi-bin/coex_Ath.cgi?gene=At1g74690&sort=all) | | IQD31 | | | **56.1** | 191.1 | 46.5 | 973.0 | 85.7 | 2643.2 |
| 120 | [At5g62550](http://atted.jp/cgi-bin/coex_Ath.cgi?gene=At5g62550&sort=all) | |  | | | **56.3** | 98.6 | 10.0 | 68.2 | 531.0 | 14194.6 |
| 121 | [At1g21090](http://atted.jp/cgi-bin/coex_Ath.cgi?gene=At1g21090&sort=all) | | glycoprotein | | | **56.6** | 77.8 | 37.1 | 5861.4 | 1157.3 | 14730.4 |
| 122 | [At3g07540](http://atted.jp/cgi-bin/coex_Ath.cgi?gene=At3g07540&sort=all) | | FH2 | | | **57.1** | 28.7 | 112.3 | 1058.6 | 8818.4 | 5172.4 |
| 123 | [At5g66560](http://atted.jp/cgi-bin/coex_Ath.cgi?gene=At5g66560&sort=all) | | NPH3 | | | **57.8** | 66.0 | 476.5 | 1665.0 | 5204.9 | 4253.7 |
| 124 | [At5g50740](http://atted.jp/cgi-bin/coex_Ath.cgi?gene=At5g50740&sort=all) | | metal ion binding | | | **58.1** | 585.3 | 71.1 | 45.2 | 117.2 | 10330.4 |
| 125 | [At5g62410](http://atted.jp/cgi-bin/coex_Ath.cgi?gene=At5g62410&sort=all) | | SMC2 | | | **58.2** | 142.5 | 15.9 | 1676.6 | 2615.5 | 1405.2 |
| 126 | 251331_s_at | |  | | | **58.9** | 60.1 | 64.1 | 3658.9 | 1178.8 | 12358.4 |
| 127 | [At3g13560](http://atted.jp/cgi-bin/coex_Ath.cgi?gene=At3g13560&sort=all) | | hydrolase | | | **59.9** | 97.6 | 46.0 | 4695.1 | 92.9 | 4425.0 |
| 128 | [At1g70470](http://atted.jp/cgi-bin/coex_Ath.cgi?gene=At1g70470&sort=all) | |  | | | **60.2** | 101.9 | 219.8 | 85.7 | 972.0 | 2585.8 |
| 129 | [At4g17610](http://atted.jp/cgi-bin/coex_Ath.cgi?gene=At4g17610&sort=all) | | tRNA | | | **60.7** | 363.7 | 594.0 | 2985.5 | 2056.1 | 10352.8 |
| 130 | [At4g31360](http://atted.jp/cgi-bin/coex_Ath.cgi?gene=At4g31360&sort=all) | | selenium binding | | | **60.7** | 357.2 | 182.4 | 1284.4 | 3469.9 | 6768.1 |
| 131 | [At5g17160](http://atted.jp/cgi-bin/coex_Ath.cgi?gene=At5g17160&sort=all) | |  | | | **61.0** | 138.9 | 32.6 | 125.9 | 176.7 | 6554.6 |
| 132 | [At5g45670](http://atted.jp/cgi-bin/coex_Ath.cgi?gene=At5g45670&sort=all) | | hydrolase | | | **61.9** | 383.4 | 15.5 | 402.2 | 2073.8 | 7933.4 |
| 133 | [At3g06030](http://atted.jp/cgi-bin/coex_Ath.cgi?gene=At3g06030&sort=all) | | ANP3 | | | **62.8** | 97.5 | 69.6 | 1132.4 | 52.0 | 1997.5 |
| 134 | [At4g11080](http://atted.jp/cgi-bin/coex_Ath.cgi?gene=At4g11080&sort=all) | | HMG1/2 | | | **63.4** | 13.4 | 111.2 | 9301.8 | 5010.8 | 7899.0 |
| 135 | [At1g77630](http://atted.jp/cgi-bin/coex_Ath.cgi?gene=At1g77630&sort=all) | | LysM | | | **63.7** | 74.2 | 393.1 | 1211.5 | 95.2 | 2688.7 |
| 136 | [At5g67070](http://atted.jp/cgi-bin/coex_Ath.cgi?gene=At5g67070&sort=all) | | RALFL34 | | | **64.2** | 212.1 | 34.1 | 1234.9 | 1676.0 | 612.8 |
| 137 | [At1g64450](http://atted.jp/cgi-bin/coex_Ath.cgi?gene=At1g64450&sort=all) | | proline-rich | | | **64.3** | 445.7 | 133.6 | 3856.2 | 879.8 | 3716.6 |
| 138 | [At2g26760](http://atted.jp/cgi-bin/coex_Ath.cgi?gene=At2g26760&sort=all) | | CYCB1;4 | | | **64.6** | 55.5 | 98.9 | 233.1 | 226.9 | 17305.7 |
| 139 | [At2g22610](http://atted.jp/cgi-bin/coex_Ath.cgi?gene=At2g22610&sort=all) | | kinesin motor | | | **64.9** | 186.2 | 54.0 | 358.1 | 442.0 | 5905.0 |
| 140 | [At3g50070](http://atted.jp/cgi-bin/coex_Ath.cgi?gene=At3g50070&sort=all) | | CYCD3;3 | | | **65.0** | 139.8 | 46.0 | 5552.4 | 3122.7 | 5731.1 |
| 141 | [246837_at](http://atted.jp/cgi-bin/coex_Ath.cgi?gene=246837_at&sort=all) | |  | | | **65.9** | 591.7 | 16.2 | 189.0 | 4433.1 | 13734.7 |
| 142 | [At1g28110](http://atted.jp/cgi-bin/coex_Ath.cgi?gene=At1g28110&sort=all) | | SCPL45 | | | **66.3** | 123.2 | 38.3 | 5908.4 | 1108.2 | 18736.7 |
| 143 | [At5g62390](http://atted.jp/cgi-bin/coex_Ath.cgi?gene=At5g62390&sort=all) | | BAG7 | | | **66.3** | 184.2 | 789.3 | 15579.8 | 3037.9 | 8129.1 |
| 144 | [At2g37080](http://atted.jp/cgi-bin/coex_Ath.cgi?gene=At2g37080&sort=all) | | RIP3 | | | **66.6** | 451.9 | 26.5 | 4403.6 | 47.8 | 887.1 |
| 145 | [At5g61130](http://atted.jp/cgi-bin/coex_Ath.cgi?gene=At5g61130&sort=all) | | PDCB1 | | | **67.2** | 53.4 | 502.0 | 1271.1 | 543.0 | 10653.3 |
| 146 | [256406_at](http://atted.jp/cgi-bin/coex_Ath.cgi?gene=256406_at&sort=all) | |  | | | **67.4** | 142.3 | 324.2 | 1882.8 | 7739.5 | 641.1 |
| 147 | [At4g21820](http://atted.jp/cgi-bin/coex_Ath.cgi?gene=At4g21820&sort=all) | | binding | | | **67.5** | 241.7 | 65.3 | 487.8 | 1791.1 | 13148.7 |
| 148 | [At5g14610](http://atted.jp/cgi-bin/coex_Ath.cgi?gene=At5g14610&sort=all) | | helicase | | | **67.7** | 298.7 | 355.9 | 323.9 | 1054.0 | 14584.1 |
| 149 | [At2g05790](http://atted.jp/cgi-bin/coex_Ath.cgi?gene=At2g05790&sort=all) | | hydrolase | | | **67.9** | 193.0 | 67.3 | 846.5 | 85.2 | 12584.1 |
| 150 | [At3g15550](http://atted.jp/cgi-bin/coex_Ath.cgi?gene=At3g15550&sort=all) | |  | | | **68.4** | 103.8 | 71.0 | 1286.3 | 11037.4 | 11320.7 |
| 151 | [At1g09200](http://atted.jp/cgi-bin/coex_Ath.cgi?gene=At1g09200&sort=all) | | histone H3 | | | **69.2** | 55.5 | 67.2 | 3238.6 | 2427.3 | 3221.5 |
| 152 | [At3g07410](http://atted.jp/cgi-bin/coex_Ath.cgi?gene=At3g07410&sort=all) | | RABA5b | | | **69.7** | 259.2 | 325.4 | 13425.4 | 1393.6 | 11717.6 |
| 153 | [At1g03780](http://atted.jp/cgi-bin/coex_Ath.cgi?gene=At1g03780&sort=all) | | TPX2 | | | **70.8** | 151.5 | 67.0 | 1369.6 | 7935.5 | 9106.5 |
| 154 | [At1g01300](http://atted.jp/cgi-bin/coex_Ath.cgi?gene=At1g01300&sort=all) | | protease | | | **70.9** | 407.3 | 479.5 | 3941.4 | 1482.7 | 10023.7 |
| 155 | [At1g56210](http://atted.jp/cgi-bin/coex_Ath.cgi?gene=At1g56210&sort=all) | | CCH | | | **71.1** | 242.8 | 299.1 | 992.5 | 445.1 | 5194.2 |
| 156 | [At5g67270](http://atted.jp/cgi-bin/coex_Ath.cgi?gene=At5g67270&sort=all) | | EB1C | | | **71.4** | 201.2 | 113.7 | 289.4 | 534.9 | 13050.8 |
| 157 | [At4g39630](http://atted.jp/cgi-bin/coex_Ath.cgi?gene=At4g39630&sort=all) | |  | | | **71.4** | 170.6 | 6.2 | 2421.2 | 3436.1 | 14914.9 |
| 158 | [At5g59870](http://atted.jp/cgi-bin/coex_Ath.cgi?gene=At5g59870&sort=all) | | HTA6 | | | **71.5** | 27.5 | 51.8 | 2233.6 | 4302.6 | 2624.0 |
| 159 | [At1g50490](http://atted.jp/cgi-bin/coex_Ath.cgi?gene=At1g50490&sort=all) | | UBC20 | | | **71.9** | 235.6 | 44.2 | 685.0 | 26.5 | 4801.8 |
| 160 | 266401_s_at | |  | | | **72.2** | 170.0 | 55.9 | 3088.4 | 4048.4 | 19721.7 |
| 161 | [At4g37110](http://atted.jp/cgi-bin/coex_Ath.cgi?gene=At4g37110&sort=all) | | protein binding | | | **72.2** | 195.1 | 198.9 | 1242.0 | 2240.4 | 13698.6 |
| 162 | [At1g47670](http://atted.jp/cgi-bin/coex_Ath.cgi?gene=At1g47670&sort=all) | | transporter | | | **72.3** | 638.8 | 45.4 | 848.8 | 8.1 | 12850.9 |
| 163 | [At1g53070](http://atted.jp/cgi-bin/coex_Ath.cgi?gene=At1g53070&sort=all) | | legume lectin | | | **72.5** | 84.8 | 650.3 | 8529.7 | 3900.2 | 3334.7 |
| 164 | [At2g37790](http://atted.jp/cgi-bin/coex_Ath.cgi?gene=At2g37790&sort=all) | | reductase | | | **73.2** | 241.5 | 942.7 | 1390.8 | 19.4 | 5268.5 |
| 165 | 247442_s_at | |  | | | **73.4** | 301.6 | 127.9 | 2875.9 | 1059.5 | 15280.5 |
| 166 | [At5g48600](http://atted.jp/cgi-bin/coex_Ath.cgi?gene=At5g48600&sort=all) | | SMC3 | | | **73.9** | 194.5 | 25.2 | 3529.9 | 14481.0 | 1046.7 |
| 167 | [At5g51590](http://atted.jp/cgi-bin/coex_Ath.cgi?gene=At5g51590&sort=all) | | DNA-binding | | | **73.9** | 288.8 | 334.4 | 2094.1 | 4094.0 | 14991.9 |
| 168 | [At5g49160](http://atted.jp/cgi-bin/coex_Ath.cgi?gene=At5g49160&sort=all) | | MET1 | | | **74.0** | 145.6 | 30.7 | 954.6 | 514.4 | 2050.4 |
| 169 | [At1g67630](http://atted.jp/cgi-bin/coex_Ath.cgi?gene=At1g67630&sort=all) | | POLA2 | | | **74.5** | 269.3 | 73.8 | 1076.1 | 2564.9 | 3344.9 |
| 170 | [At1g72670](http://atted.jp/cgi-bin/coex_Ath.cgi?gene=At1g72670&sort=all) | | iqd8 | | | **74.9** | 156.6 | 62.9 | 8488.7 | 9.7 | 4079.6 |
| 171 | [At3g07510](http://atted.jp/cgi-bin/coex_Ath.cgi?gene=At3g07510&sort=all) | |  | | | **74.9** | 25.3 | 348.5 | 2087.8 | 3402.2 | 17011.5 |
| 172 | [At1g50110](http://atted.jp/cgi-bin/coex_Ath.cgi?gene=At1g50110&sort=all) | | BCAT6 | | | **76.0** | 630.3 | 97.1 | 955.2 | 5650.4 | 19752.5 |
| 173 | [At5g65360](http://atted.jp/cgi-bin/coex_Ath.cgi?gene=At5g65360&sort=all) | | histone H3 | | | **76.2** | 55.6 | 97.1 | 3651.3 | 6328.8 | 5508.7 |
| 174 | [At5g10390](http://atted.jp/cgi-bin/coex_Ath.cgi?gene=At5g10390&sort=all) | | histone H3 | | | **76.7** | 27.6 | 52.0 | 3649.0 | 1082.2 | 14424.4 |
| 175 | [At5g67260](http://atted.jp/cgi-bin/coex_Ath.cgi?gene=At5g67260&sort=all) | | CYCD3;2 | | | **77.6** | 497.0 | 35.4 | 941.0 | 72.1 | 1008.6 |
| 176 | [At1g67750](http://atted.jp/cgi-bin/coex_Ath.cgi?gene=At1g67750&sort=all) | | lyase | | | **78.5** | 155.5 | 122.7 | 777.7 | 1739.0 | 5791.2 |
| 177 | [At5g13290](http://atted.jp/cgi-bin/coex_Ath.cgi?gene=At5g13290&sort=all) | | CRN | | | **78.6** | 150.2 | 282.6 | 2384.0 | 4159.9 | 1637.1 |
| 178 | [At3g51670](http://atted.jp/cgi-bin/coex_Ath.cgi?gene=At3g51670&sort=all) | | SEC14 cytosolic factor | | | **80.1** | 607.7 | 153.2 | 18887.3 | 12228.3 | 11949.2 |
| 179 | [At3g51740](http://atted.jp/cgi-bin/coex_Ath.cgi?gene=At3g51740&sort=all) | | IMK2 | | | **80.9** | 89.3 | 85.5 | 868.5 | 4592.0 | 17032.6 |
| 180 | [At3g12870](http://atted.jp/cgi-bin/coex_Ath.cgi?gene=At3g12870&sort=all) | |  | | | **81.6** | 167.7 | 56.0 | 3197.4 | 5873.5 | 18120.4 |
| 181 | [248633_at](http://atted.jp/cgi-bin/coex_Ath.cgi?gene=248633_at&sort=all) | |  | | | **81.8** | 123.2 | 143.8 | 6489.4 | 3829.1 | 11285.0 |
| 182 | [At5g22880](http://atted.jp/cgi-bin/coex_Ath.cgi?gene=At5g22880&sort=all) | | HTB2 | | | **82.7** | 13.4 | 77.0 | 3201.5 | 7093.4 | 16673.2 |
| 183 | [At1g57820](http://atted.jp/cgi-bin/coex_Ath.cgi?gene=At1g57820&sort=all) | | VIM1 | | | **82.9** | 38.5 | 1886.4 | 3600.9 | 323.6 | 5526.0 |
| 184 | [At1g19950](http://atted.jp/cgi-bin/coex_Ath.cgi?gene=At1g19950&sort=all) | | HVA22H | | | **83.0** | 652.9 | 159.0 | 11.8 | 177.5 | 3631.0 |
| 185 | [At4g01730](http://atted.jp/cgi-bin/coex_Ath.cgi?gene=At4g01730&sort=all) | | zinc ion binding | | | **83.1** | 150.4 | 51.2 | 1057.7 | 2788.3 | 12052.1 |
| 186 | [At4g26660](http://atted.jp/cgi-bin/coex_Ath.cgi?gene=At4g26660&sort=all) | |  | | | **83.4** | 283.6 | 77.3 | 2650.6 | 932.4 | 12293.5 |
| 187 | [At2g40550](http://atted.jp/cgi-bin/coex_Ath.cgi?gene=At2g40550&sort=all) | | ETG1 | | | **83.5** | 122.7 | 116.8 | 4142.9 | 4346.7 | 6725.5 |
| 188 | 255566_s_at | |  | | | **83.5** | 428.4 | 86.7 | 4117.3 | 1049.3 | 15244.7 |
| 189 | [At5g41880](http://atted.jp/cgi-bin/coex_Ath.cgi?gene=At5g41880&sort=all) | | POLA3 | | | **83.5** | 118.3 | 55.3 | 2380.1 | 2620.0 | 8644.4 |
| 190 | [At2g42110](http://atted.jp/cgi-bin/coex_Ath.cgi?gene=At2g42110&sort=all) | |  | | | **83.6** | 180.4 | 85.6 | 75.8 | 37.8 | 8379.1 |
| 191 | [At5g57590](http://atted.jp/cgi-bin/coex_Ath.cgi?gene=At5g57590&sort=all) | | BIO1 | | | **84.3** | 71.2 | 703.8 | 2235.6 | 2128.5 | 4210.3 |
| 192 | [At3g46940](http://atted.jp/cgi-bin/coex_Ath.cgi?gene=At3g46940&sort=all) | | DUT1 | | | **84.4** | 82.0 | 66.1 | 1865.0 | 2395.8 | 161.9 |
| 193 | [At3g11520](http://atted.jp/cgi-bin/coex_Ath.cgi?gene=At3g11520&sort=all) | | CYCB1;3 | | | **85.4** | 134.3 | 54.3 | 1069.4 | 3329.3 | 12606.5 |
| 194 | [At1g33040](http://atted.jp/cgi-bin/coex_Ath.cgi?gene=At1g33040&sort=all) | | NACA5 | | | **87.1** | 352.1 | 9.3 | 1011.9 | 1434.8 | 749.2 |
| 195 | [At2g29890](http://atted.jp/cgi-bin/coex_Ath.cgi?gene=At2g29890&sort=all) | | VLN1 | | | **87.4** | 225.7 | 27.5 | 2270.3 | 4290.2 | 11254.1 |
| 196 | [At2g29570](http://atted.jp/cgi-bin/coex_Ath.cgi?gene=At2g29570&sort=all) | | PCNA2 | | | **87.5** | 21.2 | 37.0 | 9041.7 | 880.3 | 3751.7 |
| 197 | [At2g16270](http://atted.jp/cgi-bin/coex_Ath.cgi?gene=At2g16270&sort=all) | |  | | | **87.9** | 215.2 | 45.3 | 1197.8 | 3610.2 | 6891.5 |
| 198 | [At2g24970](http://atted.jp/cgi-bin/coex_Ath.cgi?gene=At2g24970&sort=all) | |  | | | **88.0** | 148.2 | 56.5 | 4158.4 | 3230.5 | 15297.1 |
| 199 | [At2g33860](http://atted.jp/cgi-bin/coex_Ath.cgi?gene=At2g33860&sort=all) | | ETT | | | **88.6** | 258.1 | 908.8 | 2957.7 | 5205.7 | 1952.8 |
| 200 | [At4g23740](http://atted.jp/cgi-bin/coex_Ath.cgi?gene=At4g23740&sort=all) | | kinase | | | **88.6** | 184.1 | 100.2 | 428.1 | 136.6 | 1938.1 |
| 201 | [At5g43990](http://atted.jp/cgi-bin/coex_Ath.cgi?gene=At5g43990&sort=all) | | SUVR2 | | | **89.5** | 178.2 | 815.5 | 116.0 | 2448.2 | 21077.9 |
| 202 | [At1g02690](http://atted.jp/cgi-bin/coex_Ath.cgi?gene=At1g02690&sort=all) | | IMPA-6 | | | **90.4** | 115.1 | 198.1 | 6877.7 | 19.8 | 15292.6 |
| 203 | [At5g07590](http://atted.jp/cgi-bin/coex_Ath.cgi?gene=At5g07590&sort=all) | | WD-40 repeat | | | **90.5** | 216.2 | 193.0 | 229.7 | 2876.3 | 1264.9 |
| 204 | [At5g42720](http://atted.jp/cgi-bin/coex_Ath.cgi?gene=At5g42720&sort=all) | | hydrolase | | | **90.6** | 91.9 | 1656.5 | 136.8 | 2336.8 | 3996.7 |
| 205 | [At3g55660](http://atted.jp/cgi-bin/coex_Ath.cgi?gene=At3g55660&sort=all) | | ROPGEF6 | | | **90.9** | 257.6 | 32.9 | 1092.0 | 9248.3 | 3410.0 |
| 206 | [At3g08680](http://atted.jp/cgi-bin/coex_Ath.cgi?gene=At3g08680&sort=all) | | kinase | | | **91.7** | 177.5 | 196.1 | 489.0 | 53.7 | 1142.3 |
| 207 | [At1g07790](http://atted.jp/cgi-bin/coex_Ath.cgi?gene=At1g07790&sort=all) | | HTB1 | | | **92.0** | 155.8 | 145.2 | 659.1 | 344.0 | 758.5 |
| 208 | [At1g68400](http://atted.jp/cgi-bin/coex_Ath.cgi?gene=At1g68400&sort=all) | | kinase | | | **92.0** | 413.0 | 402.9 | 1640.8 | 1702.5 | 2033.2 |
| 209 | [At3g54080](http://atted.jp/cgi-bin/coex_Ath.cgi?gene=At3g54080&sort=all) | | sugar binding | | | **92.0** | 110.3 | 303.1 | 6421.3 | 215.2 | 18644.3 |
| 210 | [At1g34065](http://atted.jp/cgi-bin/coex_Ath.cgi?gene=At1g34065&sort=all) | | SAMC2 | | | **92.2** | 156.5 | 291.8 | 2235.8 | 8623.5 | 22261.8 |
| 211 | [At1g30690](http://atted.jp/cgi-bin/coex_Ath.cgi?gene=At1g30690&sort=all) | | SEC14 cytosolic factor | | | **92.5** | 99.6 | 747.9 | 1739.2 | 3115.4 | 17357.6 |
| 212 | [At3g42660](http://atted.jp/cgi-bin/coex_Ath.cgi?gene=At3g42660&sort=all) | | nucleotide binding | | | **92.6** | 166.3 | 247.6 | 399.7 | 4382.0 | 12722.0 |
| 213 | [At3g53380](http://atted.jp/cgi-bin/coex_Ath.cgi?gene=At3g53380&sort=all) | | kinase | | | **92.6** | 294.9 | 91.7 | 1020.4 | 4602.4 | 4126.2 |
| 214 | [At4g25240](http://atted.jp/cgi-bin/coex_Ath.cgi?gene=At4g25240&sort=all) | | SKS1 | | | **93.8** | 824.7 | 132.6 | 3521.3 | 38.2 | 9510.5 |
| 215 | [At2g34710](http://atted.jp/cgi-bin/coex_Ath.cgi?gene=At2g34710&sort=all) | | PHB | | | **93.9** | 218.1 | 145.8 | 2584.9 | 401.1 | 2217.5 |
| 216 | [At3g23670](http://atted.jp/cgi-bin/coex_Ath.cgi?gene=At3g23670&sort=all) | | KINESIN-12B | | | **94.1** | 208.7 | 89.5 | 1589.7 | 2348.3 | 5522.8 |
| 217 | [At2g07170](http://atted.jp/cgi-bin/coex_Ath.cgi?gene=At2g07170&sort=all) | | binding | | | **94.5** | 372.1 | 71.3 | 6835.0 | 16550.2 | 17406.9 |
| 218 | [At5g26850](http://atted.jp/cgi-bin/coex_Ath.cgi?gene=At5g26850&sort=all) | |  | | | **94.7** | 421.1 | 137.7 | 1526.6 | 1190.7 | 12785.0 |
| 219 | [At3g44050](http://atted.jp/cgi-bin/coex_Ath.cgi?gene=At3g44050&sort=all) | | kinesin motor | | | **94.8** | 293.1 | 99.4 | 1692.1 | 515.2 | 14170.8 |
| 220 | [At2g24490](http://atted.jp/cgi-bin/coex_Ath.cgi?gene=At2g24490&sort=all) | | RPA2 | | | **94.9** | 41.4 | 214.6 | 4433.6 | 1855.9 | 4201.4 |
| 221 | [At3g14190](http://atted.jp/cgi-bin/coex_Ath.cgi?gene=At3g14190&sort=all) | |  | | | **95.4** | 177.4 | 68.9 | 1437.5 | 1238.9 | 17530.4 |
| 222 | [At1g63100](http://atted.jp/cgi-bin/coex_Ath.cgi?gene=At1g63100&sort=all) | | transcription | | | **95.9** | 48.5 | 76.8 | 1388.4 | 3481.3 | 22380.4 |
| 223 | [At4g32840](http://atted.jp/cgi-bin/coex_Ath.cgi?gene=At4g32840&sort=all) | | PFK6 | | | **96.1** | 164.5 | 418.2 | 1607.6 | 2095.3 | 1213.2 |
| 224 | [At3g13510](http://atted.jp/cgi-bin/coex_Ath.cgi?gene=At3g13510&sort=all) | |  | | | **96.5** | 183.2 | 83.5 | 1967.6 | 1288.1 | 2262.8 |
| 225 | [At1g10850](http://atted.jp/cgi-bin/coex_Ath.cgi?gene=At1g10850&sort=all) | | kinase | | | **96.6** | 497.3 | 259.6 | 122.4 | 817.5 | 777.7 |
| 226 | [At2g37420](http://atted.jp/cgi-bin/coex_Ath.cgi?gene=At2g37420&sort=all) | | kinesin motor | | | **96.8** | 222.3 | 128.6 | 15765.1 | 6853.6 | 11751.2 |
| 227 | [At2g34190](http://atted.jp/cgi-bin/coex_Ath.cgi?gene=At2g34190&sort=all) | | permease | | | **97.1** | 144.9 | 56.2 | 3536.2 | 288.5 | 12984.7 |
| 228 | [At5g55820](http://atted.jp/cgi-bin/coex_Ath.cgi?gene=At5g55820&sort=all) | |  | | | **97.5** | 272.0 | 84.6 | 2064.1 | 2554.6 | 3594.4 |
| 229 | [At4g22120](http://atted.jp/cgi-bin/coex_Ath.cgi?gene=At4g22120&sort=all) | | ERD | | | **97.8** | 347.1 | 709.4 | 522.9 | 28.4 | 4303.8 |
| 230 | [At1g09450](http://atted.jp/cgi-bin/coex_Ath.cgi?gene=At1g09450&sort=all) | | haspin | | | **98.2** | 278.1 | 33.5 | 5794.0 | 4546.4 | 19438.7 |
| 231 | [At2g13820](http://atted.jp/cgi-bin/coex_Ath.cgi?gene=At2g13820&sort=all) | | LTP | | | **98.3** | 894.4 | 136.5 | 7.5 | 10.7 | 9153.5 |
| 232 | [At3g48425](http://atted.jp/cgi-bin/coex_Ath.cgi?gene=At3g48425&sort=all) | | phosphatase | | | **98.3** | 259.9 | 232.9 | 463.7 | 2300.3 | 5116.2 |
| 233 | [At3g08030](http://atted.jp/cgi-bin/coex_Ath.cgi?gene=At3g08030&sort=all) | |  | | | **99.0** | 317.4 | 142.8 | 1809.3 | 2939.3 | 16710.1 |
| 234 | [At5g55480](http://atted.jp/cgi-bin/coex_Ath.cgi?gene=At5g55480&sort=all) | | SVL1 | | | **99.1** | 447.8 | 482.8 | 60.1 | 675.5 | 4868.4 |
| 235 | [At1g43190](http://atted.jp/cgi-bin/coex_Ath.cgi?gene=At1g43190&sort=all) | | ribonucleoprotein | | | **99.4** | 367.4 | 293.3 | 1027.5 | 2249.5 | 2186.4 |
| 236 | [At5g50375](http://atted.jp/cgi-bin/coex_Ath.cgi?gene=At5g50375&sort=all) | | CPI1 | | | **99.4** | 177.5 | 151.4 | 5887.0 | 238.9 | 15626.1 |
| 237 | [At4g16340](http://atted.jp/cgi-bin/coex_Ath.cgi?gene=At4g16340&sort=all) | | SPK1 | | | **100.2** | 267.1 | 221.7 | 489.5 | 271.2 | 5606.3 |
| 238 | [At2g42840](http://atted.jp/cgi-bin/coex_Ath.cgi?gene=At2g42840&sort=all) | | PDF1 | | | **101.5** | 914.4 | 24.5 | 1208.1 | 175.9 | 684.5 |
| 239 | [At2g23700](http://atted.jp/cgi-bin/coex_Ath.cgi?gene=At2g23700&sort=all) | |  | | | **101.7** | 180.4 | 571.2 | 29.3 | 1788.0 | 690.6 |
| 240 | [At4g37650](http://atted.jp/cgi-bin/coex_Ath.cgi?gene=At4g37650&sort=all) | | SHR | | | **101.7** | 302.6 | 818.0 | 910.8 | 1773.0 | 14453.8 |
| 241 | [At1g73590](http://atted.jp/cgi-bin/coex_Ath.cgi?gene=At1g73590&sort=all) | | PIN1 | | | **102.1** | 246.5 | 219.7 | 14530.9 | 1255.4 | 6658.6 |
| 242 | [At5g11510](http://atted.jp/cgi-bin/coex_Ath.cgi?gene=At5g11510&sort=all) | | MYB3R-4 | | | **102.2** | 168.3 | 75.7 | 465.0 | 6819.2 | 4530.6 |
| 243 | [At1g01370](http://atted.jp/cgi-bin/coex_Ath.cgi?gene=At1g01370&sort=all) | | HTR12 | | | **102.3** | 239.3 | 16.1 | 3134.5 | 4249.7 | 7397.8 |
| 244 | [At2g05760](http://atted.jp/cgi-bin/coex_Ath.cgi?gene=At2g05760&sort=all) | | permease | | | **102.6** | 370.2 | 227.4 | 5111.6 | 1806.9 | 5038.9 |
| 245 | [At5g33300](http://atted.jp/cgi-bin/coex_Ath.cgi?gene=At5g33300&sort=all) | | chromosome-associated kinesin | | | **103.1** | 381.7 | 180.8 | 1460.2 | 4953.4 | 1450.2 |
| 246 | [At1g78770](http://atted.jp/cgi-bin/coex_Ath.cgi?gene=At1g78770&sort=all) | | APC6 | | | **105.7** | 83.3 | 135.7 | 6323.5 | 2853.7 | 303.7 |
| 247 | [At1g35780](http://atted.jp/cgi-bin/coex_Ath.cgi?gene=At1g35780&sort=all) | |  | | | **105.7** | 446.4 | 17.1 | 1049.2 | 7261.6 | 2547.4 |
| 248 | [At4g31890](http://atted.jp/cgi-bin/coex_Ath.cgi?gene=At4g31890&sort=all) | | armadillo/beta-catenin repeat | | | **106.4** | 296.6 | 179.8 | 397.1 | 1182.0 | 1520.2 |
| 249 | [At5g66750](http://atted.jp/cgi-bin/coex_Ath.cgi?gene=At5g66750&sort=all) | | CHR1 | | | **106.6** | 589.7 | 178.3 | 653.5 | 272.3 | 5826.9 |
| 250 | [At5g37010](http://atted.jp/cgi-bin/coex_Ath.cgi?gene=At5g37010&sort=all) | |  | | | **106.7** | 295.8 | 73.1 | 4678.8 | 11934.4 | 15313.1 |
| 251 | [At3g63130](http://atted.jp/cgi-bin/coex_Ath.cgi?gene=At3g63130&sort=all) | | RANGAP1 | | | **106.9** | 96.5 | 131.2 | 3621.7 | 327.1 | 2340.2 |
| 252 | [At2g01120](http://atted.jp/cgi-bin/coex_Ath.cgi?gene=At2g01120&sort=all) | | ORC4 | | | **107.1** | 118.8 | 129.6 | 6398.9 | 1058.4 | 8956.5 |
| 253 | [At3g57830](http://atted.jp/cgi-bin/coex_Ath.cgi?gene=At3g57830&sort=all) | | kinase | | | **108.4** | 323.2 | 118.4 | 347.3 | 6114.5 | 1960.9 |
| 254 | [At3g17360](http://atted.jp/cgi-bin/coex_Ath.cgi?gene=At3g17360&sort=all) | | POK1 | | | **108.5** | 226.7 | 240.5 | 5287.1 | 1528.9 | 13468.5 |
| 255 | [At4g24710](http://atted.jp/cgi-bin/coex_Ath.cgi?gene=At4g24710&sort=all) | | ATPase | | | **109.3** | 416.6 | 96.6 | 2456.8 | 3798.7 | 12823.6 |
| 256 | [At5g04320](http://atted.jp/cgi-bin/coex_Ath.cgi?gene=At5g04320&sort=all) | |  | | | **109.4** | 141.4 | 1183.2 | 7349.4 | 7136.3 | 1995.0 |
| 257 | [At1g66250](http://atted.jp/cgi-bin/coex_Ath.cgi?gene=At1g66250&sort=all) | | hydrolase | | | **111.1** | 689.5 | 64.3 | 11440.0 | 894.4 | 4352.7 |
| 258 | [At1g07880](http://atted.jp/cgi-bin/coex_Ath.cgi?gene=At1g07880&sort=all) | | MPK13 | | | **111.5** | 662.6 | 93.8 | 304.1 | 3503.2 | 21024.9 |
| 259 | [At5g60210](http://atted.jp/cgi-bin/coex_Ath.cgi?gene=At5g60210&sort=all) | | RIP5 | | | **111.6** | 382.8 | 237.9 | 3989.0 | 14686.4 | 3013.6 |
| 260 | [At5g15510](http://atted.jp/cgi-bin/coex_Ath.cgi?gene=At5g15510&sort=all) | |  | | | **111.6** | 188.0 | 118.6 | 9728.6 | 509.6 | 7328.2 |
| 261 | [At3g54750](http://atted.jp/cgi-bin/coex_Ath.cgi?gene=At3g54750&sort=all) | |  | | | **112.2** | 305.7 | 24.2 | 1088.2 | 752.4 | 2442.6 |
| 262 | [At1g18650](http://atted.jp/cgi-bin/coex_Ath.cgi?gene=At1g18650&sort=all) | | PDCB3 | | | **112.4** | 175.0 | 253.5 | 485.8 | 54.1 | 703.9 |
| 263 | [At4g21270](http://atted.jp/cgi-bin/coex_Ath.cgi?gene=At4g21270&sort=all) | | ATK1 | | | **113.4** | 293.1 | 34.5 | 1031.0 | 811.7 | 11286.6 |
| 264 | [At3g61820](http://atted.jp/cgi-bin/coex_Ath.cgi?gene=At3g61820&sort=all) | | protease | | | **114.3** | 247.5 | 186.9 | 5771.4 | 3160.6 | 2178.7 |
| 265 | [At4g28310](http://atted.jp/cgi-bin/coex_Ath.cgi?gene=At4g28310&sort=all) | |  | | | **114.4** | 198.9 | 120.5 | 1220.2 | 1402.9 | 2820.3 |
| 266 | [At1g15660](http://atted.jp/cgi-bin/coex_Ath.cgi?gene=At1g15660&sort=all) | | CENP-C | | | **114.9** | 237.9 | 25.5 | 6331.8 | 6019.2 | 12678.2 |
| 267 | [At1g46264](http://atted.jp/cgi-bin/coex_Ath.cgi?gene=At1g46264&sort=all) | | HSFB4 | | | **115.1** | 66.1 | 1589.6 | 1539.1 | 1178.1 | 2322.1 |
| 268 | [At2g32590](http://atted.jp/cgi-bin/coex_Ath.cgi?gene=At2g32590&sort=all) | |  | | | **115.1** | 263.4 | 56.5 | 800.9 | 629.2 | 3341.0 |
| 269 | [At5g55730](http://atted.jp/cgi-bin/coex_Ath.cgi?gene=At5g55730&sort=all) | | FLA1 | | | **115.3** | 361.1 | 439.6 | 1002.3 | 229.7 | 11337.5 |
| 270 | [At5g60150](http://atted.jp/cgi-bin/coex_Ath.cgi?gene=At5g60150&sort=all) | |  | | | **115.9** | 430.6 | 108.5 | 1474.5 | 16507.1 | 2818.9 |
| 271 | [At5g28290](http://atted.jp/cgi-bin/coex_Ath.cgi?gene=At5g28290&sort=all) | | NEK3 | | | **116.7** | 59.0 | 785.9 | 273.3 | 5695.1 | 649.9 |
| 272 | [At1g74390](http://atted.jp/cgi-bin/coex_Ath.cgi?gene=At1g74390&sort=all) | | nuclease | | | **117.0** | 82.2 | 991.1 | 5450.5 | 4513.1 | 1305.3 |
| 273 | [At4g37490](http://atted.jp/cgi-bin/coex_Ath.cgi?gene=At4g37490&sort=all) | | CYCB1;1 | | | **117.2** | 99.0 | 418.3 | 11077.0 | 1458.3 | 11423.5 |
| 274 | [At1g20930](http://atted.jp/cgi-bin/coex_Ath.cgi?gene=At1g20930&sort=all) | | CDKB2;2 | | | **117.4** | 200.1 | 78.4 | 175.7 | 2202.0 | 16713.0 |
| 275 | [At3g10310](http://atted.jp/cgi-bin/coex_Ath.cgi?gene=At3g10310&sort=all) | | ATP binding | | | **117.8** | 363.0 | 96.9 | 2654.1 | 1756.9 | 9509.1 |
| 276 | [At5g61000](http://atted.jp/cgi-bin/coex_Ath.cgi?gene=At5g61000&sort=all) | | RPA70D | | | **118.3** | 51.4 | 80.5 | 4005.5 | 7019.2 | 19365.3 |
| 277 | [At5g20540](http://atted.jp/cgi-bin/coex_Ath.cgi?gene=At5g20540&sort=all) | | BRXL4 | | | **118.4** | 235.1 | 275.8 | 55.1 | 12282.1 | 76.6 |
| 278 | 247692_s_at | |  | | | **119.3** | 35.0 | 168.6 | 3732.1 | 1010.3 | 7377.1 |
| 279 | [At5g07030](http://atted.jp/cgi-bin/coex_Ath.cgi?gene=At5g07030&sort=all) | | endopeptidase | | | **119.5** | 113.2 | 381.4 | 91.8 | 24.8 | 17891.9 |
| 280 | [At5g08390](http://atted.jp/cgi-bin/coex_Ath.cgi?gene=At5g08390&sort=all) | |  | | | **119.5** | 306.7 | 182.9 | 1478.2 | 14359.8 | 966.5 |
| 281 | [At3g17350](http://atted.jp/cgi-bin/coex_Ath.cgi?gene=At3g17350&sort=all) | |  | | | **120.3** | 83.7 | 1557.9 | 388.1 | 3757.2 | 4853.4 |
| 282 | [At4g28250](http://atted.jp/cgi-bin/coex_Ath.cgi?gene=At4g28250&sort=all) | | EXPB3 | | | **120.4** | 435.4 | 114.9 | 2406.5 | 51.9 | 2298.6 |
| 283 | [At4g17190](http://atted.jp/cgi-bin/coex_Ath.cgi?gene=At4g17190&sort=all) | | FPS2 | | | **120.7** | 385.1 | 182.5 | 2793.3 | 140.9 | 12001.3 |
| 284 | [At1g13170](http://atted.jp/cgi-bin/coex_Ath.cgi?gene=At1g13170&sort=all) | | ORP1D | | | **121.7** | 389.1 | 252.3 | 589.1 | 8434.5 | 8995.9 |
| 285 | [At5g27330](http://atted.jp/cgi-bin/coex_Ath.cgi?gene=At5g27330&sort=all) | |  | | | **121.8** | 253.3 | 321.8 | 6716.9 | 2660.4 | 18530.0 |
| 286 | [At1g15570](http://atted.jp/cgi-bin/coex_Ath.cgi?gene=At1g15570&sort=all) | | CYCA2;3 | | | **123.4** | 335.6 | 484.6 | 1401.9 | 9987.0 | 2770.4 |
| 287 | [At1g16520](http://atted.jp/cgi-bin/coex_Ath.cgi?gene=At1g16520&sort=all) | |  | | | **123.5** | 137.1 | 774.7 | 6894.9 | 185.3 | 5538.7 |
| 288 | [At1g68430](http://atted.jp/cgi-bin/coex_Ath.cgi?gene=At1g68430&sort=all) | |  | | | **124.0** | 364.5 | 477.6 | 192.6 | 263.1 | 7662.4 |
| 289 | [At3g54630](http://atted.jp/cgi-bin/coex_Ath.cgi?gene=At3g54630&sort=all) | |  | | | **124.4** | 152.4 | 93.8 | 4474.4 | 4424.6 | 21310.3 |
| 290 | [At5g58300](http://atted.jp/cgi-bin/coex_Ath.cgi?gene=At5g58300&sort=all) | | kinase | | | **124.7** | 552.6 | 482.9 | 39.8 | 1471.2 | 1593.7 |
| 291 | [At5g60690](http://atted.jp/cgi-bin/coex_Ath.cgi?gene=At5g60690&sort=all) | | REV | | | **125.4** | 168.0 | 461.9 | 8136.9 | 672.5 | 3497.7 |
| 292 | [At4g03210](http://atted.jp/cgi-bin/coex_Ath.cgi?gene=At4g03210&sort=all) | | XTH9 | | | **126.0** | 153.8 | 268.3 | 1360.5 | 862.3 | 2104.8 |
| 293 | [264552_at](http://atted.jp/cgi-bin/coex_Ath.cgi?gene=264552_at&sort=all) | |  | | | **126.5** | 447.8 | 29.6 | 1409.7 | 4776.7 | 18652.6 |
| 294 | [At5g61480](http://atted.jp/cgi-bin/coex_Ath.cgi?gene=At5g61480&sort=all) | | kinase | | | **126.5** | 317.7 | 822.2 | 217.3 | 17.2 | 1864.5 |
| 295 | [At2g28740](http://atted.jp/cgi-bin/coex_Ath.cgi?gene=At2g28740&sort=all) | | HIS4 | | | **126.8** | 68.9 | 158.7 | 3722.4 | 4729.7 | 13320.0 |
| 296 | [At1g31335](http://atted.jp/cgi-bin/coex_Ath.cgi?gene=At1g31335&sort=all) | |  | | | **127.2** | 25.5 | 1174.2 | 318.9 | 5747.8 | 12387.5 |
| 297 | [At5g08020](http://atted.jp/cgi-bin/coex_Ath.cgi?gene=At5g08020&sort=all) | | RPA70B | | | **127.2** | 123.3 | 86.3 | 2764.6 | 5248.4 | 5902.6 |
| 298 | [At1g34355](http://atted.jp/cgi-bin/coex_Ath.cgi?gene=At1g34355&sort=all) | | PS1 | | | **127.3** | 351.7 | 71.4 | 10001.8 | 8497.2 | 11876.9 |
| 299 | [At4g24790](http://atted.jp/cgi-bin/coex_Ath.cgi?gene=At4g24790&sort=all) | | polymerase | | | **128.1** | 229.5 | 1362.4 | 728.9 | 7852.9 | 13766.3 |
| 300 | [At1g05440](http://atted.jp/cgi-bin/coex_Ath.cgi?gene=At1g05440&sort=all) | |  | | | **129.0** | 377.5 | 108.4 | 12363.5 | 1794.4 | 19837.8 |
|  | | | | | | | | | | | |
| **300 coexpressed gene with At5g02140** | | | | | | | | | | | |
|  | **locus** | | **Short description** | | | **MR**  **(all)** | **MR**  **(tissue)** | **MR**  **(abiotic)** | **MR**  **(biotic)** | **MR**  **(hormone)** | **MR**  **(light)** |
| 1 | [At5g03680](http://atted.jp/cgi-bin/coex_Ath.cgi?gene=At5g03680&sort=all) | | PTL | | | **15.0** | 23.9 | 403.9 | 12760.2 | 12135.9 | 10303.2 |
| 2 | [At4g02290](http://atted.jp/cgi-bin/coex_Ath.cgi?gene=At4g02290&sort=all) | | GH9B13 | | | **16.4** | 56.5 | 1072.0 | 995.9 | 8697.3 | 16501.2 |
| 3 | [At4g37730](http://atted.jp/cgi-bin/coex_Ath.cgi?gene=At4g37730&sort=all) | | bZIP7 | | | **17.8** | 8.1 | 13171.3 | 4905.2 | 16486.2 | 11429.7 |
| 4 | [At1g60390](http://atted.jp/cgi-bin/coex_Ath.cgi?gene=At1g60390&sort=all) | | PG1 | | | **19.3** | 162.8 | 553.4 | 5070.1 | 9847.5 | 7220.4 |
| 5 | [At5g14150](http://atted.jp/cgi-bin/coex_Ath.cgi?gene=At5g14150&sort=all) | |  | | | **20.3** | 120.4 | 275.3 | 3403.9 | 4765.0 | 13932.4 |
| 6 | [At1g71870](http://atted.jp/cgi-bin/coex_Ath.cgi?gene=At1g71870&sort=all) | | MATE efflux | | | **25.1** | 52.2 | 1253.7 | 4035.8 | 13059.7 | 11100.5 |
| 7 | [At2g41510](http://atted.jp/cgi-bin/coex_Ath.cgi?gene=At2g41510&sort=all) | | CKX1 | | | **29.4** | 60.8 | 1343.1 | 5667.6 | 6089.4 | 2924.8 |
| 8 | [At1g07880](http://atted.jp/cgi-bin/coex_Ath.cgi?gene=At1g07880&sort=all) | | MPK13 | | | **40.4** | 48.5 | 953.7 | 5657.1 | 13459.4 | 11708.7 |
| 9 | [At2g35120](http://atted.jp/cgi-bin/coex_Ath.cgi?gene=At2g35120&sort=all) | | glycine cleavage system H | | | **45.4** | 85.7 | 1529.9 | 7848.2 | 11643.1 | 2884.1 |
| 10 | [At3g49750](http://atted.jp/cgi-bin/coex_Ath.cgi?gene=At3g49750&sort=all) | | RLP44 | | | **47.9** | 77.2 | 3808.0 | 688.7 | 6041.1 | 12300.5 |
| 11 | [At3g51030](http://atted.jp/cgi-bin/coex_Ath.cgi?gene=At3g51030&sort=all) | | TRX1 | | | **50.8** | 141.8 | 827.9 | 4485.9 | 5889.9 | 7273.3 |
| 12 | [At5g17620](http://atted.jp/cgi-bin/coex_Ath.cgi?gene=At5g17620&sort=all) | |  | | | **59.9** | 317.9 | 258.5 | 4471.3 | 6009.9 | 8771.4 |
| 13 | [At2g36570](http://atted.jp/cgi-bin/coex_Ath.cgi?gene=At2g36570&sort=all) | | kinase | | | **62.6** | 207.9 | 1833.0 | 7791.2 | 14518.0 | 702.9 |
| 14 | [At1g56680](http://atted.jp/cgi-bin/coex_Ath.cgi?gene=At1g56680&sort=all) | | hydrolase | | | **66.8** | 159.1 | 102.2 | 2692.7 | 11242.1 | 5357.2 |
| 15 | [At1g23340](http://atted.jp/cgi-bin/coex_Ath.cgi?gene=At1g23340&sort=all) | |  | | | **68.2** | 75.7 | 4261.5 | 7768.3 | 3103.1 | 8575.7 |
| 16 | [At1g31770](http://atted.jp/cgi-bin/coex_Ath.cgi?gene=At1g31770&sort=all) | | transporter | | | **76.8** | 43.9 | 1376.1 | 9226.1 | 10534.1 | 8173.7 |
| 17 | [At1g67750](http://atted.jp/cgi-bin/coex_Ath.cgi?gene=At1g67750&sort=all) | | lyase | | | **77.0** | 225.7 | 1311.2 | 5773.6 | 4606.2 | 2860.2 |
| 18 | [At1g22030](http://atted.jp/cgi-bin/coex_Ath.cgi?gene=At1g22030&sort=all) | |  | | | **77.6** | 35.8 | 4465.8 | 6643.0 | 4637.1 | 121.2 |
| 19 | [At5g59740](http://atted.jp/cgi-bin/coex_Ath.cgi?gene=At5g59740&sort=all) | | transporter | | | **80.8** | 421.4 | 608.7 | 2235.7 | 1626.9 | 13479.8 |
| 20 | [At1g68430](http://atted.jp/cgi-bin/coex_Ath.cgi?gene=At1g68430&sort=all) | |  | | | **84.1** | 39.7 | 1987.8 | 12847.2 | 13308.2 | 667.3 |
| 21 | [At1g67760](http://atted.jp/cgi-bin/coex_Ath.cgi?gene=At1g67760&sort=all) | | binding | | | **88.2** | 560.1 | 518.8 | 154.8 | 413.2 | 7037.6 |
| 22 | [At5g03840](http://atted.jp/cgi-bin/coex_Ath.cgi?gene=At5g03840&sort=all) | | TFL1 | | | **89.6** | 226.4 | 3014.7 | 5125.8 | 620.8 | 13186.2 |
| 23 | [At2g41300](http://atted.jp/cgi-bin/coex_Ath.cgi?gene=At2g41300&sort=all) | | SSL1 | | | **90.8** | 129.3 | 9259.5 | 1673.5 | 5183.6 | 5664.9 |
| 24 | [At2g42570](http://atted.jp/cgi-bin/coex_Ath.cgi?gene=At2g42570&sort=all) | |  | | | **91.8** | 75.4 | 3659.2 | 13057.7 | 17343.7 | 13129.4 |
| 25 | [246837_at](http://atted.jp/cgi-bin/coex_Ath.cgi?gene=246837_at&sort=all) | |  | | | **96.1** | 362.2 | 408.5 | 5314.1 | 4195.5 | 6569.5 |
| 26 | [At4g31910](http://atted.jp/cgi-bin/coex_Ath.cgi?gene=At4g31910&sort=all) | | transferase | | | **98.5** | 230.5 | 2439.9 | 12549.7 | 10217.7 | 6866.5 |
| 27 | [At5g28650](http://atted.jp/cgi-bin/coex_Ath.cgi?gene=At5g28650&sort=all) | | WRKY74 | | | **102.1** | 756.2 | 1288.0 | 2817.0 | 1338.8 | 8584.7 |
| 28 | [At3g54260](http://atted.jp/cgi-bin/coex_Ath.cgi?gene=At3g54260&sort=all) | |  | | | **102.7** | 216.4 | 1202.7 | 6205.0 | 12314.6 | 5627.2 |
| 29 | [At2g22920](http://atted.jp/cgi-bin/coex_Ath.cgi?gene=At2g22920&sort=all) | | SCPL12 | | | **109.4** | 486.6 | 658.2 | 9339.8 | 16234.7 | 18987.2 |
| 30 | [At3g50960](http://atted.jp/cgi-bin/coex_Ath.cgi?gene=At3g50960&sort=all) | | PLP3a | | | **109.7** | 62.5 | 8050.2 | 3618.1 | 15572.2 | 12017.1 |
| 31 | [At5g20640](http://atted.jp/cgi-bin/coex_Ath.cgi?gene=At5g20640&sort=all) | |  | | | **114.2** | 115.6 | 2625.8 | 10282.0 | 143.4 | 7209.5 |
| 32 | [At5g01840](http://atted.jp/cgi-bin/coex_Ath.cgi?gene=At5g01840&sort=all) | | OFP1 | | | **115.5** | 159.7 | 344.8 | 6896.9 | 13154.2 | 13085.0 |
| 33 | [At1g74055](http://atted.jp/cgi-bin/coex_Ath.cgi?gene=At1g74055&sort=all) | |  | | | **116.4** | 492.1 | 1200.5 | 6787.5 | 3822.3 | 11804.2 |
| 34 | 262939_s_at | |  | | | **121.6** | 60.2 | 3923.3 | 11969.9 | 18965.5 | 12364.8 |
| 35 | [At1g15210](http://atted.jp/cgi-bin/coex_Ath.cgi?gene=At1g15210&sort=all) | | PDR7 | | | **124.5** | 438.4 | 688.8 | 3391.8 | 14128.7 | 16479.8 |
| 36 | [At1g72730](http://atted.jp/cgi-bin/coex_Ath.cgi?gene=At1g72730&sort=all) | | translation | | | **126.6** | 394.0 | 155.8 | 15714.2 | 17290.6 | 9288.4 |
| 37 | [At1g04340](http://atted.jp/cgi-bin/coex_Ath.cgi?gene=At1g04340&sort=all) | | lesion inducing | | | **132.2** | 31.2 | 237.0 | 16599.0 | 18269.5 | 3002.7 |
| 38 | [At5g45560](http://atted.jp/cgi-bin/coex_Ath.cgi?gene=At5g45560&sort=all) | | PH | | | **133.9** | 145.0 | 1269.1 | 9113.1 | 15231.0 | 9239.6 |
| 39 | [At3g21770](http://atted.jp/cgi-bin/coex_Ath.cgi?gene=At3g21770&sort=all) | | PRXR9 | | | **134.3** | 221.5 | 5126.0 | 2330.4 | 16994.5 | 11801.2 |
| 40 | [At1g70710](http://atted.jp/cgi-bin/coex_Ath.cgi?gene=At1g70710&sort=all) | | GH9B1 | | | **136.4** | 412.9 | 893.7 | 11787.0 | 9753.1 | 15599.1 |
| 41 | [At3g54310](http://atted.jp/cgi-bin/coex_Ath.cgi?gene=At3g54310&sort=all) | |  | | | **138.9** | 232.8 | 7802.5 | 1216.1 | 62.2 | 5486.0 |
| 42 | [245485_at](http://atted.jp/cgi-bin/coex_Ath.cgi?gene=245485_at&sort=all) | |  | | | **139.9** | 490.3 | 395.4 | 10215.4 | 1079.0 | 11617.1 |
| 43 | [At5g62960](http://atted.jp/cgi-bin/coex_Ath.cgi?gene=At5g62960&sort=all) | |  | | | **141.8** | 807.5 | 236.0 | 4828.3 | 2603.9 | 202.5 |
| 44 | [At4g23490](http://atted.jp/cgi-bin/coex_Ath.cgi?gene=At4g23490&sort=all) | | fringe | | | **143.2** | 465.6 | 899.8 | 5098.0 | 13494.8 | 4520.4 |
| 45 | [At5g48060](http://atted.jp/cgi-bin/coex_Ath.cgi?gene=At5g48060&sort=all) | | C2 | | | **149.5** | 1150.8 | 1208.4 | 384.7 | 938.4 | 403.3 |
| 46 | [At3g59680](http://atted.jp/cgi-bin/coex_Ath.cgi?gene=At3g59680&sort=all) | |  | | | **151.2** | 195.8 | 8989.9 | 9830.0 | 6440.8 | 7193.9 |
| 47 | [At3g01410](http://atted.jp/cgi-bin/coex_Ath.cgi?gene=At3g01410&sort=all) | | RNase H | | | **152.5** | 863.8 | 232.8 | 2494.7 | 4357.4 | 2888.3 |
| 48 | [At4g39320](http://atted.jp/cgi-bin/coex_Ath.cgi?gene=At4g39320&sort=all) | | microtubule-associated | | | **153.1** | 371.1 | 122.3 | 11225.9 | 3764.7 | 14483.8 |
| 49 | [At4g27270](http://atted.jp/cgi-bin/coex_Ath.cgi?gene=At4g27270&sort=all) | | reductase | | | **156.7** | 69.7 | 1785.6 | 19085.7 | 15623.7 | 11289.9 |
| 50 | [At1g02810](http://atted.jp/cgi-bin/coex_Ath.cgi?gene=At1g02810&sort=all) | | pectinesterase | | | **157.0** | 292.7 | 954.7 | 1232.2 | 11061.7 | 14603.1 |
| 51 | [At2g35150](http://atted.jp/cgi-bin/coex_Ath.cgi?gene=At2g35150&sort=all) | | EXL1 | | | **158.3** | 46.2 | 17867.5 | 12166.1 | 13800.4 | 13123.5 |
| 52 | [At5g45920](http://atted.jp/cgi-bin/coex_Ath.cgi?gene=At5g45920&sort=all) | | hydrolase | | | **159.9** | 1747.6 | 631.6 | 5773.3 | 2281.7 | 7034.3 |
| 53 | [At1g68810](http://atted.jp/cgi-bin/coex_Ath.cgi?gene=At1g68810&sort=all) | | bHLH | | | **160.6** | 143.0 | 5328.5 | 9775.3 | 14414.6 | 3022.1 |
| 54 | [At1g78120](http://atted.jp/cgi-bin/coex_Ath.cgi?gene=At1g78120&sort=all) | | TPR | | | **162.5** | 286.4 | 1420.2 | 2493.1 | 10986.0 | 6631.5 |
| 55 | 248559_at | |  | | | **164.9** | 108.0 | 10132.4 | 10183.5 | 6101.8 | 14585.7 |
| 56 | [At4g11190](http://atted.jp/cgi-bin/coex_Ath.cgi?gene=At4g11190&sort=all) | | dirigent | | | **165.7** | 220.5 | 1143.8 | 8439.8 | 10218.2 | 6132.3 |
| 57 | [At5g33300](http://atted.jp/cgi-bin/coex_Ath.cgi?gene=At5g33300&sort=all) | | chromosome-associated kinesin | | | **169.6** | 271.4 | 452.9 | 9827.6 | 16083.3 | 13252.8 |
| 58 | [At2g38060](http://atted.jp/cgi-bin/coex_Ath.cgi?gene=At2g38060&sort=all) | | PHT4;2 | | | **178.4** | 236.6 | 12077.4 | 3796.3 | 2882.0 | 3476.0 |
| 59 | [At5g22740](http://atted.jp/cgi-bin/coex_Ath.cgi?gene=At5g22740&sort=all) | | CSLA02 | | | **179.6** | 200.9 | 1117.1 | 9730.6 | 11454.6 | 14174.6 |
| 60 | [At1g47670](http://atted.jp/cgi-bin/coex_Ath.cgi?gene=At1g47670&sort=all) | | transporter | | | **187.8** | 324.4 | 1590.8 | 6981.8 | 9423.6 | 3396.5 |
| 61 | [At4g14130](http://atted.jp/cgi-bin/coex_Ath.cgi?gene=At4g14130&sort=all) | | XTR7 | | | **187.8** | 20.7 | 5321.6 | 2852.8 | 12802.2 | 15449.3 |
| 62 | [At1g12310](http://atted.jp/cgi-bin/coex_Ath.cgi?gene=At1g12310&sort=all) | | calmodulin | | | **187.8** | 235.1 | 486.0 | 8094.9 | 8983.9 | 2105.7 |
| 63 | [At5g38300](http://atted.jp/cgi-bin/coex_Ath.cgi?gene=At5g38300&sort=all) | |  | | | **189.4** | 812.3 | 849.8 | 492.1 | 1744.4 | 10605.0 |
| 64 | [At5g10850](http://atted.jp/cgi-bin/coex_Ath.cgi?gene=At5g10850&sort=all) | | transposable | | | **190.2** | 676.6 | 757.6 | 6882.6 | 4359.2 | 9338.4 |
| 65 | [At1g73640](http://atted.jp/cgi-bin/coex_Ath.cgi?gene=At1g73640&sort=all) | | RABA6a | | | **190.8** | 470.1 | 3538.7 | 4567.9 | 2389.6 | 5905.9 |
| 66 | [At5g59160](http://atted.jp/cgi-bin/coex_Ath.cgi?gene=At5g59160&sort=all) | | TOPP2 | | | **196.4** | 26.8 | 383.8 | 12253.0 | 21894.1 | 19235.9 |
| 67 | [At5g67460](http://atted.jp/cgi-bin/coex_Ath.cgi?gene=At5g67460&sort=all) | | hydrolase | | | **198.5** | 316.2 | 5809.3 | 10377.8 | 4425.0 | 5909.5 |
| 68 | [At2g37590](http://atted.jp/cgi-bin/coex_Ath.cgi?gene=At2g37590&sort=all) | | zinc finger | | | **198.6** | 187.7 | 6637.6 | 2114.8 | 4627.3 | 89.8 |
| 69 | [At3g44940](http://atted.jp/cgi-bin/coex_Ath.cgi?gene=At3g44940&sort=all) | |  | | | **201.2** | 109.5 | 1808.7 | 7283.5 | 5368.8 | 12758.5 |
| 70 | [At2g47010](http://atted.jp/cgi-bin/coex_Ath.cgi?gene=At2g47010&sort=all) | |  | | | **202.4** | 92.6 | 10111.0 | 7104.2 | 10243.1 | 4846.9 |
| 71 | [At2g17630](http://atted.jp/cgi-bin/coex_Ath.cgi?gene=At2g17630&sort=all) | | transferase | | | **202.7** | 476.0 | 429.2 | 9138.5 | 12600.5 | 7864.7 |
| 72 | [At1g66950](http://atted.jp/cgi-bin/coex_Ath.cgi?gene=At1g66950&sort=all) | | PDR11 | | | **203.2** | 1213.8 | 123.9 | 4973.4 | 7491.0 | 3604.3 |
| 73 | [At2g27740](http://atted.jp/cgi-bin/coex_Ath.cgi?gene=At2g27740&sort=all) | |  | | | **204.3** | 325.8 | 3836.5 | 12694.7 | 13507.5 | 9428.8 |
| 74 | [At3g19184](http://atted.jp/cgi-bin/coex_Ath.cgi?gene=At3g19184&sort=all) | | DNA binding | | | **204.4** | 574.5 | 4615.2 | 12451.7 | 1301.5 | 1672.0 |
| 75 | [At4g08690](http://atted.jp/cgi-bin/coex_Ath.cgi?gene=At4g08690&sort=all) | | SEC14 cytosolic factor | | | **208.4** | 91.4 | 9159.1 | 12017.7 | 13057.1 | 17513.7 |
| 76 | [At1g18140](http://atted.jp/cgi-bin/coex_Ath.cgi?gene=At1g18140&sort=all) | | LAC1 | | | **211.8** | 51.4 | 11064.9 | 11964.1 | 15528.4 | 5399.5 |
| 77 | [At3g03160](http://atted.jp/cgi-bin/coex_Ath.cgi?gene=At3g03160&sort=all) | |  | | | **212.8** | 588.8 | 952.7 | 7928.5 | 8518.1 | 5586.6 |
| 78 | [At1g44970](http://atted.jp/cgi-bin/coex_Ath.cgi?gene=At1g44970&sort=all) | | peroxidase | | | **218.8** | 427.1 | 2187.8 | 1236.8 | 8610.9 | 8767.8 |
| 79 | [At5g63660](http://atted.jp/cgi-bin/coex_Ath.cgi?gene=At5g63660&sort=all) | | PDF2.5 | | | **218.9** | 725.4 | 417.6 | 5051.9 | 12163.4 | 5725.4 |
| 80 | [At5g59240](http://atted.jp/cgi-bin/coex_Ath.cgi?gene=At5g59240&sort=all) | | RPS8B | | | **221.7** | 7.5 | 494.0 | 17756.8 | 13241.7 | 15950.3 |
| 81 | [At2g44190](http://atted.jp/cgi-bin/coex_Ath.cgi?gene=At2g44190&sort=all) | | EDE1 | | | **222.3** | 1058.5 | 753.1 | 1206.6 | 2489.4 | 6472.0 |
| 82 | [At4g28250](http://atted.jp/cgi-bin/coex_Ath.cgi?gene=At4g28250&sort=all) | | EXPB3 | | | **222.6** | 103.3 | 1717.0 | 12657.9 | 14239.6 | 5853.7 |
| 83 | 266401_s_at | |  | | | **223.4** | 722.7 | 2001.3 | 6811.9 | 8087.9 | 6506.4 |
| 84 | [At3g25560](http://atted.jp/cgi-bin/coex_Ath.cgi?gene=At3g25560&sort=all) | | NIK2 | | | **225.8** | 145.3 | 10261.7 | 6717.6 | 4544.1 | 1774.2 |
| 85 | [At5g58300](http://atted.jp/cgi-bin/coex_Ath.cgi?gene=At5g58300&sort=all) | | kinase | | | **225.8** | 297.5 | 4153.2 | 10337.0 | 20478.2 | 10495.6 |
| 86 | [At3g53880](http://atted.jp/cgi-bin/coex_Ath.cgi?gene=At3g53880&sort=all) | | reductase | | | **225.9** | 23.0 | 2568.9 | 10701.8 | 17151.4 | 7526.3 |
| 87 | [At5g03870](http://atted.jp/cgi-bin/coex_Ath.cgi?gene=At5g03870&sort=all) | | glutaredoxin | | | **226.4** | 1219.9 | 504.4 | 1246.8 | 5297.2 | 4533.2 |
| 88 | [At5g24310](http://atted.jp/cgi-bin/coex_Ath.cgi?gene=At5g24310&sort=all) | | ABIL3 | | | **229.5** | 276.8 | 4850.6 | 6944.1 | 9912.0 | 11658.3 |
| 89 | [At5g11980](http://atted.jp/cgi-bin/coex_Ath.cgi?gene=At5g11980&sort=all) | | COG complex component | | | **230.7** | 495.3 | 840.1 | 3572.2 | 10308.0 | 6709.8 |
| 90 | [At5g65810](http://atted.jp/cgi-bin/coex_Ath.cgi?gene=At5g65810&sort=all) | |  | | | **232.8** | 537.9 | 161.6 | 7106.5 | 14151.3 | 9023.2 |
| 91 | [At5g02370](http://atted.jp/cgi-bin/coex_Ath.cgi?gene=At5g02370&sort=all) | | kinesin motor | | | **233.1** | 916.4 | 779.6 | 3024.7 | 7368.9 | 7884.9 |
| 92 | [At5g46700](http://atted.jp/cgi-bin/coex_Ath.cgi?gene=At5g46700&sort=all) | | TRN2 | | | **235.5** | 528.9 | 3294.7 | 8816.6 | 14389.9 | 1240.1 |
| 93 | [At1g06420](http://atted.jp/cgi-bin/coex_Ath.cgi?gene=At1g06420&sort=all) | |  | | | **240.3** | 1047.8 | 1951.7 | 13060.7 | 5517.6 | 6714.6 |
| 94 | [At3g26550](http://atted.jp/cgi-bin/coex_Ath.cgi?gene=At3g26550&sort=all) | | DC1 | | | **240.4** | 361.0 | 2173.8 | 3586.0 | 1463.9 | 7397.3 |
| 95 | [At1g06490](http://atted.jp/cgi-bin/coex_Ath.cgi?gene=At1g06490&sort=all) | | GSL07 | | | **243.6** | 1348.2 | 579.0 | 1651.5 | 2874.7 | 5587.2 |
| 96 | [At5g39320](http://atted.jp/cgi-bin/coex_Ath.cgi?gene=At5g39320&sort=all) | | dehydrogenase | | | **244.3** | 271.1 | 1134.5 | 3439.8 | 17104.7 | 14872.0 |
| 97 | [At1g75780](http://atted.jp/cgi-bin/coex_Ath.cgi?gene=At1g75780&sort=all) | | TUB1 | | | **244.3** | 160.4 | 1265.7 | 5425.2 | 11850.7 | 17314.1 |
| 98 | [At1g80690](http://atted.jp/cgi-bin/coex_Ath.cgi?gene=At1g80690&sort=all) | |  | | | **247.3** | 180.2 | 5419.9 | 4814.9 | 15143.5 | 1590.5 |
| 99 | [At2g15280](http://atted.jp/cgi-bin/coex_Ath.cgi?gene=At2g15280&sort=all) | | RTNLB10 | | | **249.0** | 388.1 | 1614.4 | 7352.8 | 6791.9 | 2451.1 |
| 100 | [At4g31340](http://atted.jp/cgi-bin/coex_Ath.cgi?gene=At4g31340&sort=all) | | myosin | | | **253.2** | 89.5 | 2310.5 | 14875.8 | 18668.0 | 13824.2 |
| 101 | [At4g20230](http://atted.jp/cgi-bin/coex_Ath.cgi?gene=At4g20230&sort=all) | | cyclase | | | **253.2** | 1082.7 | 1817.8 | 3153.9 | 11118.4 | 22545.0 |
| 102 | 266643_s_at | |  | | | **255.1** | 889.2 | 590.8 | 4701.6 | 8644.4 | 17440.3 |
| 103 | 263397_s_at | |  | | | **255.1** | 1201.9 | 1093.4 | 186.5 | 765.8 | 3717.4 |
| 104 | [At3g49860](http://atted.jp/cgi-bin/coex_Ath.cgi?gene=At3g49860&sort=all) | | ARLA1B | | | **257.2** | 970.8 | 1160.9 | 3255.2 | 6141.6 | 10377.1 |
| 105 | [At5g15460](http://atted.jp/cgi-bin/coex_Ath.cgi?gene=At5g15460&sort=all) | | MUB2 | | | **257.3** | 150.0 | 2555.7 | 13154.6 | 13123.9 | 19274.8 |
| 106 | [At2g25810](http://atted.jp/cgi-bin/coex_Ath.cgi?gene=At2g25810&sort=all) | | TIP4;1 | | | **257.4** | 844.3 | 388.2 | 4089.2 | 10994.3 | 12130.9 |
| 107 | [At5g51590](http://atted.jp/cgi-bin/coex_Ath.cgi?gene=At5g51590&sort=all) | | DNA-binding | | | **258.3** | 677.5 | 2871.0 | 2349.8 | 10181.4 | 8655.7 |
| 108 | [At3g09070](http://atted.jp/cgi-bin/coex_Ath.cgi?gene=At3g09070&sort=all) | | glycine-rich | | | **265.6** | 279.4 | 6498.1 | 19751.1 | 12255.4 | 6374.2 |
| 109 | 261555_s_at | |  | | | **268.4** | 902.4 | 2728.4 | 2896.6 | 685.0 | 13344.5 |
| 110 | [At2g43610](http://atted.jp/cgi-bin/coex_Ath.cgi?gene=At2g43610&sort=all) | | hydrolase | | | **269.6** | 447.9 | 1821.4 | 16156.3 | 13974.4 | 16348.5 |
| 111 | [At1g21090](http://atted.jp/cgi-bin/coex_Ath.cgi?gene=At1g21090&sort=all) | | glycoprotein | | | **272.7** | 1170.7 | 883.3 | 12054.6 | 3615.3 | 2344.0 |
| 112 | [At2g28250](http://atted.jp/cgi-bin/coex_Ath.cgi?gene=At2g28250&sort=all) | | NCRK | | | **273.7** | 844.4 | 1341.9 | 4121.6 | 10980.5 | 9429.5 |
| 113 | [At5g45700](http://atted.jp/cgi-bin/coex_Ath.cgi?gene=At5g45700&sort=all) | | NIF | | | **273.9** | 681.9 | 851.0 | 5674.3 | 19321.3 | 3453.0 |
| 114 | [At3g20450](http://atted.jp/cgi-bin/coex_Ath.cgi?gene=At3g20450&sort=all) | |  | | | **274.2** | 603.5 | 1931.0 | 3628.9 | 751.5 | 12382.7 |
| 115 | [At3g58800](http://atted.jp/cgi-bin/coex_Ath.cgi?gene=At3g58800&sort=all) | |  | | | **278.6** | 939.0 | 1081.9 | 4884.0 | 1921.0 | 13216.5 |
| 116 | [At2g32380](http://atted.jp/cgi-bin/coex_Ath.cgi?gene=At2g32380&sort=all) | |  | | | **278.8** | 190.6 | 1341.3 | 15986.4 | 16823.0 | 14562.8 |
| 117 | [At2g26360](http://atted.jp/cgi-bin/coex_Ath.cgi?gene=At2g26360&sort=all) | | binding | | | **279.4** | 811.8 | 788.9 | 9066.6 | 6493.0 | 11408.0 |
| 118 | [At1g68360](http://atted.jp/cgi-bin/coex_Ath.cgi?gene=At1g68360&sort=all) | | zinc finger | | | **279.6** | 161.2 | 9999.3 | 5454.9 | 4128.0 | 9845.4 |
| 119 | [At3g20150](http://atted.jp/cgi-bin/coex_Ath.cgi?gene=At3g20150&sort=all) | | kinesin motor | | | **281.7** | 950.6 | 381.2 | 2453.7 | 17578.8 | 15423.4 |
| 120 | [At3g04230](http://atted.jp/cgi-bin/coex_Ath.cgi?gene=At3g04230&sort=all) | | RPS16B | | | **285.1** | 15.9 | 1400.5 | 13940.1 | 8906.5 | 8475.4 |
| 121 | [At5g06940](http://atted.jp/cgi-bin/coex_Ath.cgi?gene=At5g06940&sort=all) | | LRR | | | **285.9** | 1721.0 | 1223.6 | 747.8 | 917.0 | 6094.1 |
| 122 | [At5g37980](http://atted.jp/cgi-bin/coex_Ath.cgi?gene=At5g37980&sort=all) | | oxidoreductase | | | **288.3** | 206.6 | 7214.3 | 752.6 | 1453.3 | 17960.0 |
| 123 | [At3g45090](http://atted.jp/cgi-bin/coex_Ath.cgi?gene=At3g45090&sort=all) | | kinase | | | **294.8** | 191.8 | 2170.8 | 16024.6 | 19231.8 | 15340.2 |
| 124 | [At3g47460](http://atted.jp/cgi-bin/coex_Ath.cgi?gene=At3g47460&sort=all) | | SMC2 | | | **296.2** | 1447.2 | 767.7 | 857.5 | 1785.4 | 5662.3 |
| 125 | [At1g10460](http://atted.jp/cgi-bin/coex_Ath.cgi?gene=At1g10460&sort=all) | | GLP7 | | | **296.4** | 686.8 | 3189.8 | 6534.6 | 3591.6 | 8405.3 |
| 126 | [At3g43810](http://atted.jp/cgi-bin/coex_Ath.cgi?gene=At3g43810&sort=all) | | CAM7 | | | **297.6** | 606.6 | 373.3 | 11398.7 | 10335.3 | 18859.3 |
| 127 | [At3g50300](http://atted.jp/cgi-bin/coex_Ath.cgi?gene=At3g50300&sort=all) | | transferase | | | **297.8** | 911.6 | 284.3 | 11035.0 | 15189.9 | 12854.3 |
| 128 | [At1g05210](http://atted.jp/cgi-bin/coex_Ath.cgi?gene=At1g05210&sort=all) | |  | | | **300.0** | 755.0 | 2080.5 | 9465.5 | 6092.9 | 156.7 |
| 129 | [At5g60200](http://atted.jp/cgi-bin/coex_Ath.cgi?gene=At5g60200&sort=all) | | TMO6 | | | **304.4** | 280.4 | 2999.6 | 7272.1 | 14252.0 | 1218.0 |
| 130 | [At5g48740](http://atted.jp/cgi-bin/coex_Ath.cgi?gene=At5g48740&sort=all) | | kinase | | | **306.8** | 773.3 | 4879.2 | 9320.8 | 2424.8 | 106.3 |
| 131 | [At4g32830](http://atted.jp/cgi-bin/coex_Ath.cgi?gene=At4g32830&sort=all) | | AUR1 | | | **308.8** | 655.8 | 1186.9 | 2503.5 | 14591.3 | 10227.1 |
| 132 | [At1g16330](http://atted.jp/cgi-bin/coex_Ath.cgi?gene=At1g16330&sort=all) | | CYCB3;1 | | | **309.1** | 827.2 | 808.9 | 7669.6 | 3376.7 | 8902.8 |
| 133 | 259077_s_at | |  | | | **309.2** | 153.8 | 1039.0 | 9772.5 | 18422.4 | 15973.1 |
| 134 | [At2g01950](http://atted.jp/cgi-bin/coex_Ath.cgi?gene=At2g01950&sort=all) | | BRL2 | | | **311.6** | 482.7 | 779.6 | 9007.2 | 16809.0 | 6593.2 |
| 135 | [At1g16070](http://atted.jp/cgi-bin/coex_Ath.cgi?gene=At1g16070&sort=all) | | TLP8 | | | **313.2** | 1146.8 | 1416.5 | 1824.8 | 7206.5 | 5585.6 |
| 136 | [At2g39830](http://atted.jp/cgi-bin/coex_Ath.cgi?gene=At2g39830&sort=all) | | DAR2 | | | **315.5** | 1759.4 | 549.7 | 4728.0 | 3501.3 | 4871.7 |
| 137 | [At3g54250](http://atted.jp/cgi-bin/coex_Ath.cgi?gene=At3g54250&sort=all) | | decarboxylase | | | **316.8** | 685.7 | 2101.4 | 9257.5 | 18374.2 | 9572.6 |
| 138 | [At2g39700](http://atted.jp/cgi-bin/coex_Ath.cgi?gene=At2g39700&sort=all) | | EXPA4 | | | **317.2** | 84.7 | 2038.5 | 14671.6 | 15495.5 | 9021.1 |
| 139 | [At1g50490](http://atted.jp/cgi-bin/coex_Ath.cgi?gene=At1g50490&sort=all) | | UBC20 | | | **318.2** | 297.8 | 1088.3 | 16167.1 | 20338.6 | 7187.4 |
| 140 | [At1g14830](http://atted.jp/cgi-bin/coex_Ath.cgi?gene=At1g14830&sort=all) | | ADL1C | | | **319.4** | 205.8 | 760.3 | 6216.8 | 19139.5 | 11336.6 |
| 141 | [At1g33170](http://atted.jp/cgi-bin/coex_Ath.cgi?gene=At1g33170&sort=all) | | dehydration-responsive | | | **319.5** | 213.1 | 5406.5 | 7074.7 | 9911.8 | 5437.6 |
| 142 | [At3g24660](http://atted.jp/cgi-bin/coex_Ath.cgi?gene=At3g24660&sort=all) | | TMKL1 | | | **321.0** | 962.0 | 1059.4 | 15767.7 | 13377.9 | 1393.0 |
| 143 | [At4g24710](http://atted.jp/cgi-bin/coex_Ath.cgi?gene=At4g24710&sort=all) | | ATPase | | | **322.4** | 1177.3 | 750.2 | 964.0 | 8006.8 | 4030.1 |
| 144 | [At4g21310](http://atted.jp/cgi-bin/coex_Ath.cgi?gene=At4g21310&sort=all) | |  | | | **324.5** | 1175.9 | 2459.6 | 1058.1 | 2716.4 | 5376.8 |
| 145 | [At5g01870](http://atted.jp/cgi-bin/coex_Ath.cgi?gene=At5g01870&sort=all) | | lipid transfer | | | **325.3** | 1296.4 | 1177.1 | 601.5 | 8228.7 | 13537.1 |
| 146 | [At4g35620](http://atted.jp/cgi-bin/coex_Ath.cgi?gene=At4g35620&sort=all) | | CYCB2;2 | | | **328.2** | 920.2 | 333.9 | 1292.2 | 6593.8 | 18423.2 |
| 147 | [At2g23380](http://atted.jp/cgi-bin/coex_Ath.cgi?gene=At2g23380&sort=all) | | CLF | | | **330.9** | 326.8 | 236.8 | 5611.4 | 18302.0 | 5849.2 |
| 148 | [At1g64920](http://atted.jp/cgi-bin/coex_Ath.cgi?gene=At1g64920&sort=all) | | transferase | | | **331.4** | 2243.8 | 9.2 | 4599.7 | 15460.4 | 9612.9 |
| 149 | [At4g17905](http://atted.jp/cgi-bin/coex_Ath.cgi?gene=At4g17905&sort=all) | | L4H | | | **331.6** | 655.6 | 2863.1 | 3770.0 | 1180.7 | 15909.8 |
| 150 | [At3g25130](http://atted.jp/cgi-bin/coex_Ath.cgi?gene=At3g25130&sort=all) | |  | | | **335.4** | 281.6 | 6902.4 | 17218.8 | 11073.9 | 20459.2 |
| 151 | [At4g24180](http://atted.jp/cgi-bin/coex_Ath.cgi?gene=At4g24180&sort=all) | | TLP1 | | | **336.5** | 698.4 | 1199.6 | 4606.6 | 5490.7 | 5036.6 |
| 152 | [At5g16720](http://atted.jp/cgi-bin/coex_Ath.cgi?gene=At5g16720&sort=all) | |  | | | **336.5** | 43.5 | 4974.2 | 13810.1 | 20582.8 | 5622.9 |
| 153 | [At5g07720](http://atted.jp/cgi-bin/coex_Ath.cgi?gene=At5g07720&sort=all) | | GMA12/MNN10 | | | **338.0** | 1035.5 | 1008.5 | 1728.0 | 7712.0 | 9773.2 |
| 154 | [At1g52820](http://atted.jp/cgi-bin/coex_Ath.cgi?gene=At1g52820&sort=all) | | dioxygenase | | | **338.7** | 2244.8 | 28.2 | 3488.2 | 6755.2 | 17906.1 |
| 155 | [At1g61760](http://atted.jp/cgi-bin/coex_Ath.cgi?gene=At1g61760&sort=all) | | HIN1-related | | | **344.3** | 1603.4 | 621.2 | 7523.1 | 2169.4 | 5150.9 |
| 156 | [At5g49560](http://atted.jp/cgi-bin/coex_Ath.cgi?gene=At5g49560&sort=all) | |  | | | **349.7** | 884.8 | 706.9 | 11694.2 | 8553.7 | 2137.7 |
| 157 | [At3g03910](http://atted.jp/cgi-bin/coex_Ath.cgi?gene=At3g03910&sort=all) | | GDH3 | | | **350.0** | 449.2 | 10088.8 | 11159.2 | 2947.4 | 158.4 |
| 158 | [At5g23430](http://atted.jp/cgi-bin/coex_Ath.cgi?gene=At5g23430&sort=all) | | transducin | | | **350.5** | 189.3 | 2274.1 | 12909.3 | 15524.9 | 3399.6 |
| 159 | [At1g23890](http://atted.jp/cgi-bin/coex_Ath.cgi?gene=At1g23890&sort=all) | | NHL repeat | | | **351.3** | 168.6 | 12069.5 | 2440.5 | 3803.4 | 2090.1 |
| 160 | [At3g08930](http://atted.jp/cgi-bin/coex_Ath.cgi?gene=At3g08930&sort=all) | |  | | | **351.4** | 200.1 | 3104.0 | 8449.6 | 9981.2 | 8750.3 |
| 161 | [At1g71692](http://atted.jp/cgi-bin/coex_Ath.cgi?gene=At1g71692&sort=all) | | AGL12 | | | **351.9** | 541.4 | 4756.1 | 5008.5 | 7729.6 | 14934.9 |
| 162 | [At4g39630](http://atted.jp/cgi-bin/coex_Ath.cgi?gene=At4g39630&sort=all) | |  | | | **353.8** | 840.2 | 1661.6 | 4146.7 | 9763.4 | 1634.0 |
| 163 | [At3g51720](http://atted.jp/cgi-bin/coex_Ath.cgi?gene=At3g51720&sort=all) | |  | | | **358.4** | 630.2 | 1652.1 | 9488.7 | 18129.2 | 18750.8 |
| 164 | [At3g11520](http://atted.jp/cgi-bin/coex_Ath.cgi?gene=At3g11520&sort=all) | | CYCB1;3 | | | **359.7** | 706.5 | 830.8 | 1818.4 | 6038.9 | 5631.2 |
| 165 | [At2g23050](http://atted.jp/cgi-bin/coex_Ath.cgi?gene=At2g23050&sort=all) | | NPY4 | | | **361.7** | 1125.8 | 419.2 | 7289.8 | 11998.2 | 12574.4 |
| 166 | [At2g01610](http://atted.jp/cgi-bin/coex_Ath.cgi?gene=At2g01610&sort=all) | | inhibitor | | | **362.8** | 832.0 | 1469.5 | 10206.2 | 2329.4 | 6597.9 |
| 167 | [At3g22790](http://atted.jp/cgi-bin/coex_Ath.cgi?gene=At3g22790&sort=all) | | kinase interacting | | | **363.8** | 1104.8 | 1301.3 | 5370.3 | 20806.7 | 9586.3 |
| 168 | [At1g31320](http://atted.jp/cgi-bin/coex_Ath.cgi?gene=At1g31320&sort=all) | | LBD4 | | | **365.2** | 237.7 | 2236.6 | 10406.1 | 14775.4 | 8033.7 |
| 169 | [At2g04650](http://atted.jp/cgi-bin/coex_Ath.cgi?gene=At2g04650&sort=all) | | pyrophosphorylase | | | **368.3** | 113.7 | 2570.5 | 18140.5 | 18390.5 | 8855.2 |
| 170 | [At3g02885](http://atted.jp/cgi-bin/coex_Ath.cgi?gene=At3g02885&sort=all) | | GASA5 | | | **371.4** | 557.0 | 309.0 | 18396.0 | 11028.6 | 2344.3 |
| 171 | [At2g47690](http://atted.jp/cgi-bin/coex_Ath.cgi?gene=At2g47690&sort=all) | | oxidoreductase | | | **371.7** | 453.9 | 1426.7 | 17424.4 | 3028.1 | 9901.8 |
| 172 | [At3g24450](http://atted.jp/cgi-bin/coex_Ath.cgi?gene=At3g24450&sort=all) | | copper-binding | | | **372.7** | 256.0 | 6995.0 | 3146.4 | 6943.1 | 14641.8 |
| 173 | [At3g30340](http://atted.jp/cgi-bin/coex_Ath.cgi?gene=At3g30340&sort=all) | | MtN21 | | | **372.9** | 789.7 | 8810.0 | 2591.9 | 937.3 | 4130.0 |
| 174 | [At2g13820](http://atted.jp/cgi-bin/coex_Ath.cgi?gene=At2g13820&sort=all) | | LTP | | | **375.4** | 882.1 | 291.1 | 4419.1 | 8503.0 | 694.0 |
| 175 | [At1g29520](http://atted.jp/cgi-bin/coex_Ath.cgi?gene=At1g29520&sort=all) | | AWPM-19-like membrane | | | **376.2** | 1062.0 | 2504.6 | 8022.2 | 2629.3 | 7788.3 |
| 176 | [At4g11450](http://atted.jp/cgi-bin/coex_Ath.cgi?gene=At4g11450&sort=all) | |  | | | **376.2** | 752.9 | 2149.5 | 5048.2 | 8217.5 | 3258.0 |
| 177 | [At3g25710](http://atted.jp/cgi-bin/coex_Ath.cgi?gene=At3g25710&sort=all) | | BHLH32 | | | **376.2** | 120.3 | 18216.8 | 17312.3 | 13677.5 | 8374.5 |
| 178 | [At2g32280](http://atted.jp/cgi-bin/coex_Ath.cgi?gene=At2g32280&sort=all) | |  | | | **376.3** | 413.2 | 3453.7 | 17362.3 | 4809.5 | 5324.9 |
| 179 | [At5g58700](http://atted.jp/cgi-bin/coex_Ath.cgi?gene=At5g58700&sort=all) | | PLC4 | | | **376.9** | 143.3 | 5947.0 | 4437.1 | 11546.6 | 14979.3 |
| 180 | [At5g66410](http://atted.jp/cgi-bin/coex_Ath.cgi?gene=At5g66410&sort=all) | | PLP3b | | | **378.9** | 356.4 | 589.6 | 9083.9 | 14149.6 | 15479.3 |
| 181 | [At1g16530](http://atted.jp/cgi-bin/coex_Ath.cgi?gene=At1g16530&sort=all) | | ASL9 | | | **379.0** | 1191.5 | 335.2 | 3850.4 | 10863.6 | 9299.1 |
| 182 | 263063_s_at | |  | | | **379.9** | 565.6 | 3980.1 | 19855.7 | 12537.8 | 6548.1 |
| 183 | [At2g40480](http://atted.jp/cgi-bin/coex_Ath.cgi?gene=At2g40480&sort=all) | |  | | | **381.1** | 198.7 | 1817.2 | 17058.8 | 18000.2 | 9834.7 |
| 184 | [At1g29310](http://atted.jp/cgi-bin/coex_Ath.cgi?gene=At1g29310&sort=all) | | transporter | | | **381.6** | 194.3 | 1021.8 | 17224.5 | 12385.5 | 8573.1 |
| 185 | [At5g43060](http://atted.jp/cgi-bin/coex_Ath.cgi?gene=At5g43060&sort=all) | | protease | | | **383.8** | 41.4 | 13407.2 | 17610.2 | 10629.5 | 2911.9 |
| 186 | [At2g44830](http://atted.jp/cgi-bin/coex_Ath.cgi?gene=At2g44830&sort=all) | | kinase | | | **384.1** | 899.5 | 2480.2 | 3435.1 | 8883.0 | 2499.2 |
| 187 | [At1g01110](http://atted.jp/cgi-bin/coex_Ath.cgi?gene=At1g01110&sort=all) | | IQD18 | | | **384.8** | 1122.0 | 655.1 | 8040.3 | 14478.8 | 14031.6 |
| 188 | [At5g42280](http://atted.jp/cgi-bin/coex_Ath.cgi?gene=At5g42280&sort=all) | | DC1 | | | **385.8** | 1281.5 | 1539.4 | 1854.0 | 5390.1 | 15754.2 |
| 189 | [At2g29660](http://atted.jp/cgi-bin/coex_Ath.cgi?gene=At2g29660&sort=all) | | zinc finger | | | **391.3** | 317.1 | 3911.7 | 15280.1 | 13722.9 | 6818.1 |
| 190 | [At4g11550](http://atted.jp/cgi-bin/coex_Ath.cgi?gene=At4g11550&sort=all) | | DC1 | | | **392.1** | 695.9 | 523.2 | 3819.2 | 9454.2 | 14172.2 |
| 191 | [At1g73780](http://atted.jp/cgi-bin/coex_Ath.cgi?gene=At1g73780&sort=all) | | LTP | | | **393.8** | 1658.2 | 400.9 | 4508.3 | 9666.7 | 15832.7 |
| 192 | [At1g76260](http://atted.jp/cgi-bin/coex_Ath.cgi?gene=At1g76260&sort=all) | | transducin | | | **393.9** | 194.7 | 3005.0 | 13986.7 | 20872.6 | 14240.1 |
| 193 | [261092_at](http://atted.jp/cgi-bin/coex_Ath.cgi?gene=261092_at&sort=all) | |  | | | **398.8** | 1015.1 | 1615.2 | 2653.8 | 4678.7 | 1622.6 |
| 194 | [At1g32930](http://atted.jp/cgi-bin/coex_Ath.cgi?gene=At1g32930&sort=all) | | transferase | | | **402.7** | 1056.8 | 3485.8 | 1062.8 | 4036.0 | 14211.4 |
| 195 | [At1g17560](http://atted.jp/cgi-bin/coex_Ath.cgi?gene=At1g17560&sort=all) | | HLL | | | **402.9** | 763.1 | 1362.6 | 6077.2 | 14426.9 | 4020.5 |
| 196 | [At1g28390](http://atted.jp/cgi-bin/coex_Ath.cgi?gene=At1g28390&sort=all) | | kinase | | | **403.5** | 254.8 | 3045.2 | 14358.4 | 9527.4 | 2837.1 |
| 197 | [At2g30050](http://atted.jp/cgi-bin/coex_Ath.cgi?gene=At2g30050&sort=all) | | transducin | | | **403.8** | 155.2 | 1698.6 | 10730.2 | 21743.3 | 4395.7 |
| 198 | [At2g31270](http://atted.jp/cgi-bin/coex_Ath.cgi?gene=At2g31270&sort=all) | | CDT1A | | | **410.2** | 1049.9 | 672.6 | 7913.1 | 19297.5 | 2513.6 |
| 199 | [At1g64910](http://atted.jp/cgi-bin/coex_Ath.cgi?gene=At1g64910&sort=all) | | transferase | | | **412.1** | 1752.5 | 45.8 | 2568.5 | 3208.6 | 10953.6 |
| 200 | [At1g61840](http://atted.jp/cgi-bin/coex_Ath.cgi?gene=At1g61840&sort=all) | | DC1 | | | **413.0** | 1583.1 | 113.6 | 5354.6 | 4839.2 | 14262.6 |
| 201 | [At5g19600](http://atted.jp/cgi-bin/coex_Ath.cgi?gene=At5g19600&sort=all) | | SULTR3;5 | | | **417.7** | 616.8 | 2887.8 | 5636.1 | 8383.0 | 12945.3 |
| 202 | [At3g03130](http://atted.jp/cgi-bin/coex_Ath.cgi?gene=At3g03130&sort=all) | |  | | | **417.9** | 1172.4 | 354.0 | 5073.1 | 11582.0 | 6895.4 |
| 203 | 252605_s_at | |  | | | **419.8** | 820.4 | 970.9 | 3413.7 | 11870.3 | 15992.0 |
| 204 | [At1g04050](http://atted.jp/cgi-bin/coex_Ath.cgi?gene=At1g04050&sort=all) | | SUVR1 | | | **423.2** | 704.1 | 3470.9 | 7706.6 | 1013.4 | 6126.3 |
| 205 | [At2g24300](http://atted.jp/cgi-bin/coex_Ath.cgi?gene=At2g24300&sort=all) | | calmodulin-binding | | | **423.9** | 500.0 | 1788.3 | 6459.1 | 4568.5 | 15399.3 |
| 206 | [At1g64650](http://atted.jp/cgi-bin/coex_Ath.cgi?gene=At1g64650&sort=all) | |  | | | **426.6** | 732.7 | 1228.9 | 9027.5 | 8976.8 | 11312.8 |
| 207 | [At1g52240](http://atted.jp/cgi-bin/coex_Ath.cgi?gene=At1g52240&sort=all) | | ROPGEF11 | | | **429.4** | 71.0 | 3273.2 | 8430.3 | 4303.4 | 3455.7 |
| 208 | [At5g42630](http://atted.jp/cgi-bin/coex_Ath.cgi?gene=At5g42630&sort=all) | | ATS | | | **429.9** | 292.7 | 6984.0 | 6741.0 | 15006.9 | 3042.7 |
| 209 | [At4g00460](http://atted.jp/cgi-bin/coex_Ath.cgi?gene=At4g00460&sort=all) | | ROPGEF3 | | | **430.4** | 903.4 | 2033.7 | 7589.1 | 16104.8 | 7366.2 |
| 210 | [At4g36180](http://atted.jp/cgi-bin/coex_Ath.cgi?gene=At4g36180&sort=all) | | LRR | | | **432.4** | 1284.4 | 1864.2 | 9746.0 | 16850.4 | 13031.9 |
| 211 | [At2g23530](http://atted.jp/cgi-bin/coex_Ath.cgi?gene=At2g23530&sort=all) | |  | | | **434.3** | 710.1 | 1940.0 | 13084.0 | 12958.2 | 2549.1 |
| 212 | [At2g42250](http://atted.jp/cgi-bin/coex_Ath.cgi?gene=At2g42250&sort=all) | | CYP712A1 | | | **435.5** | 3001.5 | 7.1 | 5124.7 | 2303.6 | 9103.7 |
| 213 | [At1g26850](http://atted.jp/cgi-bin/coex_Ath.cgi?gene=At1g26850&sort=all) | | dehydration-responsive | | | **437.6** | 34.1 | 2023.1 | 10578.0 | 9583.5 | 14531.3 |
| 214 | [At5g63940](http://atted.jp/cgi-bin/coex_Ath.cgi?gene=At5g63940&sort=all) | | kinase | | | **439.6** | 23.8 | 2148.8 | 16296.6 | 21546.7 | 4241.2 |
| 215 | [At5g15150](http://atted.jp/cgi-bin/coex_Ath.cgi?gene=At5g15150&sort=all) | | HB-3 | | | **440.5** | 566.5 | 1426.4 | 4401.3 | 15945.0 | 16805.0 |
| 216 | [At1g72250](http://atted.jp/cgi-bin/coex_Ath.cgi?gene=At1g72250&sort=all) | | kinesin motor | | | **440.6** | 973.1 | 878.2 | 10175.9 | 20921.3 | 9744.3 |
| 217 | [At4g28430](http://atted.jp/cgi-bin/coex_Ath.cgi?gene=At4g28430&sort=all) | | reticulon | | | **441.4** | 393.0 | 3365.6 | 11970.5 | 4471.7 | 9565.9 |
| 218 | [At5g20540](http://atted.jp/cgi-bin/coex_Ath.cgi?gene=At5g20540&sort=all) | | BRXL4 | | | **442.8** | 127.8 | 6435.9 | 9959.0 | 9819.5 | 9217.9 |
| 219 | [At2g16570](http://atted.jp/cgi-bin/coex_Ath.cgi?gene=At2g16570&sort=all) | | ASE1 | | | **443.7** | 1466.2 | 2778.9 | 517.8 | 2693.8 | 2671.8 |
| 220 | [At1g24320](http://atted.jp/cgi-bin/coex_Ath.cgi?gene=At1g24320&sort=all) | | glucosidase | | | **443.8** | 683.2 | 2044.9 | 7088.7 | 7951.4 | 7063.4 |
| 221 | [At2g19970](http://atted.jp/cgi-bin/coex_Ath.cgi?gene=At2g19970&sort=all) | | pathogenesis | | | **447.1** | 1304.8 | 467.9 | 16967.1 | 14985.5 | 16545.4 |
| 222 | [At2g37560](http://atted.jp/cgi-bin/coex_Ath.cgi?gene=At2g37560&sort=all) | | ORC2 | | | **450.5** | 1587.4 | 410.5 | 10508.6 | 2662.2 | 5509.4 |
| 223 | [At1g65180](http://atted.jp/cgi-bin/coex_Ath.cgi?gene=At1g65180&sort=all) | | DC1 | | | **450.9** | 498.5 | 1369.6 | 10579.6 | 5854.5 | 10769.1 |
| 224 | [At5g27450](http://atted.jp/cgi-bin/coex_Ath.cgi?gene=At5g27450&sort=all) | | MK | | | **455.1** | 240.0 | 4469.9 | 9024.0 | 14883.9 | 2911.7 |
| 225 | [At5g41060](http://atted.jp/cgi-bin/coex_Ath.cgi?gene=At5g41060&sort=all) | | zinc finger | | | **461.1** | 928.2 | 2420.8 | 4991.8 | 9534.4 | 5819.0 |
| 226 | [At5g07810](http://atted.jp/cgi-bin/coex_Ath.cgi?gene=At5g07810&sort=all) | | nuclease | | | **461.6** | 1231.1 | 2900.2 | 5299.0 | 9159.1 | 3072.5 |
| 227 | [At3g50150](http://atted.jp/cgi-bin/coex_Ath.cgi?gene=At3g50150&sort=all) | |  | | | **469.9** | 4696.3 | 189.3 | 1751.9 | 1902.4 | 962.2 |
| 228 | [At1g49910](http://atted.jp/cgi-bin/coex_Ath.cgi?gene=At1g49910&sort=all) | | WD-40 repeat | | | **470.1** | 874.7 | 7256.6 | 4165.4 | 2957.0 | 4096.8 |
| 229 | [At3g51740](http://atted.jp/cgi-bin/coex_Ath.cgi?gene=At3g51740&sort=all) | | IMK2 | | | **472.8** | 973.8 | 945.0 | 5473.4 | 18929.5 | 18687.4 |
| 230 | [At5g13840](http://atted.jp/cgi-bin/coex_Ath.cgi?gene=At5g13840&sort=all) | | FZR3 | | | **474.4** | 756.0 | 766.6 | 11434.3 | 21411.1 | 12870.9 |
| 231 | [At2g43040](http://atted.jp/cgi-bin/coex_Ath.cgi?gene=At2g43040&sort=all) | | NPG1 | | | **476.2** | 1472.2 | 3857.9 | 1625.1 | 7849.9 | 3452.1 |
| 232 | [At1g02690](http://atted.jp/cgi-bin/coex_Ath.cgi?gene=At1g02690&sort=all) | | IMPA-6 | | | **477.3** | 807.1 | 594.0 | 18232.4 | 18471.8 | 6103.6 |
| 233 | [At1g28290](http://atted.jp/cgi-bin/coex_Ath.cgi?gene=At1g28290&sort=all) | | AGP31 | | | **483.2** | 886.5 | 3833.8 | 5427.1 | 10060.0 | 16959.2 |
| 234 | [At1g44740](http://atted.jp/cgi-bin/coex_Ath.cgi?gene=At1g44740&sort=all) | |  | | | **484.6** | 1138.0 | 1281.2 | 3530.0 | 5787.8 | 6694.7 |
| 235 | [At5g50120](http://atted.jp/cgi-bin/coex_Ath.cgi?gene=At5g50120&sort=all) | | transducin | | | **485.0** | 672.3 | 2992.3 | 11578.1 | 20399.3 | 12897.0 |
| 236 | [At5g38110](http://atted.jp/cgi-bin/coex_Ath.cgi?gene=At5g38110&sort=all) | | ASF1B | | | **485.1** | 1055.0 | 1152.2 | 6526.1 | 9435.3 | 10592.9 |
| 237 | [At5g20885](http://atted.jp/cgi-bin/coex_Ath.cgi?gene=At5g20885&sort=all) | | zinc finger | | | **487.3** | 675.8 | 803.6 | 8784.1 | 8798.4 | 451.4 |
| 238 | [At1g15640](http://atted.jp/cgi-bin/coex_Ath.cgi?gene=At1g15640&sort=all) | |  | | | **487.3** | 2449.3 | 331.3 | 26.8 | 1467.0 | 9177.5 |
| 239 | [At5g65650](http://atted.jp/cgi-bin/coex_Ath.cgi?gene=At5g65650&sort=all) | |  | | | **489.2** | 95.5 | 2808.2 | 11195.8 | 19353.9 | 5787.9 |
| 240 | [At4g08150](http://atted.jp/cgi-bin/coex_Ath.cgi?gene=At4g08150&sort=all) | | KNAT1 | | | **491.0** | 1013.9 | 3169.8 | 11108.0 | 8196.0 | 7279.5 |
| 241 | [At5g48600](http://atted.jp/cgi-bin/coex_Ath.cgi?gene=At5g48600&sort=all) | | SMC3 | | | **493.5** | 1380.2 | 816.1 | 6069.2 | 3220.3 | 4455.1 |
| 242 | [At4g22212](http://atted.jp/cgi-bin/coex_Ath.cgi?gene=At4g22212&sort=all) | |  | | | **494.3** | 713.5 | 747.4 | 8840.0 | 18122.2 | 10918.7 |
| 243 | [At2g34830](http://atted.jp/cgi-bin/coex_Ath.cgi?gene=At2g34830&sort=all) | | WRKY35 | | | **496.4** | 858.8 | 2273.9 | 7482.0 | 4278.0 | 11961.0 |
| 244 | [At1g48750](http://atted.jp/cgi-bin/coex_Ath.cgi?gene=At1g48750&sort=all) | | LTP | | | **497.4** | 272.4 | 6944.2 | 14996.7 | 11229.9 | 13466.6 |
| 245 | [At3g14890](http://atted.jp/cgi-bin/coex_Ath.cgi?gene=At3g14890&sort=all) | | phosphoesterase | | | **501.3** | 544.5 | 1984.0 | 6528.8 | 12032.1 | 6677.3 |
| 246 | [At5g66560](http://atted.jp/cgi-bin/coex_Ath.cgi?gene=At5g66560&sort=all) | | NPH3 | | | **502.5** | 1097.9 | 3220.1 | 9888.7 | 7766.0 | 5904.6 |
| 247 | [At5g05510](http://atted.jp/cgi-bin/coex_Ath.cgi?gene=At5g05510&sort=all) | | kinase | | | **502.9** | 1007.3 | 2195.4 | 5237.3 | 4106.6 | 12775.6 |
| 248 | [At3g17780](http://atted.jp/cgi-bin/coex_Ath.cgi?gene=At3g17780&sort=all) | |  | | | **504.5** | 606.9 | 793.6 | 16215.3 | 16400.4 | 12682.5 |
| 249 | [At2g29750](http://atted.jp/cgi-bin/coex_Ath.cgi?gene=At2g29750&sort=all) | | UGT71C1 | | | **504.9** | 1079.3 | 1107.7 | 9558.1 | 10548.2 | 13571.5 |
| 250 | [At2g34530](http://atted.jp/cgi-bin/coex_Ath.cgi?gene=At2g34530&sort=all) | |  | | | **506.2** | 2558.7 | 241.8 | 10433.4 | 4271.9 | 22611.4 |
| 251 | [At2g18030](http://atted.jp/cgi-bin/coex_Ath.cgi?gene=At2g18030&sort=all) | | reductase | | | **506.6** | 124.9 | 654.7 | 13099.6 | 14811.7 | 11341.2 |
| 252 | [At2g03090](http://atted.jp/cgi-bin/coex_Ath.cgi?gene=At2g03090&sort=all) | | EXPA15 | | | **506.8** | 668.5 | 2177.5 | 5161.4 | 15262.3 | 2531.9 |
| 253 | [At2g20515](http://atted.jp/cgi-bin/coex_Ath.cgi?gene=At2g20515&sort=all) | |  | | | **507.1** | 1836.5 | 617.2 | 12390.0 | 14815.6 | 9270.0 |
| 254 | [At5g47770](http://atted.jp/cgi-bin/coex_Ath.cgi?gene=At5g47770&sort=all) | | FPS1 | | | **508.1** | 844.5 | 2291.3 | 7042.9 | 14617.3 | 6200.5 |
| 255 | [At2g23300](http://atted.jp/cgi-bin/coex_Ath.cgi?gene=At2g23300&sort=all) | | kinase | | | **508.6** | 403.1 | 5367.3 | 10898.0 | 7944.7 | 7697.9 |
| 256 | [At3g63300](http://atted.jp/cgi-bin/coex_Ath.cgi?gene=At3g63300&sort=all) | | phosphoinositide binding | | | **510.1** | 1392.5 | 1397.2 | 6015.3 | 5214.3 | 1586.8 |
| 257 | [At3g03200](http://atted.jp/cgi-bin/coex_Ath.cgi?gene=At3g03200&sort=all) | | anac045 | | | **514.2** | 1712.1 | 986.8 | 9289.9 | 6067.9 | 17424.9 |
| 258 | [At5g07800](http://atted.jp/cgi-bin/coex_Ath.cgi?gene=At5g07800&sort=all) | | FMO | | | **515.0** | 479.4 | 17104.0 | 4200.3 | 2834.7 | 7909.4 |
| 259 | [At2g45200](http://atted.jp/cgi-bin/coex_Ath.cgi?gene=At2g45200&sort=all) | | GOS12 | | | **515.3** | 468.8 | 2341.1 | 12962.5 | 12538.3 | 10311.5 |
| 260 | [At4g20270](http://atted.jp/cgi-bin/coex_Ath.cgi?gene=At4g20270&sort=all) | | BAM3 | | | **516.5** | 234.8 | 5321.3 | 17169.1 | 16878.6 | 9009.4 |
| 261 | [At4g14200](http://atted.jp/cgi-bin/coex_Ath.cgi?gene=At4g14200&sort=all) | |  | | | **516.5** | 1686.0 | 1170.1 | 3429.7 | 7323.4 | 11834.5 |
| 262 | [At5g56720](http://atted.jp/cgi-bin/coex_Ath.cgi?gene=At5g56720&sort=all) | | malate dehydrogenase | | | **519.9** | 2395.7 | 701.7 | 1135.8 | 3311.1 | 17722.8 |
| 263 | [At1g63640](http://atted.jp/cgi-bin/coex_Ath.cgi?gene=At1g63640&sort=all) | | kinesin motor | | | **528.0** | 1265.9 | 2177.2 | 9991.2 | 8683.4 | 17665.8 |
| 264 | [At5g60930](http://atted.jp/cgi-bin/coex_Ath.cgi?gene=At5g60930&sort=all) | | chromosome-associated kinesin | | | **529.9** | 765.8 | 1288.7 | 6599.9 | 21983.5 | 9042.6 |
| 265 | [At1g18040](http://atted.jp/cgi-bin/coex_Ath.cgi?gene=At1g18040&sort=all) | | CDKD1;3 | | | **531.5** | 891.3 | 1074.7 | 7522.0 | 16054.4 | 6226.5 |
| 266 | [At5g65970](http://atted.jp/cgi-bin/coex_Ath.cgi?gene=At5g65970&sort=all) | | MLO10 | | | **531.7** | 1022.0 | 3283.6 | 4074.7 | 10155.3 | 3716.4 |
| 267 | [At2g04800](http://atted.jp/cgi-bin/coex_Ath.cgi?gene=At2g04800&sort=all) | |  | | | **531.7** | 1240.8 | 4286.2 | 855.4 | 7780.4 | 15282.2 |
| 268 | [At2g27970](http://atted.jp/cgi-bin/coex_Ath.cgi?gene=At2g27970&sort=all) | | CKS2 | | | **533.0** | 539.0 | 2832.3 | 13825.0 | 13932.8 | 11849.5 |
| 269 | [At1g09000](http://atted.jp/cgi-bin/coex_Ath.cgi?gene=At1g09000&sort=all) | | ANP1 | | | **533.3** | 594.9 | 7191.0 | 2820.3 | 5895.4 | 4302.0 |
| 270 | [At3g24110](http://atted.jp/cgi-bin/coex_Ath.cgi?gene=At3g24110&sort=all) | | calcium-binding EF hand | | | **534.3** | 1541.1 | 2691.1 | 2332.2 | 2204.7 | 18575.6 |
| 271 | [At2g23700](http://atted.jp/cgi-bin/coex_Ath.cgi?gene=At2g23700&sort=all) | |  | | | **534.7** | 409.9 | 5886.2 | 12307.7 | 16114.6 | 5253.1 |
| 272 | [At3g25190](http://atted.jp/cgi-bin/coex_Ath.cgi?gene=At3g25190&sort=all) | | nodulin | | | **535.0** | 1257.7 | 2102.8 | 5631.5 | 7279.2 | 15259.1 |
| 273 | [At5g12270](http://atted.jp/cgi-bin/coex_Ath.cgi?gene=At5g12270&sort=all) | | oxygenase | | | **535.5** | 685.6 | 4466.5 | 5932.3 | 2441.5 | 4795.5 |
| 274 | [At5g15070](http://atted.jp/cgi-bin/coex_Ath.cgi?gene=At5g15070&sort=all) | | oxidoreductase | | | **535.5** | 1150.7 | 3085.2 | 3881.3 | 1611.2 | 12972.9 |
| 275 | [At2g18060](http://atted.jp/cgi-bin/coex_Ath.cgi?gene=At2g18060&sort=all) | | VND1 | | | **535.5** | 7.8 | 17535.9 | 4087.0 | 2294.3 | 7431.5 |
| 276 | [At5g55830](http://atted.jp/cgi-bin/coex_Ath.cgi?gene=At5g55830&sort=all) | | kinase | | | **535.7** | 908.1 | 1192.3 | 6848.4 | 13048.2 | 6409.3 |
| 277 | [At5g50820](http://atted.jp/cgi-bin/coex_Ath.cgi?gene=At5g50820&sort=all) | | anac097 | | | **536.0** | 400.7 | 9265.3 | 6251.0 | 4150.0 | 12429.2 |
| 278 | [At1g60860](http://atted.jp/cgi-bin/coex_Ath.cgi?gene=At1g60860&sort=all) | | AGD2 | | | **539.0** | 459.3 | 7502.8 | 12564.4 | 17345.2 | 14263.7 |
| 279 | [At5g62550](http://atted.jp/cgi-bin/coex_Ath.cgi?gene=At5g62550&sort=all) | |  | | | **540.5** | 645.4 | 1439.2 | 14583.2 | 19805.2 | 19293.4 |
| 280 | [At5g65420](http://atted.jp/cgi-bin/coex_Ath.cgi?gene=At5g65420&sort=all) | | CYCD4;1 | | | **544.5** | 409.4 | 6248.4 | 3857.3 | 10425.5 | 12201.7 |
| 281 | [At3g27330](http://atted.jp/cgi-bin/coex_Ath.cgi?gene=At3g27330&sort=all) | | zinc finger | | | **544.7** | 1234.9 | 1000.8 | 3207.2 | 17642.4 | 8918.5 |
| 282 | [At5g01890](http://atted.jp/cgi-bin/coex_Ath.cgi?gene=At5g01890&sort=all) | | kinase | | | **546.0** | 1066.5 | 2490.8 | 8862.7 | 10766.0 | 2926.6 |
| 283 | [At4g22120](http://atted.jp/cgi-bin/coex_Ath.cgi?gene=At4g22120&sort=all) | | ERD | | | **549.4** | 588.4 | 4133.0 | 10803.6 | 12075.3 | 7672.0 |
| 284 | [At3g54110](http://atted.jp/cgi-bin/coex_Ath.cgi?gene=At3g54110&sort=all) | | PUMP1 | | | **553.2** | 130.7 | 3141.1 | 13381.6 | 15285.7 | 10820.5 |
| 285 | [At1g60810](http://atted.jp/cgi-bin/coex_Ath.cgi?gene=At1g60810&sort=all) | | ACLA-2 | | | **558.0** | 1031.8 | 1940.5 | 15028.8 | 12210.2 | 6632.8 |
| 286 | [At3g52900](http://atted.jp/cgi-bin/coex_Ath.cgi?gene=At3g52900&sort=all) | |  | | | **561.8** | 523.9 | 7113.3 | 8902.9 | 17367.0 | 18820.9 |
| 287 | [At2g22900](http://atted.jp/cgi-bin/coex_Ath.cgi?gene=At2g22900&sort=all) | | GMA12/MNN10 | | | **563.4** | 895.8 | 1673.3 | 4410.7 | 14940.2 | 9952.4 |
| 288 | [At3g01220](http://atted.jp/cgi-bin/coex_Ath.cgi?gene=At3g01220&sort=all) | | ATHB20 | | | **563.7** | 1354.1 | 1067.8 | 8705.8 | 14555.9 | 16397.2 |
| 289 | [At5g24330](http://atted.jp/cgi-bin/coex_Ath.cgi?gene=At5g24330&sort=all) | | ATXR6 | | | **564.3** | 1804.8 | 524.5 | 2743.5 | 8898.5 | 11039.4 |
| 290 | [At5g20550](http://atted.jp/cgi-bin/coex_Ath.cgi?gene=At5g20550&sort=all) | | oxygenase | | | **564.5** | 1268.5 | 2625.3 | 3031.0 | 10908.0 | 13544.8 |
| 291 | [At3g12600](http://atted.jp/cgi-bin/coex_Ath.cgi?gene=At3g12600&sort=all) | | atnudt16 | | | **569.2** | 251.0 | 4072.0 | 12012.0 | 16114.5 | 10848.4 |
| 292 | [At1g01200](http://atted.jp/cgi-bin/coex_Ath.cgi?gene=At1g01200&sort=all) | | RABA3 | | | **569.3** | 1675.3 | 599.7 | 6432.1 | 8165.6 | 11160.1 |
| 293 | [At3g51480](http://atted.jp/cgi-bin/coex_Ath.cgi?gene=At3g51480&sort=all) | | GLR3.6 | | | **570.1** | 795.8 | 8862.8 | 593.3 | 3995.2 | 1334.8 |
| 294 | [At2g46590](http://atted.jp/cgi-bin/coex_Ath.cgi?gene=At2g46590&sort=all) | | DAG2 | | | **571.3** | 62.9 | 17849.6 | 2273.2 | 6145.8 | 14514.0 |
| 295 | [At1g70470](http://atted.jp/cgi-bin/coex_Ath.cgi?gene=At1g70470&sort=all) | |  | | | **571.8** | 507.6 | 4781.9 | 7234.9 | 14773.4 | 4771.0 |
| 296 | [At1g79820](http://atted.jp/cgi-bin/coex_Ath.cgi?gene=At1g79820&sort=all) | | SGB1 | | | **573.1** | 336.1 | 17483.1 | 2940.3 | 7867.8 | 12489.0 |
| 297 | [At5g62340](http://atted.jp/cgi-bin/coex_Ath.cgi?gene=At5g62340&sort=all) | | inhibitor | | | **573.9** | 1698.4 | 390.0 | 690.8 | 10278.0 | 8114.5 |
| 298 | [At3g61850](http://atted.jp/cgi-bin/coex_Ath.cgi?gene=At3g61850&sort=all) | | DAG1 | | | **574.5** | 546.4 | 9390.6 | 3462.0 | 9214.4 | 5623.3 |
| 299 | [At1g34065](http://atted.jp/cgi-bin/coex_Ath.cgi?gene=At1g34065&sort=all) | | SAMC2 | | | **574.6** | 1095.7 | 1782.9 | 8113.0 | 3420.3 | 6130.6 |
| 300 | [248324_at](http://atted.jp/cgi-bin/coex_Ath.cgi?gene=248324_at&sort=all) | |  | | | **576.2** | 2051.1 | 987.5 | 2709.3 | 2175.4 | 1320.1 |
|  | | | | | | | | | | | |
| **300 coexpressed gene with At5g24620** | | | | | | | | | | | |
|  | **locus** | | **Short description** | | | **MR**  **(all)** | **MR**  **(tissue)** | **MR**  **(abiotic)** | **MR**  **(biotic)** | **MR**  **(hormone)** | **MR**  **(light)** |
| 1 | [265974_at](http://atted.jp/cgi-bin/coex_Ath.cgi?gene=265974_at&sort=all) | |  | | | **2.0** | 24.3 | 103.7 | 684.0 | 1.7 | 84.8 |
| 2 | [At2g39280](http://atted.jp/cgi-bin/coex_Ath.cgi?gene=At2g39280&sort=all) | | RAB GTPase activator | | | **6.2** | 7.9 | 110.2 | 1766.0 | 39.5 | 608.8 |
| 3 | [At3g19960](http://atted.jp/cgi-bin/coex_Ath.cgi?gene=At3g19960&sort=all) | | ATM1 | | | **16.0** | 22.7 | 303.0 | 240.9 | 961.0 | 456.9 |
| 4 | [At1g08660](http://atted.jp/cgi-bin/coex_Ath.cgi?gene=At1g08660&sort=all) | | transferase | | | **20.2** | 94.8 | 136.8 | 1453.0 | 10.2 | 8341.4 |
| 5 | 263447_s_at | |  | | | **22.1** | 351.0 | 134.2 | 2701.2 | 28.3 | 438.6 |
| 6 | [At1g51690](http://atted.jp/cgi-bin/coex_Ath.cgi?gene=At1g51690&sort=all) | | B ALPHA | | | **24.2** | 4.2 | 7004.4 | 4228.9 | 99.2 | 435.1 |
| 7 | [At4g38120](http://atted.jp/cgi-bin/coex_Ath.cgi?gene=At4g38120&sort=all) | | binding | | | **24.5** | 67.2 | 678.4 | 704.0 | 15.3 | 6263.1 |
| 8 | [At5g22700](http://atted.jp/cgi-bin/coex_Ath.cgi?gene=At5g22700&sort=all) | | F-box | | | **32.8** | 1.4 | 1417.2 | 60.2 | 2748.4 | 182.7 |
| 9 | [At2g01130](http://atted.jp/cgi-bin/coex_Ath.cgi?gene=At2g01130&sort=all) | | helicase | | | **33.7** | 466.9 | 3.5 | 1203.6 | 138.7 | 1245.3 |
| 10 | [At1g24764](http://atted.jp/cgi-bin/coex_Ath.cgi?gene=At1g24764&sort=all) | | MAP70-2 | | | **38.8** | 205.7 | 488.4 | 1426.6 | 903.8 | 482.0 |
| 11 | [266472_at](http://atted.jp/cgi-bin/coex_Ath.cgi?gene=266472_at&sort=all) | |  | | | **40.6** | 153.5 | 284.3 | 1612.2 | 112.5 | 1978.4 |
| 12 | [At5g07630](http://atted.jp/cgi-bin/coex_Ath.cgi?gene=At5g07630&sort=all) | | transporter | | | **43.1** | 150.8 | 648.4 | 627.0 | 12.7 | 17.7 |
| 13 | [At5g04560](http://atted.jp/cgi-bin/coex_Ath.cgi?gene=At5g04560&sort=all) | | DME | | | **43.4** | 120.7 | 35.3 | 386.8 | 2081.3 | 5653.9 |
| 14 | 258186_s_at | |  | | | **43.6** | 381.0 | 147.0 | 11015.0 | 383.1 | 2712.2 |
| 15 | [At3g12290](http://atted.jp/cgi-bin/coex_Ath.cgi?gene=At3g12290&sort=all) | | hydrolase | | | **44.1** | 125.4 | 280.6 | 860.5 | 5814.1 | 9207.0 |
| 16 | [At3g55020](http://atted.jp/cgi-bin/coex_Ath.cgi?gene=At3g55020&sort=all) | | RabGAP | | | **44.7** | 13.1 | 440.7 | 4791.1 | 100.3 | 8.1 |
| 17 | [At4g32360](http://atted.jp/cgi-bin/coex_Ath.cgi?gene=At4g32360&sort=all) | | reductase | | | **45.2** | 34.9 | 601.0 | 1668.6 | 957.8 | 133.0 |
| 18 | [At5g58200](http://atted.jp/cgi-bin/coex_Ath.cgi?gene=At5g58200&sort=all) | |  | | | **46.2** | 375.5 | 1881.0 | 2478.2 | 29.2 | 326.6 |
| 19 | [At3g57170](http://atted.jp/cgi-bin/coex_Ath.cgi?gene=At3g57170&sort=all) | | Gpi1 | | | **48.2** | 1495.0 | 159.4 | 770.0 | 1539.0 | 9764.0 |
| 20 | [At5g22710](http://atted.jp/cgi-bin/coex_Ath.cgi?gene=At5g22710&sort=all) | |  | | | **48.4** | 13.4 | 2199.9 | 1100.3 | 293.5 | 5650.6 |
| 21 | 247273_at | |  | | | **49.0** | 24.7 | 1080.2 | 782.2 | 1305.8 | 3421.7 |
| 22 | [At3g22190](http://atted.jp/cgi-bin/coex_Ath.cgi?gene=At3g22190&sort=all) | | IQD5 | | | **49.1** | 309.8 | 398.1 | 1154.8 | 1856.9 | 35.6 |
| 23 | 255216_s_at | |  | | | **49.9** | 313.6 | 284.6 | 9678.3 | 1308.4 | 438.7 |
| 24 | [At1g20010](http://atted.jp/cgi-bin/coex_Ath.cgi?gene=At1g20010&sort=all) | | TUB5 | | | **53.0** | 97.0 | 294.5 | 259.1 | 8543.1 | 15233.8 |
| 25 | [At5g42080](http://atted.jp/cgi-bin/coex_Ath.cgi?gene=At5g42080&sort=all) | | ADL1 | | | **53.1** | 278.1 | 148.6 | 8893.0 | 218.4 | 2361.6 |
| 26 | [At5g63810](http://atted.jp/cgi-bin/coex_Ath.cgi?gene=At5g63810&sort=all) | | BGAL10 | | | **54.4** | 44.2 | 237.8 | 106.8 | 3312.8 | 15179.9 |
| 27 | [At3g03380](http://atted.jp/cgi-bin/coex_Ath.cgi?gene=At3g03380&sort=all) | | DegP7 | | | **56.1** | 167.1 | 225.8 | 5321.7 | 71.9 | 576.9 |
| 28 | [At4g00340](http://atted.jp/cgi-bin/coex_Ath.cgi?gene=At4g00340&sort=all) | | RLK4 | | | **59.6** | 89.4 | 44.8 | 387.4 | 3764.6 | 428.0 |
| 29 | [At4g03205](http://atted.jp/cgi-bin/coex_Ath.cgi?gene=At4g03205&sort=all) | | hemf2 | | | **63.2** | 568.8 | 556.5 | 1712.1 | 474.4 | 8737.0 |
| 30 | [At1g73760](http://atted.jp/cgi-bin/coex_Ath.cgi?gene=At1g73760&sort=all) | | zinc finger | | | **65.8** | 66.0 | 729.3 | 3880.1 | 7622.0 | 112.1 |
| 31 | [At2g26900](http://atted.jp/cgi-bin/coex_Ath.cgi?gene=At2g26900&sort=all) | | bile acid:sodium symporter | | | **67.6** | 176.3 | 181.8 | 267.8 | 12764.3 | 7486.3 |
| 32 | [At2g07360](http://atted.jp/cgi-bin/coex_Ath.cgi?gene=At2g07360&sort=all) | | SH3 | | | **68.7** | 226.9 | 98.3 | 3039.9 | 101.9 | 143.8 |
| 33 | [At4g26130](http://atted.jp/cgi-bin/coex_Ath.cgi?gene=At4g26130&sort=all) | |  | | | **69.4** | 105.0 | 268.4 | 610.4 | 8478.6 | 3312.6 |
| 34 | [At5g50000](http://atted.jp/cgi-bin/coex_Ath.cgi?gene=At5g50000&sort=all) | | kinase | | | **70.5** | 43.6 | 2792.1 | 1597.5 | 496.2 | 15236.3 |
| 35 | [At1g48360](http://atted.jp/cgi-bin/coex_Ath.cgi?gene=At1g48360&sort=all) | | hydrolase | | | **74.8** | 701.6 | 630.6 | 4279.2 | 4383.3 | 2181.8 |
| 36 | [At5g42620](http://atted.jp/cgi-bin/coex_Ath.cgi?gene=At5g42620&sort=all) | | zinc ion binding | | | **76.5** | 27.0 | 1488.1 | 1394.2 | 1799.0 | 567.7 |
| 37 | [At1g23080](http://atted.jp/cgi-bin/coex_Ath.cgi?gene=At1g23080&sort=all) | | PIN7 | | | **77.2** | 528.4 | 33.9 | 763.7 | 2403.4 | 7253.0 |
| 38 | [At5g49840](http://atted.jp/cgi-bin/coex_Ath.cgi?gene=At5g49840&sort=all) | | ATPase | | | **78.0** | 884.7 | 1261.3 | 745.1 | 142.9 | 1007.1 |
| 39 | [At5g62000](http://atted.jp/cgi-bin/coex_Ath.cgi?gene=At5g62000&sort=all) | | ARF2 | | | **81.8** | 183.1 | 575.2 | 9986.4 | 72.7 | 69.4 |
| 40 | [256419_at](http://atted.jp/cgi-bin/coex_Ath.cgi?gene=256419_at&sort=all) | |  | | | **82.8** | 150.8 | 374.7 | 2459.3 | 1545.1 | 1298.8 |
| 41 | [At3g33530](http://atted.jp/cgi-bin/coex_Ath.cgi?gene=At3g33530&sort=all) | | transducin | | | **83.0** | 1085.3 | 181.4 | 6757.3 | 8.7 | 1470.8 |
| 42 | [At1g75800](http://atted.jp/cgi-bin/coex_Ath.cgi?gene=At1g75800&sort=all) | | pathogenesis-related thaumatin | | | **85.9** | 229.6 | 163.0 | 2615.2 | 5607.3 | 552.3 |
| 43 | [At1g76660](http://atted.jp/cgi-bin/coex_Ath.cgi?gene=At1g76660&sort=all) | |  | | | **86.8** | 1148.5 | 20.5 | 2995.3 | 1593.8 | 4741.0 |
| 44 | [At2g25760](http://atted.jp/cgi-bin/coex_Ath.cgi?gene=At2g25760&sort=all) | | kinase | | | **89.0** | 436.4 | 395.0 | 4677.8 | 10.9 | 172.3 |
| 45 | [At5g64860](http://atted.jp/cgi-bin/coex_Ath.cgi?gene=At5g64860&sort=all) | | DPE1 | | | **94.2** | 878.1 | 123.3 | 38.3 | 11232.9 | 15393.5 |
| 46 | [At4g25970](http://atted.jp/cgi-bin/coex_Ath.cgi?gene=At4g25970&sort=all) | | PSD3 | | | **95.3** | 262.2 | 122.9 | 1143.1 | 7404.5 | 7875.0 |
| 47 | [At5g24610](http://atted.jp/cgi-bin/coex_Ath.cgi?gene=At5g24610&sort=all) | |  | | | **100.3** | 5878.0 | 1.0 | 131.3 | 4373.2 | 9599.0 |
| 48 | [At3g57470](http://atted.jp/cgi-bin/coex_Ath.cgi?gene=At3g57470&sort=all) | | insulinase | | | **100.8** | 270.0 | 326.0 | 1400.5 | 1.4 | 2897.7 |
| 49 | [At3g21060](http://atted.jp/cgi-bin/coex_Ath.cgi?gene=At3g21060&sort=all) | | transducin | | | **103.8** | 316.4 | 8.5 | 839.4 | 10213.3 | 6857.0 |
| 50 | [251348_at](http://atted.jp/cgi-bin/coex_Ath.cgi?gene=251348_at&sort=all) | |  | | | **104.3** | 990.0 | 153.8 | 296.9 | 28.8 | 1034.4 |
| 51 | [At4g13640](http://atted.jp/cgi-bin/coex_Ath.cgi?gene=At4g13640&sort=all) | | UNE16 | | | **105.0** | 749.2 | 226.6 | 384.0 | 3150.8 | 17478.1 |
| 52 | [At4g36690](http://atted.jp/cgi-bin/coex_Ath.cgi?gene=At4g36690&sort=all) | | U2AF65A | | | **105.3** | 1054.3 | 1387.9 | 818.6 | 44.5 | 1399.2 |
| 53 | [At4g00800](http://atted.jp/cgi-bin/coex_Ath.cgi?gene=At4g00800&sort=all) | | binding | | | **105.5** | 431.7 | 173.8 | 1112.1 | 293.0 | 1213.0 |
| 54 | [At5g12440](http://atted.jp/cgi-bin/coex_Ath.cgi?gene=At5g12440&sort=all) | | zinc ion binding | | | **110.6** | 291.9 | 291.7 | 1036.7 | 7737.5 | 12868.4 |
| 55 | [At4g02430](http://atted.jp/cgi-bin/coex_Ath.cgi?gene=At4g02430&sort=all) | | SR1 | | | **111.8** | 54.1 | 2799.9 | 106.6 | 31.6 | 1663.4 |
| 56 | [At4g04970](http://atted.jp/cgi-bin/coex_Ath.cgi?gene=At4g04970&sort=all) | | GSL1 | | | **113.2** | 2659.3 | 27.0 | 1451.3 | 1132.7 | 2394.4 |
| 57 | [At1g27520](http://atted.jp/cgi-bin/coex_Ath.cgi?gene=At1g27520&sort=all) | | hydrolase | | | **113.8** | 35.4 | 2517.1 | 1827.5 | 87.9 | 847.1 |
| 58 | [At1g60800](http://atted.jp/cgi-bin/coex_Ath.cgi?gene=At1g60800&sort=all) | | NIK3 | | | **114.2** | 33.5 | 3039.9 | 248.9 | 8168.5 | 3255.8 |
| 59 | [At1g03160](http://atted.jp/cgi-bin/coex_Ath.cgi?gene=At1g03160&sort=all) | | FZL | | | **115.8** | 1383.9 | 7.8 | 480.2 | 1422.7 | 654.4 |
| 60 | [At2g21380](http://atted.jp/cgi-bin/coex_Ath.cgi?gene=At2g21380&sort=all) | | kinesin motor | | | **118.4** | 1171.8 | 406.7 | 194.7 | 321.7 | 8338.7 |
| 61 | [At1g65960](http://atted.jp/cgi-bin/coex_Ath.cgi?gene=At1g65960&sort=all) | | GAD2 | | | **119.0** | 140.0 | 3409.4 | 1117.1 | 395.1 | 7043.8 |
| 62 | [At5g18590](http://atted.jp/cgi-bin/coex_Ath.cgi?gene=At5g18590&sort=all) | | kelch repeat | | | **120.6** | 207.3 | 40.1 | 815.8 | 9088.7 | 6158.3 |
| 63 | [At3g29320](http://atted.jp/cgi-bin/coex_Ath.cgi?gene=At3g29320&sort=all) | | phosphorylase | | | **122.3** | 947.7 | 88.9 | 192.6 | 17382.3 | 10675.7 |
| 64 | 245764_s_at | |  | | | **122.8** | 455.5 | 452.5 | 3769.4 | 17.9 | 1002.1 |
| 65 | [At3g61010](http://atted.jp/cgi-bin/coex_Ath.cgi?gene=At3g61010&sort=all) | | hydrolase | | | **122.9** | 795.9 | 294.8 | 335.4 | 23.8 | 727.8 |
| 66 | [At2g01220](http://atted.jp/cgi-bin/coex_Ath.cgi?gene=At2g01220&sort=all) | | transferase | | | **123.7** | 184.0 | 1767.8 | 2043.2 | 372.7 | 65.3 |
| 67 | [At2g21660](http://atted.jp/cgi-bin/coex_Ath.cgi?gene=At2g21660&sort=all) | | CCR2 | | | **123.8** | 6.3 | 231.3 | 156.3 | 2009.8 | 13554.0 |
| 68 | [At1g55350](http://atted.jp/cgi-bin/coex_Ath.cgi?gene=At1g55350&sort=all) | | DEK1 | | | **124.3** | 422.7 | 977.4 | 652.9 | 1645.2 | 426.1 |
| 69 | [At5g15230](http://atted.jp/cgi-bin/coex_Ath.cgi?gene=At5g15230&sort=all) | | GASA4 | | | **124.9** | 190.1 | 220.3 | 1526.5 | 17488.9 | 16523.0 |
| 70 | [At3g15160](http://atted.jp/cgi-bin/coex_Ath.cgi?gene=At3g15160&sort=all) | |  | | | **133.9** | 494.9 | 231.7 | 3392.2 | 4634.5 | 9933.5 |
| 71 | [At1g10200](http://atted.jp/cgi-bin/coex_Ath.cgi?gene=At1g10200&sort=all) | | WLIM1 | | | **134.7** | 331.2 | 302.6 | 266.7 | 15157.6 | 8742.4 |
| 72 | [At3g11040](http://atted.jp/cgi-bin/coex_Ath.cgi?gene=At3g11040&sort=all) | | hydrolase | | | **136.0** | 313.4 | 670.4 | 4199.5 | 954.1 | 14290.6 |
| 73 | [At2g45810](http://atted.jp/cgi-bin/coex_Ath.cgi?gene=At2g45810&sort=all) | | DEAD | | | **136.5** | 209.5 | 1748.3 | 1174.9 | 171.2 | 2915.4 |
| 74 | [At5g14920](http://atted.jp/cgi-bin/coex_Ath.cgi?gene=At5g14920&sort=all) | | gibberellin-regulated | | | **141.8** | 420.0 | 423.1 | 714.8 | 14742.7 | 18558.6 |
| 75 | [At4g16060](http://atted.jp/cgi-bin/coex_Ath.cgi?gene=At4g16060&sort=all) | |  | | | **145.7** | 325.2 | 648.8 | 3028.6 | 1548.5 | 2587.3 |
| 76 | [At2g25300](http://atted.jp/cgi-bin/coex_Ath.cgi?gene=At2g25300&sort=all) | | transferase | | | **145.8** | 740.9 | 1634.1 | 2221.5 | 70.7 | 5537.3 |
| 77 | [At1g24120](http://atted.jp/cgi-bin/coex_Ath.cgi?gene=At1g24120&sort=all) | | ARL1 | | | **146.5** | 1370.0 | 5.0 | 670.7 | 4067.0 | 4204.5 |
| 78 | [At5g08590](http://atted.jp/cgi-bin/coex_Ath.cgi?gene=At5g08590&sort=all) | | SNRK2.1 | | | **150.6** | 441.4 | 805.7 | 1127.0 | 556.8 | 1102.5 |
| 79 | [At1g25375](http://atted.jp/cgi-bin/coex_Ath.cgi?gene=At1g25375&sort=all) | | lactamase | | | **150.6** | 707.3 | 618.5 | 1870.1 | 1026.1 | 4626.8 |
| 80 | [At2g48060](http://atted.jp/cgi-bin/coex_Ath.cgi?gene=At2g48060&sort=all) | |  | | | **152.0** | 1952.4 | 316.8 | 135.8 | 563.7 | 613.0 |
| 81 | [At5g49980](http://atted.jp/cgi-bin/coex_Ath.cgi?gene=At5g49980&sort=all) | | AFB5 | | | **152.9** | 77.8 | 520.8 | 2985.3 | 13249.9 | 2980.9 |
| 82 | [At4g32060](http://atted.jp/cgi-bin/coex_Ath.cgi?gene=At4g32060&sort=all) | | calcium-binding EF hand | | | **154.1** | 2206.2 | 540.5 | 10.0 | 314.3 | 6854.7 |
| 83 | [At5g24670](http://atted.jp/cgi-bin/coex_Ath.cgi?gene=At5g24670&sort=all) | | hydrolase | | | **154.1** | 28.2 | 5404.4 | 6386.0 | 48.0 | 688.9 |
| 84 | [At5g11580](http://atted.jp/cgi-bin/coex_Ath.cgi?gene=At5g11580&sort=all) | | RCC1 | | | **155.8** | 2629.3 | 74.8 | 48.1 | 5487.2 | 3172.7 |
| 85 | [At5g25560](http://atted.jp/cgi-bin/coex_Ath.cgi?gene=At5g25560&sort=all) | | zinc finger | | | **156.1** | 583.7 | 3650.9 | 1593.0 | 46.7 | 274.3 |
| 86 | [At1g05805](http://atted.jp/cgi-bin/coex_Ath.cgi?gene=At1g05805&sort=all) | | bHLH | | | **156.8** | 1269.5 | 143.3 | 4621.2 | 1803.5 | 5080.4 |
| 87 | [At5g67570](http://atted.jp/cgi-bin/coex_Ath.cgi?gene=At5g67570&sort=all) | | DG1 | | | **158.3** | 336.6 | 742.4 | 1947.3 | 479.7 | 1282.5 |
| 88 | [At1g12730](http://atted.jp/cgi-bin/coex_Ath.cgi?gene=At1g12730&sort=all) | | cell division cycle | | | **159.2** | 164.2 | 271.5 | 38.0 | 12857.7 | 5978.1 |
| 89 | [At2g16940](http://atted.jp/cgi-bin/coex_Ath.cgi?gene=At2g16940&sort=all) | | RRM | | | **160.0** | 333.3 | 1384.1 | 7942.6 | 378.3 | 1874.8 |
| 90 | [At1g26150](http://atted.jp/cgi-bin/coex_Ath.cgi?gene=At1g26150&sort=all) | | PERK10 | | | **161.9** | 404.5 | 529.9 | 1303.7 | 697.5 | 5184.1 |
| 91 | [At5g55230](http://atted.jp/cgi-bin/coex_Ath.cgi?gene=At5g55230&sort=all) | | MAP65-1 | | | **162.2** | 1022.1 | 55.9 | 781.5 | 6433.1 | 10670.1 |
| 92 | [256280_at](http://atted.jp/cgi-bin/coex_Ath.cgi?gene=256280_at&sort=all) | |  | | | **163.7** | 85.7 | 340.1 | 4275.8 | 12455.3 | 6934.1 |
| 93 | [At2g36720](http://atted.jp/cgi-bin/coex_Ath.cgi?gene=At2g36720&sort=all) | | transcription | | | **163.9** | 159.4 | 939.3 | 3149.3 | 1111.4 | 6009.2 |
| 94 | [At2g18750](http://atted.jp/cgi-bin/coex_Ath.cgi?gene=At2g18750&sort=all) | | calmodulin-binding | | | **169.0** | 2180.7 | 31.8 | 8838.7 | 223.9 | 361.9 |
| 95 | [At5g53480](http://atted.jp/cgi-bin/coex_Ath.cgi?gene=At5g53480&sort=all) | | importin beta-2 | | | **169.9** | 211.2 | 616.5 | 2457.1 | 1202.2 | 4970.2 |
| 96 | [At4g16180](http://atted.jp/cgi-bin/coex_Ath.cgi?gene=At4g16180&sort=all) | |  | | | **170.3** | 205.5 | 410.1 | 5617.4 | 766.5 | 731.3 |
| 97 | [At5g47500](http://atted.jp/cgi-bin/coex_Ath.cgi?gene=At5g47500&sort=all) | | pectinesterase | | | **172.0** | 1464.7 | 400.1 | 10908.6 | 5.3 | 5693.3 |
| 98 | [At2g26200](http://atted.jp/cgi-bin/coex_Ath.cgi?gene=At2g26200&sort=all) | |  | | | **172.8** | 405.7 | 265.5 | 182.1 | 6850.1 | 918.5 |
| 99 | [At4g03190](http://atted.jp/cgi-bin/coex_Ath.cgi?gene=At4g03190&sort=all) | | GRH1 | | | **174.9** | 371.5 | 1129.1 | 1741.3 | 3525.4 | 18788.8 |
| 100 | [At1g13280](http://atted.jp/cgi-bin/coex_Ath.cgi?gene=At1g13280&sort=all) | | AOC4 | | | **177.1** | 138.5 | 421.1 | 10944.7 | 7695.1 | 15031.7 |
| 101 | [At1g01220](http://atted.jp/cgi-bin/coex_Ath.cgi?gene=At1g01220&sort=all) | | kinase | | | **179.4** | 52.1 | 3134.6 | 758.2 | 9828.6 | 10075.0 |
| 102 | [At1g16710](http://atted.jp/cgi-bin/coex_Ath.cgi?gene=At1g16710&sort=all) | | HAC12 | | | **180.1** | 1059.4 | 52.3 | 4333.2 | 1530.5 | 2282.1 |
| 103 | [At4g35335](http://atted.jp/cgi-bin/coex_Ath.cgi?gene=At4g35335&sort=all) | | transporter | | | **182.0** | 28.4 | 3997.4 | 3306.0 | 459.1 | 623.5 |
| 104 | [At1g69830](http://atted.jp/cgi-bin/coex_Ath.cgi?gene=At1g69830&sort=all) | | AMY3 | | | **184.4** | 1642.6 | 190.6 | 41.0 | 2782.9 | 3299.5 |
| 105 | [At5g65310](http://atted.jp/cgi-bin/coex_Ath.cgi?gene=At5g65310&sort=all) | | ATHB5 | | | **185.5** | 126.4 | 300.7 | 646.8 | 9103.6 | 6533.8 |
| 106 | [At3g43610](http://atted.jp/cgi-bin/coex_Ath.cgi?gene=At3g43610&sort=all) | | tubulin binding | | | **187.9** | 390.0 | 585.1 | 1248.6 | 1285.7 | 8689.2 |
| 107 | [At5g26850](http://atted.jp/cgi-bin/coex_Ath.cgi?gene=At5g26850&sort=all) | |  | | | **188.3** | 937.6 | 104.3 | 1086.9 | 3060.5 | 16630.0 |
| 108 | [At1g53390](http://atted.jp/cgi-bin/coex_Ath.cgi?gene=At1g53390&sort=all) | | ATPase | | | **190.2** | 1685.5 | 88.3 | 4096.7 | 848.3 | 2039.7 |
| 109 | [At3g52640](http://atted.jp/cgi-bin/coex_Ath.cgi?gene=At3g52640&sort=all) | | nicastrin | | | **190.3** | 560.4 | 166.8 | 828.9 | 632.5 | 1122.0 |
| 110 | [At1g21730](http://atted.jp/cgi-bin/coex_Ath.cgi?gene=At1g21730&sort=all) | | MKRP1 | | | **193.9** | 3016.2 | 415.9 | 151.7 | 189.7 | 2734.5 |
| 111 | [At2g27900](http://atted.jp/cgi-bin/coex_Ath.cgi?gene=At2g27900&sort=all) | |  | | | **194.6** | 712.4 | 776.5 | 1057.2 | 25.2 | 3270.0 |
| 112 | [At4g14570](http://atted.jp/cgi-bin/coex_Ath.cgi?gene=At4g14570&sort=all) | | peptidase | | | **194.7** | 1492.4 | 250.4 | 2006.0 | 1002.1 | 5552.3 |
| 113 | [At5g43830](http://atted.jp/cgi-bin/coex_Ath.cgi?gene=At5g43830&sort=all) | |  | | | **197.6** | 989.5 | 6.9 | 515.0 | 5999.0 | 3405.6 |
| 114 | [At2g28070](http://atted.jp/cgi-bin/coex_Ath.cgi?gene=At2g28070&sort=all) | | transporter | | | **198.5** | 113.0 | 2346.5 | 2848.0 | 198.9 | 6363.1 |
| 115 | [At1g48330](http://atted.jp/cgi-bin/coex_Ath.cgi?gene=At1g48330&sort=all) | |  | | | **199.0** | 869.9 | 186.4 | 402.0 | 13688.8 | 5295.3 |
| 116 | [At5g24830](http://atted.jp/cgi-bin/coex_Ath.cgi?gene=At5g24830&sort=all) | | PPR | | | **201.9** | 153.0 | 977.6 | 1857.3 | 567.7 | 4913.5 |
| 117 | [At2g01170](http://atted.jp/cgi-bin/coex_Ath.cgi?gene=At2g01170&sort=all) | | BAT1 | | | **202.9** | 861.4 | 67.0 | 175.3 | 17118.9 | 11437.2 |
| 118 | [At1g67080](http://atted.jp/cgi-bin/coex_Ath.cgi?gene=At1g67080&sort=all) | | ABA4 | | | **204.2** | 65.8 | 1815.8 | 1834.6 | 3398.8 | 1981.3 |
| 119 | [At3g54790](http://atted.jp/cgi-bin/coex_Ath.cgi?gene=At3g54790&sort=all) | | U-box | | | **207.7** | 217.6 | 82.2 | 1124.9 | 14026.3 | 4962.9 |
| 120 | [At1g02300](http://atted.jp/cgi-bin/coex_Ath.cgi?gene=At1g02300&sort=all) | | protease | | | **208.0** | 90.1 | 497.1 | 514.9 | 2791.7 | 1675.8 |
| 121 | [At4g25880](http://atted.jp/cgi-bin/coex_Ath.cgi?gene=At4g25880&sort=all) | | APUM6 | | | **208.9** | 234.9 | 251.3 | 4150.7 | 3611.6 | 312.7 |
| 122 | [At1g54410](http://atted.jp/cgi-bin/coex_Ath.cgi?gene=At1g54410&sort=all) | | dehydrin | | | **210.6** | 669.8 | 1741.0 | 34.2 | 864.8 | 6202.4 |
| 123 | [At2g43550](http://atted.jp/cgi-bin/coex_Ath.cgi?gene=At2g43550&sort=all) | | inhibitor | | | **213.9** | 126.5 | 675.6 | 815.7 | 2542.7 | 3679.0 |
| 124 | 246175_s_at | |  | | | **214.0** | 568.0 | 1525.0 | 27.2 | 53.1 | 1135.0 |
| 125 | [At5g48960](http://atted.jp/cgi-bin/coex_Ath.cgi?gene=At5g48960&sort=all) | | nucleotidase | | | **215.4** | 166.4 | 397.5 | 3593.3 | 2706.3 | 6524.6 |
| 126 | [At3g13690](http://atted.jp/cgi-bin/coex_Ath.cgi?gene=At3g13690&sort=all) | | kinase | | | **217.1** | 497.2 | 645.3 | 880.9 | 3573.3 | 8475.7 |
| 127 | [At2g46560](http://atted.jp/cgi-bin/coex_Ath.cgi?gene=At2g46560&sort=all) | | transducin | | | **217.2** | 448.1 | 2109.8 | 3368.7 | 114.2 | 9522.3 |
| 128 | 245928_s_at | |  | | | **220.6** | 238.2 | 1340.3 | 4014.7 | 1499.2 | 1141.1 |
| 129 | [At5g27380](http://atted.jp/cgi-bin/coex_Ath.cgi?gene=At5g27380&sort=all) | | GSH2 | | | **220.8** | 714.2 | 363.9 | 11057.3 | 743.3 | 1915.5 |
| 130 | [245432_at](http://atted.jp/cgi-bin/coex_Ath.cgi?gene=245432_at&sort=all) | |  | | | **222.3** | 195.5 | 953.1 | 85.1 | 10226.1 | 2244.6 |
| 131 | [At1g60070](http://atted.jp/cgi-bin/coex_Ath.cgi?gene=At1g60070&sort=all) | | transporter | | | **223.1** | 190.4 | 3200.8 | 1105.5 | 1090.6 | 7706.0 |
| 132 | [At3g59110](http://atted.jp/cgi-bin/coex_Ath.cgi?gene=At3g59110&sort=all) | | kinase | | | **223.3** | 699.3 | 242.8 | 595.7 | 5836.6 | 4395.9 |
| 133 | 255292_s_at | |  | | | **223.4** | 885.9 | 1245.8 | 1040.3 | 266.4 | 2398.0 |
| 134 | [At3g20630](http://atted.jp/cgi-bin/coex_Ath.cgi?gene=At3g20630&sort=all) | | UBP14 | | | **225.0** | 256.1 | 567.6 | 5657.6 | 266.6 | 484.9 |
| 135 | [At5g02770](http://atted.jp/cgi-bin/coex_Ath.cgi?gene=At5g02770&sort=all) | |  | | | **226.0** | 192.6 | 899.4 | 1876.9 | 606.3 | 919.3 |
| 136 | [At2g39900](http://atted.jp/cgi-bin/coex_Ath.cgi?gene=At2g39900&sort=all) | | LIM | | | **229.6** | 1635.8 | 400.2 | 55.1 | 11065.2 | 7388.6 |
| 137 | [At3g55920](http://atted.jp/cgi-bin/coex_Ath.cgi?gene=At3g55920&sort=all) | | rotamase | | | **231.9** | 515.3 | 1619.4 | 2195.0 | 635.5 | 4483.1 |
| 138 | [At5g49470](http://atted.jp/cgi-bin/coex_Ath.cgi?gene=At5g49470&sort=all) | | kinase | | | **234.0** | 1165.6 | 2751.1 | 5254.0 | 43.8 | 1119.7 |
| 139 | [At1g76630](http://atted.jp/cgi-bin/coex_Ath.cgi?gene=At1g76630&sort=all) | | TPR | | | **235.2** | 1401.8 | 146.6 | 2452.6 | 151.0 | 610.5 |
| 140 | [At4g24290](http://atted.jp/cgi-bin/coex_Ath.cgi?gene=At4g24290&sort=all) | |  | | | **235.4** | 859.6 | 158.4 | 9197.0 | 137.6 | 887.4 |
| 141 | [At5g65760](http://atted.jp/cgi-bin/coex_Ath.cgi?gene=At5g65760&sort=all) | | S28 | | | **235.4** | 108.0 | 371.0 | 5920.9 | 11531.3 | 11672.9 |
| 142 | [At5g27970](http://atted.jp/cgi-bin/coex_Ath.cgi?gene=At5g27970&sort=all) | | binding | | | **235.6** | 84.6 | 2230.8 | 1736.6 | 2057.7 | 2386.8 |
| 143 | [At2g40840](http://atted.jp/cgi-bin/coex_Ath.cgi?gene=At2g40840&sort=all) | | DPE2 | | | **236.1** | 1261.8 | 83.0 | 255.5 | 16011.2 | 9967.0 |
| 144 | [At1g09980](http://atted.jp/cgi-bin/coex_Ath.cgi?gene=At1g09980&sort=all) | |  | | | **239.0** | 1100.8 | 517.7 | 3659.8 | 99.8 | 907.6 |
| 145 | [At3g09410](http://atted.jp/cgi-bin/coex_Ath.cgi?gene=At3g09410&sort=all) | | pectinacetylesterase | | | **239.7** | 1279.6 | 333.5 | 1924.6 | 1754.0 | 1222.7 |
| 146 | [At1g62430](http://atted.jp/cgi-bin/coex_Ath.cgi?gene=At1g62430&sort=all) | | CDS1 | | | **240.5** | 1565.0 | 138.8 | 171.0 | 2392.8 | 1454.6 |
| 147 | [At3g46970](http://atted.jp/cgi-bin/coex_Ath.cgi?gene=At3g46970&sort=all) | | PHS2 | | | **241.1** | 1518.2 | 193.4 | 55.2 | 17179.7 | 10011.7 |
| 148 | [At1g12930](http://atted.jp/cgi-bin/coex_Ath.cgi?gene=At1g12930&sort=all) | | importin | | | **244.1** | 69.3 | 1848.2 | 4257.8 | 449.1 | 388.5 |
| 149 | 252576_s_at | |  | | | **250.4** | 203.2 | 1031.8 | 3352.9 | 776.8 | 660.3 |
| 150 | [At5g41260](http://atted.jp/cgi-bin/coex_Ath.cgi?gene=At5g41260&sort=all) | | kinase | | | **250.5** | 2418.9 | 284.0 | 222.8 | 467.6 | 12319.7 |
| 151 | [At4g31050](http://atted.jp/cgi-bin/coex_Ath.cgi?gene=At4g31050&sort=all) | | LIP2p | | | **250.7** | 1085.5 | 92.9 | 120.0 | 11744.1 | 1051.2 |
| 152 | [At5g08120](http://atted.jp/cgi-bin/coex_Ath.cgi?gene=At5g08120&sort=all) | | MBP2C | | | **251.1** | 327.8 | 1021.7 | 453.7 | 6257.1 | 7366.7 |
| 153 | [At2g39190](http://atted.jp/cgi-bin/coex_Ath.cgi?gene=At2g39190&sort=all) | | ATH8 | | | **251.4** | 966.9 | 527.5 | 2428.3 | 876.7 | 4338.2 |
| 154 | [At1g13930](http://atted.jp/cgi-bin/coex_Ath.cgi?gene=At1g13930&sort=all) | |  | | | **251.7** | 270.1 | 849.5 | 159.3 | 20281.5 | 11892.0 |
| 155 | [At2g02400](http://atted.jp/cgi-bin/coex_Ath.cgi?gene=At2g02400&sort=all) | | reductase | | | **252.6** | 1093.0 | 465.8 | 743.0 | 14377.3 | 11031.7 |
| 156 | [At5g42420](http://atted.jp/cgi-bin/coex_Ath.cgi?gene=At5g42420&sort=all) | | transporter | | | **252.9** | 1338.5 | 307.6 | 235.8 | 4204.8 | 10902.6 |
| 157 | [At3g27325](http://atted.jp/cgi-bin/coex_Ath.cgi?gene=At3g27325&sort=all) | | hydrolase | | | **258.0** | 782.0 | 362.7 | 3161.0 | 160.2 | 2523.0 |
| 158 | [At2g40410](http://atted.jp/cgi-bin/coex_Ath.cgi?gene=At2g40410&sort=all) | | nuclease | | | **258.2** | 1820.4 | 586.9 | 385.2 | 1380.2 | 865.8 |
| 159 | [At1g33680](http://atted.jp/cgi-bin/coex_Ath.cgi?gene=At1g33680&sort=all) | | RNA binding | | | **258.6** | 737.5 | 646.0 | 5317.0 | 363.8 | 5532.4 |
| 160 | [At2g22650](http://atted.jp/cgi-bin/coex_Ath.cgi?gene=At2g22650&sort=all) | | oxidoreductase | | | **262.7** | 42.4 | 4193.9 | 2483.4 | 5017.4 | 5060.7 |
| 161 | [At2g20850](http://atted.jp/cgi-bin/coex_Ath.cgi?gene=At2g20850&sort=all) | | SRF1 | | | **263.7** | 492.0 | 686.6 | 696.5 | 1109.1 | 5834.2 |
| 162 | 253528_s_at | |  | | | **263.9** | 308.9 | 940.9 | 7144.1 | 1182.7 | 9265.5 |
| 163 | [At5g35980](http://atted.jp/cgi-bin/coex_Ath.cgi?gene=At5g35980&sort=all) | | YAK1 | | | **269.3** | 3882.5 | 41.0 | 6802.4 | 135.7 | 955.9 |
| 164 | [At5g47770](http://atted.jp/cgi-bin/coex_Ath.cgi?gene=At5g47770&sort=all) | | FPS1 | | | **270.5** | 204.1 | 3834.0 | 3171.1 | 10390.7 | 7871.2 |
| 165 | [At1g75190](http://atted.jp/cgi-bin/coex_Ath.cgi?gene=At1g75190&sort=all) | |  | | | **272.1** | 1217.4 | 263.2 | 2777.5 | 3488.2 | 956.1 |
| 166 | [At5g37830](http://atted.jp/cgi-bin/coex_Ath.cgi?gene=At5g37830&sort=all) | | OXP1 | | | **272.1** | 263.3 | 257.2 | 5991.4 | 4224.8 | 3926.3 |
| 167 | [At3g62980](http://atted.jp/cgi-bin/coex_Ath.cgi?gene=At3g62980&sort=all) | | TIR1 | | | **272.5** | 661.3 | 151.0 | 13989.0 | 3148.7 | 4598.4 |
| 168 | [At2g27170](http://atted.jp/cgi-bin/coex_Ath.cgi?gene=At2g27170&sort=all) | | TTN7 | | | **275.1** | 760.2 | 416.6 | 5518.9 | 149.0 | 138.9 |
| 169 | [At1g06470](http://atted.jp/cgi-bin/coex_Ath.cgi?gene=At1g06470&sort=all) | | translocator | | | **276.4** | 647.0 | 2651.3 | 124.4 | 798.1 | 2553.5 |
| 170 | [At3g28860](http://atted.jp/cgi-bin/coex_Ath.cgi?gene=At3g28860&sort=all) | | ABCB19 | | | **276.5** | 602.7 | 1619.4 | 827.5 | 5730.1 | 16524.2 |
| 171 | [At4g00630](http://atted.jp/cgi-bin/coex_Ath.cgi?gene=At4g00630&sort=all) | | KEA2 | | | **278.6** | 664.1 | 737.4 | 2700.9 | 893.1 | 159.9 |
| 172 | [At1g79500](http://atted.jp/cgi-bin/coex_Ath.cgi?gene=At1g79500&sort=all) | | kdsA1 | | | **280.8** | 1931.0 | 89.5 | 1017.7 | 3455.4 | 10164.6 |
| 173 | [At3g14310](http://atted.jp/cgi-bin/coex_Ath.cgi?gene=At3g14310&sort=all) | | PME3 | | | **281.2** | 994.3 | 486.0 | 3218.9 | 1465.8 | 6483.5 |
| 174 | [At1g79990](http://atted.jp/cgi-bin/coex_Ath.cgi?gene=At1g79990&sort=all) | | protein binding | | | **283.6** | 687.7 | 2012.9 | 3981.7 | 68.2 | 6489.3 |
| 175 | [At4g24900](http://atted.jp/cgi-bin/coex_Ath.cgi?gene=At4g24900&sort=all) | |  | | | **283.9** | 335.8 | 11690.2 | 131.4 | 75.1 | 10705.1 |
| 176 | [At1g68560](http://atted.jp/cgi-bin/coex_Ath.cgi?gene=At1g68560&sort=all) | | XYL1 | | | **285.9** | 135.1 | 998.1 | 503.0 | 2242.2 | 15791.2 |
| 177 | [At3g20780](http://atted.jp/cgi-bin/coex_Ath.cgi?gene=At3g20780&sort=all) | | TOP6B | | | **286.3** | 1171.2 | 444.4 | 5003.8 | 96.3 | 11374.0 |
| 178 | [At5g61990](http://atted.jp/cgi-bin/coex_Ath.cgi?gene=At5g61990&sort=all) | | PPR | | | **288.4** | 418.2 | 1467.4 | 9586.5 | 2658.4 | 7258.5 |
| 179 | [At1g31650](http://atted.jp/cgi-bin/coex_Ath.cgi?gene=At1g31650&sort=all) | | ROPGEF14 | | | **290.7** | 112.0 | 2031.2 | 1450.3 | 12634.3 | 2823.4 |
| 180 | [At5g46460](http://atted.jp/cgi-bin/coex_Ath.cgi?gene=At5g46460&sort=all) | | PPR | | | **292.7** | 1051.2 | 123.6 | 4256.4 | 17724.5 | 3275.5 |
| 181 | [At1g08680](http://atted.jp/cgi-bin/coex_Ath.cgi?gene=At1g08680&sort=all) | | ZIGA4 | | | **294.8** | 449.7 | 791.2 | 4566.1 | 874.8 | 220.0 |
| 182 | [At3g02710](http://atted.jp/cgi-bin/coex_Ath.cgi?gene=At3g02710&sort=all) | | NAP | | | **302.5** | 128.1 | 1384.6 | 4198.9 | 1190.4 | 6090.5 |
| 183 | [At4g22540](http://atted.jp/cgi-bin/coex_Ath.cgi?gene=At4g22540&sort=all) | | ORP2A | | | **305.2** | 4634.2 | 20.1 | 289.0 | 1809.5 | 11087.8 |
| 184 | 256770_at | |  | | | **314.0** | 1267.2 | 340.4 | 3292.8 | 589.6 | 16326.5 |
| 185 | [At3g06130](http://atted.jp/cgi-bin/coex_Ath.cgi?gene=At3g06130&sort=all) | | heavy-metal-associated | | | **314.9** | 671.9 | 2499.5 | 4488.8 | 659.4 | 10616.3 |
| 186 | [At1g10760](http://atted.jp/cgi-bin/coex_Ath.cgi?gene=At1g10760&sort=all) | | SEX1 | | | **315.4** | 2565.2 | 320.6 | 20.1 | 14669.6 | 10269.8 |
| 187 | [At3g53800](http://atted.jp/cgi-bin/coex_Ath.cgi?gene=At3g53800&sort=all) | | armadillo/beta-catenin repeat | | | **317.4** | 3231.9 | 63.1 | 42.9 | 7941.8 | 20016.8 |
| 188 | [At1g02205](http://atted.jp/cgi-bin/coex_Ath.cgi?gene=At1g02205&sort=all) | | CER1 | | | **320.4** | 453.5 | 3460.9 | 2488.3 | 2881.2 | 2279.7 |
| 189 | [At4g17150](http://atted.jp/cgi-bin/coex_Ath.cgi?gene=At4g17150&sort=all) | |  | | | **320.5** | 1131.0 | 559.4 | 65.5 | 10140.9 | 10820.7 |
| 190 | [At5g57700](http://atted.jp/cgi-bin/coex_Ath.cgi?gene=At5g57700&sort=all) | | BNR | | | **322.4** | 911.1 | 769.8 | 581.5 | 4897.9 | 9881.3 |
| 191 | [At1g53710](http://atted.jp/cgi-bin/coex_Ath.cgi?gene=At1g53710&sort=all) | | phosphatase | | | **324.1** | 1299.3 | 517.5 | 4113.2 | 1666.2 | 1136.2 |
| 192 | 246654_s_at | |  | | | **324.1** | 297.1 | 2722.1 | 2796.5 | 3309.5 | 4743.1 |
| 193 | [At3g06580](http://atted.jp/cgi-bin/coex_Ath.cgi?gene=At3g06580&sort=all) | | GALK | | | **324.9** | 601.5 | 234.2 | 7564.2 | 528.7 | 1956.9 |
| 194 | [At5g02860](http://atted.jp/cgi-bin/coex_Ath.cgi?gene=At5g02860&sort=all) | | PPR | | | **327.6** | 599.6 | 350.6 | 335.7 | 11019.4 | 19210.4 |
| 195 | 258148_s_at | |  | | | **328.2** | 1086.0 | 26.1 | 3839.9 | 6509.2 | 7687.6 |
| 196 | [At1g54370](http://atted.jp/cgi-bin/coex_Ath.cgi?gene=At1g54370&sort=all) | | NHX5 | | | **328.5** | 934.0 | 2789.3 | 1678.3 | 266.9 | 360.1 |
| 197 | [At4g33490](http://atted.jp/cgi-bin/coex_Ath.cgi?gene=At4g33490&sort=all) | | endopeptidase | | | **328.9** | 897.5 | 477.1 | 329.5 | 2094.8 | 19454.7 |
| 198 | [At2g13560](http://atted.jp/cgi-bin/coex_Ath.cgi?gene=At2g13560&sort=all) | | oxidoreductase | | | **331.4** | 650.4 | 2411.8 | 2227.0 | 1700.2 | 15082.3 |
| 199 | [At1g52150](http://atted.jp/cgi-bin/coex_Ath.cgi?gene=At1g52150&sort=all) | | HB-15 | | | **335.3** | 523.6 | 3881.8 | 5215.0 | 334.9 | 1299.2 |
| 200 | 245881_at | |  | | | **335.7** | 257.5 | 890.1 | 1861.3 | 7667.9 | 5088.4 |
| 201 | [At3g04910](http://atted.jp/cgi-bin/coex_Ath.cgi?gene=At3g04910&sort=all) | | WNK1 | | | **336.8** | 3546.6 | 289.8 | 122.7 | 860.5 | 4732.4 |
| 202 | [At5g13740](http://atted.jp/cgi-bin/coex_Ath.cgi?gene=At5g13740&sort=all) | | ZIF1 | | | **336.9** | 699.3 | 1263.4 | 3355.2 | 5771.7 | 2763.3 |
| 203 | [At5g36890](http://atted.jp/cgi-bin/coex_Ath.cgi?gene=At5g36890&sort=all) | | BGLU42 | | | **339.0** | 22.7 | 1230.4 | 791.5 | 8501.1 | 7790.4 |
| 204 | [At3g55850](http://atted.jp/cgi-bin/coex_Ath.cgi?gene=At3g55850&sort=all) | | LAF3 | | | **339.1** | 1382.2 | 3135.3 | 4231.0 | 112.7 | 102.0 |
| 205 | [At1g71696](http://atted.jp/cgi-bin/coex_Ath.cgi?gene=At1g71696&sort=all) | | SOL1 | | | **339.5** | 932.3 | 6521.6 | 831.1 | 33.8 | 1260.2 |
| 206 | [At3g51310](http://atted.jp/cgi-bin/coex_Ath.cgi?gene=At3g51310&sort=all) | | VPS35C | | | **340.9** | 1390.7 | 852.7 | 5438.9 | 1100.3 | 3724.0 |
| 207 | [At5g49945](http://atted.jp/cgi-bin/coex_Ath.cgi?gene=At5g49945&sort=all) | |  | | | **341.2** | 904.4 | 277.8 | 1156.0 | 13330.5 | 4430.9 |
| 208 | [At4g14270](http://atted.jp/cgi-bin/coex_Ath.cgi?gene=At4g14270&sort=all) | |  | | | **341.6** | 190.4 | 418.4 | 258.5 | 9121.3 | 17344.5 |
| 209 | [At1g70210](http://atted.jp/cgi-bin/coex_Ath.cgi?gene=At1g70210&sort=all) | | CYCD1;1 | | | **344.6** | 867.2 | 1073.2 | 3777.7 | 3495.2 | 190.7 |
| 210 | [At5g40870](http://atted.jp/cgi-bin/coex_Ath.cgi?gene=At5g40870&sort=all) | | UK | | | **345.2** | 1401.4 | 638.8 | 836.4 | 4388.5 | 9322.2 |
| 211 | [At1g22060](http://atted.jp/cgi-bin/coex_Ath.cgi?gene=At1g22060&sort=all) | |  | | | **345.7** | 990.9 | 1121.0 | 1870.2 | 986.7 | 5915.0 |
| 212 | [At1g67140](http://atted.jp/cgi-bin/coex_Ath.cgi?gene=At1g67140&sort=all) | | SWEETIE | | | **348.2** | 77.4 | 8254.6 | 5259.4 | 1113.6 | 3510.2 |
| 213 | [At3g14840](http://atted.jp/cgi-bin/coex_Ath.cgi?gene=At3g14840&sort=all) | | kinase | | | **348.9** | 545.0 | 1159.0 | 4754.0 | 3153.6 | 17477.2 |
| 214 | [At3g28130](http://atted.jp/cgi-bin/coex_Ath.cgi?gene=At3g28130&sort=all) | | MtN21 | | | **349.4** | 150.0 | 1520.0 | 1453.8 | 7016.1 | 2137.6 |
| 215 | [At4g02120](http://atted.jp/cgi-bin/coex_Ath.cgi?gene=At4g02120&sort=all) | | ligase | | | **350.7** | 513.9 | 3577.5 | 7369.4 | 540.9 | 154.7 |
| 216 | [254679_at](http://atted.jp/cgi-bin/coex_Ath.cgi?gene=254679_at&sort=all) | |  | | | **351.4** | 1310.3 | 2208.1 | 1934.1 | 235.7 | 115.5 |
| 217 | [At1g14460](http://atted.jp/cgi-bin/coex_Ath.cgi?gene=At1g14460&sort=all) | | polymerase | | | **353.6** | 139.9 | 3565.3 | 2794.3 | 2322.7 | 5931.5 |
| 218 | [At2g26730](http://atted.jp/cgi-bin/coex_Ath.cgi?gene=At2g26730&sort=all) | | kinase | | | **354.1** | 121.0 | 2649.4 | 850.7 | 9670.5 | 1868.8 |
| 219 | [At2g22830](http://atted.jp/cgi-bin/coex_Ath.cgi?gene=At2g22830&sort=all) | | SQE2 | | | **354.4** | 656.6 | 1010.3 | 549.5 | 5598.1 | 16196.8 |
| 220 | [At1g51940](http://atted.jp/cgi-bin/coex_Ath.cgi?gene=At1g51940&sort=all) | | LysM | | | **355.0** | 1154.7 | 603.5 | 194.4 | 5816.4 | 12796.5 |
| 221 | [At1g20540](http://atted.jp/cgi-bin/coex_Ath.cgi?gene=At1g20540&sort=all) | | transducin | | | **355.1** | 614.9 | 3016.2 | 3133.5 | 2484.2 | 5975.0 |
| 222 | [At5g58600](http://atted.jp/cgi-bin/coex_Ath.cgi?gene=At5g58600&sort=all) | | PMR5 | | | **358.8** | 1164.9 | 498.1 | 106.9 | 7383.5 | 16287.4 |
| 223 | [At2g39080](http://atted.jp/cgi-bin/coex_Ath.cgi?gene=At2g39080&sort=all) | | binding | | | **359.2** | 465.4 | 119.0 | 2166.0 | 2040.1 | 5318.8 |
| 224 | [At4g34980](http://atted.jp/cgi-bin/coex_Ath.cgi?gene=At4g34980&sort=all) | | SLP2 | | | **363.5** | 175.0 | 491.1 | 3762.8 | 7910.1 | 5174.7 |
| 225 | [At2g45540](http://atted.jp/cgi-bin/coex_Ath.cgi?gene=At2g45540&sort=all) | | beige | | | **363.9** | 643.0 | 2034.0 | 2135.0 | 927.8 | 8419.6 |
| 226 | [At4g38520](http://atted.jp/cgi-bin/coex_Ath.cgi?gene=At4g38520&sort=all) | | PP2C | | | **364.0** | 157.6 | 2037.5 | 3065.4 | 7465.8 | 5739.6 |
| 227 | [At4g17140](http://atted.jp/cgi-bin/coex_Ath.cgi?gene=At4g17140&sort=all) | |  | | | **364.4** | 3381.5 | 308.0 | 391.5 | 392.7 | 2297.9 |
| 228 | [266748_at](http://atted.jp/cgi-bin/coex_Ath.cgi?gene=266748_at&sort=all) | |  | | | **366.3** | 74.7 | 3717.3 | 12807.1 | 590.8 | 272.9 |
| 229 | [At2g02570](http://atted.jp/cgi-bin/coex_Ath.cgi?gene=At2g02570&sort=all) | | nucleic acid binding | | | **366.4** | 833.7 | 491.9 | 633.0 | 945.0 | 811.8 |
| 230 | [265599_at](http://atted.jp/cgi-bin/coex_Ath.cgi?gene=265599_at&sort=all) | |  | | | **367.7** | 4008.2 | 304.3 | 667.1 | 822.5 | 140.2 |
| 231 | [At3g06450](http://atted.jp/cgi-bin/coex_Ath.cgi?gene=At3g06450&sort=all) | | anion exchange | | | **370.5** | 811.7 | 64.9 | 4015.3 | 2915.6 | 12398.5 |
| 232 | [At2g39580](http://atted.jp/cgi-bin/coex_Ath.cgi?gene=At2g39580&sort=all) | |  | | | **372.5** | 2566.2 | 51.6 | 6070.3 | 418.3 | 7164.7 |
| 233 | [At4g17020](http://atted.jp/cgi-bin/coex_Ath.cgi?gene=At4g17020&sort=all) | | transcription | | | **373.3** | 631.6 | 338.1 | 1035.2 | 2630.3 | 632.5 |
| 234 | [At3g19490](http://atted.jp/cgi-bin/coex_Ath.cgi?gene=At3g19490&sort=all) | | NHD1 | | | **373.5** | 4276.0 | 19.9 | 1217.3 | 7956.1 | 8665.0 |
| 235 | [At3g01310](http://atted.jp/cgi-bin/coex_Ath.cgi?gene=At3g01310&sort=all) | | oxidoreductase | | | **377.4** | 1547.4 | 270.3 | 57.8 | 7930.7 | 12516.0 |
| 236 | [At5g08530](http://atted.jp/cgi-bin/coex_Ath.cgi?gene=At5g08530&sort=all) | | CI51 | | | **379.0** | 108.0 | 280.7 | 16037.4 | 14201.8 | 6512.0 |
| 237 | 260118_s_at | |  | | | **384.1** | 447.9 | 3057.5 | 3355.8 | 2111.9 | 6192.1 |
| 238 | [At2g35450](http://atted.jp/cgi-bin/coex_Ath.cgi?gene=At2g35450&sort=all) | | hydrolase | | | **388.5** | 1121.8 | 352.3 | 1271.3 | 1069.6 | 12381.4 |
| 239 | [At5g13980](http://atted.jp/cgi-bin/coex_Ath.cgi?gene=At5g13980&sort=all) | | hydrolase | | | **389.2** | 1133.6 | 1013.2 | 2082.0 | 12249.2 | 5623.6 |
| 240 | [At5g18230](http://atted.jp/cgi-bin/coex_Ath.cgi?gene=At5g18230&sort=all) | | NOT2/NOT3/NOT5 | | | **395.9** | 1906.4 | 1597.0 | 4888.7 | 30.2 | 1382.5 |
| 241 | [At4g27440](http://atted.jp/cgi-bin/coex_Ath.cgi?gene=At4g27440&sort=all) | | PORB | | | **397.8** | 2955.3 | 74.0 | 393.7 | 12417.1 | 18911.0 |
| 242 | [At3g19400](http://atted.jp/cgi-bin/coex_Ath.cgi?gene=At3g19400&sort=all) | | cysteinease | | | **399.4** | 1318.5 | 880.0 | 1190.8 | 963.8 | 1728.7 |
| 243 | [At4g33080](http://atted.jp/cgi-bin/coex_Ath.cgi?gene=At4g33080&sort=all) | | kinase | | | **402.3** | 180.6 | 8125.4 | 10660.1 | 84.2 | 173.1 |
| 244 | [At1g70620](http://atted.jp/cgi-bin/coex_Ath.cgi?gene=At1g70620&sort=all) | | cyclin | | | **402.4** | 2507.6 | 1579.4 | 656.0 | 15.6 | 8056.2 |
| 245 | 262062_s_at | |  | | | **402.6** | 1294.7 | 1292.5 | 3994.5 | 73.0 | 5533.9 |
| 246 | [At3g16470](http://atted.jp/cgi-bin/coex_Ath.cgi?gene=At3g16470&sort=all) | | JR1 | | | **403.9** | 51.5 | 10014.0 | 14466.5 | 2308.3 | 579.5 |
| 247 | [At4g38600](http://atted.jp/cgi-bin/coex_Ath.cgi?gene=At4g38600&sort=all) | | KAK | | | **404.1** | 581.6 | 549.8 | 4737.6 | 339.4 | 2636.8 |
| 248 | [At5g47240](http://atted.jp/cgi-bin/coex_Ath.cgi?gene=At5g47240&sort=all) | | atnudt8 | | | **408.4** | 1623.7 | 386.0 | 6568.1 | 1133.0 | 1832.2 |
| 249 | [At1g56230](http://atted.jp/cgi-bin/coex_Ath.cgi?gene=At1g56230&sort=all) | |  | | | **408.9** | 1287.3 | 681.9 | 2617.8 | 7772.7 | 167.4 |
| 250 | [At5g16290](http://atted.jp/cgi-bin/coex_Ath.cgi?gene=At5g16290&sort=all) | | synthase | | | **412.2** | 173.2 | 2593.5 | 9399.2 | 833.4 | 7337.6 |
| 251 | [At5g62350](http://atted.jp/cgi-bin/coex_Ath.cgi?gene=At5g62350&sort=all) | | FL5-2I22 | | | **412.8** | 324.0 | 650.9 | 2543.1 | 3084.4 | 16625.6 |
| 252 | [At3g60510](http://atted.jp/cgi-bin/coex_Ath.cgi?gene=At3g60510&sort=all) | | isomerase | | | **415.1** | 240.9 | 1253.0 | 2385.0 | 11943.6 | 3384.0 |
| 253 | [At3g30300](http://atted.jp/cgi-bin/coex_Ath.cgi?gene=At3g30300&sort=all) | |  | | | **418.7** | 442.5 | 2490.8 | 2370.2 | 2869.7 | 5207.2 |
| 254 | [At1g69690](http://atted.jp/cgi-bin/coex_Ath.cgi?gene=At1g69690&sort=all) | | transcription | | | **420.6** | 88.6 | 3580.3 | 2157.6 | 5643.3 | 11390.6 |
| 255 | [At2g39930](http://atted.jp/cgi-bin/coex_Ath.cgi?gene=At2g39930&sort=all) | | ISA1 | | | **420.6** | 643.2 | 760.0 | 1975.0 | 3946.6 | 10591.6 |
| 256 | [At3g50590](http://atted.jp/cgi-bin/coex_Ath.cgi?gene=At3g50590&sort=all) | | nucleotide binding | | | **422.5** | 706.0 | 673.6 | 2876.9 | 1686.8 | 919.8 |
| 257 | [At5g44750](http://atted.jp/cgi-bin/coex_Ath.cgi?gene=At5g44750&sort=all) | | REV1 | | | **423.5** | 2957.5 | 2086.0 | 1595.6 | 67.8 | 1119.5 |
| 258 | [At1g71860](http://atted.jp/cgi-bin/coex_Ath.cgi?gene=At1g71860&sort=all) | | PTP1 | | | **425.6** | 164.2 | 6634.7 | 16660.9 | 287.5 | 10334.5 |
| 259 | [At3g16180](http://atted.jp/cgi-bin/coex_Ath.cgi?gene=At3g16180&sort=all) | | POT | | | **427.1** | 1907.8 | 700.6 | 86.3 | 8391.2 | 15897.9 |
| 260 | [At5g24490](http://atted.jp/cgi-bin/coex_Ath.cgi?gene=At5g24490&sort=all) | | ribosome | | | **432.1** | 2106.1 | 223.1 | 390.3 | 3542.3 | 18851.3 |
| 261 | [At1g34340](http://atted.jp/cgi-bin/coex_Ath.cgi?gene=At1g34340&sort=all) | | thioesterase | | | **432.6** | 6365.9 | 1317.6 | 550.4 | 46.9 | 1201.3 |
| 262 | [At4g34960](http://atted.jp/cgi-bin/coex_Ath.cgi?gene=At4g34960&sort=all) | | rotamase | | | **434.4** | 335.8 | 1945.3 | 3681.2 | 2118.5 | 6011.2 |
| 263 | [At3g62000](http://atted.jp/cgi-bin/coex_Ath.cgi?gene=At3g62000&sort=all) | | transferase | | | **435.2** | 361.3 | 4503.2 | 4849.4 | 1452.7 | 1910.2 |
| 264 | [At4g09020](http://atted.jp/cgi-bin/coex_Ath.cgi?gene=At4g09020&sort=all) | | ISA3 | | | **435.3** | 441.2 | 261.2 | 269.0 | 19135.4 | 12717.9 |
| 265 | [At1g32810](http://atted.jp/cgi-bin/coex_Ath.cgi?gene=At1g32810&sort=all) | | protein binding | | | **435.3** | 912.8 | 471.5 | 2094.5 | 3677.2 | 212.4 |
| 266 | [At2g19170](http://atted.jp/cgi-bin/coex_Ath.cgi?gene=At2g19170&sort=all) | | SLP3 | | | **437.4** | 1024.5 | 1195.8 | 1673.9 | 5192.3 | 10998.5 |
| 267 | [At5g38150](http://atted.jp/cgi-bin/coex_Ath.cgi?gene=At5g38150&sort=all) | | PMI15 | | | **438.0** | 454.0 | 2939.6 | 2184.5 | 349.2 | 7465.8 |
| 268 | [At5g35170](http://atted.jp/cgi-bin/coex_Ath.cgi?gene=At5g35170&sort=all) | | kinase | | | **438.5** | 2324.3 | 546.1 | 867.2 | 130.9 | 7144.9 |
| 269 | [At2g23740](http://atted.jp/cgi-bin/coex_Ath.cgi?gene=At2g23740&sort=all) | | transcription | | | **439.2** | 1318.8 | 511.5 | 1719.2 | 1086.8 | 2352.7 |
| 270 | [At4g24390](http://atted.jp/cgi-bin/coex_Ath.cgi?gene=At4g24390&sort=all) | | FBX14 | | | **439.4** | 56.0 | 1281.3 | 1058.5 | 10077.1 | 15253.8 |
| 271 | [At2g27980](http://atted.jp/cgi-bin/coex_Ath.cgi?gene=At2g27980&sort=all) | | protein binding | | | **440.4** | 301.9 | 4061.7 | 4350.7 | 2472.2 | 2022.0 |
| 272 | [At1g80800](http://atted.jp/cgi-bin/coex_Ath.cgi?gene=At1g80800&sort=all) | |  | | | **440.6** | 259.5 | 4130.0 | 1516.5 | 9587.9 | 6526.2 |
| 273 | [At5g63420](http://atted.jp/cgi-bin/coex_Ath.cgi?gene=At5g63420&sort=all) | | emb2746 | | | **442.4** | 2410.4 | 150.6 | 434.2 | 3403.9 | 5561.8 |
| 274 | [At1g52410](http://atted.jp/cgi-bin/coex_Ath.cgi?gene=At1g52410&sort=all) | | TSA1 | | | **442.8** | 56.9 | 3670.3 | 16266.5 | 2516.8 | 5438.3 |
| 275 | [At4g00400](http://atted.jp/cgi-bin/coex_Ath.cgi?gene=At4g00400&sort=all) | | GPAT8 | | | **443.9** | 674.0 | 820.0 | 2125.3 | 4259.2 | 1115.4 |
| 276 | [At1g80790](http://atted.jp/cgi-bin/coex_Ath.cgi?gene=At1g80790&sort=all) | | XH | | | **444.8** | 166.9 | 1749.2 | 6770.0 | 2852.8 | 944.4 |
| 277 | [At5g13840](http://atted.jp/cgi-bin/coex_Ath.cgi?gene=At5g13840&sort=all) | | FZR3 | | | **447.7** | 1923.0 | 653.0 | 14344.2 | 39.0 | 2081.6 |
| 278 | [At4g32730](http://atted.jp/cgi-bin/coex_Ath.cgi?gene=At4g32730&sort=all) | | PC-MYB1 | | | **449.0** | 843.0 | 1138.4 | 1106.1 | 1214.3 | 3301.4 |
| 279 | 252344_s_at | |  | | | **449.0** | 2936.1 | 80.0 | 214.6 | 4581.0 | 1754.3 |
| 280 | [At5g35750](http://atted.jp/cgi-bin/coex_Ath.cgi?gene=At5g35750&sort=all) | | AHK2 | | | **450.3** | 663.5 | 2007.3 | 2174.8 | 3531.7 | 591.1 |
| 281 | [At1g60860](http://atted.jp/cgi-bin/coex_Ath.cgi?gene=At1g60860&sort=all) | | AGD2 | | | **450.4** | 1511.6 | 538.3 | 1489.3 | 3174.9 | 3172.2 |
| 282 | [249135_at](http://atted.jp/cgi-bin/coex_Ath.cgi?gene=249135_at&sort=all) | |  | | | **451.8** | 2749.1 | 346.8 | 658.3 | 777.3 | 3739.0 |
| 283 | [At2g47390](http://atted.jp/cgi-bin/coex_Ath.cgi?gene=At2g47390&sort=all) | | peptidase | | | **455.8** | 1683.2 | 593.9 | 1072.2 | 1262.4 | 6222.4 |
| 284 | [At5g66880](http://atted.jp/cgi-bin/coex_Ath.cgi?gene=At5g66880&sort=all) | | SNRK2.3 | | | **455.8** | 612.2 | 322.7 | 166.5 | 15418.2 | 15086.1 |
| 285 | [At2g28310](http://atted.jp/cgi-bin/coex_Ath.cgi?gene=At2g28310&sort=all) | |  | | | **456.6** | 239.7 | 4106.2 | 2066.2 | 4391.8 | 1131.4 |
| 286 | [At1g65370](http://atted.jp/cgi-bin/coex_Ath.cgi?gene=At1g65370&sort=all) | | MATH | | | **457.5** | 192.1 | 7812.8 | 694.4 | 802.2 | 9967.0 |
| 287 | [At5g19010](http://atted.jp/cgi-bin/coex_Ath.cgi?gene=At5g19010&sort=all) | | MPK16 | | | **458.1** | 1280.7 | 1471.9 | 691.9 | 1394.4 | 15758.6 |
| 288 | [At4g36940](http://atted.jp/cgi-bin/coex_Ath.cgi?gene=At4g36940&sort=all) | | NAPRT1 | | | **458.6** | 449.6 | 1198.6 | 2539.2 | 3598.1 | 1851.7 |
| 289 | [At3g26740](http://atted.jp/cgi-bin/coex_Ath.cgi?gene=At3g26740&sort=all) | | CCL | | | **460.0** | 2626.7 | 144.5 | 377.6 | 5736.9 | 19369.9 |
| 290 | [At5g67130](http://atted.jp/cgi-bin/coex_Ath.cgi?gene=At5g67130&sort=all) | | hydrolase | | | **464.0** | 1208.2 | 628.2 | 2287.1 | 1161.8 | 14565.8 |
| 291 | [At1g28320](http://atted.jp/cgi-bin/coex_Ath.cgi?gene=At1g28320&sort=all) | | DEG15 | | | **466.8** | 3738.7 | 552.5 | 942.9 | 550.0 | 5259.2 |
| 292 | [At5g14270](http://atted.jp/cgi-bin/coex_Ath.cgi?gene=At5g14270&sort=all) | | BET9 | | | **470.2** | 1032.0 | 511.4 | 607.3 | 4248.5 | 10800.9 |
| 293 | [At5g02670](http://atted.jp/cgi-bin/coex_Ath.cgi?gene=At5g02670&sort=all) | |  | | | **470.4** | 65.0 | 3140.2 | 1069.0 | 10226.6 | 2733.4 |
| 294 | [At2g05760](http://atted.jp/cgi-bin/coex_Ath.cgi?gene=At2g05760&sort=all) | | permease | | | **476.3** | 831.0 | 1196.7 | 187.2 | 3217.5 | 9607.9 |
| 295 | 254389_s_at | |  | | | **478.1** | 86.9 | 1979.7 | 20084.0 | 5066.1 | 2199.6 |
| 296 | [At1g10130](http://atted.jp/cgi-bin/coex_Ath.cgi?gene=At1g10130&sort=all) | | ECA3 | | | **478.8** | 1032.3 | 748.8 | 4924.6 | 1612.7 | 5591.1 |
| 297 | [At3g13340](http://atted.jp/cgi-bin/coex_Ath.cgi?gene=At3g13340&sort=all) | | WD-40 repeat | | | **478.9** | 4586.8 | 125.9 | 1258.8 | 957.9 | 1006.2 |
| 298 | [At1g75090](http://atted.jp/cgi-bin/coex_Ath.cgi?gene=At1g75090&sort=all) | | glycosylase | | | **479.8** | 778.8 | 1000.1 | 1879.2 | 14166.8 | 2980.1 |
| 299 | [At3g08550](http://atted.jp/cgi-bin/coex_Ath.cgi?gene=At3g08550&sort=all) | | KOB1 | | | **480.3** | 422.1 | 537.0 | 1913.9 | 5216.5 | 5288.0 |
| 300 | [At3g49140](http://atted.jp/cgi-bin/coex_Ath.cgi?gene=At3g49140&sort=all) | |  | | | **480.5** | 658.6 | 4238.9 | 1329.2 | 3707.7 | 4751.9 |
|  | | | | | | | | | | | |
| **300 coexpressed gene with At1g19320** | | | | | | | | | | | |
|  | | **locus** | | **Short description** | | **MR**  **(all)** | **MR**  **(tissue)** | **MR**  **(abiotic)** | **MR**  **(biotic)** | **MR**  **(hormone)** | **MR**  **(light)** |
| 1 | | [At5g52500](http://atted.jp/cgi-bin/coex_Ath.cgi?gene=At5g52500&sort=all) | |  | | **5.5** | 1.0 | 2045.3 | 6402.4 | 1649.7 | 10203.1 |
| 2 | | [At1g36340](http://atted.jp/cgi-bin/coex_Ath.cgi?gene=At1g36340&sort=all) | | UBC31 | | **9.2** | 9.5 | 469.9 | 1342.2 | 6061.3 | 1280.3 |
| 3 | | [At3g50990](http://atted.jp/cgi-bin/coex_Ath.cgi?gene=At3g50990&sort=all) | | peroxidase | | **11.4** | 13.5 | 1995.8 | 1166.1 | 3116.5 | 1895.8 |
| 4 | | [At2g36710](http://atted.jp/cgi-bin/coex_Ath.cgi?gene=At2g36710&sort=all) | | pectinesterase | | **11.8** | 2.5 | 7543.0 | 1914.9 | 3009.2 | 15498.2 |
| 5 | | [At2g32370](http://atted.jp/cgi-bin/coex_Ath.cgi?gene=At2g32370&sort=all) | | HDG3 | | **14.7** | 3.2 | 380.9 | 3851.2 | 2222.9 | 1374.7 |
| 6 | | [At5g10220](http://atted.jp/cgi-bin/coex_Ath.cgi?gene=At5g10220&sort=all) | | ANN6 | | **15.5** | 22.6 | 770.6 | 3614.1 | 6665.9 | 2880.9 |
| 7 | | [At3g18180](http://atted.jp/cgi-bin/coex_Ath.cgi?gene=At3g18180&sort=all) | | transferase | | **19.3** | 10.6 | 3170.0 | 220.0 | 13274.0 | 602.0 |
| 8 | | [At1g50650](http://atted.jp/cgi-bin/coex_Ath.cgi?gene=At1g50650&sort=all) | | Stig1 | | **20.9** | 22.9 | 1309.0 | 5040.2 | 4513.2 | 8360.0 |
| 9 | | [At5g11220](http://atted.jp/cgi-bin/coex_Ath.cgi?gene=At5g11220&sort=all) | |  | | **21.6** | 1.7 | 4239.8 | 6376.9 | 3445.2 | 11921.2 |
| 10 | | [At1g09550](http://atted.jp/cgi-bin/coex_Ath.cgi?gene=At1g09550&sort=all) | | pectinacetylesterase | | **24.2** | 12.7 | 1634.4 | 2757.7 | 2523.8 | 511.8 |
| 11 | | [At2g14690](http://atted.jp/cgi-bin/coex_Ath.cgi?gene=At2g14690&sort=all) | | hydrolase | | **24.6** | 6.0 | 1342.7 | 5591.6 | 11165.5 | 1160.0 |
| 12 | | [At5g05030](http://atted.jp/cgi-bin/coex_Ath.cgi?gene=At5g05030&sort=all) | |  | | **26.5** | 7.4 | 4955.4 | 4306.3 | 5493.4 | 7051.9 |
| 13 | | [At1g59930](http://atted.jp/cgi-bin/coex_Ath.cgi?gene=At1g59930&sort=all) | |  | | **29.1** | 10.8 | 2417.6 | 3712.4 | 16685.1 | 7063.9 |
| 14 | | [At1g62340](http://atted.jp/cgi-bin/coex_Ath.cgi?gene=At1g62340&sort=all) | | ALE1 | | **29.1** | 15.8 | 2926.0 | 5921.1 | 4483.4 | 2363.3 |
| 15 | | [At1g72580](http://atted.jp/cgi-bin/coex_Ath.cgi?gene=At1g72580&sort=all) | |  | | **30.1** | 15.9 | 1441.1 | 5530.8 | 760.2 | 11384.5 |
| 16 | | [At2g44250](http://atted.jp/cgi-bin/coex_Ath.cgi?gene=At2g44250&sort=all) | |  | | **31.0** | 8.5 | 5312.3 | 5841.2 | 1378.2 | 1388.7 |
| 17 | | [At4g29620](http://atted.jp/cgi-bin/coex_Ath.cgi?gene=At4g29620&sort=all) | | hydrolase | | **32.5** | 14.3 | 3661.2 | 2425.8 | 1420.6 | 119.3 |
| 18 | | [At2g27700](http://atted.jp/cgi-bin/coex_Ath.cgi?gene=At2g27700&sort=all) | | translation | | **33.6** | 19.0 | 3044.5 | 4749.2 | 6218.6 | 6859.9 |
| 19 | | [At4g08530](http://atted.jp/cgi-bin/coex_Ath.cgi?gene=At4g08530&sort=all) | | LTP | | **34.5** | 6.9 | 3908.0 | 4700.7 | 2125.9 | 394.5 |
| 20 | | [At1g28170](http://atted.jp/cgi-bin/coex_Ath.cgi?gene=At1g28170&sort=all) | | transferase | | **35.8** | 3.7 | 11514.2 | 9672.2 | 3789.6 | 10988.5 |
| 21 | | [At2g04750](http://atted.jp/cgi-bin/coex_Ath.cgi?gene=At2g04750&sort=all) | | fimbrin-like | | **35.9** | 8.1 | 2377.0 | 6647.3 | 1434.7 | 10594.2 |
| 22 | | [264934_at](http://atted.jp/cgi-bin/coex_Ath.cgi?gene=264934_at&sort=all) | |  | | **37.8** | 35.1 | 2463.2 | 1257.2 | 2054.5 | 1685.9 |
| 23 | | [At4g27550](http://atted.jp/cgi-bin/coex_Ath.cgi?gene=At4g27550&sort=all) | | TPS4 | | **38.1** | 17.4 | 1040.1 | 2831.6 | 1894.1 | 4357.4 |
| 24 | | [At1g59670](http://atted.jp/cgi-bin/coex_Ath.cgi?gene=At1g59670&sort=all) | | GSTU15 | | **40.2** | 20.1 | 4130.2 | 3479.0 | 11107.1 | 21680.5 |
| 25 | | [At2g15325](http://atted.jp/cgi-bin/coex_Ath.cgi?gene=At2g15325&sort=all) | | LTP | | **42.8** | 42.0 | 2545.6 | 2439.1 | 9367.0 | 13533.9 |
| 26 | | [At5g37730](http://atted.jp/cgi-bin/coex_Ath.cgi?gene=At5g37730&sort=all) | |  | | **43.8** | 15.5 | 9413.4 | 980.0 | 5970.9 | 7685.8 |
| 27 | | [At3g21830](http://atted.jp/cgi-bin/coex_Ath.cgi?gene=At3g21830&sort=all) | | ASK8 | | **44.4** | 43.8 | 5044.8 | 3750.3 | 7767.2 | 2675.2 |
| 28 | | [At4g11180](http://atted.jp/cgi-bin/coex_Ath.cgi?gene=At4g11180&sort=all) | | dirigent | | **45.3** | 50.6 | 5625.9 | 5589.6 | 724.5 | 6059.4 |
| 29 | | [At2g36760](http://atted.jp/cgi-bin/coex_Ath.cgi?gene=At2g36760&sort=all) | | UGT73C2 | | **45.7** | 32.8 | 4884.9 | 2238.7 | 2107.0 | 1363.9 |
| 30 | | [At3g13370](http://atted.jp/cgi-bin/coex_Ath.cgi?gene=At3g13370&sort=all) | |  | | **47.0** | 43.5 | 3062.2 | 4105.4 | 747.5 | 1325.7 |
| 31 | | [At3g08900](http://atted.jp/cgi-bin/coex_Ath.cgi?gene=At3g08900&sort=all) | | RGP3 | | **48.1** | 48.9 | 7468.4 | 2915.1 | 10719.8 | 13437.4 |
| 32 | | [At3g24250](http://atted.jp/cgi-bin/coex_Ath.cgi?gene=At3g24250&sort=all) | | glycine-rich | | **48.2** | 46.4 | 3198.3 | 6667.8 | 3433.4 | 10270.9 |
| 33 | | [At3g25750](http://atted.jp/cgi-bin/coex_Ath.cgi?gene=At3g25750&sort=all) | | F-box | | **49.2** | 29.9 | 2169.4 | 3014.8 | 5766.1 | 1475.1 |
| 34 | | [At5g38960](http://atted.jp/cgi-bin/coex_Ath.cgi?gene=At5g38960&sort=all) | | germin-like | | **50.4** | 5.8 | 3341.2 | 9745.0 | 2824.1 | 4744.2 |
| 35 | | [At4g21080](http://atted.jp/cgi-bin/coex_Ath.cgi?gene=At4g21080&sort=all) | | zinc finger | | **50.9** | 37.1 | 1446.0 | 7439.6 | 3394.1 | 11189.8 |
| 36 | | [At2g18260](http://atted.jp/cgi-bin/coex_Ath.cgi?gene=At2g18260&sort=all) | | SYP112 | | **53.9** | 9.4 | 10738.0 | 9554.9 | 6561.3 | 21219.6 |
| 37 | | 249662_s_at | |  | | **54.1** | 8.9 | 4725.4 | 2723.8 | 8531.6 | 10353.0 |
| 38 | | [At3g52550](http://atted.jp/cgi-bin/coex_Ath.cgi?gene=At3g52550&sort=all) | |  | | **56.3** | 10.3 | 12679.3 | 5154.1 | 433.1 | 5450.9 |
| 39 | | [At4g22430](http://atted.jp/cgi-bin/coex_Ath.cgi?gene=At4g22430&sort=all) | |  | | **57.2** | 37.4 | 6534.6 | 3594.2 | 2579.9 | 16863.3 |
| 40 | | [At2g03190](http://atted.jp/cgi-bin/coex_Ath.cgi?gene=At2g03190&sort=all) | | ASK16 | | **57.5** | 64.0 | 851.1 | 3197.4 | 4732.6 | 11029.2 |
| 41 | | [At2g23570](http://atted.jp/cgi-bin/coex_Ath.cgi?gene=At2g23570&sort=all) | | MES19 | | **58.5** | 18.9 | 533.6 | 4916.9 | 5026.5 | 367.8 |
| 42 | | [At2g23550](http://atted.jp/cgi-bin/coex_Ath.cgi?gene=At2g23550&sort=all) | | MES6 | | **58.5** | 64.2 | 5399.3 | 7964.2 | 6526.0 | 15055.7 |
| 43 | | [At1g31530](http://atted.jp/cgi-bin/coex_Ath.cgi?gene=At1g31530&sort=all) | | phosphatase | | **62.0** | 53.4 | 918.3 | 3648.5 | 4633.3 | 6669.3 |
| 44 | | [At5g45980](http://atted.jp/cgi-bin/coex_Ath.cgi?gene=At5g45980&sort=all) | | WOX8 | | **63.6** | 23.0 | 963.0 | 5770.0 | 5739.6 | 3618.1 |
| 45 | | 261103_s_at | |  | | **63.9** | 49.4 | 2402.3 | 1580.7 | 5106.2 | 9683.4 |
| 46 | | [At5g12460](http://atted.jp/cgi-bin/coex_Ath.cgi?gene=At5g12460&sort=all) | |  | | **64.3** | 60.1 | 629.9 | 6661.9 | 4859.8 | 18050.7 |
| 47 | | [At5g07160](http://atted.jp/cgi-bin/coex_Ath.cgi?gene=At5g07160&sort=all) | | transcription | | **66.5** | 19.6 | 5969.2 | 7726.4 | 3996.2 | 594.4 |
| 48 | | [At1g69860](http://atted.jp/cgi-bin/coex_Ath.cgi?gene=At1g69860&sort=all) | | POT | | **68.2** | 21.4 | 3175.1 | 9760.8 | 2478.7 | 14579.0 |
| 49 | | 262012_s_at | |  | | **69.1** | 38.2 | 4691.7 | 5926.6 | 3579.0 | 14206.8 |
| 50 | | [At2g23580](http://atted.jp/cgi-bin/coex_Ath.cgi?gene=At2g23580&sort=all) | | MES4 | | **69.6** | 86.5 | 3076.5 | 5385.6 | 13133.7 | 1150.8 |
| 51 | | 255261_s_at | |  | | **70.3** | 804.9 | 12305.8 | 60.8 | 13499.1 | 8938.1 |
| 52 | | [At1g28300](http://atted.jp/cgi-bin/coex_Ath.cgi?gene=At1g28300&sort=all) | | LEC2 | | **71.0** | 22.0 | 7997.5 | 5357.0 | 10236.5 | 2008.5 |
| 53 | | [At2g23260](http://atted.jp/cgi-bin/coex_Ath.cgi?gene=At2g23260&sort=all) | | UGT84B1 | | **72.2** | 66.2 | 8322.7 | 4589.6 | 4153.9 | 1206.9 |
| 54 | | [At4g29580](http://atted.jp/cgi-bin/coex_Ath.cgi?gene=At4g29580&sort=all) | | hydrolase | | **75.0** | 22.1 | 3848.9 | 6566.9 | 5244.2 | 1047.4 |
| 55 | | [At3g21840](http://atted.jp/cgi-bin/coex_Ath.cgi?gene=At3g21840&sort=all) | | ASK7 | | **76.2** | 83.1 | 10252.9 | 3552.4 | 5947.2 | 11887.6 |
| 56 | | [At1g71250](http://atted.jp/cgi-bin/coex_Ath.cgi?gene=At1g71250&sort=all) | | hydrolase | | **77.0** | 111.1 | 3510.1 | 2132.2 | 461.2 | 7000.3 |
| 57 | | [At5g13600](http://atted.jp/cgi-bin/coex_Ath.cgi?gene=At5g13600&sort=all) | | NPH3 | | **77.4** | 66.3 | 1577.4 | 4457.8 | 4483.4 | 4026.9 |
| 58 | | [At5g65370](http://atted.jp/cgi-bin/coex_Ath.cgi?gene=At5g65370&sort=all) | | ENTH | | **77.9** | 72.0 | 1976.0 | 4895.5 | 5423.9 | 2924.0 |
| 59 | | [At1g71120](http://atted.jp/cgi-bin/coex_Ath.cgi?gene=At1g71120&sort=all) | | GLIP6 | | **78.7** | 83.8 | 2830.1 | 3166.6 | 3775.2 | 1267.3 |
| 60 | | 253433_s_at | |  | | **79.9** | 87.6 | 6740.6 | 10463.0 | 4860.7 | 16305.3 |
| 61 | | [At5g38180](http://atted.jp/cgi-bin/coex_Ath.cgi?gene=At5g38180&sort=all) | | LTP | | **82.3** | 110.0 | 4078.0 | 7126.3 | 7740.3 | 19379.8 |
| 62 | | [At1g59800](http://atted.jp/cgi-bin/coex_Ath.cgi?gene=At1g59800&sort=all) | | cullin | | **82.8** | 56.6 | 4766.2 | 10393.0 | 1669.2 | 4723.9 |
| 63 | | [At2g20170](http://atted.jp/cgi-bin/coex_Ath.cgi?gene=At2g20170&sort=all) | |  | | **83.9** | 44.4 | 2843.5 | 3938.2 | 2822.2 | 207.2 |
| 64 | | [At1g21970](http://atted.jp/cgi-bin/coex_Ath.cgi?gene=At1g21970&sort=all) | | LEC1 | | **84.3** | 55.7 | 8902.5 | 3909.9 | 1592.6 | 346.0 |
| 65 | | [At3g27785](http://atted.jp/cgi-bin/coex_Ath.cgi?gene=At3g27785&sort=all) | | MYB118 | | **84.5** | 128.5 | 2782.7 | 2079.4 | 1384.1 | 118.3 |
| 66 | | [At5g45770](http://atted.jp/cgi-bin/coex_Ath.cgi?gene=At5g45770&sort=all) | | RLP55 | | **85.2** | 46.3 | 1952.6 | 8799.4 | 2070.1 | 19003.6 |
| 67 | | [At2g21420](http://atted.jp/cgi-bin/coex_Ath.cgi?gene=At2g21420&sort=all) | | zinc finger | | **85.5** | 53.9 | 4168.4 | 3385.3 | 804.8 | 7380.9 |
| 68 | | [At2g25540](http://atted.jp/cgi-bin/coex_Ath.cgi?gene=At2g25540&sort=all) | | CESA10 | | **89.5** | 125.6 | 708.2 | 2860.7 | 730.9 | 19524.0 |
| 69 | | 262589_s_at | |  | | **90.3** | 91.2 | 3770.9 | 8091.8 | 4624.3 | 12223.4 |
| 70 | | [At2g17750](http://atted.jp/cgi-bin/coex_Ath.cgi?gene=At2g17750&sort=all) | | NIP1 | | **91.5** | 34.3 | 7679.1 | 5733.8 | 5903.5 | 5570.8 |
| 71 | | [At1g49800](http://atted.jp/cgi-bin/coex_Ath.cgi?gene=At1g49800&sort=all) | |  | | **92.2** | 51.7 | 2099.8 | 11607.2 | 5173.2 | 363.1 |
| 72 | | [At3g10590](http://atted.jp/cgi-bin/coex_Ath.cgi?gene=At3g10590&sort=all) | | myb | | **94.4** | 71.6 | 14871.9 | 6105.1 | 5867.3 | 1574.5 |
| 73 | | 257411_s_at | |  | | **94.7** | 33.8 | 7504.8 | 3464.6 | 4557.8 | 4428.3 |
| 74 | | [At5g57920](http://atted.jp/cgi-bin/coex_Ath.cgi?gene=At5g57920&sort=all) | | plastocyanin-like | | **95.0** | 54.8 | 7593.1 | 4970.3 | 5800.7 | 19137.3 |
| 75 | | [At2g20160](http://atted.jp/cgi-bin/coex_Ath.cgi?gene=At2g20160&sort=all) | | MEO | | **95.5** | 62.5 | 1213.4 | 7978.1 | 5244.8 | 13318.7 |
| 76 | | [At5g53100](http://atted.jp/cgi-bin/coex_Ath.cgi?gene=At5g53100&sort=all) | | oxidoreductase | | **95.9** | 76.5 | 5308.2 | 11188.1 | 4977.6 | 19854.0 |
| 77 | | [At2g24950](http://atted.jp/cgi-bin/coex_Ath.cgi?gene=At2g24950&sort=all) | |  | | **100.1** | 27.8 | 4590.1 | 6604.5 | 2253.0 | 11993.8 |
| 78 | | [At3g20030](http://atted.jp/cgi-bin/coex_Ath.cgi?gene=At3g20030&sort=all) | | F-box | | **101.5** | 56.3 | 4562.1 | 5407.0 | 9386.2 | 20702.1 |
| 79 | | [At5g50750](http://atted.jp/cgi-bin/coex_Ath.cgi?gene=At5g50750&sort=all) | | RGP4 | | **102.7** | 161.0 | 2089.8 | 1299.3 | 4008.8 | 2320.7 |
| 80 | | [At2g38890](http://atted.jp/cgi-bin/coex_Ath.cgi?gene=At2g38890&sort=all) | |  | | **103.7** | 40.8 | 1472.3 | 4105.6 | 7999.6 | 5014.6 |
| 81 | | [258234_at](http://atted.jp/cgi-bin/coex_Ath.cgi?gene=258234_at&sort=all) | |  | | **104.5** | 97.1 | 3046.2 | 12122.2 | 5520.3 | 8049.8 |
| 82 | | [At3g28360](http://atted.jp/cgi-bin/coex_Ath.cgi?gene=At3g28360&sort=all) | | PGP16 | | **105.3** | 112.4 | 7683.1 | 3273.8 | 16322.7 | 1151.4 |
| 83 | | [At1g62070](http://atted.jp/cgi-bin/coex_Ath.cgi?gene=At1g62070&sort=all) | |  | | **106.4** | 145.5 | 1918.4 | 3122.6 | 3329.7 | 199.9 |
| 84 | | [At1g23200](http://atted.jp/cgi-bin/coex_Ath.cgi?gene=At1g23200&sort=all) | | pectinesterase | | **107.2** | 178.2 | 2270.9 | 534.9 | 458.5 | 856.9 |
| 85 | | [At1g20500](http://atted.jp/cgi-bin/coex_Ath.cgi?gene=At1g20500&sort=all) | | ligase | | **108.3** | 143.4 | 525.4 | 4938.2 | 13798.1 | 1414.4 |
| 86 | | [At3g60700](http://atted.jp/cgi-bin/coex_Ath.cgi?gene=At3g60700&sort=all) | |  | | **110.3** | 52.5 | 1729.3 | 2063.1 | 911.5 | 11275.6 |
| 87 | | [At1g34580](http://atted.jp/cgi-bin/coex_Ath.cgi?gene=At1g34580&sort=all) | | transporter | | **110.7** | 83.4 | 7664.3 | 8539.9 | 12706.4 | 8795.5 |
| 88 | | [At2g35290](http://atted.jp/cgi-bin/coex_Ath.cgi?gene=At2g35290&sort=all) | |  | | **111.0** | 354.8 | 1219.4 | 131.5 | 1251.5 | 13695.0 |
| 89 | | [At5g47670](http://atted.jp/cgi-bin/coex_Ath.cgi?gene=At5g47670&sort=all) | | NF-YB6 | | **111.4** | 156.8 | 1416.9 | 1804.5 | 4521.4 | 773.3 |
| 90 | | [At3g21860](http://atted.jp/cgi-bin/coex_Ath.cgi?gene=At3g21860&sort=all) | | ASK10 | | **112.4** | 43.0 | 7836.8 | 7806.8 | 5032.6 | 5302.2 |
| 91 | | [At1g68510](http://atted.jp/cgi-bin/coex_Ath.cgi?gene=At1g68510&sort=all) | | LBD42 | | **115.0** | 104.5 | 2152.4 | 6446.4 | 5745.1 | 149.2 |
| 92 | | [At5g27200](http://atted.jp/cgi-bin/coex_Ath.cgi?gene=At5g27200&sort=all) | | ACP5 | | **115.1** | 141.4 | 2917.9 | 5107.5 | 1139.1 | 2490.3 |
| 93 | | [At4g22400](http://atted.jp/cgi-bin/coex_Ath.cgi?gene=At4g22400&sort=all) | |  | | **115.6** | 84.0 | 1061.0 | 4612.9 | 12014.6 | 1585.8 |
| 94 | | [At1g71690](http://atted.jp/cgi-bin/coex_Ath.cgi?gene=At1g71690&sort=all) | |  | | **115.9** | 137.2 | 4744.4 | 2039.1 | 1637.6 | 15319.4 |
| 95 | | [At3g42830](http://atted.jp/cgi-bin/coex_Ath.cgi?gene=At3g42830&sort=all) | | Roc1/Rbx1/Hrt1 | | **117.7** | 89.7 | 1518.9 | 2114.2 | 4417.2 | 16740.8 |
| 96 | | [At5g63070](http://atted.jp/cgi-bin/coex_Ath.cgi?gene=At5g63070&sort=all) | | S15 | | **122.0** | 70.4 | 5739.2 | 16318.1 | 4571.7 | 14895.6 |
| 97 | | [248226_at](http://atted.jp/cgi-bin/coex_Ath.cgi?gene=248226_at&sort=all) | |  | | **124.0** | 467.2 | 14.4 | 294.3 | 13000.5 | 956.8 |
| 98 | | [At4g17660](http://atted.jp/cgi-bin/coex_Ath.cgi?gene=At4g17660&sort=all) | | kinase | | **124.7** | 21.0 | 8188.7 | 9897.9 | 4683.7 | 1889.0 |
| 99 | | [At5g08460](http://atted.jp/cgi-bin/coex_Ath.cgi?gene=At5g08460&sort=all) | | hydrolase | | **125.1** | 153.9 | 9315.1 | 4071.8 | 496.8 | 7228.1 |
| 100 | | [At5g22810](http://atted.jp/cgi-bin/coex_Ath.cgi?gene=At5g22810&sort=all) | | lipase | | **125.4** | 146.1 | 3938.6 | 4752.1 | 7641.5 | 10625.5 |
| 101 | | [At1g11070](http://atted.jp/cgi-bin/coex_Ath.cgi?gene=At1g11070&sort=all) | |  | | **126.0** | 45.0 | 4057.9 | 14163.3 | 1606.1 | 13007.4 |
| 102 | | [At4g09820](http://atted.jp/cgi-bin/coex_Ath.cgi?gene=At4g09820&sort=all) | | TT8 | | **127.6** | 145.9 | 4439.7 | 3861.8 | 11540.4 | 1165.1 |
| 103 | | [At4g01140](http://atted.jp/cgi-bin/coex_Ath.cgi?gene=At4g01140&sort=all) | |  | | **128.4** | 375.4 | 3072.9 | 321.6 | 8440.6 | 11544.8 |
| 104 | | [At2g32750](http://atted.jp/cgi-bin/coex_Ath.cgi?gene=At2g32750&sort=all) | | exostosin | | **132.2** | 127.4 | 8139.1 | 2331.7 | 3009.1 | 11356.0 |
| 105 | | [At1g11590](http://atted.jp/cgi-bin/coex_Ath.cgi?gene=At1g11590&sort=all) | | methylesterase | | **132.9** | 147.5 | 4051.0 | 3281.1 | 800.0 | 2056.9 |
| 106 | | [At3g14760](http://atted.jp/cgi-bin/coex_Ath.cgi?gene=At3g14760&sort=all) | |  | | **134.0** | 82.7 | 16787.3 | 9538.7 | 2411.6 | 3512.6 |
| 107 | | [At4g30590](http://atted.jp/cgi-bin/coex_Ath.cgi?gene=At4g30590&sort=all) | | plastocyanin-like | | **134.7** | 119.8 | 3362.9 | 3734.1 | 7176.2 | 2106.3 |
| 108 | | [At2g17310](http://atted.jp/cgi-bin/coex_Ath.cgi?gene=At2g17310&sort=all) | | SON1 | | **135.7** | 27.1 | 1351.4 | 10477.6 | 3011.9 | 15317.1 |
| 109 | | [At4g39650](http://atted.jp/cgi-bin/coex_Ath.cgi?gene=At4g39650&sort=all) | | GGT2 | | **136.5** | 62.3 | 622.1 | 4006.8 | 6871.2 | 3561.9 |
| 110 | | [At5g55360](http://atted.jp/cgi-bin/coex_Ath.cgi?gene=At5g55360&sort=all) | | synthase | | **142.0** | 80.7 | 213.8 | 5298.7 | 4801.4 | 17777.9 |
| 111 | | [At5g60630](http://atted.jp/cgi-bin/coex_Ath.cgi?gene=At5g60630&sort=all) | |  | | **142.4** | 70.4 | 11579.4 | 9491.6 | 5480.9 | 1019.3 |
| 112 | | [At1g16980](http://atted.jp/cgi-bin/coex_Ath.cgi?gene=At1g16980&sort=all) | | TPS2 | | **144.9** | 158.3 | 838.9 | 9177.5 | 2095.0 | 18467.1 |
| 113 | | [249492_at](http://atted.jp/cgi-bin/coex_Ath.cgi?gene=249492_at&sort=all) | |  | | **147.4** | 133.8 | 10014.6 | 3881.7 | 6770.4 | 204.5 |
| 114 | | [At1g64290](http://atted.jp/cgi-bin/coex_Ath.cgi?gene=At1g64290&sort=all) | | F-box | | **149.7** | 77.4 | 3059.4 | 7303.6 | 3163.2 | 4747.1 |
| 115 | | [At2g43050](http://atted.jp/cgi-bin/coex_Ath.cgi?gene=At2g43050&sort=all) | | PMEPCRD | | **151.7** | 147.8 | 3573.9 | 3194.8 | 12454.9 | 2641.4 |
| 116 | | 250894_s_at | |  | | **153.2** | 171.6 | 10300.0 | 3839.7 | 7815.3 | 4219.8 |
| 117 | | [At1g78390](http://atted.jp/cgi-bin/coex_Ath.cgi?gene=At1g78390&sort=all) | | NCED9 | | **157.0** | 197.1 | 1804.7 | 6251.6 | 1614.3 | 548.3 |
| 118 | | [At1g28590](http://atted.jp/cgi-bin/coex_Ath.cgi?gene=At1g28590&sort=all) | | lipase | | **157.5** | 154.3 | 15386.1 | 11850.0 | 21110.8 | 7627.1 |
| 119 | | 264614_s_at | |  | | **158.5** | 160.1 | 4519.3 | 3181.2 | 7066.5 | 9742.4 |
| 120 | | [At3g52970](http://atted.jp/cgi-bin/coex_Ath.cgi?gene=At3g52970&sort=all) | | CYP76G1 | | **159.4** | 146.3 | 7765.0 | 6170.8 | 2589.3 | 2895.4 |
| 121 | | [At3g21850](http://atted.jp/cgi-bin/coex_Ath.cgi?gene=At3g21850&sort=all) | | ASK9 | | **160.4** | 90.7 | 15595.3 | 7674.7 | 20871.0 | 21238.2 |
| 122 | | [At4g33600](http://atted.jp/cgi-bin/coex_Ath.cgi?gene=At4g33600&sort=all) | |  | | **163.1** | 189.9 | 3715.0 | 3941.1 | 13988.3 | 82.8 |
| 123 | | [At2g25220](http://atted.jp/cgi-bin/coex_Ath.cgi?gene=At2g25220&sort=all) | | kinase | | **163.3** | 64.7 | 7902.9 | 11684.6 | 9837.0 | 6624.7 |
| 124 | | [At5g39130](http://atted.jp/cgi-bin/coex_Ath.cgi?gene=At5g39130&sort=all) | | germin-like | | **165.1** | 156.6 | 20894.3 | 3996.1 | 4803.7 | 7655.0 |
| 125 | | [At5g41800](http://atted.jp/cgi-bin/coex_Ath.cgi?gene=At5g41800&sort=all) | | transporter | | **166.6** | 1055.6 | 2360.5 | 81.0 | 10954.4 | 7843.0 |
| 126 | | [At2g28650](http://atted.jp/cgi-bin/coex_Ath.cgi?gene=At2g28650&sort=all) | | EXO70H8 | | **166.9** | 178.1 | 6296.6 | 10016.2 | 5251.8 | 16228.0 |
| 127 | | [At1g26680](http://atted.jp/cgi-bin/coex_Ath.cgi?gene=At1g26680&sort=all) | | transcription | | **168.4** | 187.3 | 8316.8 | 2140.0 | 6467.0 | 14139.7 |
| 128 | | [At4g29570](http://atted.jp/cgi-bin/coex_Ath.cgi?gene=At4g29570&sort=all) | | hydrolase | | **169.8** | 109.5 | 5078.2 | 2529.4 | 11971.7 | 3339.5 |
| 129 | | [At3g21620](http://atted.jp/cgi-bin/coex_Ath.cgi?gene=At3g21620&sort=all) | | ERD | | **170.4** | 71.6 | 6448.9 | 5002.4 | 11504.6 | 6164.2 |
| 130 | | [At1g27080](http://atted.jp/cgi-bin/coex_Ath.cgi?gene=At1g27080&sort=all) | | NRT1.6 | | **170.9** | 183.3 | 1391.9 | 4891.1 | 8228.8 | 8339.9 |
| 131 | | [At5g26120](http://atted.jp/cgi-bin/coex_Ath.cgi?gene=At5g26120&sort=all) | | ASD2 | | **174.9** | 223.8 | 1244.5 | 3946.8 | 5618.9 | 1319.9 |
| 132 | | [At3g29300](http://atted.jp/cgi-bin/coex_Ath.cgi?gene=At3g29300&sort=all) | |  | | **175.5** | 93.4 | 11909.0 | 5351.1 | 3344.8 | 4952.0 |
| 133 | | [253507_at](http://atted.jp/cgi-bin/coex_Ath.cgi?gene=253507_at&sort=all) | |  | | **176.8** | 95.6 | 8787.3 | 9125.2 | 7883.9 | 20605.0 |
| 134 | | [At2g29370](http://atted.jp/cgi-bin/coex_Ath.cgi?gene=At2g29370&sort=all) | | dehydrogenase | | **177.2** | 45.9 | 9718.1 | 8754.4 | 14758.8 | 14028.1 |
| 135 | | [At1g56100](http://atted.jp/cgi-bin/coex_Ath.cgi?gene=At1g56100&sort=all) | | inhibitor | | **180.8** | 197.6 | 4098.2 | 5352.8 | 6646.2 | 17131.1 |
| 136 | | [At5g38170](http://atted.jp/cgi-bin/coex_Ath.cgi?gene=At5g38170&sort=all) | | LTP | | **181.8** | 226.5 | 10732.2 | 5440.4 | 9683.2 | 13875.2 |
| 137 | | 267139_s_at | |  | | **184.7** | 62.6 | 15046.9 | 7385.5 | 2856.5 | 8376.9 |
| 138 | | [At1g04380](http://atted.jp/cgi-bin/coex_Ath.cgi?gene=At1g04380&sort=all) | | dioxygenase | | **185.1** | 229.5 | 11985.2 | 7814.5 | 4911.1 | 18550.2 |
| 139 | | [At2g46960](http://atted.jp/cgi-bin/coex_Ath.cgi?gene=At2g46960&sort=all) | | CYP709B1 | | **185.2** | 253.5 | 5344.0 | 4043.2 | 983.5 | 2297.3 |
| 140 | | [At4g19460](http://atted.jp/cgi-bin/coex_Ath.cgi?gene=At4g19460&sort=all) | | transferase | | **185.6** | 140.6 | 2886.0 | 4583.5 | 8583.3 | 13354.6 |
| 141 | | [At3g13540](http://atted.jp/cgi-bin/coex_Ath.cgi?gene=At3g13540&sort=all) | | MYB5 | | **188.0** | 216.1 | 6730.6 | 2669.1 | 2878.8 | 10640.6 |
| 142 | | [At5g52330](http://atted.jp/cgi-bin/coex_Ath.cgi?gene=At5g52330&sort=all) | | MATH | | **189.6** | 193.3 | 3521.9 | 3778.7 | 34.3 | 10030.4 |
| 143 | | [At1g61720](http://atted.jp/cgi-bin/coex_Ath.cgi?gene=At1g61720&sort=all) | | BAN | | **192.1** | 236.5 | 1168.6 | 2226.4 | 5524.8 | 7149.5 |
| 144 | | [At5g13670](http://atted.jp/cgi-bin/coex_Ath.cgi?gene=At5g13670&sort=all) | | MtN21 | | **192.4** | 192.4 | 2701.5 | 9424.8 | 1119.7 | 9837.3 |
| 145 | | [At4g03930](http://atted.jp/cgi-bin/coex_Ath.cgi?gene=At4g03930&sort=all) | | pectinesterase | | **193.2** | 273.2 | 2334.2 | 5321.0 | 7395.3 | 7168.6 |
| 146 | | [At3g56520](http://atted.jp/cgi-bin/coex_Ath.cgi?gene=At3g56520&sort=all) | | NAM | | **193.7** | 64.2 | 3387.7 | 8246.7 | 3646.6 | 1170.6 |
| 147 | | 264735_s_at | |  | | **195.4** | 284.0 | 814.1 | 2590.8 | 3912.9 | 7540.5 |
| 148 | | [At1g09370](http://atted.jp/cgi-bin/coex_Ath.cgi?gene=At1g09370&sort=all) | | pectinesterase | | **196.0** | 202.1 | 5347.5 | 7764.3 | 8791.5 | 8914.4 |
| 149 | | [At4g33330](http://atted.jp/cgi-bin/coex_Ath.cgi?gene=At4g33330&sort=all) | | PGSIP3 | | **196.9** | 262.6 | 5280.1 | 2409.2 | 4152.8 | 454.9 |
| 150 | | [At3g17600](http://atted.jp/cgi-bin/coex_Ath.cgi?gene=At3g17600&sort=all) | | IAA31 | | **197.3** | 211.1 | 2459.9 | 8725.2 | 9718.0 | 22038.9 |
| 151 | | [At2g42860](http://atted.jp/cgi-bin/coex_Ath.cgi?gene=At2g42860&sort=all) | |  | | **199.0** | 275.4 | 285.1 | 3423.9 | 4381.2 | 1575.7 |
| 152 | | [At5g07260](http://atted.jp/cgi-bin/coex_Ath.cgi?gene=At5g07260&sort=all) | | homeobox | | **199.7** | 255.4 | 387.4 | 7259.1 | 12563.4 | 2048.7 |
| 153 | | [At1g04645](http://atted.jp/cgi-bin/coex_Ath.cgi?gene=At1g04645&sort=all) | | self-incompatibility | | **201.4** | 216.0 | 14012.9 | 3296.4 | 3746.3 | 8838.1 |
| 154 | | [At4g28380](http://atted.jp/cgi-bin/coex_Ath.cgi?gene=At4g28380&sort=all) | | LRR | | **203.6** | 330.2 | 3167.8 | 2538.7 | 2440.4 | 3275.0 |
| 155 | | [At4g23070](http://atted.jp/cgi-bin/coex_Ath.cgi?gene=At4g23070&sort=all) | | RBL7 | | **205.4** | 850.8 | 3339.2 | 137.6 | 6036.1 | 4305.8 |
| 156 | | [At5g24900](http://atted.jp/cgi-bin/coex_Ath.cgi?gene=At5g24900&sort=all) | | CYP714A2 | | **205.9** | 132.5 | 12362.1 | 3613.3 | 5787.8 | 326.9 |
| 157 | | [At3g11180](http://atted.jp/cgi-bin/coex_Ath.cgi?gene=At3g11180&sort=all) | | oxygenase | | **206.0** | 281.1 | 11282.2 | 1838.3 | 6025.3 | 3738.9 |
| 158 | | [At1g71691](http://atted.jp/cgi-bin/coex_Ath.cgi?gene=At1g71691&sort=all) | | hydrolase | | **207.5** | 260.1 | 3680.1 | 5222.5 | 11195.8 | 5693.1 |
| 159 | | [At5g51480](http://atted.jp/cgi-bin/coex_Ath.cgi?gene=At5g51480&sort=all) | | SKS2 | | **210.3** | 271.2 | 1909.0 | 526.7 | 3895.3 | 6985.6 |
| 160 | | [At2g34890](http://atted.jp/cgi-bin/coex_Ath.cgi?gene=At2g34890&sort=all) | | ligase | | **214.9** | 92.8 | 5286.8 | 2955.2 | 8033.6 | 10972.4 |
| 161 | | [At3g58740](http://atted.jp/cgi-bin/coex_Ath.cgi?gene=At3g58740&sort=all) | | CSY1 | | **215.0** | 290.6 | 3649.3 | 2076.1 | 1962.0 | 1231.5 |
| 162 | | [At3g62610](http://atted.jp/cgi-bin/coex_Ath.cgi?gene=At3g62610&sort=all) | | MYB11 | | **215.1** | 167.7 | 7900.8 | 7871.1 | 9031.7 | 14832.2 |
| 163 | | [At1g20940](http://atted.jp/cgi-bin/coex_Ath.cgi?gene=At1g20940&sort=all) | | F-box | | **216.4** | 62.0 | 7936.5 | 6669.3 | 3233.5 | 7997.5 |
| 164 | | [At1g53500](http://atted.jp/cgi-bin/coex_Ath.cgi?gene=At1g53500&sort=all) | | MUM4 | | **218.7** | 205.0 | 7743.6 | 883.9 | 12258.5 | 12874.1 |
| 165 | | [At1g43640](http://atted.jp/cgi-bin/coex_Ath.cgi?gene=At1g43640&sort=all) | | TLP5 | | **219.0** | 277.9 | 1966.8 | 5724.9 | 2653.1 | 14990.1 |
| 166 | | [At1g21070](http://atted.jp/cgi-bin/coex_Ath.cgi?gene=At1g21070&sort=all) | | transporter | | **220.6** | 234.4 | 12810.2 | 1312.5 | 17529.5 | 17961.6 |
| 167 | | [At1g05280](http://atted.jp/cgi-bin/coex_Ath.cgi?gene=At1g05280&sort=all) | | fringe | | **221.0** | 253.1 | 12512.7 | 3534.9 | 16086.9 | 1859.9 |
| 168 | | [At1g79840](http://atted.jp/cgi-bin/coex_Ath.cgi?gene=At1g79840&sort=all) | | GL2 | | **221.6** | 181.0 | 17670.7 | 9417.8 | 4792.5 | 15673.8 |
| 169 | | [At4g25750](http://atted.jp/cgi-bin/coex_Ath.cgi?gene=At4g25750&sort=all) | | transporter | | **223.7** | 175.9 | 3987.1 | 7952.7 | 10631.3 | 18521.4 |
| 170 | | [At2g28120](http://atted.jp/cgi-bin/coex_Ath.cgi?gene=At2g28120&sort=all) | | nodulin | | **223.7** | 248.5 | 14418.4 | 818.8 | 1281.9 | 9974.9 |
| 171 | | [At5g07200](http://atted.jp/cgi-bin/coex_Ath.cgi?gene=At5g07200&sort=all) | | YAP169 | | **223.9** | 241.4 | 4539.0 | 7141.4 | 4464.5 | 247.6 |
| 172 | | [At3g54320](http://atted.jp/cgi-bin/coex_Ath.cgi?gene=At3g54320&sort=all) | | WRI1 | | **225.5** | 214.6 | 789.9 | 11425.8 | 2769.7 | 1278.9 |
| 173 | | [At5g48100](http://atted.jp/cgi-bin/coex_Ath.cgi?gene=At5g48100&sort=all) | | TT10 | | **225.6** | 332.3 | 1643.4 | 3983.4 | 4431.6 | 596.7 |
| 174 | | [At5g47530](http://atted.jp/cgi-bin/coex_Ath.cgi?gene=At5g47530&sort=all) | | auxin-responsive | | **227.4** | 208.6 | 7758.7 | 3926.1 | 1815.0 | 12302.6 |
| 175 | | [At4g31760](http://atted.jp/cgi-bin/coex_Ath.cgi?gene=At4g31760&sort=all) | | peroxidase | | **227.5** | 152.6 | 881.2 | 6260.9 | 6634.7 | 19390.3 |
| 176 | | [At1g25410](http://atted.jp/cgi-bin/coex_Ath.cgi?gene=At1g25410&sort=all) | | IPT6 | | **227.5** | 322.1 | 1803.8 | 3306.9 | 5820.7 | 584.5 |
| 177 | | [At5g38160](http://atted.jp/cgi-bin/coex_Ath.cgi?gene=At5g38160&sort=all) | | LTP | | **230.1** | 284.9 | 2324.7 | 1612.6 | 8769.6 | 2572.4 |
| 178 | | [At4g15750](http://atted.jp/cgi-bin/coex_Ath.cgi?gene=At4g15750&sort=all) | | inhibitor | | **231.6** | 229.1 | 6307.8 | 11480.7 | 8163.0 | 9287.1 |
| 179 | | [At4g37360](http://atted.jp/cgi-bin/coex_Ath.cgi?gene=At4g37360&sort=all) | | CYP81D2 | | **235.0** | 351.1 | 1306.0 | 2973.3 | 2401.3 | 1163.9 |
| 180 | | [At1g78220](http://atted.jp/cgi-bin/coex_Ath.cgi?gene=At1g78220&sort=all) | | GRF13 | | **235.3** | 119.0 | 2666.1 | 5716.0 | 4243.0 | 14964.5 |
| 181 | | [At3g25260](http://atted.jp/cgi-bin/coex_Ath.cgi?gene=At3g25260&sort=all) | | POT | | **235.7** | 286.4 | 3249.8 | 2827.4 | 1331.3 | 9920.5 |
| 182 | | [At1g80330](http://atted.jp/cgi-bin/coex_Ath.cgi?gene=At1g80330&sort=all) | | GA3OX4 | | **237.0** | 286.7 | 9354.4 | 2007.3 | 1578.2 | 8016.4 |
| 183 | | [At2g47750](http://atted.jp/cgi-bin/coex_Ath.cgi?gene=At2g47750&sort=all) | | GH3.9 | | **241.1** | 269.8 | 8029.8 | 8797.5 | 8055.9 | 4124.1 |
| 184 | | [At5g51210](http://atted.jp/cgi-bin/coex_Ath.cgi?gene=At5g51210&sort=all) | | OLEO3 | | **241.5** | 295.5 | 3808.0 | 2962.1 | 7696.2 | 7619.1 |
| 185 | | [At1g76500](http://atted.jp/cgi-bin/coex_Ath.cgi?gene=At1g76500&sort=all) | | SOB3 | | **243.6** | 233.0 | 6968.8 | 4363.8 | 10773.1 | 8767.2 |
| 186 | | [At4g38000](http://atted.jp/cgi-bin/coex_Ath.cgi?gene=At4g38000&sort=all) | | zinc finger | | **245.3** | 235.5 | 3090.0 | 7495.3 | 2798.0 | 2654.1 |
| 187 | | [At5g59590](http://atted.jp/cgi-bin/coex_Ath.cgi?gene=At5g59590&sort=all) | | UGT76E2 | | **249.3** | 321.6 | 587.8 | 484.2 | 9291.8 | 16385.1 |
| 188 | | [At1g22480](http://atted.jp/cgi-bin/coex_Ath.cgi?gene=At1g22480&sort=all) | | plastocyanin-like | | **249.8** | 343.9 | 1786.2 | 8803.0 | 9745.9 | 721.4 |
| 189 | | [At4g10490](http://atted.jp/cgi-bin/coex_Ath.cgi?gene=At4g10490&sort=all) | | oxygenase | | **250.2** | 315.6 | 314.8 | 2927.8 | 3764.8 | 6289.0 |
| 190 | | [At1g02720](http://atted.jp/cgi-bin/coex_Ath.cgi?gene=At1g02720&sort=all) | | GATL5 | | **251.0** | 207.4 | 11398.8 | 5367.7 | 10599.3 | 20456.1 |
| 191 | | [At5g55320](http://atted.jp/cgi-bin/coex_Ath.cgi?gene=At5g55320&sort=all) | | MBOAT | | **251.1** | 243.8 | 5373.8 | 4740.8 | 5478.5 | 5560.2 |
| 192 | | [At1g61440](http://atted.jp/cgi-bin/coex_Ath.cgi?gene=At1g61440&sort=all) | | kinase | | **251.9** | 109.3 | 8681.1 | 1846.8 | 1783.8 | 6754.9 |
| 193 | | [At1g60970](http://atted.jp/cgi-bin/coex_Ath.cgi?gene=At1g60970&sort=all) | | clathrin adaptor complex | | **252.5** | 320.6 | 5735.3 | 696.5 | 944.1 | 325.9 |
| 194 | | [At3g04200](http://atted.jp/cgi-bin/coex_Ath.cgi?gene=At3g04200&sort=all) | | germin-like | | **254.2** | 434.9 | 1221.7 | 2051.0 | 4793.2 | 1007.0 |
| 195 | | [At5g55950](http://atted.jp/cgi-bin/coex_Ath.cgi?gene=At5g55950&sort=all) | | transporter | | **259.0** | 190.7 | 823.7 | 8745.7 | 1743.9 | 18718.1 |
| 196 | | [At1g72790](http://atted.jp/cgi-bin/coex_Ath.cgi?gene=At1g72790&sort=all) | | glycoprotein | | **259.7** | 577.9 | 11728.2 | 116.5 | 1363.4 | 8549.4 |
| 197 | | [At1g57780](http://atted.jp/cgi-bin/coex_Ath.cgi?gene=At1g57780&sort=all) | | heavy-metal-associated | | **261.1** | 113.8 | 6387.3 | 9496.6 | 8888.1 | 16347.0 |
| 198 | | [At3g12203](http://atted.jp/cgi-bin/coex_Ath.cgi?gene=At3g12203&sort=all) | | scpl17 | | **261.9** | 330.2 | 6070.8 | 4584.0 | 12247.3 | 17890.8 |
| 199 | | 255069_s_at | |  | | **262.3** | 35.4 | 17222.2 | 8511.4 | 7663.1 | 15073.1 |
| 200 | | [At5g18290](http://atted.jp/cgi-bin/coex_Ath.cgi?gene=At5g18290&sort=all) | | SIP1;2 | | **262.5** | 288.4 | 12124.8 | 1031.9 | 12628.5 | 6086.0 |
| 201 | | [At2g45830](http://atted.jp/cgi-bin/coex_Ath.cgi?gene=At2g45830&sort=all) | | DTA2 | | **263.2** | 147.4 | 14631.3 | 5360.1 | 19327.4 | 2270.4 |
| 202 | | [At5g35550](http://atted.jp/cgi-bin/coex_Ath.cgi?gene=At5g35550&sort=all) | | TT2 | | **267.5** | 315.2 | 3459.5 | 3389.5 | 2726.4 | 21616.6 |
| 203 | | [At1g04660](http://atted.jp/cgi-bin/coex_Ath.cgi?gene=At1g04660&sort=all) | | glycine-rich | | **268.3** | 301.7 | 6282.7 | 2463.0 | 13372.4 | 4426.0 |
| 204 | | [At1g19440](http://atted.jp/cgi-bin/coex_Ath.cgi?gene=At1g19440&sort=all) | | KCS4 | | **269.5** | 767.9 | 10212.6 | 1120.3 | 15477.9 | 19327.1 |
| 205 | | [At5g48950](http://atted.jp/cgi-bin/coex_Ath.cgi?gene=At5g48950&sort=all) | | thioesterase | | **271.5** | 408.8 | 2411.9 | 1368.7 | 3693.5 | 501.3 |
| 206 | | [At5g47330](http://atted.jp/cgi-bin/coex_Ath.cgi?gene=At5g47330&sort=all) | | thioesterase | | **275.8** | 241.0 | 2270.3 | 8376.2 | 11723.1 | 18328.2 |
| 207 | | [At4g00220](http://atted.jp/cgi-bin/coex_Ath.cgi?gene=At4g00220&sort=all) | | JLO | | **276.1** | 408.9 | 2518.5 | 5489.4 | 1229.5 | 686.5 |
| 208 | | 245072_s_at | |  | | **277.7** | 391.6 | 3757.0 | 4960.8 | 2303.6 | 4510.7 |
| 209 | | [At2g44470](http://atted.jp/cgi-bin/coex_Ath.cgi?gene=At2g44470&sort=all) | | BGLU29 | | **278.6** | 388.8 | 631.9 | 10165.6 | 1254.0 | 18670.4 |
| 210 | | [At4g27140](http://atted.jp/cgi-bin/coex_Ath.cgi?gene=At4g27140&sort=all) | | 2S seed storage 1 | | **281.2** | 603.8 | 547.5 | 50.8 | 15457.8 | 1174.9 |
| 211 | | [At2g31010](http://atted.jp/cgi-bin/coex_Ath.cgi?gene=At2g31010&sort=all) | | kinase | | **284.1** | 5281.5 | 55.7 | 35.5 | 4972.5 | 13600.7 |
| 212 | | [At1g71890](http://atted.jp/cgi-bin/coex_Ath.cgi?gene=At1g71890&sort=all) | | SUC5 | | **284.2** | 209.4 | 9531.6 | 15729.2 | 18558.3 | 10858.5 |
| 213 | | [At5g47150](http://atted.jp/cgi-bin/coex_Ath.cgi?gene=At5g47150&sort=all) | | YDG | | **286.6** | 377.8 | 12432.8 | 5891.6 | 4738.8 | 8541.5 |
| 214 | | [At4g01500](http://atted.jp/cgi-bin/coex_Ath.cgi?gene=At4g01500&sort=all) | | NGA4 | | **287.9** | 211.1 | 2764.5 | 3674.7 | 3582.2 | 9013.3 |
| 215 | | [At4g32350](http://atted.jp/cgi-bin/coex_Ath.cgi?gene=At4g32350&sort=all) | |  | | **288.5** | 295.1 | 9837.4 | 578.9 | 6986.7 | 11453.1 |
| 216 | | [At1g31290](http://atted.jp/cgi-bin/coex_Ath.cgi?gene=At1g31290&sort=all) | | PAZ | | **288.9** | 108.8 | 10226.6 | 4731.8 | 6918.5 | 20201.3 |
| 217 | | [At4g21050](http://atted.jp/cgi-bin/coex_Ath.cgi?gene=At4g21050&sort=all) | | zinc finger | | **289.0** | 218.5 | 2975.6 | 6286.9 | 14812.5 | 1451.8 |
| 218 | | [At3g49300](http://atted.jp/cgi-bin/coex_Ath.cgi?gene=At3g49300&sort=all) | | proline-rich | | **289.2** | 361.1 | 1100.8 | 1084.7 | 2524.2 | 2345.9 |
| 219 | | [At4g34520](http://atted.jp/cgi-bin/coex_Ath.cgi?gene=At4g34520&sort=all) | | KCS18 | | **290.7** | 428.0 | 6718.3 | 2443.3 | 4212.9 | 18577.0 |
| 220 | | [At1g12550](http://atted.jp/cgi-bin/coex_Ath.cgi?gene=At1g12550&sort=all) | | oxidoreductase | | **291.1** | 293.0 | 7638.7 | 2260.8 | 17438.5 | 6172.5 |
| 221 | | [At1g24540](http://atted.jp/cgi-bin/coex_Ath.cgi?gene=At1g24540&sort=all) | | CYP86C1 | | **292.6** | 370.4 | 3381.6 | 2911.4 | 3791.1 | 15074.8 |
| 222 | | [At5g26250](http://atted.jp/cgi-bin/coex_Ath.cgi?gene=At5g26250&sort=all) | | transporter | | **293.4** | 173.8 | 2175.6 | 4229.0 | 8540.2 | 3815.1 |
| 223 | | [264556_at](http://atted.jp/cgi-bin/coex_Ath.cgi?gene=264556_at&sort=all) | |  | | **295.2** | 348.1 | 8455.8 | 8142.0 | 7115.2 | 6101.9 |
| 224 | | 250612_s_at | |  | | **296.0** | 202.6 | 173.0 | 7539.6 | 2402.7 | 6760.2 |
| 225 | | [At5g09370](http://atted.jp/cgi-bin/coex_Ath.cgi?gene=At5g09370&sort=all) | | LTP | | **296.9** | 322.8 | 3240.1 | 6636.5 | 4927.2 | 13377.7 |
| 226 | | [At3g19240](http://atted.jp/cgi-bin/coex_Ath.cgi?gene=At3g19240&sort=all) | |  | | **297.3** | 516.0 | 1120.8 | 470.5 | 8258.5 | 11254.9 |
| 227 | | [At1g53690](http://atted.jp/cgi-bin/coex_Ath.cgi?gene=At1g53690&sort=all) | | RNA polymerase | | **298.0** | 280.9 | 4412.3 | 6072.0 | 16665.5 | 7547.2 |
| 228 | | [At2g45420](http://atted.jp/cgi-bin/coex_Ath.cgi?gene=At2g45420&sort=all) | | LBD18 | | **298.7** | 246.9 | 10794.3 | 5428.2 | 2843.3 | 1619.3 |
| 229 | | [At2g38900](http://atted.jp/cgi-bin/coex_Ath.cgi?gene=At2g38900&sort=all) | | I-type | | **300.4** | 413.0 | 445.5 | 5161.9 | 2031.9 | 245.3 |
| 230 | | [At2g34700](http://atted.jp/cgi-bin/coex_Ath.cgi?gene=At2g34700&sort=all) | | extensin | | **302.5** | 397.7 | 742.7 | 4904.5 | 18648.8 | 3630.5 |
| 231 | | [At5g49190](http://atted.jp/cgi-bin/coex_Ath.cgi?gene=At5g49190&sort=all) | | SUS2 | | **304.9** | 438.2 | 5845.2 | 7446.1 | 4769.5 | 1417.8 |
| 232 | | [At1g19190](http://atted.jp/cgi-bin/coex_Ath.cgi?gene=At1g19190&sort=all) | | hydrolase | | **308.3** | 304.0 | 12877.7 | 4588.6 | 8486.3 | 11349.2 |
| 233 | | [At5g60820](http://atted.jp/cgi-bin/coex_Ath.cgi?gene=At5g60820&sort=all) | | zinc finger | | **309.2** | 1013.6 | 6605.1 | 541.6 | 10459.0 | 18418.3 |
| 234 | | [At1g27910](http://atted.jp/cgi-bin/coex_Ath.cgi?gene=At1g27910&sort=all) | | PUB45 | | **315.4** | 4375.0 | 866.8 | 18.7 | 6938.1 | 9093.2 |
| 235 | | [At3g01570](http://atted.jp/cgi-bin/coex_Ath.cgi?gene=At3g01570&sort=all) | | oleosin | | **315.5** | 452.0 | 1575.3 | 3666.6 | 8952.4 | 1341.5 |
| 236 | | [At5g38195](http://atted.jp/cgi-bin/coex_Ath.cgi?gene=At5g38195&sort=all) | | LTP | | **317.0** | 440.8 | 5887.1 | 9355.5 | 7069.5 | 6261.4 |
| 237 | | [At3g03230](http://atted.jp/cgi-bin/coex_Ath.cgi?gene=At3g03230&sort=all) | | thioesterase | | **317.5** | 382.4 | 640.7 | 3002.5 | 6575.6 | 6234.9 |
| 238 | | [At5g65710](http://atted.jp/cgi-bin/coex_Ath.cgi?gene=At5g65710&sort=all) | | HSL2 | | **319.8** | 228.0 | 14975.4 | 15471.8 | 13116.8 | 10530.8 |
| 239 | | [At3g63040](http://atted.jp/cgi-bin/coex_Ath.cgi?gene=At3g63040&sort=all) | |  | | **322.8** | 473.7 | 1554.9 | 4830.6 | 5903.8 | 747.4 |
| 240 | | [At1g72220](http://atted.jp/cgi-bin/coex_Ath.cgi?gene=At1g72220&sort=all) | | zinc finger | | **323.4** | 427.8 | 6190.6 | 2347.5 | 11787.3 | 1577.5 |
| 241 | | [At3g02590](http://atted.jp/cgi-bin/coex_Ath.cgi?gene=At3g02590&sort=all) | | desaturase | | **323.7** | 326.8 | 9518.8 | 5303.2 | 5143.8 | 18132.4 |
| 242 | | [At2g27380](http://atted.jp/cgi-bin/coex_Ath.cgi?gene=At2g27380&sort=all) | | EPR1 | | **324.4** | 534.5 | 2619.0 | 3706.6 | 2781.7 | 3462.2 |
| 243 | | [At1g54955](http://atted.jp/cgi-bin/coex_Ath.cgi?gene=At1g54955&sort=all) | | transposable | | **325.0** | 244.8 | 2119.9 | 3785.4 | 8992.3 | 15116.2 |
| 244 | | [At5g51490](http://atted.jp/cgi-bin/coex_Ath.cgi?gene=At5g51490&sort=all) | | pectinesterase | | **329.1** | 433.0 | 17045.0 | 3218.9 | 12207.1 | 4028.1 |
| 245 | | [At5g59170](http://atted.jp/cgi-bin/coex_Ath.cgi?gene=At5g59170&sort=all) | | proline-rich | | **329.2** | 536.5 | 2492.4 | 2372.6 | 2188.0 | 2567.5 |
| 246 | | [At4g26590](http://atted.jp/cgi-bin/coex_Ath.cgi?gene=At4g26590&sort=all) | | OPT5 | | **329.7** | 443.5 | 1387.2 | 7501.8 | 4828.8 | 127.2 |
| 247 | | [At1g47540](http://atted.jp/cgi-bin/coex_Ath.cgi?gene=At1g47540&sort=all) | | inhibitor | | **331.7** | 458.5 | 1306.8 | 4484.4 | 17347.7 | 2276.4 |
| 248 | | [At1g28030](http://atted.jp/cgi-bin/coex_Ath.cgi?gene=At1g28030&sort=all) | | oxygenase | | **333.7** | 600.1 | 3490.1 | 433.5 | 6920.9 | 2845.9 |
| 249 | | [At4g01420](http://atted.jp/cgi-bin/coex_Ath.cgi?gene=At4g01420&sort=all) | | CBL5 | | **335.2** | 190.5 | 4337.8 | 3837.6 | 13282.2 | 20368.0 |
| 250 | | [At2g47670](http://atted.jp/cgi-bin/coex_Ath.cgi?gene=At2g47670&sort=all) | | inhibitor | | **335.6** | 311.7 | 4501.4 | 14013.6 | 5580.8 | 4976.5 |
| 251 | | [At5g62110](http://atted.jp/cgi-bin/coex_Ath.cgi?gene=At5g62110&sort=all) | | DNA binding | | **335.7** | 125.9 | 15813.8 | 13294.3 | 3350.1 | 12982.5 |
| 252 | | [At3g45130](http://atted.jp/cgi-bin/coex_Ath.cgi?gene=At3g45130&sort=all) | | LAS1 | | **336.0** | 149.1 | 2037.7 | 14898.9 | 6381.3 | 11062.5 |
| 253 | | [At3g56260](http://atted.jp/cgi-bin/coex_Ath.cgi?gene=At3g56260&sort=all) | |  | | **339.3** | 705.0 | 583.5 | 1411.3 | 3703.3 | 12417.3 |
| 254 | | [At3g49380](http://atted.jp/cgi-bin/coex_Ath.cgi?gene=At3g49380&sort=all) | | iqd15 | | **339.4** | 185.7 | 15102.3 | 3175.8 | 10966.1 | 12076.7 |
| 255 | | [At1g11960](http://atted.jp/cgi-bin/coex_Ath.cgi?gene=At1g11960&sort=all) | |  | | **343.1** | 254.8 | 1537.6 | 162.2 | 13698.8 | 7748.0 |
| 256 | | [At5g44360](http://atted.jp/cgi-bin/coex_Ath.cgi?gene=At5g44360&sort=all) | | FAD-binding | | **345.0** | 499.1 | 3919.7 | 4522.4 | 7144.1 | 2360.8 |
| 257 | | [At2g21510](http://atted.jp/cgi-bin/coex_Ath.cgi?gene=At2g21510&sort=all) | | N-terminal | | **345.6** | 431.3 | 2986.3 | 3105.7 | 14326.3 | 9077.3 |
| 258 | | [At5g25470](http://atted.jp/cgi-bin/coex_Ath.cgi?gene=At5g25470&sort=all) | | DNA binding | | **349.6** | 460.9 | 9447.5 | 1199.0 | 7996.6 | 9337.3 |
| 259 | | [266388_at](http://atted.jp/cgi-bin/coex_Ath.cgi?gene=266388_at&sort=all) | |  | | **349.9** | 95.7 | 8371.5 | 15656.8 | 2517.4 | 20494.5 |
| 260 | | [At1g05160](http://atted.jp/cgi-bin/coex_Ath.cgi?gene=At1g05160&sort=all) | | CYP88A3 | | **350.0** | 210.2 | 10466.3 | 18663.1 | 18194.7 | 1502.0 |
| 261 | | [At5g40420](http://atted.jp/cgi-bin/coex_Ath.cgi?gene=At5g40420&sort=all) | | OLEO2 | | **350.1** | 419.5 | 2259.7 | 7172.4 | 17312.0 | 7487.1 |
| 262 | | [At5g19700](http://atted.jp/cgi-bin/coex_Ath.cgi?gene=At5g19700&sort=all) | | MATE efflux | | **350.5** | 142.1 | 4291.9 | 2238.3 | 1037.9 | 389.8 |
| 263 | | 257366_s_at | |  | | **353.4** | 233.2 | 5506.5 | 10283.6 | 5093.8 | 4363.5 |
| 264 | | [At4g28520](http://atted.jp/cgi-bin/coex_Ath.cgi?gene=At4g28520&sort=all) | | CRU3 | | **354.0** | 449.8 | 3111.6 | 6884.3 | 14472.8 | 258.9 |
| 265 | | [At3g48950](http://atted.jp/cgi-bin/coex_Ath.cgi?gene=At3g48950&sort=all) | | pectinase | | **356.3** | 208.6 | 3825.6 | 4198.3 | 3614.0 | 1436.7 |
| 266 | | [At2g01770](http://atted.jp/cgi-bin/coex_Ath.cgi?gene=At2g01770&sort=all) | | VIT1 | | **357.3** | 533.0 | 4171.7 | 4949.0 | 2317.6 | 9441.2 |
| 267 | | [At5g03795](http://atted.jp/cgi-bin/coex_Ath.cgi?gene=At5g03795&sort=all) | |  | | **358.2** | 591.3 | 2952.6 | 2337.6 | 2740.6 | 366.7 |
| 268 | | [At4g35985](http://atted.jp/cgi-bin/coex_Ath.cgi?gene=At4g35985&sort=all) | | senescence/dehydration-associated | | **358.6** | 1169.8 | 1747.8 | 97.6 | 12607.7 | 9616.6 |
| 269 | | [At5g60130](http://atted.jp/cgi-bin/coex_Ath.cgi?gene=At5g60130&sort=all) | | transcription | | **359.5** | 109.6 | 7406.5 | 8855.1 | 2591.2 | 21442.1 |
| 270 | | [At4g31900](http://atted.jp/cgi-bin/coex_Ath.cgi?gene=At4g31900&sort=all) | | PKR2 | | **360.5** | 125.9 | 2234.5 | 9333.3 | 4973.3 | 710.4 |
| 271 | | [At1g01900](http://atted.jp/cgi-bin/coex_Ath.cgi?gene=At1g01900&sort=all) | | SBTI1.1 | | **361.4** | 282.2 | 9288.3 | 14918.1 | 1214.9 | 12021.5 |
| 272 | | [At1g07120](http://atted.jp/cgi-bin/coex_Ath.cgi?gene=At1g07120&sort=all) | |  | | **362.1** | 567.8 | 6987.4 | 2986.7 | 6926.2 | 8509.1 |
| 273 | | [At4g38410](http://atted.jp/cgi-bin/coex_Ath.cgi?gene=At4g38410&sort=all) | | dehydrin | | **365.7** | 272.1 | 3489.5 | 2993.0 | 12168.3 | 7444.2 |
| 274 | | [At4g12890](http://atted.jp/cgi-bin/coex_Ath.cgi?gene=At4g12890&sort=all) | | GILT | | **366.6** | 431.6 | 4947.4 | 4431.7 | 12805.1 | 5489.7 |
| 275 | | [At1g32770](http://atted.jp/cgi-bin/coex_Ath.cgi?gene=At1g32770&sort=all) | | ANAC012 | | **368.4** | 523.6 | 14731.3 | 6803.4 | 11193.0 | 8682.3 |
| 276 | | [At5g44120](http://atted.jp/cgi-bin/coex_Ath.cgi?gene=At5g44120&sort=all) | | CRA1 | | **368.5** | 404.7 | 5319.5 | 4437.1 | 18466.9 | 1279.8 |
| 277 | | [At4g36700](http://atted.jp/cgi-bin/coex_Ath.cgi?gene=At4g36700&sort=all) | | cupin | | **369.2** | 344.6 | 4354.0 | 15515.1 | 15440.9 | 5605.5 |
| 278 | | [At3g20210](http://atted.jp/cgi-bin/coex_Ath.cgi?gene=At3g20210&sort=all) | | DELTA-VPE | | **369.8** | 397.6 | 3965.7 | 9204.2 | 4659.6 | 14817.2 |
| 279 | | [At3g25280](http://atted.jp/cgi-bin/coex_Ath.cgi?gene=At3g25280&sort=all) | | POT | | **371.6** | 38.5 | 2906.9 | 8087.6 | 5698.3 | 18246.4 |
| 280 | | [At5g57520](http://atted.jp/cgi-bin/coex_Ath.cgi?gene=At5g57520&sort=all) | | ZFP2 | | **373.5** | 408.8 | 9068.3 | 8183.5 | 8633.0 | 10310.9 |
| 281 | | [At1g63300](http://atted.jp/cgi-bin/coex_Ath.cgi?gene=At1g63300&sort=all) | |  | | **374.1** | 269.8 | 22580.4 | 11434.5 | 12485.6 | 20708.4 |
| 282 | | [At5g49180](http://atted.jp/cgi-bin/coex_Ath.cgi?gene=At5g49180&sort=all) | | pectinesterase | | **375.8** | 483.8 | 3882.8 | 4385.2 | 3908.0 | 2093.3 |
| 283 | | [At2g27690](http://atted.jp/cgi-bin/coex_Ath.cgi?gene=At2g27690&sort=all) | | CYP94C1 | | **376.5** | 890.0 | 3699.5 | 158.2 | 8457.0 | 1115.2 |
| 284 | | [At4g25140](http://atted.jp/cgi-bin/coex_Ath.cgi?gene=At4g25140&sort=all) | | OLEO1 | | **376.8** | 519.0 | 4006.2 | 5091.9 | 12312.0 | 301.7 |
| 285 | | [At4g09480](http://atted.jp/cgi-bin/coex_Ath.cgi?gene=At4g09480&sort=all) | | transposable | | **378.9** | 283.3 | 266.4 | 10163.0 | 8635.3 | 15269.4 |
| 286 | | [At3g13900](http://atted.jp/cgi-bin/coex_Ath.cgi?gene=At3g13900&sort=all) | | ATPase | | **379.9** | 595.9 | 474.8 | 3176.7 | 2506.8 | 5512.9 |
| 287 | | [At4g29640](http://atted.jp/cgi-bin/coex_Ath.cgi?gene=At4g29640&sort=all) | | hydrolase | | **380.4** | 180.6 | 4885.4 | 4503.8 | 2153.0 | 735.9 |
| 288 | | [At5g62800](http://atted.jp/cgi-bin/coex_Ath.cgi?gene=At5g62800&sort=all) | | SINA | | **380.9** | 530.7 | 5454.6 | 4863.8 | 3089.6 | 4805.7 |
| 289 | | [At1g65480](http://atted.jp/cgi-bin/coex_Ath.cgi?gene=At1g65480&sort=all) | | FT | | **381.1** | 332.3 | 17922.6 | 13511.9 | 6263.8 | 1291.0 |
| 290 | | [At4g27150](http://atted.jp/cgi-bin/coex_Ath.cgi?gene=At4g27150&sort=all) | | 2S seed storage 2 | | **384.0** | 547.2 | 830.5 | 1856.2 | 15107.2 | 416.2 |
| 291 | | [At4g27160](http://atted.jp/cgi-bin/coex_Ath.cgi?gene=At4g27160&sort=all) | | AT2S3 | | **385.1** | 549.2 | 3971.2 | 1224.5 | 14179.1 | 385.1 |
| 292 | | [At1g03880](http://atted.jp/cgi-bin/coex_Ath.cgi?gene=At1g03880&sort=all) | | CRU2 | | **385.8** | 577.9 | 5383.1 | 2266.2 | 16259.0 | 2364.6 |
| 293 | | [At1g48910](http://atted.jp/cgi-bin/coex_Ath.cgi?gene=At1g48910&sort=all) | | YUC10 | | **390.9** | 624.4 | 1480.9 | 7270.2 | 1964.4 | 579.9 |
| 294 | | [At5g58500](http://atted.jp/cgi-bin/coex_Ath.cgi?gene=At5g58500&sort=all) | | LSH5 | | **391.1** | 412.8 | 16041.5 | 8503.7 | 8912.0 | 13665.5 |
| 295 | | 251055_at | |  | | **391.9** | 951.9 | 11710.9 | 167.5 | 12567.7 | 10590.8 |
| 296 | | [At4g37950](http://atted.jp/cgi-bin/coex_Ath.cgi?gene=At4g37950&sort=all) | | lyase | | **395.4** | 225.4 | 501.0 | 4378.4 | 10027.5 | 1352.5 |
| 297 | | [At1g15010](http://atted.jp/cgi-bin/coex_Ath.cgi?gene=At1g15010&sort=all) | |  | | **395.5** | 593.7 | 1740.0 | 308.6 | 5032.9 | 14387.7 |
| 298 | | [At1g70140](http://atted.jp/cgi-bin/coex_Ath.cgi?gene=At1g70140&sort=all) | | ATFH8 | | **397.1** | 817.4 | 4643.1 | 758.8 | 15388.2 | 16619.2 |
| 299 | | [At1g47610](http://atted.jp/cgi-bin/coex_Ath.cgi?gene=At1g47610&sort=all) | | transducin | | **397.4** | 491.6 | 4694.2 | 3600.5 | 3602.2 | 4827.8 |
| 300 | | [At1g75250](http://atted.jp/cgi-bin/coex_Ath.cgi?gene=At1g75250&sort=all) | | ATRL6 | | **397.5** | 442.2 | 8297.6 | 2716.5 | 1851.2 | 525.7 |
|  | | | | | |  | | | | | |
| **300 coexpressed gene with At1g20030** | | | | | | | | | | | |
|  | | **locus** | | **Short description** | **MR**  **(all)** | | **MR**  **(tissue)** | **MR**  **(abiotic)** | **MR**  **(biotic)** | **MR**  **(hormone)** | **MR**  **(light)** |
| 1 | | [At5g14960](http://atted.jp/cgi-bin/coex_Ath.cgi?gene=At5g14960&sort=all) | | DEL2 | **3.0** | | 1057.0 | 16.4 | 742.8 | 3142.0 | 18499.3 |
| 2 | | [At4g23040](http://atted.jp/cgi-bin/coex_Ath.cgi?gene=At4g23040&sort=all) | | UBX | **3.5** | | 2158.6 | 6.9 | 31.0 | 13078.9 | 4066.1 |
| 3 | | [At1g64890](http://atted.jp/cgi-bin/coex_Ath.cgi?gene=At1g64890&sort=all) | | transporter | **4.1** | | 2969.5 | 18.1 | 43.1 | 161.7 | 2074.2 |
| 4 | | [At2g31360](http://atted.jp/cgi-bin/coex_Ath.cgi?gene=At2g31360&sort=all) | | ADS2 | **4.6** | | 529.0 | 81.6 | 1.0 | 18804.6 | 13793.9 |
| 5 | | [At1g79460](http://atted.jp/cgi-bin/coex_Ath.cgi?gene=At1g79460&sort=all) | | GA2 | **4.9** | | 897.0 | 201.1 | 207.3 | 1828.3 | 5746.5 |
| 6 | | [At1g22770](http://atted.jp/cgi-bin/coex_Ath.cgi?gene=At1g22770&sort=all) | | GI | **6.6** | | 4649.5 | 2.8 | 56.7 | 7386.7 | 21394.3 |
| 7 | | [At4g18270](http://atted.jp/cgi-bin/coex_Ath.cgi?gene=At4g18270&sort=all) | | TRANS11 | **8.5** | | 11273.0 | 1.7 | 5.9 | 706.8 | 12717.7 |
| 8 | | [At1g51610](http://atted.jp/cgi-bin/coex_Ath.cgi?gene=At1g51610&sort=all) | | MTPc4 | **9.4** | | 3994.1 | 23.1 | 12.3 | 16948.7 | 5723.8 |
| 9 | | [At4g09500](http://atted.jp/cgi-bin/coex_Ath.cgi?gene=At4g09500&sort=all) | | transferase | **12.5** | | 2532.0 | 21.0 | 58.2 | 139.7 | 12777.9 |
| 10 | | [At5g24470](http://atted.jp/cgi-bin/coex_Ath.cgi?gene=At5g24470&sort=all) | | APRR5 | **13.4** | | 8221.4 | 32.1 | 1.4 | 3225.2 | 18137.8 |
| 11 | | [At4g18520](http://atted.jp/cgi-bin/coex_Ath.cgi?gene=At4g18520&sort=all) | |  | **15.9** | | 2924.0 | 89.9 | 30.2 | 5836.7 | 15095.9 |
| 12 | | [At2g22080](http://atted.jp/cgi-bin/coex_Ath.cgi?gene=At2g22080&sort=all) | |  | **18.3** | | 1977.9 | 80.9 | 733.5 | 5216.7 | 4754.0 |
| 13 | | [At3g55760](http://atted.jp/cgi-bin/coex_Ath.cgi?gene=At3g55760&sort=all) | |  | **19.1** | | 8037.9 | 11.6 | 77.4 | 1547.6 | 8999.5 |
| 14 | | [At4g25500](http://atted.jp/cgi-bin/coex_Ath.cgi?gene=At4g25500&sort=all) | | RSP35 | **19.8** | | 3863.7 | 18.0 | 856.0 | 16095.5 | 8277.0 |
| 15 | | [At1g78600](http://atted.jp/cgi-bin/coex_Ath.cgi?gene=At1g78600&sort=all) | | LZF1 | **21.1** | | 10254.9 | 34.4 | 51.0 | 1005.8 | 19751.8 |
| 16 | | [At1g47710](http://atted.jp/cgi-bin/coex_Ath.cgi?gene=At1g47710&sort=all) | | protease | **24.7** | | 5504.4 | 24.6 | 1974.2 | 16750.9 | 17134.5 |
| 17 | | [At1g19370](http://atted.jp/cgi-bin/coex_Ath.cgi?gene=At1g19370&sort=all) | |  | **26.3** | | 8302.6 | 32.6 | 93.8 | 21208.2 | 7738.2 |
| 18 | | [At5g57110](http://atted.jp/cgi-bin/coex_Ath.cgi?gene=At5g57110&sort=all) | | ACA8 | **26.6** | | 4817.5 | 100.1 | 205.0 | 1423.2 | 17378.5 |
| 19 | | [At5g61810](http://atted.jp/cgi-bin/coex_Ath.cgi?gene=At5g61810&sort=all) | | mitochondrial substrate carrier | **26.7** | | 5124.7 | 13.9 | 2603.9 | 74.9 | 16306.5 |
| 20 | | [At4g36010](http://atted.jp/cgi-bin/coex_Ath.cgi?gene=At4g36010&sort=all) | | pathogenesis-related thaumatin | **27.9** | | 982.0 | 99.6 | 1173.8 | 2197.4 | 3191.8 |
| 21 | | [At5g18540](http://atted.jp/cgi-bin/coex_Ath.cgi?gene=At5g18540&sort=all) | |  | **29.9** | | 2373.1 | 141.2 | 294.8 | 2392.5 | 20489.3 |
| 22 | | [At4g35300](http://atted.jp/cgi-bin/coex_Ath.cgi?gene=At4g35300&sort=all) | | TMT2 | **34.0** | | 7368.1 | 82.2 | 16.6 | 653.3 | 10151.0 |
| 23 | | [At3g56720](http://atted.jp/cgi-bin/coex_Ath.cgi?gene=At3g56720&sort=all) | |  | **34.0** | | 2505.8 | 121.7 | 202.8 | 14280.3 | 5507.0 |
| 24 | | [At1g51090](http://atted.jp/cgi-bin/coex_Ath.cgi?gene=At1g51090&sort=all) | | heavy-metal-associated | **36.5** | | 17687.1 | 45.7 | 55.5 | 239.5 | 9573.2 |
| 25 | | [At4g38580](http://atted.jp/cgi-bin/coex_Ath.cgi?gene=At4g38580&sort=all) | | ATFP6 | **37.0** | | 5398.4 | 36.4 | 190.8 | 17600.1 | 8861.9 |
| 26 | | [At5g45610](http://atted.jp/cgi-bin/coex_Ath.cgi?gene=At5g45610&sort=all) | | SUV2 | **37.4** | | 2876.9 | 108.3 | 109.5 | 15649.9 | 18790.9 |
| 27 | | [At4g01130](http://atted.jp/cgi-bin/coex_Ath.cgi?gene=At4g01130&sort=all) | | acetylesterase | **38.3** | | 3684.3 | 91.2 | 58.8 | 587.0 | 19567.8 |
| 28 | | [At1g49720](http://atted.jp/cgi-bin/coex_Ath.cgi?gene=At1g49720&sort=all) | | ABF1 | **39.4** | | 14601.5 | 83.9 | 78.8 | 1989.2 | 20442.6 |
| 29 | | [At4g18390](http://atted.jp/cgi-bin/coex_Ath.cgi?gene=At4g18390&sort=all) | | TCP2 | **40.2** | | 4653.9 | 16.6 | 15.0 | 1162.7 | 19041.1 |
| 30 | | [At1g07420](http://atted.jp/cgi-bin/coex_Ath.cgi?gene=At1g07420&sort=all) | | SMO2-1 | **41.6** | | 7284.4 | 127.2 | 10.9 | 1984.3 | 7346.1 |
| 31 | | [At1g49560](http://atted.jp/cgi-bin/coex_Ath.cgi?gene=At1g49560&sort=all) | | myb | **43.0** | | 10210.0 | 60.7 | 45.9 | 13836.7 | 15904.4 |
| 32 | | [At2g21130](http://atted.jp/cgi-bin/coex_Ath.cgi?gene=At2g21130&sort=all) | | CYP2 | **44.4** | | 3739.1 | 100.9 | 455.0 | 2132.2 | 17370.5 |
| 33 | | [At1g27630](http://atted.jp/cgi-bin/coex_Ath.cgi?gene=At1g27630&sort=all) | | CYCT1;3 | **44.7** | | 14563.9 | 58.8 | 192.7 | 9212.4 | 11445.3 |
| 34 | | [At4g14230](http://atted.jp/cgi-bin/coex_Ath.cgi?gene=At4g14230&sort=all) | | CBS | **44.7** | | 13438.3 | 87.6 | 49.8 | 10855.7 | 4786.3 |
| 35 | | [At4g04340](http://atted.jp/cgi-bin/coex_Ath.cgi?gene=At4g04340&sort=all) | | ERD | **47.0** | | 4483.2 | 112.4 | 62.3 | 12250.2 | 18291.4 |
| 36 | | [At1g62430](http://atted.jp/cgi-bin/coex_Ath.cgi?gene=At1g62430&sort=all) | | CDS1 | **47.1** | | 7986.0 | 75.8 | 15.2 | 240.7 | 1496.0 |
| 37 | | [At2g02100](http://atted.jp/cgi-bin/coex_Ath.cgi?gene=At2g02100&sort=all) | | LCR69 | **47.7** | | 1327.0 | 113.0 | 322.1 | 15531.3 | 18710.4 |
| 38 | | [At1g12845](http://atted.jp/cgi-bin/coex_Ath.cgi?gene=At1g12845&sort=all) | |  | **47.8** | | 4072.7 | 82.6 | 28.5 | 141.5 | 2133.2 |
| 39 | | [At2g22830](http://atted.jp/cgi-bin/coex_Ath.cgi?gene=At2g22830&sort=all) | | SQE2 | **49.4** | | 7090.2 | 71.5 | 211.8 | 6648.0 | 2825.8 |
| 40 | | [At5g06830](http://atted.jp/cgi-bin/coex_Ath.cgi?gene=At5g06830&sort=all) | |  | **49.5** | | 4405.5 | 40.6 | 769.1 | 11686.8 | 14271.7 |
| 41 | | [At3g08880](http://atted.jp/cgi-bin/coex_Ath.cgi?gene=At3g08880&sort=all) | |  | **49.7** | | 2536.1 | 113.6 | 841.9 | 1790.1 | 12223.5 |
| 42 | | [At2g38670](http://atted.jp/cgi-bin/coex_Ath.cgi?gene=At2g38670&sort=all) | | PECT1 | **49.8** | | 1132.1 | 178.5 | 168.5 | 9709.1 | 8415.7 |
| 43 | | [At5g02860](http://atted.jp/cgi-bin/coex_Ath.cgi?gene=At5g02860&sort=all) | | PPR | **49.8** | | 4849.2 | 46.9 | 251.1 | 16320.4 | 15654.5 |
| 44 | | [At5g05840](http://atted.jp/cgi-bin/coex_Ath.cgi?gene=At5g05840&sort=all) | |  | **50.1** | | 6054.1 | 76.6 | 341.2 | 13530.2 | 17971.9 |
| 45 | | [At4g28320](http://atted.jp/cgi-bin/coex_Ath.cgi?gene=At4g28320&sort=all) | | cellulase | **50.6** | | 2039.2 | 216.8 | 1518.8 | 10285.0 | 14190.1 |
| 46 | | [At5g14270](http://atted.jp/cgi-bin/coex_Ath.cgi?gene=At5g14270&sort=all) | | BET9 | **51.2** | | 7481.8 | 80.3 | 882.8 | 18428.4 | 5720.1 |
| 47 | | [At2g42530](http://atted.jp/cgi-bin/coex_Ath.cgi?gene=At2g42530&sort=all) | | COR15B | **51.2** | | 10069.0 | 12.7 | 344.1 | 45.2 | 1958.8 |
| 48 | | [At1g12710](http://atted.jp/cgi-bin/coex_Ath.cgi?gene=At1g12710&sort=all) | | PP2-A12 | **56.6** | | 17985.1 | 55.2 | 60.0 | 3175.5 | 8804.5 |
| 49 | | [At4g39260](http://atted.jp/cgi-bin/coex_Ath.cgi?gene=At4g39260&sort=all) | | GR-RBP8 | **59.0** | | 4667.1 | 110.6 | 277.5 | 22601.6 | 1489.8 |
| 50 | | [At4g25480](http://atted.jp/cgi-bin/coex_Ath.cgi?gene=At4g25480&sort=all) | | DREB1A | **59.8** | | 11767.5 | 205.4 | 7.8 | 378.5 | 16022.1 |
| 51 | | [At1g56300](http://atted.jp/cgi-bin/coex_Ath.cgi?gene=At1g56300&sort=all) | | N-terminal | **61.7** | | 11441.8 | 80.7 | 111.8 | 758.5 | 18528.5 |
| 52 | | [At2g39920](http://atted.jp/cgi-bin/coex_Ath.cgi?gene=At2g39920&sort=all) | | acid phosphatase class B | **61.7** | | 16219.0 | 35.2 | 28.2 | 6381.0 | 10823.2 |
| 53 | | 246481_s_at | |  | **61.8** | | 7845.9 | 59.6 | 1190.4 | 387.5 | 15833.1 |
| 54 | | [At5g39410](http://atted.jp/cgi-bin/coex_Ath.cgi?gene=At5g39410&sort=all) | | binding | **63.9** | | 4809.2 | 119.7 | 908.3 | 10705.9 | 19778.1 |
| 55 | | [At2g26200](http://atted.jp/cgi-bin/coex_Ath.cgi?gene=At2g26200&sort=all) | |  | **65.4** | | 3061.4 | 131.1 | 60.6 | 19742.8 | 4765.2 |
| 56 | | [At3g55580](http://atted.jp/cgi-bin/coex_Ath.cgi?gene=At3g55580&sort=all) | | RCC1 | **65.7** | | 6146.1 | 266.1 | 18.4 | 4377.0 | 17928.2 |
| 57 | | [At3g24515](http://atted.jp/cgi-bin/coex_Ath.cgi?gene=At3g24515&sort=all) | | UBC37 | **66.5** | | 3521.9 | 108.4 | 7.5 | 6604.9 | 10992.3 |
| 58 | | [At3g15040](http://atted.jp/cgi-bin/coex_Ath.cgi?gene=At3g15040&sort=all) | |  | **67.7** | | 10155.3 | 31.4 | 246.7 | 562.3 | 221.9 |
| 59 | | [At5g10790](http://atted.jp/cgi-bin/coex_Ath.cgi?gene=At5g10790&sort=all) | | UBP22 | **68.8** | | 16226.2 | 33.5 | 3644.5 | 4145.2 | 1585.0 |
| 60 | | [At3g58570](http://atted.jp/cgi-bin/coex_Ath.cgi?gene=At3g58570&sort=all) | | helicase | **69.6** | | 1872.0 | 119.4 | 2421.8 | 19071.0 | 1560.4 |
| 61 | | [At3g53990](http://atted.jp/cgi-bin/coex_Ath.cgi?gene=At3g53990&sort=all) | | USP | **70.5** | | 16914.7 | 1.4 | 70.3 | 1121.1 | 5987.8 |
| 62 | | [At5g44210](http://atted.jp/cgi-bin/coex_Ath.cgi?gene=At5g44210&sort=all) | | ERF9 | **70.6** | | 154.1 | 206.0 | 270.6 | 19718.8 | 11193.6 |
| 63 | | 266772_s_at | |  | **70.8** | | 1910.2 | 326.2 | 10625.2 | 10357.1 | 14494.2 |
| 64 | | [At3g05030](http://atted.jp/cgi-bin/coex_Ath.cgi?gene=At3g05030&sort=all) | | NHX2 | **72.2** | | 2358.7 | 476.5 | 98.6 | 4109.5 | 15417.2 |
| 65 | | [At1g43620](http://atted.jp/cgi-bin/coex_Ath.cgi?gene=At1g43620&sort=all) | | UGT80B1 | **73.5** | | 18069.9 | 66.2 | 53.7 | 1790.3 | 7335.4 |
| 66 | | [At1g53035](http://atted.jp/cgi-bin/coex_Ath.cgi?gene=At1g53035&sort=all) | |  | **77.4** | | 9059.4 | 73.3 | 99.6 | 21105.7 | 17218.5 |
| 67 | | [At3g60980](http://atted.jp/cgi-bin/coex_Ath.cgi?gene=At3g60980&sort=all) | | PPR | **77.4** | | 10168.9 | 28.7 | 1348.3 | 15770.7 | 9072.6 |
| 68 | | [At1g11210](http://atted.jp/cgi-bin/coex_Ath.cgi?gene=At1g11210&sort=all) | |  | **77.9** | | 16816.8 | 31.3 | 527.5 | 706.5 | 17072.8 |
| 69 | | [At4g30660](http://atted.jp/cgi-bin/coex_Ath.cgi?gene=At4g30660&sort=all) | | hydrophobic | **78.8** | | 4652.8 | 118.9 | 135.3 | 417.6 | 232.6 |
| 70 | | [At1g70420](http://atted.jp/cgi-bin/coex_Ath.cgi?gene=At1g70420&sort=all) | |  | **80.4** | | 17336.6 | 14.3 | 89.8 | 353.1 | 19478.8 |
| 71 | | [At2g22840](http://atted.jp/cgi-bin/coex_Ath.cgi?gene=At2g22840&sort=all) | | GRF1 | **83.4** | | 76.7 | 92.3 | 862.8 | 10113.3 | 13527.9 |
| 72 | | [At5g62640](http://atted.jp/cgi-bin/coex_Ath.cgi?gene=At5g62640&sort=all) | | ELF5 | **84.6** | | 2378.9 | 110.3 | 5656.3 | 20209.5 | 8845.8 |
| 73 | | [At1g32860](http://atted.jp/cgi-bin/coex_Ath.cgi?gene=At1g32860&sort=all) | | hydrolase | **88.4** | | 2198.7 | 91.4 | 828.7 | 7988.7 | 14302.9 |
| 74 | | [At2g40130](http://atted.jp/cgi-bin/coex_Ath.cgi?gene=At2g40130&sort=all) | | heat shock | **89.1** | | 7418.1 | 30.2 | 3048.3 | 917.4 | 15410.3 |
| 75 | | 265244_at | |  | **90.2** | | 10631.2 | 22.9 | 1169.0 | 839.2 | 11003.0 |
| 76 | | [At1g28070](http://atted.jp/cgi-bin/coex_Ath.cgi?gene=At1g28070&sort=all) | |  | **90.3** | | 4838.1 | 431.6 | 124.3 | 1624.4 | 3062.3 |
| 77 | | [At5g62910](http://atted.jp/cgi-bin/coex_Ath.cgi?gene=At5g62910&sort=all) | | protein binding | **93.1** | | 8129.9 | 103.2 | 387.9 | 14936.0 | 5988.0 |
| 78 | | [At2g22450](http://atted.jp/cgi-bin/coex_Ath.cgi?gene=At2g22450&sort=all) | | riboflavin biosynthesis | **93.5** | | 6657.5 | 185.9 | 642.8 | 15655.1 | 12722.1 |
| 79 | | [At2g25950](http://atted.jp/cgi-bin/coex_Ath.cgi?gene=At2g25950&sort=all) | |  | **93.7** | | 7800.5 | 181.7 | 112.4 | 7841.2 | 17941.0 |
| 80 | | [At2g19450](http://atted.jp/cgi-bin/coex_Ath.cgi?gene=At2g19450&sort=all) | | TAG1 | **98.2** | | 14766.8 | 52.5 | 1161.0 | 21574.8 | 9795.6 |
| 81 | | [At5g52310](http://atted.jp/cgi-bin/coex_Ath.cgi?gene=At5g52310&sort=all) | | LTI78 | **98.4** | | 17617.8 | 148.8 | 189.2 | 490.5 | 14809.3 |
| 82 | | [At3g26290](http://atted.jp/cgi-bin/coex_Ath.cgi?gene=At3g26290&sort=all) | | CYP71B26 | **98.9** | | 1742.0 | 831.2 | 105.2 | 692.2 | 12666.2 |
| 83 | | [At2g34200](http://atted.jp/cgi-bin/coex_Ath.cgi?gene=At2g34200&sort=all) | | zinc finger | **99.2** | | 3899.8 | 255.3 | 514.3 | 10366.3 | 3167.8 |
| 84 | | [At1g67660](http://atted.jp/cgi-bin/coex_Ath.cgi?gene=At1g67660&sort=all) | | nuclease | **101.8** | | 4783.4 | 186.5 | 87.4 | 13474.7 | 8693.8 |
| 85 | | [At4g30650](http://atted.jp/cgi-bin/coex_Ath.cgi?gene=At4g30650&sort=all) | | hydrophobic | **102.2** | | 7218.2 | 118.9 | 739.8 | 3154.5 | 8737.4 |
| 86 | | [At2g34090](http://atted.jp/cgi-bin/coex_Ath.cgi?gene=At2g34090&sort=all) | | MEE18 | **103.3** | | 9856.6 | 82.7 | 21.2 | 7526.4 | 11884.5 |
| 87 | | [At2g21660](http://atted.jp/cgi-bin/coex_Ath.cgi?gene=At2g21660&sort=all) | | CCR2 | **105.9** | | 8017.2 | 144.2 | 645.1 | 19683.0 | 11616.1 |
| 88 | | [At3g05800](http://atted.jp/cgi-bin/coex_Ath.cgi?gene=At3g05800&sort=all) | | AIF1 | **106.4** | | 17122.4 | 58.3 | 177.7 | 7691.4 | 17708.1 |
| 89 | | [At1g49650](http://atted.jp/cgi-bin/coex_Ath.cgi?gene=At1g49650&sort=all) | | cell death associated | **107.1** | | 1342.1 | 257.4 | 1486.7 | 20258.0 | 9384.2 |
| 90 | | 248523_s_at | |  | **108.0** | | 1829.3 | 94.6 | 352.5 | 10313.4 | 9505.7 |
| 91 | | [At4g16146](http://atted.jp/cgi-bin/coex_Ath.cgi?gene=At4g16146&sort=all) | |  | **109.4** | | 11371.5 | 107.8 | 423.7 | 6796.8 | 10059.6 |
| 92 | | [At4g26670](http://atted.jp/cgi-bin/coex_Ath.cgi?gene=At4g26670&sort=all) | | Tim17/Tim22/Tim23 | **112.7** | | 3988.4 | 241.7 | 374.4 | 21438.5 | 13741.0 |
| 93 | | [At2g47850](http://atted.jp/cgi-bin/coex_Ath.cgi?gene=At2g47850&sort=all) | | CCCH-type | **114.5** | | 2018.8 | 467.9 | 973.2 | 19349.5 | 21927.5 |
| 94 | | 259990_s_at | |  | **115.2** | | 11167.1 | 178.4 | 791.5 | 1985.9 | 8111.7 |
| 95 | | [At2g47890](http://atted.jp/cgi-bin/coex_Ath.cgi?gene=At2g47890&sort=all) | | zinc finger | **115.9** | | 16972.1 | 57.2 | 50.8 | 1831.4 | 15265.2 |
| 96 | | [At2g15970](http://atted.jp/cgi-bin/coex_Ath.cgi?gene=At2g15970&sort=all) | | COR413-PM1 | **117.2** | | 14002.1 | 89.0 | 87.5 | 330.3 | 12558.5 |
| 97 | | [At2g28900](http://atted.jp/cgi-bin/coex_Ath.cgi?gene=At2g28900&sort=all) | | OEP16-1 | **119.6** | | 5710.9 | 192.6 | 581.6 | 16220.9 | 11142.6 |
| 98 | | [At1g54830](http://atted.jp/cgi-bin/coex_Ath.cgi?gene=At1g54830&sort=all) | | NF-YC3 | **121.2** | | 8713.6 | 87.7 | 113.5 | 466.3 | 9724.9 |
| 99 | | [At5g58600](http://atted.jp/cgi-bin/coex_Ath.cgi?gene=At5g58600&sort=all) | | PMR5 | **123.7** | | 2896.3 | 105.8 | 101.8 | 17125.5 | 12337.2 |
| 100 | | [At2g28840](http://atted.jp/cgi-bin/coex_Ath.cgi?gene=At2g28840&sort=all) | | ankyrin repeat | **124.4** | | 9457.1 | 65.9 | 1040.2 | 2877.0 | 14691.0 |
| 101 | | [At3g05640](http://atted.jp/cgi-bin/coex_Ath.cgi?gene=At3g05640&sort=all) | | PP2C | **125.4** | | 14197.7 | 160.4 | 523.2 | 73.3 | 8820.3 |
| 102 | | [At2g19810](http://atted.jp/cgi-bin/coex_Ath.cgi?gene=At2g19810&sort=all) | | CCCH-type | **127.6** | | 1252.2 | 611.0 | 3742.5 | 15.0 | 3112.1 |
| 103 | | 250557_at | |  | **127.7** | | 5383.9 | 115.6 | 1647.8 | 13497.6 | 1070.9 |
| 104 | | [At2g01170](http://atted.jp/cgi-bin/coex_Ath.cgi?gene=At2g01170&sort=all) | | BAT1 | **127.9** | | 12856.8 | 103.2 | 105.5 | 20056.3 | 236.5 |
| 105 | | [At1g20070](http://atted.jp/cgi-bin/coex_Ath.cgi?gene=At1g20070&sort=all) | |  | **129.2** | | 5405.0 | 324.8 | 66.5 | 4452.8 | 14778.5 |
| 106 | | [At5g48250](http://atted.jp/cgi-bin/coex_Ath.cgi?gene=At5g48250&sort=all) | | zinc finger | **130.1** | | 18850.8 | 199.0 | 848.4 | 7177.7 | 12058.8 |
| 107 | | [At4g29190](http://atted.jp/cgi-bin/coex_Ath.cgi?gene=At4g29190&sort=all) | | CCCH-type | **131.8** | | 5769.9 | 95.6 | 381.5 | 522.3 | 12860.0 |
| 108 | | [At5g16380](http://atted.jp/cgi-bin/coex_Ath.cgi?gene=At5g16380&sort=all) | |  | **133.4** | | 12522.1 | 55.5 | 582.0 | 13036.5 | 15234.6 |
| 109 | | [At4g29610](http://atted.jp/cgi-bin/coex_Ath.cgi?gene=At4g29610&sort=all) | | hydrolase | **133.9** | | 11116.5 | 46.0 | 889.6 | 11230.8 | 10647.0 |
| 110 | | [At4g14270](http://atted.jp/cgi-bin/coex_Ath.cgi?gene=At4g14270&sort=all) | |  | **135.3** | | 7296.8 | 356.5 | 172.2 | 1247.1 | 11817.5 |
| 111 | | [At1g18360](http://atted.jp/cgi-bin/coex_Ath.cgi?gene=At1g18360&sort=all) | | hydrolase | **135.6** | | 3022.0 | 69.9 | 307.4 | 2205.5 | 97.0 |
| 112 | | [At3g12490](http://atted.jp/cgi-bin/coex_Ath.cgi?gene=At3g12490&sort=all) | | CYSB | **139.2** | | 7215.8 | 42.7 | 3427.0 | 8597.7 | 9788.4 |
| 113 | | [At5g50450](http://atted.jp/cgi-bin/coex_Ath.cgi?gene=At5g50450&sort=all) | | zinc finger | **140.1** | | 7686.9 | 344.8 | 196.8 | 6271.4 | 268.9 |
| 114 | | [At3g08510](http://atted.jp/cgi-bin/coex_Ath.cgi?gene=At3g08510&sort=all) | | PLC2 | **140.6** | | 11316.7 | 211.6 | 109.9 | 2329.5 | 19610.0 |
| 115 | | [At5g16040](http://atted.jp/cgi-bin/coex_Ath.cgi?gene=At5g16040&sort=all) | | RCC1 | **140.7** | | 559.7 | 124.2 | 503.5 | 19446.2 | 13175.7 |
| 116 | | [At4g02340](http://atted.jp/cgi-bin/coex_Ath.cgi?gene=At4g02340&sort=all) | | hydrolase | **144.1** | | 7448.9 | 91.7 | 618.4 | 11885.3 | 11801.0 |
| 117 | | [At1g79440](http://atted.jp/cgi-bin/coex_Ath.cgi?gene=At1g79440&sort=all) | | ALDH5F1 | **144.3** | | 20442.1 | 207.6 | 369.3 | 8845.3 | 9004.7 |
| 118 | | [At5g47020](http://atted.jp/cgi-bin/coex_Ath.cgi?gene=At5g47020&sort=all) | | glycine-rich | **145.0** | | 3841.4 | 363.3 | 334.2 | 434.2 | 8493.1 |
| 119 | | [At2g27200](http://atted.jp/cgi-bin/coex_Ath.cgi?gene=At2g27200&sort=all) | | GTP-binding | **146.2** | | 15226.6 | 31.0 | 5653.0 | 8646.0 | 14396.3 |
| 120 | | [At1g53110](http://atted.jp/cgi-bin/coex_Ath.cgi?gene=At1g53110&sort=all) | |  | **146.3** | | 9736.6 | 346.8 | 486.5 | 3849.7 | 5006.0 |
| 121 | | [At4g18530](http://atted.jp/cgi-bin/coex_Ath.cgi?gene=At4g18530&sort=all) | |  | **148.1** | | 11561.9 | 1.7 | 79.5 | 829.5 | 13951.6 |
| 122 | | [At4g23920](http://atted.jp/cgi-bin/coex_Ath.cgi?gene=At4g23920&sort=all) | | UGE2 | **148.7** | | 6230.3 | 285.0 | 2557.3 | 331.1 | 11992.2 |
| 123 | | [At5g62360](http://atted.jp/cgi-bin/coex_Ath.cgi?gene=At5g62360&sort=all) | | inhibitor | **150.1** | | 13008.1 | 110.6 | 595.3 | 15543.6 | 2094.1 |
| 124 | | [At1g29395](http://atted.jp/cgi-bin/coex_Ath.cgi?gene=At1g29395&sort=all) | | COR414-TM1 | **152.3** | | 18403.0 | 30.7 | 346.5 | 105.6 | 15510.6 |
| 125 | | [At5g54960](http://atted.jp/cgi-bin/coex_Ath.cgi?gene=At5g54960&sort=all) | | PDC2 | **154.8** | | 17070.0 | 152.3 | 631.9 | 17970.5 | 19802.4 |
| 126 | | [At1g25320](http://atted.jp/cgi-bin/coex_Ath.cgi?gene=At1g25320&sort=all) | | kinase | **155.7** | | 1758.0 | 402.8 | 68.0 | 15130.7 | 7251.8 |
| 127 | | [At5g27930](http://atted.jp/cgi-bin/coex_Ath.cgi?gene=At5g27930&sort=all) | | PP2C | **156.5** | | 14788.4 | 59.9 | 150.6 | 1190.1 | 590.5 |
| 128 | | [At5g08500](http://atted.jp/cgi-bin/coex_Ath.cgi?gene=At5g08500&sort=all) | | CLPTM1 | **157.9** | | 8873.9 | 113.5 | 4851.5 | 21977.3 | 12538.2 |
| 129 | | [At1g28050](http://atted.jp/cgi-bin/coex_Ath.cgi?gene=At1g28050&sort=all) | | zinc finger | **158.4** | | 20055.9 | 118.3 | 2357.5 | 5333.6 | 2070.7 |
| 130 | | [At5g14550](http://atted.jp/cgi-bin/coex_Ath.cgi?gene=At5g14550&sort=all) | |  | **159.4** | | 16418.4 | 164.2 | 988.7 | 13989.7 | 8743.5 |
| 131 | | [At4g04330](http://atted.jp/cgi-bin/coex_Ath.cgi?gene=At4g04330&sort=all) | |  | **159.8** | | 11281.9 | 155.2 | 89.4 | 6163.8 | 18066.3 |
| 132 | | [At2g23340](http://atted.jp/cgi-bin/coex_Ath.cgi?gene=At2g23340&sort=all) | | DEAR3 | **160.5** | | 6572.7 | 160.5 | 10974.0 | 11366.3 | 12669.1 |
| 133 | | [At1g33970](http://atted.jp/cgi-bin/coex_Ath.cgi?gene=At1g33970&sort=all) | | AIG | **164.2** | | 15074.9 | 61.5 | 402.9 | 3588.7 | 460.0 |
| 134 | | [At2g45660](http://atted.jp/cgi-bin/coex_Ath.cgi?gene=At2g45660&sort=all) | | AGL20 | **164.9** | | 10993.7 | 72.5 | 37.0 | 322.1 | 5391.9 |
| 135 | | [At3g53030](http://atted.jp/cgi-bin/coex_Ath.cgi?gene=At3g53030&sort=all) | | SRPK4 | **165.8** | | 6286.4 | 158.4 | 2350.3 | 1687.7 | 15183.9 |
| 136 | | [At5g03470](http://atted.jp/cgi-bin/coex_Ath.cgi?gene=At5g03470&sort=all) | | B' ALPHA | **168.6** | | 16730.5 | 139.4 | 151.7 | 5344.9 | 5100.0 |
| 137 | | [At3g15440](http://atted.jp/cgi-bin/coex_Ath.cgi?gene=At3g15440&sort=all) | |  | **168.8** | | 11796.5 | 135.9 | 8806.9 | 9039.5 | 5239.4 |
| 138 | | [At5g40550](http://atted.jp/cgi-bin/coex_Ath.cgi?gene=At5g40550&sort=all) | |  | **172.3** | | 2300.1 | 603.0 | 534.1 | 8765.9 | 21237.5 |
| 139 | | [At2g17840](http://atted.jp/cgi-bin/coex_Ath.cgi?gene=At2g17840&sort=all) | | ERD7 | **174.1** | | 20847.5 | 33.3 | 6144.1 | 197.9 | 17223.6 |
| 140 | | [At4g12750](http://atted.jp/cgi-bin/coex_Ath.cgi?gene=At4g12750&sort=all) | | transcription | **174.5** | | 2058.8 | 198.6 | 979.3 | 20885.1 | 13089.6 |
| 141 | | [At2g18350](http://atted.jp/cgi-bin/coex_Ath.cgi?gene=At2g18350&sort=all) | | HB24 | **176.7** | | 10948.9 | 153.7 | 1812.1 | 1966.8 | 3533.1 |
| 142 | | [248745_at](http://atted.jp/cgi-bin/coex_Ath.cgi?gene=248745_at&sort=all) | |  | **176.9** | | 19308.3 | 226.9 | 389.7 | 4599.0 | 11004.9 |
| 143 | | [At1g48330](http://atted.jp/cgi-bin/coex_Ath.cgi?gene=At1g48330&sort=all) | |  | **177.0** | | 5241.5 | 579.3 | 3.5 | 9373.6 | 1854.0 |
| 144 | | [At1g73760](http://atted.jp/cgi-bin/coex_Ath.cgi?gene=At1g73760&sort=all) | | zinc finger | **178.0** | | 6019.7 | 190.1 | 160.3 | 4964.1 | 370.9 |
| 145 | | [At5g11460](http://atted.jp/cgi-bin/coex_Ath.cgi?gene=At5g11460&sort=all) | | senescence-associated | **178.2** | | 1655.4 | 875.7 | 1302.5 | 13701.2 | 21335.5 |
| 146 | | [At1g67370](http://atted.jp/cgi-bin/coex_Ath.cgi?gene=At1g67370&sort=all) | | ASY1 | **180.3** | | 5694.5 | 330.1 | 4810.7 | 7517.5 | 9455.6 |
| 147 | | [At4g24800](http://atted.jp/cgi-bin/coex_Ath.cgi?gene=At4g24800&sort=all) | | MA3 | **182.6** | | 1794.8 | 734.8 | 205.3 | 3971.1 | 2181.5 |
| 148 | | [At4g09020](http://atted.jp/cgi-bin/coex_Ath.cgi?gene=At4g09020&sort=all) | | ISA3 | **182.8** | | 8841.5 | 306.2 | 355.0 | 5664.3 | 17199.6 |
| 149 | | 249616_s_at | |  | **183.3** | | 19806.0 | 184.8 | 5662.6 | 13173.6 | 13309.5 |
| 150 | | [At5g42420](http://atted.jp/cgi-bin/coex_Ath.cgi?gene=At5g42420&sort=all) | | transporter | **184.2** | | 7526.7 | 225.5 | 184.8 | 19619.0 | 6147.0 |
| 151 | | [At1g75190](http://atted.jp/cgi-bin/coex_Ath.cgi?gene=At1g75190&sort=all) | |  | **186.9** | | 5144.9 | 242.3 | 470.2 | 15627.3 | 2835.3 |
| 152 | | [264429_at](http://atted.jp/cgi-bin/coex_Ath.cgi?gene=264429_at&sort=all) | |  | **187.7** | | 3543.3 | 494.8 | 2483.7 | 686.4 | 15186.5 |
| 153 | | [At3g16740](http://atted.jp/cgi-bin/coex_Ath.cgi?gene=At3g16740&sort=all) | | F-box | **191.7** | | 13459.0 | 183.3 | 994.7 | 5733.9 | 3417.4 |
| 154 | | [At2g32390](http://atted.jp/cgi-bin/coex_Ath.cgi?gene=At2g32390&sort=all) | | GLR3.5 | **192.7** | | 14205.2 | 123.4 | 53.0 | 19014.0 | 14613.0 |
| 155 | | [At5g61380](http://atted.jp/cgi-bin/coex_Ath.cgi?gene=At5g61380&sort=all) | | TOC1 | **193.4** | | 15007.9 | 215.8 | 4569.2 | 6216.9 | 19349.9 |
| 156 | | [At1g75370](http://atted.jp/cgi-bin/coex_Ath.cgi?gene=At1g75370&sort=all) | | SEC14 cytosolic factor | **193.4** | | 16215.1 | 62.2 | 93.9 | 106.1 | 19646.1 |
| 157 | | [At5g67570](http://atted.jp/cgi-bin/coex_Ath.cgi?gene=At5g67570&sort=all) | | DG1 | **195.5** | | 5902.5 | 501.3 | 1020.1 | 19091.1 | 6786.5 |
| 158 | | [At3g05880](http://atted.jp/cgi-bin/coex_Ath.cgi?gene=At3g05880&sort=all) | | RCI2A | **196.2** | | 15216.0 | 107.8 | 391.8 | 226.2 | 7579.4 |
| 159 | | [At2g46550](http://atted.jp/cgi-bin/coex_Ath.cgi?gene=At2g46550&sort=all) | |  | **198.1** | | 4377.8 | 357.0 | 213.8 | 10004.9 | 11661.1 |
| 160 | | [At1g68490](http://atted.jp/cgi-bin/coex_Ath.cgi?gene=At1g68490&sort=all) | |  | **198.3** | | 7232.8 | 170.8 | 1566.1 | 3454.8 | 14744.8 |
| 161 | | [At4g11600](http://atted.jp/cgi-bin/coex_Ath.cgi?gene=At4g11600&sort=all) | | GPX6 | **202.1** | | 18459.6 | 63.5 | 467.3 | 2333.8 | 20834.7 |
| 162 | | [At5g11150](http://atted.jp/cgi-bin/coex_Ath.cgi?gene=At5g11150&sort=all) | | VAMP713 | **202.2** | | 13954.8 | 249.1 | 1376.2 | 9013.5 | 9950.6 |
| 163 | | [At3g07080](http://atted.jp/cgi-bin/coex_Ath.cgi?gene=At3g07080&sort=all) | | membrane | **202.6** | | 12472.6 | 176.1 | 66.6 | 9166.7 | 7072.8 |
| 164 | | [At5g26040](http://atted.jp/cgi-bin/coex_Ath.cgi?gene=At5g26040&sort=all) | | HDA2 | **203.9** | | 18186.5 | 162.9 | 75.1 | 6706.9 | 3736.4 |
| 165 | | [At1g75140](http://atted.jp/cgi-bin/coex_Ath.cgi?gene=At1g75140&sort=all) | |  | **204.8** | | 2815.0 | 785.4 | 585.6 | 18996.5 | 9526.5 |
| 166 | | [At3g57170](http://atted.jp/cgi-bin/coex_Ath.cgi?gene=At3g57170&sort=all) | | Gpi1 | **207.1** | | 2366.1 | 932.0 | 321.0 | 8218.9 | 13786.8 |
| 167 | | [At3g50700](http://atted.jp/cgi-bin/coex_Ath.cgi?gene=At3g50700&sort=all) | | IDD2 | **207.1** | | 8905.7 | 327.4 | 55.2 | 12835.6 | 6961.0 |
| 168 | | [At3g47160](http://atted.jp/cgi-bin/coex_Ath.cgi?gene=At3g47160&sort=all) | | protein binding | **208.5** | | 20052.9 | 199.9 | 244.1 | 3138.0 | 3328.1 |
| 169 | | [At1g20696](http://atted.jp/cgi-bin/coex_Ath.cgi?gene=At1g20696&sort=all) | | HMGB3 | **208.9** | | 4082.0 | 344.8 | 1523.0 | 5437.8 | 4459.2 |
| 170 | | [At2g28360](http://atted.jp/cgi-bin/coex_Ath.cgi?gene=At2g28360&sort=all) | |  | **212.3** | | 14474.6 | 141.8 | 464.4 | 10985.0 | 12442.9 |
| 171 | | [At4g33670](http://atted.jp/cgi-bin/coex_Ath.cgi?gene=At4g33670&sort=all) | | L-GalDH | **213.0** | | 3646.5 | 507.1 | 318.9 | 10783.7 | 13618.7 |
| 172 | | [At1g12730](http://atted.jp/cgi-bin/coex_Ath.cgi?gene=At1g12730&sort=all) | | cell division cycle | **216.6** | | 6540.5 | 835.5 | 63.1 | 9494.2 | 3291.4 |
| 173 | | [At5g42900](http://atted.jp/cgi-bin/coex_Ath.cgi?gene=At5g42900&sort=all) | | COR27 | **218.4** | | 10704.1 | 300.5 | 4928.4 | 1352.3 | 15119.5 |
| 174 | | [At4g28140](http://atted.jp/cgi-bin/coex_Ath.cgi?gene=At4g28140&sort=all) | | transcription | **220.0** | | 11664.1 | 150.5 | 714.6 | 14415.6 | 16654.1 |
| 175 | | [At1g14685](http://atted.jp/cgi-bin/coex_Ath.cgi?gene=At1g14685&sort=all) | | BPC2 | **220.6** | | 1347.0 | 381.7 | 5624.6 | 10111.8 | 5509.5 |
| 176 | | [At5g25990](http://atted.jp/cgi-bin/coex_Ath.cgi?gene=At5g25990&sort=all) | |  | **221.9** | | 9400.8 | 245.1 | 275.0 | 11264.2 | 2760.4 |
| 177 | | [At1g78440](http://atted.jp/cgi-bin/coex_Ath.cgi?gene=At1g78440&sort=all) | | GA2OX1 | **222.7** | | 5476.2 | 177.3 | 6703.6 | 4806.7 | 21720.1 |
| 178 | | [At2g36390](http://atted.jp/cgi-bin/coex_Ath.cgi?gene=At2g36390&sort=all) | | SBE2.1 | **223.1** | | 11484.4 | 299.9 | 681.6 | 3093.8 | 21267.1 |
| 179 | | [At3g51390](http://atted.jp/cgi-bin/coex_Ath.cgi?gene=At3g51390&sort=all) | | zinc finger | **224.1** | | 14610.6 | 155.4 | 321.2 | 17014.9 | 3364.9 |
| 180 | | [At4g33980](http://atted.jp/cgi-bin/coex_Ath.cgi?gene=At4g33980&sort=all) | |  | **225.7** | | 18794.3 | 219.0 | 4354.9 | 2601.3 | 11806.7 |
| 181 | | [At1g69830](http://atted.jp/cgi-bin/coex_Ath.cgi?gene=At1g69830&sort=all) | | AMY3 | **226.0** | | 7098.3 | 256.1 | 340.9 | 5196.0 | 11837.8 |
| 182 | | [At5g10780](http://atted.jp/cgi-bin/coex_Ath.cgi?gene=At5g10780&sort=all) | |  | **227.7** | | 9316.8 | 97.2 | 1900.6 | 19840.4 | 14675.0 |
| 183 | | [At3g01310](http://atted.jp/cgi-bin/coex_Ath.cgi?gene=At3g01310&sort=all) | | oxidoreductase | **227.9** | | 16753.8 | 283.2 | 60.2 | 14031.4 | 10302.4 |
| 184 | | 256595_x_at | |  | **229.0** | | 7340.7 | 358.8 | 4489.9 | 260.4 | 19214.8 |
| 185 | | [At4g31290](http://atted.jp/cgi-bin/coex_Ath.cgi?gene=At4g31290&sort=all) | | ChaC-like | **230.1** | | 4062.4 | 75.5 | 1091.6 | 820.9 | 12645.0 |
| 186 | | [At5g46860](http://atted.jp/cgi-bin/coex_Ath.cgi?gene=At5g46860&sort=all) | | VAM3 | **231.3** | | 15372.1 | 325.8 | 1796.7 | 18138.0 | 3684.8 |
| 187 | | 267335_s_at | |  | **232.2** | | 2899.9 | 220.4 | 2189.6 | 2276.3 | 6567.9 |
| 188 | | [At1g08040](http://atted.jp/cgi-bin/coex_Ath.cgi?gene=At1g08040&sort=all) | |  | **232.7** | | 6210.0 | 606.0 | 1507.7 | 206.0 | 4560.8 |
| 189 | | [At3g12980](http://atted.jp/cgi-bin/coex_Ath.cgi?gene=At3g12980&sort=all) | | HAC5 | **232.7** | | 15762.5 | 92.3 | 2290.9 | 19695.8 | 1309.7 |
| 190 | | [At3g14000](http://atted.jp/cgi-bin/coex_Ath.cgi?gene=At3g14000&sort=all) | | BRXL2 | **235.2** | | 257.9 | 87.8 | 290.0 | 1917.7 | 10154.7 |
| 191 | | [At1g21790](http://atted.jp/cgi-bin/coex_Ath.cgi?gene=At1g21790&sort=all) | |  | **239.0** | | 9774.2 | 349.4 | 4589.1 | 19.9 | 14165.7 |
| 192 | | [At5g55180](http://atted.jp/cgi-bin/coex_Ath.cgi?gene=At5g55180&sort=all) | | hydrolase | **240.2** | | 154.1 | 231.9 | 10832.0 | 104.4 | 15159.2 |
| 193 | | [At5g18525](http://atted.jp/cgi-bin/coex_Ath.cgi?gene=At5g18525&sort=all) | | kinase | **245.0** | | 20620.6 | 135.7 | 1655.7 | 5430.8 | 7646.6 |
| 194 | | [At4g25520](http://atted.jp/cgi-bin/coex_Ath.cgi?gene=At4g25520&sort=all) | | SLK1 | **246.4** | | 10428.6 | 26.1 | 13290.0 | 3973.0 | 6970.4 |
| 195 | | [At2g42520](http://atted.jp/cgi-bin/coex_Ath.cgi?gene=At2g42520&sort=all) | | helicase | **247.7** | | 9628.0 | 122.2 | 5867.7 | 19329.5 | 3020.0 |
| 196 | | [At1g76580](http://atted.jp/cgi-bin/coex_Ath.cgi?gene=At1g76580&sort=all) | | transcription | **248.6** | | 9824.0 | 273.4 | 8987.6 | 258.8 | 9001.3 |
| 197 | | [At1g68500](http://atted.jp/cgi-bin/coex_Ath.cgi?gene=At1g68500&sort=all) | |  | **249.3** | | 18741.1 | 230.4 | 4526.6 | 365.6 | 15896.8 |
| 198 | | [At4g33700](http://atted.jp/cgi-bin/coex_Ath.cgi?gene=At4g33700&sort=all) | | CBS | **254.5** | | 5218.8 | 686.0 | 2125.4 | 2000.6 | 11656.1 |
| 199 | | [At5g26230](http://atted.jp/cgi-bin/coex_Ath.cgi?gene=At5g26230&sort=all) | |  | **254.5** | | 4903.3 | 336.1 | 317.0 | 8108.9 | 4844.1 |
| 200 | | [At4g09560](http://atted.jp/cgi-bin/coex_Ath.cgi?gene=At4g09560&sort=all) | | binding | **258.1** | | 5775.0 | 342.0 | 1480.7 | 7207.4 | 16600.7 |
| 201 | | [At3g59820](http://atted.jp/cgi-bin/coex_Ath.cgi?gene=At3g59820&sort=all) | | calcium-binding mitochondrial | **259.0** | | 7799.4 | 174.4 | 2020.3 | 12786.4 | 19697.5 |
| 202 | | [At1g10090](http://atted.jp/cgi-bin/coex_Ath.cgi?gene=At1g10090&sort=all) | |  | **259.4** | | 21358.2 | 33.5 | 1812.1 | 446.2 | 10136.8 |
| 203 | | [At1g17460](http://atted.jp/cgi-bin/coex_Ath.cgi?gene=At1g17460&sort=all) | | TRFL3 | **261.6** | | 875.9 | 952.3 | 1132.6 | 20792.3 | 21458.0 |
| 204 | | [At2g42540](http://atted.jp/cgi-bin/coex_Ath.cgi?gene=At2g42540&sort=all) | | COR15A | **261.9** | | 14142.0 | 79.2 | 1530.2 | 24.5 | 7917.6 |
| 205 | | [At5g04940](http://atted.jp/cgi-bin/coex_Ath.cgi?gene=At5g04940&sort=all) | | SUVH1 | **267.8** | | 459.7 | 266.9 | 1627.3 | 19433.8 | 2845.0 |
| 206 | | 263823_s_at | |  | **269.6** | | 15127.3 | 397.3 | 2249.8 | 8078.5 | 11101.9 |
| 207 | | [At5g47060](http://atted.jp/cgi-bin/coex_Ath.cgi?gene=At5g47060&sort=all) | | senescence-associated | **269.6** | | 3694.3 | 162.2 | 5640.9 | 4270.9 | 8636.9 |
| 208 | | [At1g14680](http://atted.jp/cgi-bin/coex_Ath.cgi?gene=At1g14680&sort=all) | |  | **271.2** | | 2541.4 | 254.3 | 15756.1 | 18412.0 | 9580.1 |
| 209 | | [At5g62090](http://atted.jp/cgi-bin/coex_Ath.cgi?gene=At5g62090&sort=all) | | SLK2 | **271.4** | | 3220.3 | 618.0 | 2885.1 | 477.0 | 16086.2 |
| 210 | | [At4g27440](http://atted.jp/cgi-bin/coex_Ath.cgi?gene=At4g27440&sort=all) | | PORB | **274.5** | | 5499.3 | 154.6 | 68.8 | 14538.6 | 8718.0 |
| 211 | | [At4g15490](http://atted.jp/cgi-bin/coex_Ath.cgi?gene=At4g15490&sort=all) | | UGT84A3 | **276.4** | | 8886.6 | 158.4 | 1007.1 | 1119.5 | 10391.1 |
| 212 | | [At5g25210](http://atted.jp/cgi-bin/coex_Ath.cgi?gene=At5g25210&sort=all) | |  | **276.7** | | 18462.4 | 235.2 | 2069.5 | 12186.3 | 4460.3 |
| 213 | | [At5g51570](http://atted.jp/cgi-bin/coex_Ath.cgi?gene=At5g51570&sort=all) | | band 7 | **277.0** | | 15749.0 | 137.0 | 6174.8 | 17678.1 | 5738.1 |
| 214 | | [At5g64860](http://atted.jp/cgi-bin/coex_Ath.cgi?gene=At5g64860&sort=all) | | DPE1 | **277.7** | | 6777.2 | 457.8 | 159.9 | 14974.0 | 13044.2 |
| 215 | | [At5g27280](http://atted.jp/cgi-bin/coex_Ath.cgi?gene=At5g27280&sort=all) | | zinc finger | **280.9** | | 18838.3 | 137.0 | 278.9 | 1292.0 | 10783.2 |
| 216 | | [At5g02810](http://atted.jp/cgi-bin/coex_Ath.cgi?gene=At5g02810&sort=all) | | PRR7 | **281.2** | | 5316.2 | 959.1 | 115.5 | 10800.2 | 13732.6 |
| 217 | | [245432_at](http://atted.jp/cgi-bin/coex_Ath.cgi?gene=245432_at&sort=all) | |  | **284.9** | | 14239.7 | 203.2 | 34.4 | 17820.4 | 8764.0 |
| 218 | | [At4g02370](http://atted.jp/cgi-bin/coex_Ath.cgi?gene=At4g02370&sort=all) | |  | **285.4** | | 18406.4 | 154.9 | 1828.1 | 635.3 | 2405.5 |
| 219 | | 252475_s_at | |  | **286.4** | | 18764.8 | 259.0 | 2892.9 | 5041.8 | 12243.4 |
| 220 | | [At5g11580](http://atted.jp/cgi-bin/coex_Ath.cgi?gene=At5g11580&sort=all) | | RCC1 | **287.8** | | 6451.6 | 592.4 | 185.1 | 19849.1 | 14843.5 |
| 221 | | [At1g10760](http://atted.jp/cgi-bin/coex_Ath.cgi?gene=At1g10760&sort=all) | | SEX1 | **291.1** | | 10450.7 | 195.3 | 133.4 | 1255.7 | 20008.9 |
| 222 | | [At2g38465](http://atted.jp/cgi-bin/coex_Ath.cgi?gene=At2g38465&sort=all) | |  | **291.3** | | 17347.8 | 129.4 | 543.0 | 551.6 | 7694.1 |
| 223 | | [At3g05220](http://atted.jp/cgi-bin/coex_Ath.cgi?gene=At3g05220&sort=all) | | heavy-metal-associated | **291.7** | | 6495.0 | 273.6 | 6128.1 | 21271.6 | 10278.4 |
| 224 | | [At1g66410](http://atted.jp/cgi-bin/coex_Ath.cgi?gene=At1g66410&sort=all) | | CAM4 | **292.4** | | 6084.2 | 240.1 | 4368.2 | 7753.5 | 6820.9 |
| 225 | | [At3g46370](http://atted.jp/cgi-bin/coex_Ath.cgi?gene=At3g46370&sort=all) | | kinase | **292.6** | | 13873.9 | 320.9 | 177.6 | 6950.2 | 16141.4 |
| 226 | | [At1g72340](http://atted.jp/cgi-bin/coex_Ath.cgi?gene=At1g72340&sort=all) | | translation | **293.0** | | 7292.6 | 187.3 | 1766.3 | 21545.1 | 5808.6 |
| 227 | | [At3g46970](http://atted.jp/cgi-bin/coex_Ath.cgi?gene=At3g46970&sort=all) | | PHS2 | **293.1** | | 8329.6 | 257.6 | 142.3 | 12345.6 | 22163.5 |
| 228 | | [At3g50970](http://atted.jp/cgi-bin/coex_Ath.cgi?gene=At3g50970&sort=all) | | LTI30 | **294.5** | | 12758.8 | 105.5 | 4144.6 | 652.7 | 13628.4 |
| 229 | | [At2g19860](http://atted.jp/cgi-bin/coex_Ath.cgi?gene=At2g19860&sort=all) | | HXK2 | **294.5** | | 4381.2 | 475.1 | 2256.6 | 5101.9 | 13352.4 |
| 230 | | [At2g37520](http://atted.jp/cgi-bin/coex_Ath.cgi?gene=At2g37520&sort=all) | | PHD finger | **295.1** | | 21674.0 | 51.6 | 2727.2 | 8663.3 | 7707.6 |
| 231 | | [At3g05120](http://atted.jp/cgi-bin/coex_Ath.cgi?gene=At3g05120&sort=all) | | GID1A | **295.2** | | 7001.0 | 293.5 | 1933.2 | 14198.1 | 1144.9 |
| 232 | | [At4g39730](http://atted.jp/cgi-bin/coex_Ath.cgi?gene=At4g39730&sort=all) | | lipid-associated | **295.5** | | 5402.7 | 639.5 | 363.9 | 1888.3 | 15612.0 |
| 233 | | [At2g28720](http://atted.jp/cgi-bin/coex_Ath.cgi?gene=At2g28720&sort=all) | | H2B | **296.5** | | 10487.2 | 404.3 | 306.7 | 14808.2 | 6406.1 |
| 234 | | [At3g19960](http://atted.jp/cgi-bin/coex_Ath.cgi?gene=At3g19960&sort=all) | | ATM1 | **297.4** | | 8136.0 | 226.9 | 892.4 | 14302.9 | 4015.9 |
| 235 | | [At2g39900](http://atted.jp/cgi-bin/coex_Ath.cgi?gene=At2g39900&sort=all) | | LIM | **297.4** | | 6690.4 | 348.7 | 56.0 | 11967.0 | 814.7 |
| 236 | | [At1g76790](http://atted.jp/cgi-bin/coex_Ath.cgi?gene=At1g76790&sort=all) | | transferase | **298.4** | | 5815.9 | 145.7 | 566.1 | 13969.3 | 1133.8 |
| 237 | | [At2g03340](http://atted.jp/cgi-bin/coex_Ath.cgi?gene=At2g03340&sort=all) | | WRKY3 | **299.5** | | 4975.9 | 492.8 | 804.1 | 13888.0 | 9058.5 |
| 238 | | [At2g25930](http://atted.jp/cgi-bin/coex_Ath.cgi?gene=At2g25930&sort=all) | | ELF3 | **300.0** | | 10893.5 | 348.6 | 5813.2 | 19520.2 | 17702.9 |
| 239 | | [At2g22190](http://atted.jp/cgi-bin/coex_Ath.cgi?gene=At2g22190&sort=all) | | phosphatase | **301.2** | | 19298.9 | 259.6 | 436.9 | 209.8 | 14609.4 |
| 240 | | [At1g09350](http://atted.jp/cgi-bin/coex_Ath.cgi?gene=At1g09350&sort=all) | | GolS3 | **301.9** | | 18043.9 | 405.3 | 1552.2 | 1867.7 | 17150.7 |
| 241 | | [At5g54930](http://atted.jp/cgi-bin/coex_Ath.cgi?gene=At5g54930&sort=all) | | AT hook motif | **302.6** | | 5455.8 | 549.9 | 3697.9 | 13017.4 | 17158.6 |
| 242 | | [At5g59950](http://atted.jp/cgi-bin/coex_Ath.cgi?gene=At5g59950&sort=all) | | RNA and export factor-binding | **305.4** | | 3754.3 | 339.3 | 8541.8 | 4658.8 | 1265.8 |
| 243 | | [At1g20450](http://atted.jp/cgi-bin/coex_Ath.cgi?gene=At1g20450&sort=all) | | ERD10 | **308.6** | | 17060.4 | 195.8 | 2625.8 | 559.6 | 18636.4 |
| 244 | | [At1g17665](http://atted.jp/cgi-bin/coex_Ath.cgi?gene=At1g17665&sort=all) | |  | **308.9** | | 17112.2 | 373.0 | 4473.0 | 18381.0 | 19129.2 |
| 245 | | [At5g47240](http://atted.jp/cgi-bin/coex_Ath.cgi?gene=At5g47240&sort=all) | | atnudt8 | **309.0** | | 16058.6 | 170.3 | 916.1 | 771.0 | 6255.2 |
| 246 | | [At3g07274](http://atted.jp/cgi-bin/coex_Ath.cgi?gene=At3g07274&sort=all) | |  | **310.1** | | 20167.0 | 98.2 | 1637.9 | 5518.9 | 8634.8 |
| 247 | | [At4g13640](http://atted.jp/cgi-bin/coex_Ath.cgi?gene=At4g13640&sort=all) | | UNE16 | **312.0** | | 2648.8 | 169.9 | 764.2 | 15482.4 | 7752.5 |
| 248 | | [At3g18440](http://atted.jp/cgi-bin/coex_Ath.cgi?gene=At3g18440&sort=all) | | ALMT9 | **314.5** | | 8012.3 | 111.1 | 6388.5 | 11698.7 | 15104.8 |
| 249 | | 253879_s_at | |  | **315.5** | | 6716.8 | 291.4 | 7065.6 | 1636.5 | 17877.2 |
| 250 | | 259767_s_at | |  | **315.9** | | 1531.0 | 390.4 | 6783.5 | 21319.2 | 19007.9 |
| 251 | | [At1g21670](http://atted.jp/cgi-bin/coex_Ath.cgi?gene=At1g21670&sort=all) | |  | **317.0** | | 13952.8 | 100.0 | 1810.2 | 895.0 | 12980.4 |
| 252 | | [At2g34850](http://atted.jp/cgi-bin/coex_Ath.cgi?gene=At2g34850&sort=all) | | MEE25 | **320.4** | | 2854.5 | 1018.6 | 15240.8 | 170.5 | 4234.2 |
| 253 | | [At3g18080](http://atted.jp/cgi-bin/coex_Ath.cgi?gene=At3g18080&sort=all) | | BGLU44 | **321.6** | | 5514.8 | 448.3 | 12.7 | 4121.9 | 11332.6 |
| 254 | | [At5g61040](http://atted.jp/cgi-bin/coex_Ath.cgi?gene=At5g61040&sort=all) | |  | **325.1** | | 2736.4 | 349.6 | 697.4 | 19700.0 | 12269.6 |
| 255 | | [At1g20693](http://atted.jp/cgi-bin/coex_Ath.cgi?gene=At1g20693&sort=all) | | HMGB2 | **325.9** | | 9359.8 | 399.3 | 1423.2 | 6751.5 | 6746.4 |
| 256 | | [At5g26570](http://atted.jp/cgi-bin/coex_Ath.cgi?gene=At5g26570&sort=all) | | GWD3 | **327.6** | | 8902.8 | 373.2 | 774.4 | 9739.9 | 19803.0 |
| 257 | | [At4g18240](http://atted.jp/cgi-bin/coex_Ath.cgi?gene=At4g18240&sort=all) | | ATSS4 | **327.7** | | 15233.2 | 350.1 | 164.7 | 12049.1 | 14108.5 |
| 258 | | [At2g45560](http://atted.jp/cgi-bin/coex_Ath.cgi?gene=At2g45560&sort=all) | | CYP76C1 | **329.1** | | 18972.8 | 148.7 | 9.2 | 13801.8 | 19102.2 |
| 259 | | [At4g16750](http://atted.jp/cgi-bin/coex_Ath.cgi?gene=At4g16750&sort=all) | | transcription | **332.6** | | 10752.3 | 333.5 | 2170.5 | 244.9 | 15639.9 |
| 260 | | [At1g09320](http://atted.jp/cgi-bin/coex_Ath.cgi?gene=At1g09320&sort=all) | | agenet | **332.6** | | 5858.8 | 209.6 | 2954.7 | 19417.2 | 14857.3 |
| 261 | | [At2g02570](http://atted.jp/cgi-bin/coex_Ath.cgi?gene=At2g02570&sort=all) | | nucleic acid binding | **334.0** | | 9761.9 | 248.8 | 1501.2 | 10958.5 | 7594.3 |
| 262 | | [At1g13930](http://atted.jp/cgi-bin/coex_Ath.cgi?gene=At1g13930&sort=all) | |  | **334.2** | | 10847.5 | 221.7 | 45.1 | 490.2 | 14893.7 |
| 263 | | [At2g27950](http://atted.jp/cgi-bin/coex_Ath.cgi?gene=At2g27950&sort=all) | |  | **335.1** | | 12458.0 | 60.5 | 8544.5 | 728.4 | 11242.3 |
| 264 | | [At1g67300](http://atted.jp/cgi-bin/coex_Ath.cgi?gene=At1g67300&sort=all) | | transporter | **339.4** | | 8105.5 | 328.2 | 6735.0 | 836.0 | 9651.0 |
| 265 | | 253586_s_at | |  | **339.5** | | 12838.0 | 310.7 | 159.2 | 5111.8 | 12611.4 |
| 266 | | [At5g16820](http://atted.jp/cgi-bin/coex_Ath.cgi?gene=At5g16820&sort=all) | | HSF3 | **341.1** | | 16614.3 | 156.5 | 175.7 | 5730.2 | 18759.7 |
| 267 | | [At3g02250](http://atted.jp/cgi-bin/coex_Ath.cgi?gene=At3g02250&sort=all) | |  | **341.4** | | 3885.5 | 101.2 | 2528.6 | 7518.8 | 12653.1 |
| 268 | | [At3g58680](http://atted.jp/cgi-bin/coex_Ath.cgi?gene=At3g58680&sort=all) | | MBF1B | **345.6** | | 6493.2 | 382.3 | 3350.3 | 18079.8 | 16419.9 |
| 269 | | [At1g12120](http://atted.jp/cgi-bin/coex_Ath.cgi?gene=At1g12120&sort=all) | |  | **346.5** | | 5247.0 | 948.8 | 177.0 | 4491.5 | 9317.3 |
| 270 | | [At1g30360](http://atted.jp/cgi-bin/coex_Ath.cgi?gene=At1g30360&sort=all) | | ERD4 | **346.5** | | 10110.9 | 234.7 | 2280.7 | 2396.0 | 15738.6 |
| 271 | | [At5g63860](http://atted.jp/cgi-bin/coex_Ath.cgi?gene=At5g63860&sort=all) | | UVR8 | **346.9** | | 4815.7 | 777.6 | 734.5 | 13373.7 | 2743.1 |
| 272 | | [At4g33490](http://atted.jp/cgi-bin/coex_Ath.cgi?gene=At4g33490&sort=all) | | endopeptidase | **347.3** | | 19754.3 | 401.4 | 86.5 | 4433.4 | 11567.5 |
| 273 | | [At2g42670](http://atted.jp/cgi-bin/coex_Ath.cgi?gene=At2g42670&sort=all) | |  | **352.1** | | 8003.8 | 554.6 | 1558.8 | 13808.6 | 8429.2 |
| 274 | | [At2g12550](http://atted.jp/cgi-bin/coex_Ath.cgi?gene=At2g12550&sort=all) | | ubiquitin-associated (UBA)/TS-N | **353.6** | | 8559.9 | 81.9 | 9287.7 | 19241.2 | 4337.6 |
| 275 | | [At1g61240](http://atted.jp/cgi-bin/coex_Ath.cgi?gene=At1g61240&sort=all) | |  | **356.2** | | 1268.4 | 905.1 | 2088.4 | 4882.0 | 12042.4 |
| 276 | | [At5g61370](http://atted.jp/cgi-bin/coex_Ath.cgi?gene=At5g61370&sort=all) | | PPR | **357.4** | | 2756.2 | 285.7 | 12808.9 | 11817.4 | 2724.4 |
| 277 | | [At1g13740](http://atted.jp/cgi-bin/coex_Ath.cgi?gene=At1g13740&sort=all) | | AFP2 | **358.8** | | 2440.2 | 446.4 | 3727.0 | 450.9 | 21896.7 |
| 278 | | [At3g22180](http://atted.jp/cgi-bin/coex_Ath.cgi?gene=At3g22180&sort=all) | | zinc finger | **361.0** | | 1565.5 | 458.0 | 541.4 | 21225.1 | 5664.1 |
| 279 | | [At5g01200](http://atted.jp/cgi-bin/coex_Ath.cgi?gene=At5g01200&sort=all) | | myb | **361.6** | | 2713.7 | 353.3 | 2761.6 | 3312.8 | 15103.4 |
| 280 | | [At2g21590](http://atted.jp/cgi-bin/coex_Ath.cgi?gene=At2g21590&sort=all) | | APL4 | **365.4** | | 2801.9 | 693.0 | 3184.3 | 344.8 | 19356.5 |
| 281 | | [At5g51180](http://atted.jp/cgi-bin/coex_Ath.cgi?gene=At5g51180&sort=all) | |  | **365.5** | | 11190.7 | 204.3 | 2755.9 | 14023.9 | 3967.0 |
| 282 | | 256116_at | |  | **367.6** | | 4205.5 | 495.7 | 3598.7 | 2843.9 | 8807.9 |
| 283 | | [At1g77000](http://atted.jp/cgi-bin/coex_Ath.cgi?gene=At1g77000&sort=all) | | SKP2B | **370.1** | | 10553.5 | 127.2 | 3227.8 | 165.3 | 3455.7 |
| 284 | | [At4g24960](http://atted.jp/cgi-bin/coex_Ath.cgi?gene=At4g24960&sort=all) | | HVA22D | **370.8** | | 3007.4 | 907.5 | 5358.8 | 221.2 | 22267.8 |
| 285 | | [At1g77680](http://atted.jp/cgi-bin/coex_Ath.cgi?gene=At1g77680&sort=all) | | nuclease | **372.6** | | 14656.9 | 355.0 | 5317.0 | 1596.7 | 13653.9 |
| 286 | | [At4g01550](http://atted.jp/cgi-bin/coex_Ath.cgi?gene=At4g01550&sort=all) | | anac069 | **374.4** | | 20292.9 | 315.0 | 588.0 | 1329.9 | 14493.6 |
| 287 | | [At2g37580](http://atted.jp/cgi-bin/coex_Ath.cgi?gene=At2g37580&sort=all) | | zinc finger | **375.3** | | 13361.6 | 309.9 | 5575.8 | 1708.1 | 11320.8 |
| 288 | | [At1g63240](http://atted.jp/cgi-bin/coex_Ath.cgi?gene=At1g63240&sort=all) | |  | **377.0** | | 1606.7 | 1094.5 | 681.0 | 1939.1 | 14339.0 |
| 289 | | [At1g32090](http://atted.jp/cgi-bin/coex_Ath.cgi?gene=At1g32090&sort=all) | | ERD | **381.1** | | 3065.1 | 857.0 | 551.0 | 15348.6 | 13534.3 |
| 290 | | [At2g15890](http://atted.jp/cgi-bin/coex_Ath.cgi?gene=At2g15890&sort=all) | | MEE14 | **384.3** | | 14338.0 | 346.1 | 322.2 | 14980.9 | 2504.7 |
| 291 | | [At3g62700](http://atted.jp/cgi-bin/coex_Ath.cgi?gene=At3g62700&sort=all) | | MRP10 | **386.3** | | 7593.6 | 1069.1 | 60.3 | 470.1 | 2547.7 |
| 292 | | [At3g05520](http://atted.jp/cgi-bin/coex_Ath.cgi?gene=At3g05520&sort=all) | | F-actin capping | **389.4** | | 1457.0 | 2293.2 | 1534.5 | 2309.8 | 4102.4 |
| 293 | | [At2g40935](http://atted.jp/cgi-bin/coex_Ath.cgi?gene=At2g40935&sort=all) | |  | **389.6** | | 2746.3 | 784.3 | 1153.2 | 16657.4 | 4673.9 |
| 294 | | [At5g24610](http://atted.jp/cgi-bin/coex_Ath.cgi?gene=At5g24610&sort=all) | |  | **390.8** | | 2476.5 | 652.1 | 1850.1 | 2760.2 | 10605.0 |
| 295 | | [At3g60400](http://atted.jp/cgi-bin/coex_Ath.cgi?gene=At3g60400&sort=all) | | transcription | **393.9** | | 2932.2 | 685.6 | 4818.3 | 14632.1 | 12532.7 |
| 296 | | [At4g16880](http://atted.jp/cgi-bin/coex_Ath.cgi?gene=At4g16880&sort=all) | | disease resistance | **395.1** | | 9097.5 | 436.4 | 67.2 | 12103.0 | 17028.5 |
| 297 | | [At1g21730](http://atted.jp/cgi-bin/coex_Ath.cgi?gene=At1g21730&sort=all) | | MKRP1 | **396.3** | | 4160.8 | 564.3 | 52.7 | 6536.2 | 10343.0 |
| 298 | | [At3g11020](http://atted.jp/cgi-bin/coex_Ath.cgi?gene=At3g11020&sort=all) | | DREB2B | **396.8** | | 14442.1 | 517.0 | 210.8 | 5782.5 | 18996.7 |
| 299 | | [At3g05660](http://atted.jp/cgi-bin/coex_Ath.cgi?gene=At3g05660&sort=all) | | RLP33 | **399.1** | | 19276.7 | 243.9 | 4392.8 | 10695.1 | 14154.4 |
| 300 | | [At3g06620](http://atted.jp/cgi-bin/coex_Ath.cgi?gene=At3g06620&sort=all) | | kinase | **406.2** | | 16623.8 | 241.3 | 3561.6 | 12122.2 | 6250.4 |
|  | | | | | | | | | | | |
| **300 coexpressed gene with At1g75040** | | | | | | | | | | | |
|  | | **locus** | | **Short description** | **MR**  **(all)** | | **MR**  **(tissue)** | **MR**  **(abiotic)** | **MR**  **(biotic)** | **MR**  **(hormone)** | **MR**  **(light)** |
| 1 | | [At3g57260](http://atted.jp/cgi-bin/coex_Ath.cgi?gene=At3g57260&sort=all) | | BGL2 | **2.2** | | 7.8 | 16.9 | 34.6 | 2.0 | 955.3 |
| 2 | | [At5g10760](http://atted.jp/cgi-bin/coex_Ath.cgi?gene=At5g10760&sort=all) | | protease | **3.5** | | 10.2 | 11.2 | 110.0 | 3.7 | 4119.3 |
| 3 | | 254265_s_at | |  | **4.6** | | 4.1 | 15.8 | 61.0 | 8.9 | 3057.7 |
| 4 | | [At2g32680](http://atted.jp/cgi-bin/coex_Ath.cgi?gene=At2g32680&sort=all) | | RLP23 | **6.0** | | 20.7 | 9.9 | 55.3 | 13.4 | 2444.0 |
| 5 | | [At1g35710](http://atted.jp/cgi-bin/coex_Ath.cgi?gene=At1g35710&sort=all) | | kinase | **10.2** | | 15.7 | 60.4 | 76.5 | 23.3 | 2809.5 |
| 6 | | [At2g14610](http://atted.jp/cgi-bin/coex_Ath.cgi?gene=At2g14610&sort=all) | | PR1 | **10.5** | | 20.1 | 95.2 | 42.4 | 8.9 | 12559.1 |
| 7 | | [At2g18660](http://atted.jp/cgi-bin/coex_Ath.cgi?gene=At2g18660&sort=all) | | EXLB3 | **10.5** | | 37.4 | 40.8 | 8.5 | 5.3 | 4262.4 |
| 8 | | [At5g60900](http://atted.jp/cgi-bin/coex_Ath.cgi?gene=At5g60900&sort=all) | | RLK1 | **12.5** | | 18.0 | 92.0 | 208.6 | 159.2 | 2674.2 |
| 9 | | [At5g54610](http://atted.jp/cgi-bin/coex_Ath.cgi?gene=At5g54610&sort=all) | | ANK | **14.4** | | 8.7 | 287.6 | 464.6 | 20.6 | 15590.1 |
| 10 | | [At3g50480](http://atted.jp/cgi-bin/coex_Ath.cgi?gene=At3g50480&sort=all) | | HR4 | **16.3** | | 32.2 | 222.6 | 467.0 | 8.5 | 8910.6 |
| 11 | | [At3g25010](http://atted.jp/cgi-bin/coex_Ath.cgi?gene=At3g25010&sort=all) | | RLP41 | **16.6** | | 30.5 | 103.5 | 95.2 | 962.9 | 20901.4 |
| 12 | | [At4g23150](http://atted.jp/cgi-bin/coex_Ath.cgi?gene=At4g23150&sort=all) | | CRK7 | **17.0** | | 34.0 | 51.3 | 147.0 | 5.9 | 133.9 |
| 13 | | [At3g28540](http://atted.jp/cgi-bin/coex_Ath.cgi?gene=At3g28540&sort=all) | | ATPase | **18.4** | | 35.3 | 41.4 | 922.9 | 134.0 | 18941.3 |
| 14 | | [At4g02420](http://atted.jp/cgi-bin/coex_Ath.cgi?gene=At4g02420&sort=all) | | kinase | **18.4** | | 23.0 | 268.9 | 1380.8 | 6071.9 | 14676.5 |
| 15 | | 256431_s_at | |  | **21.3** | | 24.8 | 163.6 | 63.1 | 66.7 | 20995.3 |
| 16 | | [At2g24160](http://atted.jp/cgi-bin/coex_Ath.cgi?gene=At2g24160&sort=all) | |  | **21.5** | | 18.0 | 1401.0 | 476.9 | 64.5 | 1000.5 |
| 17 | | [At5g10380](http://atted.jp/cgi-bin/coex_Ath.cgi?gene=At5g10380&sort=all) | | RING1 | **22.6** | | 43.6 | 1573.6 | 845.6 | 137.5 | 3411.7 |
| 18 | | [At5g55450](http://atted.jp/cgi-bin/coex_Ath.cgi?gene=At5g55450&sort=all) | | LTP | **22.6** | | 913.9 | 7.8 | 14.5 | 6.3 | 527.8 |
| 19 | | [At2g14560](http://atted.jp/cgi-bin/coex_Ath.cgi?gene=At2g14560&sort=all) | | LURP1 | **23.9** | | 5.7 | 1469.7 | 587.8 | 8.8 | 11304.4 |
| 20 | | [At1g13470](http://atted.jp/cgi-bin/coex_Ath.cgi?gene=At1g13470&sort=all) | |  | **24.0** | | 68.4 | 127.8 | 249.7 | 15.5 | 18239.0 |
| 21 | | [At1g21250](http://atted.jp/cgi-bin/coex_Ath.cgi?gene=At1g21250&sort=all) | | WAK1 | **25.7** | | 51.6 | 672.9 | 324.1 | 15.1 | 7966.1 |
| 22 | | [At4g04500](http://atted.jp/cgi-bin/coex_Ath.cgi?gene=At4g04500&sort=all) | | CRK37 | **27.7** | | 46.3 | 518.0 | 480.4 | 2929.8 | 5243.2 |
| 23 | | [At2g04450](http://atted.jp/cgi-bin/coex_Ath.cgi?gene=At2g04450&sort=all) | | NUDT6 | **29.3** | | 47.7 | 573.9 | 175.6 | 148.5 | 20917.9 |
| 24 | | [At1g76960](http://atted.jp/cgi-bin/coex_Ath.cgi?gene=At1g76960&sort=all) | |  | **30.0** | | 34.1 | 125.3 | 597.9 | 9369.5 | 20591.6 |
| 25 | | [At3g57240](http://atted.jp/cgi-bin/coex_Ath.cgi?gene=At3g57240&sort=all) | | BG3 | **32.5** | | 737.4 | 67.2 | 15.0 | 4.2 | 2567.0 |
| 26 | | 257763_s_at | |  | **34.2** | | 19.0 | 615.5 | 187.6 | 39.2 | 8527.1 |
| 27 | | [At1g08450](http://atted.jp/cgi-bin/coex_Ath.cgi?gene=At1g08450&sort=all) | | CRT3 | **35.2** | | 287.0 | 635.3 | 21.6 | 44.2 | 5485.1 |
| 28 | | [At1g69730](http://atted.jp/cgi-bin/coex_Ath.cgi?gene=At1g69730&sort=all) | | kinase | **37.8** | | 76.7 | 447.6 | 1353.2 | 231.2 | 22073.7 |
| 29 | | [At3g05650](http://atted.jp/cgi-bin/coex_Ath.cgi?gene=At3g05650&sort=all) | | RLP32 | **38.1** | | 69.8 | 1262.1 | 1730.9 | 9395.6 | 639.1 |
| 30 | | [At4g11890](http://atted.jp/cgi-bin/coex_Ath.cgi?gene=At4g11890&sort=all) | | kinase | **38.6** | | 27.5 | 76.4 | 212.2 | 61.2 | 5758.5 |
| 31 | | [At3g45860](http://atted.jp/cgi-bin/coex_Ath.cgi?gene=At3g45860&sort=all) | | CRK4 | **39.8** | | 51.5 | 15627.1 | 1419.1 | 297.0 | 12583.8 |
| 32 | | [At1g35230](http://atted.jp/cgi-bin/coex_Ath.cgi?gene=At1g35230&sort=all) | | AGP5 | **40.1** | | 28.0 | 61.2 | 139.4 | 341.5 | 113.5 |
| 33 | | 262926_s_at | |  | **41.7** | | 21.7 | 7495.9 | 2404.9 | 1233.2 | 6616.8 |
| 34 | | [At2g32160](http://atted.jp/cgi-bin/coex_Ath.cgi?gene=At2g32160&sort=all) | |  | **43.4** | | 24.5 | 7123.4 | 5255.7 | 5794.1 | 12056.3 |
| 35 | | [At4g10500](http://atted.jp/cgi-bin/coex_Ath.cgi?gene=At4g10500&sort=all) | | oxygenase | **43.4** | | 209.4 | 589.6 | 13.4 | 1098.3 | 13843.7 |
| 36 | | [At1g09080](http://atted.jp/cgi-bin/coex_Ath.cgi?gene=At1g09080&sort=all) | | BIP3 | **44.2** | | 38.9 | 421.9 | 646.7 | 4695.4 | 3648.9 |
| 37 | | [At2g17040](http://atted.jp/cgi-bin/coex_Ath.cgi?gene=At2g17040&sort=all) | | anac036 | **45.0** | | 22.8 | 678.0 | 1023.3 | 756.9 | 3864.4 |
| 38 | | [At3g60420](http://atted.jp/cgi-bin/coex_Ath.cgi?gene=At3g60420&sort=all) | |  | **47.3** | | 8.1 | 804.2 | 843.5 | 13.3 | 1017.8 |
| 39 | | [At1g69720](http://atted.jp/cgi-bin/coex_Ath.cgi?gene=At1g69720&sort=all) | | ho3 | **47.4** | | 159.8 | 863.9 | 555.3 | 349.3 | 1697.1 |
| 40 | | [At4g13810](http://atted.jp/cgi-bin/coex_Ath.cgi?gene=At4g13810&sort=all) | | RLP47 | **49.2** | | 73.5 | 525.0 | 1322.3 | 12234.3 | 14293.2 |
| 41 | | [At4g23220](http://atted.jp/cgi-bin/coex_Ath.cgi?gene=At4g23220&sort=all) | | CRK14 | **50.0** | | 34.5 | 22.6 | 1517.6 | 395.6 | 21445.1 |
| 42 | | [At3g22231](http://atted.jp/cgi-bin/coex_Ath.cgi?gene=At3g22231&sort=all) | | PCC1 | **50.1** | | 88.1 | 7349.1 | 464.7 | 22.0 | 12734.8 |
| 43 | | [At4g14400](http://atted.jp/cgi-bin/coex_Ath.cgi?gene=At4g14400&sort=all) | | ACD6 | **50.6** | | 48.8 | 3918.1 | 1812.6 | 14.9 | 12770.9 |
| 44 | | [At2g29120](http://atted.jp/cgi-bin/coex_Ath.cgi?gene=At2g29120&sort=all) | | GLR2.7 | **51.5** | | 203.5 | 60.8 | 1253.2 | 78.0 | 15383.7 |
| 45 | | 252450_s_at | |  | **53.5** | | 87.3 | 292.5 | 375.6 | 5453.4 | 22416.9 |
| 46 | | [At5g64000](http://atted.jp/cgi-bin/coex_Ath.cgi?gene=At5g64000&sort=all) | | SAL2 | **53.8** | | 121.9 | 1813.7 | 33.9 | 31.8 | 15817.1 |
| 47 | | [At1g23090](http://atted.jp/cgi-bin/coex_Ath.cgi?gene=At1g23090&sort=all) | | AST91 | **54.8** | | 283.5 | 1444.1 | 3587.8 | 4886.7 | 12546.1 |
| 48 | | [At2g43570](http://atted.jp/cgi-bin/coex_Ath.cgi?gene=At2g43570&sort=all) | | CHI | **54.8** | | 121.1 | 26.1 | 122.2 | 1106.2 | 13514.1 |
| 49 | | [At5g52810](http://atted.jp/cgi-bin/coex_Ath.cgi?gene=At5g52810&sort=all) | | ornithine cyclodeaminase/mu-crystallin | **55.0** | | 123.5 | 805.1 | 182.2 | 81.6 | 3921.6 |
| 50 | | [At5g24530](http://atted.jp/cgi-bin/coex_Ath.cgi?gene=At5g24530&sort=all) | | DMR6 | **56.9** | | 682.0 | 17.5 | 4.2 | 46.6 | 15131.0 |
| 51 | | 254741_s_at | |  | **57.4** | | 117.2 | 80.2 | 1203.4 | 3322.7 | 18106.3 |
| 52 | | [At3g08870](http://atted.jp/cgi-bin/coex_Ath.cgi?gene=At3g08870&sort=all) | | kinase | **59.0** | | 166.2 | 48.7 | 1789.0 | 4096.4 | 8576.8 |
| 53 | | [At3g07520](http://atted.jp/cgi-bin/coex_Ath.cgi?gene=At3g07520&sort=all) | | GLR1.4 | **59.2** | | 288.0 | 1291.8 | 59.7 | 719.2 | 835.0 |
| 54 | | 261986_s_at | |  | **63.8** | | 115.0 | 2881.6 | 1166.0 | 3445.7 | 3245.9 |
| 55 | | [245771_at](http://atted.jp/cgi-bin/coex_Ath.cgi?gene=245771_at&sort=all) | |  | **65.5** | | 127.3 | 2460.8 | 9542.5 | 16739.8 | 18818.7 |
| 56 | | [At3g24900](http://atted.jp/cgi-bin/coex_Ath.cgi?gene=At3g24900&sort=all) | | RLP39 | **66.5** | | 260.1 | 248.6 | 105.2 | 6936.3 | 21102.1 |
| 57 | | [At4g04220](http://atted.jp/cgi-bin/coex_Ath.cgi?gene=At4g04220&sort=all) | | RLP46 | **66.9** | | 145.2 | 67.0 | 71.0 | 148.8 | 15958.0 |
| 58 | | [At3g45290](http://atted.jp/cgi-bin/coex_Ath.cgi?gene=At3g45290&sort=all) | | MLO3 | **70.2** | | 182.1 | 17.6 | 732.6 | 3231.2 | 18715.5 |
| 59 | | [At5g24210](http://atted.jp/cgi-bin/coex_Ath.cgi?gene=At5g24210&sort=all) | | lipase | **73.0** | | 114.5 | 6574.0 | 1633.5 | 37.1 | 17550.7 |
| 60 | | [At2g24850](http://atted.jp/cgi-bin/coex_Ath.cgi?gene=At2g24850&sort=all) | | TAT3 | **73.8** | | 34.6 | 561.0 | 844.1 | 156.7 | 2187.3 |
| 61 | | [At4g23610](http://atted.jp/cgi-bin/coex_Ath.cgi?gene=At4g23610&sort=all) | |  | **76.0** | | 56.4 | 172.2 | 679.6 | 382.0 | 10145.7 |
| 62 | | [At1g65500](http://atted.jp/cgi-bin/coex_Ath.cgi?gene=At1g65500&sort=all) | |  | **76.4** | | 349.8 | 138.2 | 318.1 | 18.5 | 18204.6 |
| 63 | | [At2g29350](http://atted.jp/cgi-bin/coex_Ath.cgi?gene=At2g29350&sort=all) | | SAG13 | **81.8** | | 143.2 | 66.8 | 1178.6 | 5086.9 | 7139.2 |
| 64 | | [At4g23310](http://atted.jp/cgi-bin/coex_Ath.cgi?gene=At4g23310&sort=all) | | CRK23 | **83.0** | | 399.4 | 170.7 | 55.5 | 7500.4 | 7852.7 |
| 65 | | [At5g22380](http://atted.jp/cgi-bin/coex_Ath.cgi?gene=At5g22380&sort=all) | | anac090 | **84.3** | | 75.3 | 1201.0 | 4741.4 | 5225.3 | 7190.9 |
| 66 | | [At2g37710](http://atted.jp/cgi-bin/coex_Ath.cgi?gene=At2g37710&sort=all) | | RLK | **87.2** | | 123.0 | 79.9 | 1775.6 | 73.8 | 4879.9 |
| 67 | | [At2g31880](http://atted.jp/cgi-bin/coex_Ath.cgi?gene=At2g31880&sort=all) | | SOBIR1 | **89.2** | | 256.9 | 173.9 | 264.2 | 43.0 | 11547.4 |
| 68 | | 263776_s_at | |  | **89.5** | | 151.0 | 554.1 | 1230.7 | 43.9 | 21713.5 |
| 69 | | [At3g25020](http://atted.jp/cgi-bin/coex_Ath.cgi?gene=At3g25020&sort=all) | | RLP42 | **89.5** | | 245.3 | 1210.5 | 361.3 | 15240.5 | 3270.1 |
| 70 | | [At1g21240](http://atted.jp/cgi-bin/coex_Ath.cgi?gene=At1g21240&sort=all) | | WAK3 | **90.4** | | 130.0 | 211.4 | 183.1 | 817.5 | 1405.7 |
| 71 | | [At3g04210](http://atted.jp/cgi-bin/coex_Ath.cgi?gene=At3g04210&sort=all) | | TIR-NBS | **90.9** | | 56.1 | 10357.0 | 1959.3 | 237.9 | 845.6 |
| 72 | | [At4g14365](http://atted.jp/cgi-bin/coex_Ath.cgi?gene=At4g14365&sort=all) | | zinc finger | **92.8** | | 96.9 | 234.9 | 957.7 | 110.1 | 13962.3 |
| 73 | | [At3g50770](http://atted.jp/cgi-bin/coex_Ath.cgi?gene=At3g50770&sort=all) | | calmodulin | **94.5** | | 132.9 | 266.1 | 3981.4 | 1493.3 | 3998.8 |
| 74 | | [At1g33960](http://atted.jp/cgi-bin/coex_Ath.cgi?gene=At1g33960&sort=all) | | AIG1 | **94.8** | | 59.0 | 524.9 | 1423.9 | 97.0 | 10415.7 |
| 75 | | [248083_at](http://atted.jp/cgi-bin/coex_Ath.cgi?gene=248083_at&sort=all) | |  | **95.8** | | 358.7 | 326.3 | 17.7 | 5185.9 | 827.7 |
| 76 | | [At4g08470](http://atted.jp/cgi-bin/coex_Ath.cgi?gene=At4g08470&sort=all) | | MAPKKK10 | **99.0** | | 119.7 | 1065.2 | 731.8 | 157.0 | 21710.9 |
| 77 | | [At1g66880](http://atted.jp/cgi-bin/coex_Ath.cgi?gene=At1g66880&sort=all) | | kinase | **99.3** | | 215.8 | 82.2 | 311.5 | 196.6 | 8091.5 |
| 78 | | [At1g21270](http://atted.jp/cgi-bin/coex_Ath.cgi?gene=At1g21270&sort=all) | | WAK2 | **100.9** | | 59.6 | 11650.7 | 1437.8 | 6297.6 | 15517.9 |
| 79 | | 245760_s_at | |  | **107.1** | | 97.5 | 2726.4 | 4014.4 | 1226.1 | 12053.1 |
| 80 | | [At1g66960](http://atted.jp/cgi-bin/coex_Ath.cgi?gene=At1g66960&sort=all) | | cyclase | **108.2** | | 541.2 | 6069.4 | 149.1 | 5045.5 | 3547.8 |
| 81 | | [At1g03400](http://atted.jp/cgi-bin/coex_Ath.cgi?gene=At1g03400&sort=all) | | dioxygenase | **110.1** | | 114.6 | 10925.8 | 2885.1 | 110.8 | 10998.4 |
| 82 | | [At4g03450](http://atted.jp/cgi-bin/coex_Ath.cgi?gene=At4g03450&sort=all) | | ankyrin repeat | **113.8** | | 97.3 | 229.4 | 500.8 | 1593.7 | 21749.2 |
| 83 | | [At2g23200](http://atted.jp/cgi-bin/coex_Ath.cgi?gene=At2g23200&sort=all) | | kinase | **114.6** | | 97.9 | 3294.4 | 2145.0 | 375.6 | 18647.5 |
| 84 | | [At5g12890](http://atted.jp/cgi-bin/coex_Ath.cgi?gene=At5g12890&sort=all) | | transferase | **114.9** | | 251.2 | 4437.9 | 1999.5 | 2682.5 | 11166.6 |
| 85 | | [At1g55910](http://atted.jp/cgi-bin/coex_Ath.cgi?gene=At1g55910&sort=all) | | ZIP11 | **114.9** | | 238.2 | 1199.4 | 1072.7 | 155.1 | 18586.2 |
| 86 | | [At1g56150](http://atted.jp/cgi-bin/coex_Ath.cgi?gene=At1g56150&sort=all) | | auxin-responsive | **116.2** | | 140.1 | 2609.6 | 2945.6 | 8185.4 | 311.3 |
| 87 | | [254244_at](http://atted.jp/cgi-bin/coex_Ath.cgi?gene=254244_at&sort=all) | |  | **119.5** | | 247.4 | 46.9 | 1230.9 | 1108.7 | 15190.2 |
| 88 | | [At2g26440](http://atted.jp/cgi-bin/coex_Ath.cgi?gene=At2g26440&sort=all) | | pectinesterase | **121.5** | | 638.6 | 1287.7 | 1907.5 | 315.6 | 12897.7 |
| 89 | | [At1g17610](http://atted.jp/cgi-bin/coex_Ath.cgi?gene=At1g17610&sort=all) | | disease resistance | **122.0** | | 199.0 | 6496.2 | 2058.1 | 533.4 | 18163.7 |
| 90 | | [At5g52760](http://atted.jp/cgi-bin/coex_Ath.cgi?gene=At5g52760&sort=all) | | heavy-metal-associated | **122.0** | | 59.8 | 790.2 | 368.1 | 60.8 | 1513.7 |
| 91 | | [At1g23840](http://atted.jp/cgi-bin/coex_Ath.cgi?gene=At1g23840&sort=all) | |  | **122.5** | | 255.6 | 199.3 | 32.5 | 2265.4 | 21464.4 |
| 92 | | [At5g36930](http://atted.jp/cgi-bin/coex_Ath.cgi?gene=At5g36930&sort=all) | | TIR-NBS-LRR | **123.0** | | 157.8 | 3847.9 | 1233.6 | 614.8 | 1237.4 |
| 93 | | [At4g23260](http://atted.jp/cgi-bin/coex_Ath.cgi?gene=At4g23260&sort=all) | | CRK18 | **126.8** | | 185.3 | 4189.5 | 2031.6 | 841.2 | 1480.8 |
| 94 | | [At5g52740](http://atted.jp/cgi-bin/coex_Ath.cgi?gene=At5g52740&sort=all) | | heavy-metal-associated | **126.8** | | 338.0 | 483.4 | 141.6 | 5708.3 | 1432.8 |
| 95 | | [At1g59620](http://atted.jp/cgi-bin/coex_Ath.cgi?gene=At1g59620&sort=all) | | CW9 | **129.8** | | 60.7 | 2389.5 | 823.5 | 754.8 | 16869.5 |
| 96 | | [At3g47250](http://atted.jp/cgi-bin/coex_Ath.cgi?gene=At3g47250&sort=all) | |  | **130.0** | | 246.0 | 6570.2 | 2185.3 | 9576.3 | 21556.0 |
| 97 | | [At1g71390](http://atted.jp/cgi-bin/coex_Ath.cgi?gene=At1g71390&sort=all) | | RLP11 | **130.3** | | 180.9 | 5871.2 | 845.2 | 21560.0 | 9054.0 |
| 98 | | [At1g57630](http://atted.jp/cgi-bin/coex_Ath.cgi?gene=At1g57630&sort=all) | | TIR | **130.6** | | 38.5 | 459.5 | 1786.5 | 659.0 | 18592.9 |
| 99 | | [At3g47480](http://atted.jp/cgi-bin/coex_Ath.cgi?gene=At3g47480&sort=all) | | calcium-binding EF hand | **130.8** | | 59.2 | 1722.9 | 661.1 | 132.2 | 20012.9 |
| 100 | | [At4g24120](http://atted.jp/cgi-bin/coex_Ath.cgi?gene=At4g24120&sort=all) | | YSL1 | **131.3** | | 250.7 | 2215.4 | 13362.8 | 3520.9 | 13624.8 |
| 101 | | [At3g26230](http://atted.jp/cgi-bin/coex_Ath.cgi?gene=At3g26230&sort=all) | | CYP71B24 | **133.8** | | 119.2 | 2594.9 | 3789.2 | 1777.6 | 13624.0 |
| 102 | | [At3g48650](http://atted.jp/cgi-bin/coex_Ath.cgi?gene=At3g48650&sort=all) | |  | **135.1** | | 49.4 | 532.8 | 2638.0 | 475.3 | 3468.1 |
| 103 | | [At4g11900](http://atted.jp/cgi-bin/coex_Ath.cgi?gene=At4g11900&sort=all) | | kinase | **136.8** | | 45.0 | 9016.1 | 4657.2 | 217.5 | 21680.8 |
| 104 | | [At4g22260](http://atted.jp/cgi-bin/coex_Ath.cgi?gene=At4g22260&sort=all) | | IM | **137.0** | | 196.9 | 2748.4 | 3054.4 | 3591.1 | 22195.2 |
| 105 | | [At4g28490](http://atted.jp/cgi-bin/coex_Ath.cgi?gene=At4g28490&sort=all) | | HAE | **137.2** | | 354.5 | 324.1 | 886.7 | 5428.8 | 11400.2 |
| 106 | | [At2g41090](http://atted.jp/cgi-bin/coex_Ath.cgi?gene=At2g41090&sort=all) | | CaBP-22 | **138.6** | | 167.7 | 18848.2 | 1680.3 | 36.0 | 16638.4 |
| 107 | | [At1g16260](http://atted.jp/cgi-bin/coex_Ath.cgi?gene=At1g16260&sort=all) | | kinase | **140.9** | | 172.4 | 13396.5 | 2049.3 | 1622.0 | 15925.6 |
| 108 | | [At3g55500](http://atted.jp/cgi-bin/coex_Ath.cgi?gene=At3g55500&sort=all) | | EXPA16 | **141.0** | | 622.6 | 355.3 | 10498.9 | 13662.1 | 6572.2 |
| 109 | | [At3g56710](http://atted.jp/cgi-bin/coex_Ath.cgi?gene=At3g56710&sort=all) | | SIB1 | **141.4** | | 38.5 | 771.4 | 503.3 | 155.4 | 451.7 |
| 110 | | 263405_s_at | |  | **143.5** | | 151.3 | 4220.9 | 5782.7 | 13153.0 | 18447.1 |
| 111 | | 255344_s_at | |  | **144.0** | | 224.2 | 10150.8 | 38.8 | 2665.1 | 7488.7 |
| 112 | | [At5g39030](http://atted.jp/cgi-bin/coex_Ath.cgi?gene=At5g39030&sort=all) | | kinase | **144.6** | | 349.0 | 1222.4 | 3254.6 | 10308.3 | 17205.7 |
| 113 | | [At3g23010](http://atted.jp/cgi-bin/coex_Ath.cgi?gene=At3g23010&sort=all) | | RLP36 | **146.4** | | 107.0 | 2036.3 | 2567.9 | 5893.1 | 8176.2 |
| 114 | | [At5g03350](http://atted.jp/cgi-bin/coex_Ath.cgi?gene=At5g03350&sort=all) | | legume lectin | **148.6** | | 1656.8 | 6729.8 | 273.4 | 14.5 | 15550.0 |
| 115 | | [At5g18470](http://atted.jp/cgi-bin/coex_Ath.cgi?gene=At5g18470&sort=all) | | curculin-like lectin | **149.0** | | 624.5 | 86.8 | 67.7 | 106.2 | 13641.2 |
| 116 | | [At4g23320](http://atted.jp/cgi-bin/coex_Ath.cgi?gene=At4g23320&sort=all) | | CRK24 | **150.1** | | 191.2 | 168.7 | 2156.3 | 2742.4 | 1574.5 |
| 117 | | [At2g36970](http://atted.jp/cgi-bin/coex_Ath.cgi?gene=At2g36970&sort=all) | | transferase | **153.8** | | 627.6 | 55.1 | 2783.7 | 4490.9 | 4830.2 |
| 118 | | [At5g59670](http://atted.jp/cgi-bin/coex_Ath.cgi?gene=At5g59670&sort=all) | | kinase | **155.9** | | 155.0 | 2939.7 | 1139.9 | 1187.5 | 2737.5 |
| 119 | | [At2g44240](http://atted.jp/cgi-bin/coex_Ath.cgi?gene=At2g44240&sort=all) | |  | **156.4** | | 808.8 | 6668.1 | 452.2 | 10740.5 | 16757.4 |
| 120 | | [At5g61010](http://atted.jp/cgi-bin/coex_Ath.cgi?gene=At5g61010&sort=all) | | EXO70E2 | **157.0** | | 439.4 | 744.9 | 1385.9 | 31.5 | 12277.5 |
| 121 | | [At4g26070](http://atted.jp/cgi-bin/coex_Ath.cgi?gene=At4g26070&sort=all) | | MEK1 | **159.0** | | 137.2 | 3271.0 | 681.6 | 233.0 | 20322.6 |
| 122 | | [At5g40060](http://atted.jp/cgi-bin/coex_Ath.cgi?gene=At5g40060&sort=all) | | ATP binding | **159.8** | | 56.1 | 1556.0 | 5003.8 | 3445.1 | 2535.1 |
| 123 | | [At3g28930](http://atted.jp/cgi-bin/coex_Ath.cgi?gene=At3g28930&sort=all) | | AIG2 | **162.4** | | 512.0 | 222.9 | 734.0 | 701.5 | 13853.7 |
| 124 | | [At5g44580](http://atted.jp/cgi-bin/coex_Ath.cgi?gene=At5g44580&sort=all) | |  | **164.8** | | 501.0 | 1291.4 | 4944.7 | 57.2 | 22350.9 |
| 125 | | [At1g15790](http://atted.jp/cgi-bin/coex_Ath.cgi?gene=At1g15790&sort=all) | |  | **166.5** | | 607.4 | 927.9 | 214.8 | 4416.6 | 1148.0 |
| 126 | | [At3g01080](http://atted.jp/cgi-bin/coex_Ath.cgi?gene=At3g01080&sort=all) | | WRKY58 | **169.7** | | 299.7 | 5618.6 | 1991.6 | 12242.9 | 313.6 |
| 127 | | 266746_s_at | |  | **170.8** | | 1358.1 | 23.0 | 124.9 | 111.2 | 7396.0 |
| 128 | | [At5g44820](http://atted.jp/cgi-bin/coex_Ath.cgi?gene=At5g44820&sort=all) | |  | **173.0** | | 578.5 | 266.8 | 566.9 | 295.7 | 19534.1 |
| 129 | | 262832_s_at | |  | **178.0** | | 1369.9 | 28.6 | 93.0 | 695.8 | 6313.5 |
| 130 | | [260919_at](http://atted.jp/cgi-bin/coex_Ath.cgi?gene=260919_at&sort=all) | |  | **179.3** | | 244.7 | 1036.8 | 887.5 | 336.0 | 1922.2 |
| 131 | | [At4g37560](http://atted.jp/cgi-bin/coex_Ath.cgi?gene=At4g37560&sort=all) | | hydrolase | **182.2** | | 122.5 | 2851.9 | 4678.0 | 7064.0 | 13851.5 |
| 132 | | [At1g66970](http://atted.jp/cgi-bin/coex_Ath.cgi?gene=At1g66970&sort=all) | | SVL2 | **182.3** | | 241.2 | 17008.1 | 2719.4 | 260.7 | 10045.0 |
| 133 | | 264107_s_at | |  | **183.2** | | 331.6 | 894.1 | 861.0 | 329.1 | 17486.7 |
| 134 | | 249480_s_at | |  | **185.3** | | 265.3 | 9723.1 | 2323.2 | 92.7 | 1091.9 |
| 135 | | [At2g35940](http://atted.jp/cgi-bin/coex_Ath.cgi?gene=At2g35940&sort=all) | | BLH1 | **186.0** | | 518.3 | 319.7 | 3765.4 | 5030.1 | 13604.0 |
| 136 | | [At3g26210](http://atted.jp/cgi-bin/coex_Ath.cgi?gene=At3g26210&sort=all) | | CYP71B23 | **188.2** | | 1183.7 | 31.6 | 493.4 | 88.4 | 20457.3 |
| 137 | | [At3g49340](http://atted.jp/cgi-bin/coex_Ath.cgi?gene=At3g49340&sort=all) | | cysteinease | **188.8** | | 90.8 | 1403.1 | 3758.0 | 7106.3 | 18848.8 |
| 138 | | 262374_s_at | |  | **190.1** | | 162.6 | 8897.3 | 2963.0 | 15.0 | 6962.9 |
| 139 | | [At3g48640](http://atted.jp/cgi-bin/coex_Ath.cgi?gene=At3g48640&sort=all) | |  | **195.3** | | 98.9 | 728.9 | 2328.9 | 640.0 | 5991.6 |
| 140 | | [At2g15042](http://atted.jp/cgi-bin/coex_Ath.cgi?gene=At2g15042&sort=all) | | protein binding | **195.7** | | 146.2 | 3155.8 | 3047.3 | 7787.8 | 499.3 |
| 141 | | [At1g30900](http://atted.jp/cgi-bin/coex_Ath.cgi?gene=At1g30900&sort=all) | | vacuolar sorting receptor | **196.1** | | 1377.3 | 207.7 | 253.2 | 85.2 | 19432.1 |
| 142 | | [At3g03560](http://atted.jp/cgi-bin/coex_Ath.cgi?gene=At3g03560&sort=all) | |  | **196.3** | | 575.1 | 621.6 | 562.4 | 419.2 | 22139.4 |
| 143 | | [At1g71040](http://atted.jp/cgi-bin/coex_Ath.cgi?gene=At1g71040&sort=all) | | LPR2 | **196.9** | | 734.3 | 351.3 | 1208.3 | 212.1 | 13678.7 |
| 144 | | [At4g33050](http://atted.jp/cgi-bin/coex_Ath.cgi?gene=At4g33050&sort=all) | | EDA39 | **199.6** | | 327.8 | 492.7 | 2073.1 | 324.3 | 14250.0 |
| 145 | | [At1g77810](http://atted.jp/cgi-bin/coex_Ath.cgi?gene=At1g77810&sort=all) | | transferase | **199.7** | | 407.4 | 1197.7 | 238.8 | 12411.6 | 5102.9 |
| 146 | | [At1g67865](http://atted.jp/cgi-bin/coex_Ath.cgi?gene=At1g67865&sort=all) | |  | **205.6** | | 250.4 | 15926.3 | 16626.9 | 13682.8 | 2254.5 |
| 147 | | 266615_s_at | |  | **207.0** | | 268.4 | 1708.0 | 2106.7 | 982.2 | 10663.1 |
| 148 | | [At1g64400](http://atted.jp/cgi-bin/coex_Ath.cgi?gene=At1g64400&sort=all) | | synthetase | **207.3** | | 712.3 | 1173.1 | 3848.8 | 255.3 | 7825.8 |
| 149 | | [At2g24860](http://atted.jp/cgi-bin/coex_Ath.cgi?gene=At2g24860&sort=all) | | chaperonin | **209.8** | | 473.4 | 1265.5 | 5182.0 | 8912.1 | 13960.5 |
| 150 | | [At4g21380](http://atted.jp/cgi-bin/coex_Ath.cgi?gene=At4g21380&sort=all) | | ARK3 | **210.2** | | 184.0 | 2169.1 | 1878.5 | 370.5 | 2282.9 |
| 151 | | [At4g23240](http://atted.jp/cgi-bin/coex_Ath.cgi?gene=At4g23240&sort=all) | | CRK16 | **210.7** | | 115.9 | 14521.6 | 7376.7 | 11123.2 | 3934.0 |
| 152 | | [At3g56400](http://atted.jp/cgi-bin/coex_Ath.cgi?gene=At3g56400&sort=all) | | WRKY70 | **211.5** | | 255.9 | 6824.4 | 651.4 | 63.2 | 14954.8 |
| 153 | | 262119_s_at | |  | **213.3** | | 1998.3 | 24.5 | 174.3 | 35.6 | 7875.6 |
| 154 | | [At4g23130](http://atted.jp/cgi-bin/coex_Ath.cgi?gene=At4g23130&sort=all) | | CRK5 | **217.1** | | 176.2 | 724.4 | 3635.8 | 367.5 | 4166.0 |
| 155 | | [At5g46500](http://atted.jp/cgi-bin/coex_Ath.cgi?gene=At5g46500&sort=all) | |  | **218.5** | | 186.0 | 3596.1 | 1148.5 | 14767.5 | 1979.4 |
| 156 | | [At4g38560](http://atted.jp/cgi-bin/coex_Ath.cgi?gene=At4g38560&sort=all) | |  | **219.0** | | 180.6 | 425.9 | 1710.4 | 503.8 | 11564.5 |
| 157 | | [At2g15080](http://atted.jp/cgi-bin/coex_Ath.cgi?gene=At2g15080&sort=all) | | RLP19 | **219.7** | | 788.4 | 1797.1 | 1619.8 | 2245.2 | 396.7 |
| 158 | | [At2g40750](http://atted.jp/cgi-bin/coex_Ath.cgi?gene=At2g40750&sort=all) | | WRKY54 | **221.7** | | 171.3 | 6209.9 | 718.9 | 168.0 | 8508.4 |
| 159 | | [At1g73805](http://atted.jp/cgi-bin/coex_Ath.cgi?gene=At1g73805&sort=all) | | calmodulin binding | **222.6** | | 146.4 | 1737.8 | 290.7 | 98.0 | 1516.9 |
| 160 | | [At2g40600](http://atted.jp/cgi-bin/coex_Ath.cgi?gene=At2g40600&sort=all) | | appr-1-p processing enzyme | **222.9** | | 543.3 | 10537.3 | 411.9 | 24.4 | 10885.4 |
| 161 | | [At3g50950](http://atted.jp/cgi-bin/coex_Ath.cgi?gene=At3g50950&sort=all) | | CC-NBS-LRR | **224.5** | | 370.0 | 1077.2 | 4208.9 | 71.1 | 16720.9 |
| 162 | | [At4g16860](http://atted.jp/cgi-bin/coex_Ath.cgi?gene=At4g16860&sort=all) | | RPP4 | **226.4** | | 122.9 | 12579.4 | 3892.8 | 21697.5 | 3168.9 |
| 163 | | [At3g14620](http://atted.jp/cgi-bin/coex_Ath.cgi?gene=At3g14620&sort=all) | | CYP72A8 | **227.6** | | 538.9 | 2202.4 | 579.7 | 1326.9 | 13928.5 |
| 164 | | [At3g62410](http://atted.jp/cgi-bin/coex_Ath.cgi?gene=At3g62410&sort=all) | | CP12-2 | **232.2** | | 264.3 | 10378.2 | 9141.0 | 1379.3 | 15416.0 |
| 165 | | [At1g63880](http://atted.jp/cgi-bin/coex_Ath.cgi?gene=At1g63880&sort=all) | | TIR-NBS-LRR | **233.7** | | 160.0 | 3724.6 | 6784.1 | 8436.9 | 8831.5 |
| 166 | | [At5g11060](http://atted.jp/cgi-bin/coex_Ath.cgi?gene=At5g11060&sort=all) | | KNAT4 | **235.7** | | 204.0 | 5974.8 | 5416.9 | 13131.2 | 17257.5 |
| 167 | | [At1g74710](http://atted.jp/cgi-bin/coex_Ath.cgi?gene=At1g74710&sort=all) | | SID2 | **236.6** | | 117.1 | 2515.0 | 1131.1 | 190.4 | 17548.4 |
| 168 | | [At3g28890](http://atted.jp/cgi-bin/coex_Ath.cgi?gene=At3g28890&sort=all) | | RLP43 | **238.7** | | 178.6 | 2515.2 | 2604.2 | 14160.5 | 20593.8 |
| 169 | | [At5g13320](http://atted.jp/cgi-bin/coex_Ath.cgi?gene=At5g13320&sort=all) | | PBS3 | **239.7** | | 88.8 | 3424.4 | 1862.0 | 68.9 | 7374.6 |
| 170 | | [At1g34750](http://atted.jp/cgi-bin/coex_Ath.cgi?gene=At1g34750&sort=all) | | PP2C | **240.2** | | 720.5 | 662.4 | 1629.9 | 82.0 | 10833.7 |
| 171 | | [At1g27060](http://atted.jp/cgi-bin/coex_Ath.cgi?gene=At1g27060&sort=all) | | RCC1 | **240.8** | | 139.2 | 14287.4 | 1741.0 | 7875.0 | 1426.5 |
| 172 | | 257634_s_at | |  | **240.8** | | 161.6 | 11737.1 | 3216.0 | 20817.4 | 18369.1 |
| 173 | | [At5g52750](http://atted.jp/cgi-bin/coex_Ath.cgi?gene=At5g52750&sort=all) | | heavy-metal-associated | **244.9** | | 297.5 | 384.5 | 866.2 | 177.2 | 3906.9 |
| 174 | | [At1g29240](http://atted.jp/cgi-bin/coex_Ath.cgi?gene=At1g29240&sort=all) | |  | **247.8** | | 252.8 | 1476.1 | 1245.5 | 15294.2 | 17602.0 |
| 175 | | [At3g15840](http://atted.jp/cgi-bin/coex_Ath.cgi?gene=At3g15840&sort=all) | | PIFI | **248.8** | | 238.3 | 15693.5 | 6444.0 | 17798.5 | 13124.1 |
| 176 | | [At1g11330](http://atted.jp/cgi-bin/coex_Ath.cgi?gene=At1g11330&sort=all) | | kinase | **249.9** | | 622.5 | 868.0 | 377.7 | 152.3 | 20413.5 |
| 177 | | [At1g29720](http://atted.jp/cgi-bin/coex_Ath.cgi?gene=At1g29720&sort=all) | | kinase | **250.2** | | 182.7 | 2415.1 | 11316.1 | 8432.9 | 2531.5 |
| 178 | | [At1g10920](http://atted.jp/cgi-bin/coex_Ath.cgi?gene=At1g10920&sort=all) | | LOV1 | **251.2** | | 100.4 | 8825.4 | 2259.7 | 18661.4 | 19398.8 |
| 179 | | [At2g41180](http://atted.jp/cgi-bin/coex_Ath.cgi?gene=At2g41180&sort=all) | | sigA-binding | **251.7** | | 1293.9 | 353.1 | 1558.3 | 227.3 | 6792.4 |
| 180 | | [At3g26830](http://atted.jp/cgi-bin/coex_Ath.cgi?gene=At3g26830&sort=all) | | PAD3 | **254.6** | | 417.3 | 144.6 | 490.4 | 573.8 | 12143.0 |
| 181 | | [At5g04930](http://atted.jp/cgi-bin/coex_Ath.cgi?gene=At5g04930&sort=all) | | ALA1 | **255.5** | | 1028.7 | 150.7 | 339.1 | 365.5 | 3790.0 |
| 182 | | [At4g29810](http://atted.jp/cgi-bin/coex_Ath.cgi?gene=At4g29810&sort=all) | | MKK2 | **255.8** | | 1605.6 | 1540.3 | 94.6 | 1345.0 | 19586.3 |
| 183 | | [At1g78410](http://atted.jp/cgi-bin/coex_Ath.cgi?gene=At1g78410&sort=all) | | VQ motif | **259.4** | | 158.3 | 514.7 | 643.8 | 946.6 | 7123.2 |
| 184 | | [At1g31580](http://atted.jp/cgi-bin/coex_Ath.cgi?gene=At1g31580&sort=all) | | ECS1 | **261.6** | | 529.6 | 9710.2 | 718.1 | 13.0 | 20103.3 |
| 185 | | [At5g01540](http://atted.jp/cgi-bin/coex_Ath.cgi?gene=At5g01540&sort=all) | | LECRKA4.1 | **261.9** | | 114.2 | 433.3 | 3296.7 | 586.5 | 16643.1 |
| 186 | | [At4g00955](http://atted.jp/cgi-bin/coex_Ath.cgi?gene=At4g00955&sort=all) | |  | **263.5** | | 470.6 | 1677.5 | 1820.7 | 734.3 | 2751.0 |
| 187 | | [At2g25440](http://atted.jp/cgi-bin/coex_Ath.cgi?gene=At2g25440&sort=all) | | RLP20 | **264.0** | | 486.7 | 1163.8 | 2757.6 | 17688.4 | 8060.5 |
| 188 | | [At5g40780](http://atted.jp/cgi-bin/coex_Ath.cgi?gene=At5g40780&sort=all) | | LHT1 | **265.5** | | 700.8 | 165.5 | 1841.1 | 1096.6 | 9440.2 |
| 189 | | [At5g13720](http://atted.jp/cgi-bin/coex_Ath.cgi?gene=At5g13720&sort=all) | |  | **267.1** | | 199.3 | 8523.3 | 14345.0 | 12718.0 | 6797.9 |
| 190 | | [At3g26600](http://atted.jp/cgi-bin/coex_Ath.cgi?gene=At3g26600&sort=all) | | ARO4 | **268.4** | | 402.3 | 1904.0 | 851.5 | 2130.4 | 17768.4 |
| 191 | | [At3g48090](http://atted.jp/cgi-bin/coex_Ath.cgi?gene=At3g48090&sort=all) | | EDS1 | **270.4** | | 807.0 | 1190.5 | 692.4 | 98.9 | 3508.8 |
| 192 | | [At4g17570](http://atted.jp/cgi-bin/coex_Ath.cgi?gene=At4g17570&sort=all) | | GATA26 | **278.2** | | 238.0 | 2791.2 | 2879.3 | 11091.6 | 20215.2 |
| 193 | | [At4g34120](http://atted.jp/cgi-bin/coex_Ath.cgi?gene=At4g34120&sort=all) | | LEJ1 | **280.2** | | 604.3 | 3391.2 | 2798.9 | 11615.6 | 19622.2 |
| 194 | | [At4g16990](http://atted.jp/cgi-bin/coex_Ath.cgi?gene=At4g16990&sort=all) | | RLM3 | **283.5** | | 261.1 | 18439.8 | 13971.9 | 2159.2 | 21164.4 |
| 195 | | [At1g67860](http://atted.jp/cgi-bin/coex_Ath.cgi?gene=At1g67860&sort=all) | |  | **285.8** | | 273.8 | 13460.5 | 21564.0 | 22377.8 | 639.8 |
| 196 | | [264628_at](http://atted.jp/cgi-bin/coex_Ath.cgi?gene=264628_at&sort=all) | |  | **286.1** | | 1150.4 | 1053.3 | 269.7 | 9052.9 | 20264.7 |
| 197 | | [At5g44870](http://atted.jp/cgi-bin/coex_Ath.cgi?gene=At5g44870&sort=all) | | TIR-NBS-LRR | **286.4** | | 214.1 | 6726.4 | 5564.6 | 4651.5 | 18300.4 |
| 198 | | [At3g17700](http://atted.jp/cgi-bin/coex_Ath.cgi?gene=At3g17700&sort=all) | | CNBT1 | **288.1** | | 189.3 | 1383.3 | 1864.1 | 267.5 | 4890.0 |
| 199 | | [At3g48080](http://atted.jp/cgi-bin/coex_Ath.cgi?gene=At3g48080&sort=all) | | lipase | **288.2** | | 703.6 | 1275.5 | 500.7 | 744.0 | 782.7 |
| 200 | | [At4g22980](http://atted.jp/cgi-bin/coex_Ath.cgi?gene=At4g22980&sort=all) | |  | **288.4** | | 184.5 | 758.5 | 2015.3 | 4567.2 | 12379.5 |
| 201 | | [At1g02475](http://atted.jp/cgi-bin/coex_Ath.cgi?gene=At1g02475&sort=all) | |  | **291.3** | | 193.9 | 10747.0 | 7521.7 | 17729.5 | 20707.7 |
| 202 | | [At3g55130](http://atted.jp/cgi-bin/coex_Ath.cgi?gene=At3g55130&sort=all) | | WBC19 | **291.9** | | 1182.4 | 460.8 | 357.5 | 6657.3 | 19266.9 |
| 203 | | [At1g10340](http://atted.jp/cgi-bin/coex_Ath.cgi?gene=At1g10340&sort=all) | | ankyrin repeat | **292.9** | | 452.1 | 722.6 | 3080.7 | 138.8 | 19383.4 |
| 204 | | [At4g23810](http://atted.jp/cgi-bin/coex_Ath.cgi?gene=At4g23810&sort=all) | | WRKY53 | **293.6** | | 272.6 | 674.9 | 1630.4 | 335.7 | 7586.9 |
| 205 | | [At4g26090](http://atted.jp/cgi-bin/coex_Ath.cgi?gene=At4g26090&sort=all) | | RPS2 | **293.7** | | 287.2 | 1891.2 | 1314.5 | 951.5 | 3829.2 |
| 206 | | [At4g04490](http://atted.jp/cgi-bin/coex_Ath.cgi?gene=At4g04490&sort=all) | | CRK36 | **295.5** | | 114.3 | 1545.7 | 1855.2 | 4140.3 | 7097.8 |
| 207 | | [At2g39210](http://atted.jp/cgi-bin/coex_Ath.cgi?gene=At2g39210&sort=all) | | nodulin | **297.8** | | 1671.8 | 75.8 | 626.1 | 48.7 | 11055.8 |
| 208 | | [At1g01560](http://atted.jp/cgi-bin/coex_Ath.cgi?gene=At1g01560&sort=all) | | MPK11 | **300.1** | | 112.0 | 549.9 | 3399.9 | 886.0 | 6914.6 |
| 209 | | [At4g08850](http://atted.jp/cgi-bin/coex_Ath.cgi?gene=At4g08850&sort=all) | | kinase | **300.2** | | 964.8 | 867.8 | 2065.4 | 91.8 | 10126.8 |
| 210 | | [At1g05010](http://atted.jp/cgi-bin/coex_Ath.cgi?gene=At1g05010&sort=all) | | EFE | **300.3** | | 816.3 | 4579.8 | 2440.2 | 6559.4 | 7937.0 |
| 211 | | [At3g60540](http://atted.jp/cgi-bin/coex_Ath.cgi?gene=At3g60540&sort=all) | | sec61beta | **300.4** | | 2460.5 | 489.2 | 289.3 | 345.5 | 22624.5 |
| 212 | | [At5g25440](http://atted.jp/cgi-bin/coex_Ath.cgi?gene=At5g25440&sort=all) | | kinase | **300.9** | | 399.5 | 1682.3 | 3942.8 | 176.3 | 7640.7 |
| 213 | | [At3g44350](http://atted.jp/cgi-bin/coex_Ath.cgi?gene=At3g44350&sort=all) | | anac061 | **305.1** | | 158.6 | 3653.0 | 3771.4 | 2203.1 | 1453.5 |
| 214 | | [At5g56260](http://atted.jp/cgi-bin/coex_Ath.cgi?gene=At5g56260&sort=all) | | transferase | **305.2** | | 707.2 | 9308.5 | 809.5 | 1620.5 | 20947.4 |
| 215 | | [At3g26200](http://atted.jp/cgi-bin/coex_Ath.cgi?gene=At3g26200&sort=all) | | CYP71B22 | **305.6** | | 1650.0 | 2335.8 | 2224.5 | 4617.7 | 1300.8 |
| 216 | | [At4g25940](http://atted.jp/cgi-bin/coex_Ath.cgi?gene=At4g25940&sort=all) | | ENTH | **308.2** | | 574.8 | 7034.4 | 3562.3 | 319.1 | 3700.6 |
| 217 | | [At5g60950](http://atted.jp/cgi-bin/coex_Ath.cgi?gene=At5g60950&sort=all) | | COBL5 | **310.4** | | 3347.6 | 75.7 | 7.2 | 17.9 | 3723.6 |
| 218 | | [At1g68600](http://atted.jp/cgi-bin/coex_Ath.cgi?gene=At1g68600&sort=all) | |  | **311.9** | | 97.0 | 8213.4 | 12784.8 | 15549.6 | 20398.6 |
| 219 | | [At2g17120](http://atted.jp/cgi-bin/coex_Ath.cgi?gene=At2g17120&sort=all) | | LYM2 | **312.4** | | 1605.3 | 310.5 | 379.5 | 110.1 | 5006.9 |
| 220 | | [At1g74440](http://atted.jp/cgi-bin/coex_Ath.cgi?gene=At1g74440&sort=all) | |  | **312.7** | | 481.1 | 1732.3 | 1405.5 | 44.5 | 22371.9 |
| 221 | | [At1g17600](http://atted.jp/cgi-bin/coex_Ath.cgi?gene=At1g17600&sort=all) | | TIR-NBS-LRR | **314.5** | | 342.2 | 3479.7 | 3107.3 | 429.3 | 1077.6 |
| 222 | | [At4g39830](http://atted.jp/cgi-bin/coex_Ath.cgi?gene=At4g39830&sort=all) | | oxidase | **314.8** | | 93.8 | 1263.3 | 1450.0 | 2676.3 | 14476.8 |
| 223 | | [At1g52590](http://atted.jp/cgi-bin/coex_Ath.cgi?gene=At1g52590&sort=all) | |  | **317.1** | | 330.7 | 4636.9 | 3788.4 | 4920.6 | 22524.3 |
| 224 | | [At5g53370](http://atted.jp/cgi-bin/coex_Ath.cgi?gene=At5g53370&sort=all) | | PMEPCRF | **317.3** | | 952.5 | 2404.9 | 2029.5 | 2326.9 | 19223.9 |
| 225 | | [At5g25050](http://atted.jp/cgi-bin/coex_Ath.cgi?gene=At5g25050&sort=all) | | transporter | **321.7** | | 422.0 | 4021.2 | 3582.3 | 2253.5 | 10569.1 |
| 226 | | 265109_s_at | |  | **322.8** | | 221.8 | 11440.5 | 5641.4 | 16824.5 | 21442.1 |
| 227 | | [At1g51805](http://atted.jp/cgi-bin/coex_Ath.cgi?gene=At1g51805&sort=all) | | kinase | **322.8** | | 199.4 | 7877.4 | 20094.5 | 3875.2 | 17800.6 |
| 228 | | [At1g22890](http://atted.jp/cgi-bin/coex_Ath.cgi?gene=At1g22890&sort=all) | |  | **326.2** | | 567.4 | 3264.9 | 2117.2 | 1824.3 | 14152.4 |
| 229 | | [At3g18370](http://atted.jp/cgi-bin/coex_Ath.cgi?gene=At3g18370&sort=all) | | SYTF | **326.9** | | 791.5 | 1110.2 | 935.5 | 189.9 | 20343.8 |
| 230 | | [At4g13510](http://atted.jp/cgi-bin/coex_Ath.cgi?gene=At4g13510&sort=all) | | AMT1;1 | **328.4** | | 602.5 | 4855.2 | 1293.4 | 34.5 | 7409.4 |
| 231 | | [At2g33080](http://atted.jp/cgi-bin/coex_Ath.cgi?gene=At2g33080&sort=all) | | RLP28 | **330.2** | | 1995.4 | 430.7 | 2277.7 | 3947.1 | 1950.6 |
| 232 | | [At5g13030](http://atted.jp/cgi-bin/coex_Ath.cgi?gene=At5g13030&sort=all) | |  | **330.2** | | 534.3 | 2747.3 | 265.6 | 6214.8 | 14809.2 |
| 233 | | [At5g50160](http://atted.jp/cgi-bin/coex_Ath.cgi?gene=At5g50160&sort=all) | | FRO8 | **330.5** | | 107.2 | 18270.9 | 5797.4 | 10083.5 | 10237.4 |
| 234 | | [At2g02450](http://atted.jp/cgi-bin/coex_Ath.cgi?gene=At2g02450&sort=all) | | ANAC035 | **333.9** | | 162.8 | 13274.5 | 20612.3 | 1941.2 | 8967.8 |
| 235 | | [At2g29110](http://atted.jp/cgi-bin/coex_Ath.cgi?gene=At2g29110&sort=all) | | GLR2.8 | **334.9** | | 513.6 | 5301.0 | 1394.9 | 10572.5 | 209.1 |
| 236 | | [At5g22300](http://atted.jp/cgi-bin/coex_Ath.cgi?gene=At5g22300&sort=all) | | NIT4 | **335.4** | | 636.8 | 1084.4 | 4647.1 | 6658.7 | 7070.5 |
| 237 | | [At4g14220](http://atted.jp/cgi-bin/coex_Ath.cgi?gene=At4g14220&sort=all) | | RHF1A | **337.3** | | 607.8 | 69.2 | 462.2 | 648.0 | 21251.8 |
| 238 | | [At5g58570](http://atted.jp/cgi-bin/coex_Ath.cgi?gene=At5g58570&sort=all) | |  | **338.3** | | 494.0 | 2494.1 | 6189.7 | 19247.7 | 12440.0 |
| 239 | | [At5g53870](http://atted.jp/cgi-bin/coex_Ath.cgi?gene=At5g53870&sort=all) | | plastocyanin-like | **341.6** | | 1117.5 | 579.0 | 1285.4 | 8226.4 | 17031.6 |
| 240 | | [At5g58120](http://atted.jp/cgi-bin/coex_Ath.cgi?gene=At5g58120&sort=all) | | TIR-NBS-LRR | **344.3** | | 346.4 | 2422.5 | 3576.1 | 749.6 | 13140.1 |
| 241 | | [At4g14610](http://atted.jp/cgi-bin/coex_Ath.cgi?gene=At4g14610&sort=all) | |  | **350.3** | | 1102.7 | 421.2 | 1109.8 | 321.6 | 19951.5 |
| 242 | | [At1g78200](http://atted.jp/cgi-bin/coex_Ath.cgi?gene=At1g78200&sort=all) | | PP2C | **351.0** | | 191.2 | 5155.3 | 4222.5 | 15411.8 | 22548.3 |
| 243 | | [At5g39670](http://atted.jp/cgi-bin/coex_Ath.cgi?gene=At5g39670&sort=all) | | calcium-binding EF hand | **352.4** | | 376.2 | 1427.5 | 680.2 | 412.8 | 19692.1 |
| 244 | | [At1g72900](http://atted.jp/cgi-bin/coex_Ath.cgi?gene=At1g72900&sort=all) | | TIR-NBS | **353.5** | | 604.1 | 1531.1 | 1454.1 | 885.9 | 20180.0 |
| 245 | | [At3g62820](http://atted.jp/cgi-bin/coex_Ath.cgi?gene=At3g62820&sort=all) | | inhibitor | **353.9** | | 529.8 | 2019.2 | 15828.7 | 11734.8 | 13675.9 |
| 246 | | 263111_s_at | |  | **353.9** | | 319.7 | 15577.9 | 5102.4 | 657.6 | 21576.7 |
| 247 | | [At5g19240](http://atted.jp/cgi-bin/coex_Ath.cgi?gene=At5g19240&sort=all) | |  | **354.5** | | 956.6 | 882.4 | 1404.1 | 71.4 | 13996.3 |
| 248 | | [At3g57460](http://atted.jp/cgi-bin/coex_Ath.cgi?gene=At3g57460&sort=all) | | catalytic | **356.0** | | 62.5 | 904.8 | 4227.5 | 9645.4 | 11523.0 |
| 249 | | [At1g65490](http://atted.jp/cgi-bin/coex_Ath.cgi?gene=At1g65490&sort=all) | |  | **356.4** | | 243.1 | 9456.9 | 8805.6 | 2891.4 | 2871.4 |
| 250 | | [At2g43850](http://atted.jp/cgi-bin/coex_Ath.cgi?gene=At2g43850&sort=all) | | APK1 | **359.8** | | 707.7 | 7981.7 | 844.5 | 1035.8 | 10186.0 |
| 251 | | [At1g23130](http://atted.jp/cgi-bin/coex_Ath.cgi?gene=At1g23130&sort=all) | | Bet v I allergen | **361.2** | | 608.8 | 11105.8 | 17693.0 | 22194.7 | 418.6 |
| 252 | | [At4g23730](http://atted.jp/cgi-bin/coex_Ath.cgi?gene=At4g23730&sort=all) | | epimerase | **365.2** | | 297.1 | 4488.9 | 11750.0 | 6740.1 | 18119.9 |
| 253 | | [At4g11840](http://atted.jp/cgi-bin/coex_Ath.cgi?gene=At4g11840&sort=all) | | PLDGAMMA3 | **365.5** | | 339.9 | 1482.5 | 423.2 | 19470.2 | 22317.4 |
| 254 | | [At5g38210](http://atted.jp/cgi-bin/coex_Ath.cgi?gene=At5g38210&sort=all) | | kinase | **365.8** | | 989.0 | 283.0 | 1854.6 | 882.6 | 2103.6 |
| 255 | | [At5g49570](http://atted.jp/cgi-bin/coex_Ath.cgi?gene=At5g49570&sort=all) | | PNG1 | **367.9** | | 952.5 | 776.1 | 481.4 | 3245.1 | 18388.5 |
| 256 | | [At3g28480](http://atted.jp/cgi-bin/coex_Ath.cgi?gene=At3g28480&sort=all) | | oxygenase | **368.2** | | 1518.3 | 2280.6 | 202.5 | 330.8 | 19567.9 |
| 257 | | [At2g46600](http://atted.jp/cgi-bin/coex_Ath.cgi?gene=At2g46600&sort=all) | | calcium-binding | **368.4** | | 733.8 | 1551.0 | 6122.1 | 1114.2 | 9173.1 |
| 258 | | [At4g01700](http://atted.jp/cgi-bin/coex_Ath.cgi?gene=At4g01700&sort=all) | | chitinase | **373.3** | | 1684.1 | 176.3 | 935.3 | 77.1 | 7672.4 |
| 259 | | [At5g61520](http://atted.jp/cgi-bin/coex_Ath.cgi?gene=At5g61520&sort=all) | | transporter | **374.1** | | 840.4 | 1223.8 | 2625.1 | 6127.0 | 19725.1 |
| 260 | | [At2g24390](http://atted.jp/cgi-bin/coex_Ath.cgi?gene=At2g24390&sort=all) | |  | **374.5** | | 66.5 | 14425.9 | 2747.8 | 502.3 | 10359.3 |
| 261 | | [At5g48380](http://atted.jp/cgi-bin/coex_Ath.cgi?gene=At5g48380&sort=all) | | BIR1 | **375.5** | | 1246.5 | 564.0 | 912.6 | 275.2 | 16033.3 |
| 262 | | [At5g27830](http://atted.jp/cgi-bin/coex_Ath.cgi?gene=At5g27830&sort=all) | |  | **377.1** | | 621.2 | 3097.7 | 247.0 | 5954.9 | 20658.0 |
| 263 | | [At3g13950](http://atted.jp/cgi-bin/coex_Ath.cgi?gene=At3g13950&sort=all) | |  | **379.0** | | 1275.7 | 114.3 | 1020.2 | 5100.8 | 16156.4 |
| 264 | | 249552_s_at | |  | **379.3** | | 1815.5 | 46.0 | 1963.8 | 5114.6 | 686.3 |
| 265 | | [At2g33530](http://atted.jp/cgi-bin/coex_Ath.cgi?gene=At2g33530&sort=all) | | scpl46 | **380.2** | | 561.3 | 3132.8 | 2538.1 | 4351.9 | 16649.6 |
| 266 | | 248851_s_at | |  | **380.8** | | 129.4 | 10847.2 | 8370.6 | 1040.0 | 6812.0 |
| 267 | | [At4g17090](http://atted.jp/cgi-bin/coex_Ath.cgi?gene=At4g17090&sort=all) | | CT-BMY | **380.9** | | 421.4 | 3907.9 | 8419.4 | 505.9 | 11451.2 |
| 268 | | [At5g44420](http://atted.jp/cgi-bin/coex_Ath.cgi?gene=At5g44420&sort=all) | | PDF1.2 | **383.7** | | 1408.6 | 130.1 | 781.7 | 17053.4 | 1089.3 |
| 269 | | [At4g39210](http://atted.jp/cgi-bin/coex_Ath.cgi?gene=At4g39210&sort=all) | | APL3 | **383.9** | | 509.4 | 1175.6 | 7004.2 | 691.7 | 22403.0 |
| 270 | | [At1g09130](http://atted.jp/cgi-bin/coex_Ath.cgi?gene=At1g09130&sort=all) | | protease | **384.0** | | 538.2 | 3197.9 | 16569.5 | 4856.1 | 18818.6 |
| 271 | | [At4g11000](http://atted.jp/cgi-bin/coex_Ath.cgi?gene=At4g11000&sort=all) | | ankyrin repeat | **384.6** | | 526.4 | 1099.7 | 2220.3 | 734.5 | 5748.9 |
| 272 | | [At2g03530](http://atted.jp/cgi-bin/coex_Ath.cgi?gene=At2g03530&sort=all) | | UPS2 | **389.2** | | 522.4 | 6811.7 | 2191.4 | 3623.5 | 22730.5 |
| 273 | | [At3g18830](http://atted.jp/cgi-bin/coex_Ath.cgi?gene=At3g18830&sort=all) | | PLT5 | **393.0** | | 1294.7 | 36.1 | 3222.2 | 926.5 | 11254.0 |
| 274 | | [At1g66980](http://atted.jp/cgi-bin/coex_Ath.cgi?gene=At1g66980&sort=all) | | phosphodiesterase | **394.5** | | 297.2 | 13244.5 | 4588.2 | 12522.0 | 10601.6 |
| 275 | | [At1g61380](http://atted.jp/cgi-bin/coex_Ath.cgi?gene=At1g61380&sort=all) | | SD1-29 | **395.8** | | 445.7 | 882.2 | 7355.4 | 306.9 | 20015.7 |
| 276 | | [At2g30770](http://atted.jp/cgi-bin/coex_Ath.cgi?gene=At2g30770&sort=all) | | CYP71A13 | **396.6** | | 174.2 | 105.9 | 1322.5 | 3670.2 | 542.4 |
| 277 | | [At4g23180](http://atted.jp/cgi-bin/coex_Ath.cgi?gene=At4g23180&sort=all) | | CRK10 | **396.8** | | 483.1 | 2014.8 | 4345.8 | 607.0 | 14808.8 |
| 278 | | [At2g37540](http://atted.jp/cgi-bin/coex_Ath.cgi?gene=At2g37540&sort=all) | | SDR | **399.0** | | 374.1 | 15902.3 | 17717.5 | 615.4 | 6430.9 |
| 279 | | [At1g49050](http://atted.jp/cgi-bin/coex_Ath.cgi?gene=At1g49050&sort=all) | | protease | **399.2** | | 1104.0 | 551.1 | 725.1 | 142.8 | 9732.4 |
| 280 | | [At3g51660](http://atted.jp/cgi-bin/coex_Ath.cgi?gene=At3g51660&sort=all) | | MIF | **400.0** | | 574.2 | 1873.4 | 3564.0 | 8207.1 | 18415.1 |
| 281 | | [At1g28600](http://atted.jp/cgi-bin/coex_Ath.cgi?gene=At1g28600&sort=all) | | lipase | **400.6** | | 467.6 | 7078.0 | 18006.5 | 2740.3 | 11988.4 |
| 282 | | [At2g45630](http://atted.jp/cgi-bin/coex_Ath.cgi?gene=At2g45630&sort=all) | | oxidoreductase | **404.4** | | 456.5 | 1425.2 | 10901.6 | 14169.5 | 20370.8 |
| 283 | | [At4g23600](http://atted.jp/cgi-bin/coex_Ath.cgi?gene=At4g23600&sort=all) | | CORI3 | **405.6** | | 763.0 | 2586.5 | 6548.8 | 16151.6 | 126.5 |
| 284 | | [At1g78620](http://atted.jp/cgi-bin/coex_Ath.cgi?gene=At1g78620&sort=all) | | integral membrane | **405.8** | | 564.6 | 10850.4 | 1395.8 | 1938.9 | 22184.7 |
| 285 | | 252291_s_at | |  | **405.9** | | 1852.8 | 90.7 | 1417.5 | 19957.3 | 16021.2 |
| 286 | | [At3g22240](http://atted.jp/cgi-bin/coex_Ath.cgi?gene=At3g22240&sort=all) | |  | **406.0** | | 1310.0 | 5120.3 | 513.7 | 21.8 | 6721.9 |
| 287 | | [At3g52430](http://atted.jp/cgi-bin/coex_Ath.cgi?gene=At3g52430&sort=all) | | PAD4 | **406.9** | | 744.3 | 1440.0 | 832.9 | 266.9 | 19431.4 |
| 288 | | [At5g06530](http://atted.jp/cgi-bin/coex_Ath.cgi?gene=At5g06530&sort=all) | | transporter | **410.3** | | 158.8 | 2355.7 | 20429.7 | 4238.5 | 16735.3 |
| 289 | | [At5g09660](http://atted.jp/cgi-bin/coex_Ath.cgi?gene=At5g09660&sort=all) | | PMDH2 | **412.6** | | 434.3 | 18925.3 | 19178.2 | 13897.6 | 15313.5 |
| 290 | | [At3g24140](http://atted.jp/cgi-bin/coex_Ath.cgi?gene=At3g24140&sort=all) | | FMA | **414.2** | | 490.6 | 16729.1 | 9471.0 | 17532.9 | 1168.3 |
| 291 | | [At1g12320](http://atted.jp/cgi-bin/coex_Ath.cgi?gene=At1g12320&sort=all) | |  | **416.8** | | 426.5 | 11710.5 | 1899.7 | 17075.0 | 7201.7 |
| 292 | | [At1g74640](http://atted.jp/cgi-bin/coex_Ath.cgi?gene=At1g74640&sort=all) | |  | **416.8** | | 680.8 | 7229.2 | 4537.4 | 5859.5 | 13919.0 |
| 293 | | [At1g06650](http://atted.jp/cgi-bin/coex_Ath.cgi?gene=At1g06650&sort=all) | | dioxygenase | **418.9** | | 505.4 | 4377.6 | 3479.3 | 13857.8 | 18640.9 |
| 294 | | [At4g21210](http://atted.jp/cgi-bin/coex_Ath.cgi?gene=At4g21210&sort=all) | | ATRP1 | **421.0** | | 546.5 | 17874.6 | 11100.6 | 11196.4 | 11196.9 |
| 295 | | [At1g07000](http://atted.jp/cgi-bin/coex_Ath.cgi?gene=At1g07000&sort=all) | | EXO70B2 | **422.7** | | 1066.0 | 409.8 | 2350.0 | 155.2 | 13333.6 |
| 296 | | 246927_s_at | |  | **424.0** | | 333.9 | 593.5 | 2029.0 | 1174.7 | 15595.0 |
| 297 | | [At4g21940](http://atted.jp/cgi-bin/coex_Ath.cgi?gene=At4g21940&sort=all) | | CPK15 | **427.0** | | 259.3 | 1213.6 | 8202.8 | 9813.4 | 4814.7 |
| 298 | | [At5g17990](http://atted.jp/cgi-bin/coex_Ath.cgi?gene=At5g17990&sort=all) | | TRP1 | **427.2** | | 2528.2 | 924.0 | 1006.3 | 1284.1 | 16793.4 |
| 299 | | [At4g02410](http://atted.jp/cgi-bin/coex_Ath.cgi?gene=At4g02410&sort=all) | | kinase | **428.5** | | 732.1 | 455.2 | 4211.3 | 8197.2 | 15678.3 |
| 300 | | [At1g65540](http://atted.jp/cgi-bin/coex_Ath.cgi?gene=At1g65540&sort=all) | | calcium-binding EF hand | **429.9** | | 932.1 | 1604.5 | 194.5 | 5357.6 | 22345.5 |
|  | | | | | | | | | | | |
| **300 coexpressed gene with At1g75050** | | | | | | | | | | | |
|  | | **locus** | | **Short description** | **MR**  **(all)** | | **MR**  **(tissue)** | **MR**  **(abiotic)** | **MR**  **(biotic)** | **MR**  **(hormone)** | **MR**  **(light)** |
| 1 | | [At5g40260](http://atted.jp/cgi-bin/coex_Ath.cgi?gene=At5g40260&sort=all) | | MtN3 | **2.6** | | 2.8 | 1917.2 | 9298.2 | 1249.1 | 17290.4 |
| 2 | | [At1g07340](http://atted.jp/cgi-bin/coex_Ath.cgi?gene=At1g07340&sort=all) | | STP2 | **7.2** | | 15.2 | 1039.0 | 1495.7 | 748.2 | 18164.9 |
| 3 | | [At5g15720](http://atted.jp/cgi-bin/coex_Ath.cgi?gene=At5g15720&sort=all) | | GLIP7 | **9.5** | | 13.7 | 883.2 | 4434.5 | 3197.6 | 18734.1 |
| 4 | | [At5g54010](http://atted.jp/cgi-bin/coex_Ath.cgi?gene=At5g54010&sort=all) | | transferase | **12.0** | | 11.8 | 10225.8 | 7132.6 | 7783.4 | 4538.7 |
| 5 | | [At1g08065](http://atted.jp/cgi-bin/coex_Ath.cgi?gene=At1g08065&sort=all) | | ACA5 | **17.6** | | 13.5 | 39.5 | 1120.8 | 2393.4 | 13114.2 |
| 6 | | [At1g56360](http://atted.jp/cgi-bin/coex_Ath.cgi?gene=At1g56360&sort=all) | | PAP6 | **17.9** | | 17.0 | 1709.2 | 4681.8 | 2719.5 | 1789.4 |
| 7 | | [At2g16910](http://atted.jp/cgi-bin/coex_Ath.cgi?gene=At2g16910&sort=all) | | AMS | **18.2** | | 30.4 | 50.2 | 10151.2 | 1692.2 | 10804.3 |
| 8 | | [At3g21000](http://atted.jp/cgi-bin/coex_Ath.cgi?gene=At3g21000&sort=all) | |  | **20.1** | | 25.9 | 101.3 | 15077.8 | 11481.6 | 2415.3 |
| 9 | | [At5g53190](http://atted.jp/cgi-bin/coex_Ath.cgi?gene=At5g53190&sort=all) | | MtN3 | **21.4** | | 22.6 | 9.6 | 375.4 | 4355.1 | 5384.6 |
| 10 | | [At1g75790](http://atted.jp/cgi-bin/coex_Ath.cgi?gene=At1g75790&sort=all) | | sks18 | **21.5** | | 50.5 | 29.4 | 4005.2 | 3735.8 | 1855.0 |
| 11 | | [At5g09500](http://atted.jp/cgi-bin/coex_Ath.cgi?gene=At5g09500&sort=all) | | RPS15C | **24.0** | | 46.4 | 4778.0 | 71.6 | 4283.3 | 931.4 |
| 12 | | [At5g20330](http://atted.jp/cgi-bin/coex_Ath.cgi?gene=At5g20330&sort=all) | | BETAG4 | **24.0** | | 38.1 | 7674.2 | 1340.7 | 5113.9 | 14533.8 |
| 13 | | [At1g22015](http://atted.jp/cgi-bin/coex_Ath.cgi?gene=At1g22015&sort=all) | | DD46 | **30.5** | | 42.4 | 1201.2 | 852.9 | 2927.1 | 6756.3 |
| 14 | | [At1g06250](http://atted.jp/cgi-bin/coex_Ath.cgi?gene=At1g06250&sort=all) | | lipase | **31.4** | | 46.7 | 8002.9 | 1137.3 | 675.7 | 11500.6 |
| 15 | | [At1g13140](http://atted.jp/cgi-bin/coex_Ath.cgi?gene=At1g13140&sort=all) | | CYP86C3 | **33.3** | | 58.6 | 2840.9 | 1847.5 | 4511.2 | 9847.5 |
| 16 | | [At5g40040](http://atted.jp/cgi-bin/coex_Ath.cgi?gene=At5g40040&sort=all) | | RPP2E | **35.1** | | 25.5 | 9070.0 | 482.8 | 2074.2 | 2352.3 |
| 17 | | [At4g27330](http://atted.jp/cgi-bin/coex_Ath.cgi?gene=At4g27330&sort=all) | | SPL | **36.1** | | 32.8 | 533.3 | 4460.3 | 1975.2 | 21868.4 |
| 18 | | [At4g00920](http://atted.jp/cgi-bin/coex_Ath.cgi?gene=At4g00920&sort=all) | | COP1-interacting | **36.6** | | 55.2 | 8190.8 | 4090.8 | 2448.9 | 7880.9 |
| 19 | | [At1g18280](http://atted.jp/cgi-bin/coex_Ath.cgi?gene=At1g18280&sort=all) | | LTP | **37.8** | | 52.7 | 1978.5 | 729.7 | 2219.7 | 6722.2 |
| 20 | | [At4g08670](http://atted.jp/cgi-bin/coex_Ath.cgi?gene=At4g08670&sort=all) | | LTP | **38.0** | | 90.9 | 2901.0 | 2787.1 | 901.7 | 18430.7 |
| 21 | | [At1g79780](http://atted.jp/cgi-bin/coex_Ath.cgi?gene=At1g79780&sort=all) | |  | **40.4** | | 27.4 | 303.0 | 6465.0 | 4829.3 | 548.9 |
| 22 | | [At5g20710](http://atted.jp/cgi-bin/coex_Ath.cgi?gene=At5g20710&sort=all) | | BGAL7 | **40.4** | | 57.0 | 2497.7 | 340.6 | 7689.4 | 7627.0 |
| 23 | | [At4g26830](http://atted.jp/cgi-bin/coex_Ath.cgi?gene=At4g26830&sort=all) | | hydrolase | **41.5** | | 55.4 | 4648.8 | 11563.9 | 2554.6 | 742.1 |
| 24 | | [At4g14815](http://atted.jp/cgi-bin/coex_Ath.cgi?gene=At4g14815&sort=all) | | LTP | **41.8** | | 68.8 | 344.3 | 313.5 | 2454.4 | 12661.6 |
| 25 | | [At1g74540](http://atted.jp/cgi-bin/coex_Ath.cgi?gene=At1g74540&sort=all) | | CYP98A8 | **43.2** | | 71.0 | 3366.1 | 718.7 | 3044.5 | 6939.3 |
| 26 | | [At2g28680](http://atted.jp/cgi-bin/coex_Ath.cgi?gene=At2g28680&sort=all) | | cupin | **45.1** | | 10.7 | 1197.5 | 771.8 | 386.7 | 3223.5 |
| 27 | | [At1g06170](http://atted.jp/cgi-bin/coex_Ath.cgi?gene=At1g06170&sort=all) | | bHLH | **46.3** | | 64.5 | 3424.5 | 12554.2 | 405.6 | 12664.4 |
| 28 | | [At2g03740](http://atted.jp/cgi-bin/coex_Ath.cgi?gene=At2g03740&sort=all) | | LEA | **47.0** | | 63.2 | 6400.7 | 2041.3 | 1136.0 | 568.0 |
| 29 | | [At5g43340](http://atted.jp/cgi-bin/coex_Ath.cgi?gene=At5g43340&sort=all) | | PHT6 | **51.8** | | 32.1 | 2896.2 | 962.9 | 518.1 | 4538.3 |
| 30 | | [At1g28710](http://atted.jp/cgi-bin/coex_Ath.cgi?gene=At1g28710&sort=all) | |  | **53.3** | | 73.5 | 12781.2 | 11974.8 | 12060.7 | 11737.2 |
| 31 | | [At1g61110](http://atted.jp/cgi-bin/coex_Ath.cgi?gene=At1g61110&sort=all) | | anac025 | **54.9** | | 63.2 | 2346.8 | 2621.7 | 2261.8 | 19140.4 |
| 32 | | [At1g28375](http://atted.jp/cgi-bin/coex_Ath.cgi?gene=At1g28375&sort=all) | |  | **55.4** | | 83.2 | 1017.1 | 10107.9 | 2197.2 | 17336.1 |
| 33 | | [At1g33430](http://atted.jp/cgi-bin/coex_Ath.cgi?gene=At1g33430&sort=all) | | transferase | **57.2** | | 79.4 | 40.8 | 502.5 | 1035.4 | 17912.8 |
| 34 | | [At3g15400](http://atted.jp/cgi-bin/coex_Ath.cgi?gene=At3g15400&sort=all) | | ATA20 | **57.3** | | 85.5 | 25.7 | 11875.3 | 3387.5 | 3692.6 |
| 35 | | [At3g51590](http://atted.jp/cgi-bin/coex_Ath.cgi?gene=At3g51590&sort=all) | | LTP12 | **59.0** | | 90.8 | 2249.5 | 5224.1 | 7123.0 | 561.3 |
| 36 | | [At1g75940](http://atted.jp/cgi-bin/coex_Ath.cgi?gene=At1g75940&sort=all) | | ATA27 | **64.3** | | 109.5 | 1870.9 | 1132.5 | 1431.3 | 2568.0 |
| 37 | | [At4g10260](http://atted.jp/cgi-bin/coex_Ath.cgi?gene=At4g10260&sort=all) | | kinase | **64.3** | | 51.2 | 772.8 | 3068.7 | 6475.4 | 22527.0 |
| 38 | | [At5g07520](http://atted.jp/cgi-bin/coex_Ath.cgi?gene=At5g07520&sort=all) | | GRP18 | **65.3** | | 91.8 | 5613.4 | 2867.8 | 3291.4 | 3357.8 |
| 39 | | [At5g61940](http://atted.jp/cgi-bin/coex_Ath.cgi?gene=At5g61940&sort=all) | | hydrolase | **65.4** | | 64.9 | 10560.5 | 10110.7 | 9859.7 | 6635.6 |
| 40 | | [At1g80660](http://atted.jp/cgi-bin/coex_Ath.cgi?gene=At1g80660&sort=all) | | AHA9 | **65.8** | | 111.7 | 4302.6 | 5461.0 | 3106.9 | 5372.1 |
| 41 | | [At5g03620](http://atted.jp/cgi-bin/coex_Ath.cgi?gene=At5g03620&sort=all) | | subtilase | **65.8** | | 118.1 | 475.6 | 1478.3 | 216.5 | 1568.0 |
| 42 | | [At1g48940](http://atted.jp/cgi-bin/coex_Ath.cgi?gene=At1g48940&sort=all) | | plastocyanin-like | **66.1** | | 86.0 | 2397.3 | 4355.7 | 2694.7 | 10510.0 |
| 43 | | [At1g75910](http://atted.jp/cgi-bin/coex_Ath.cgi?gene=At1g75910&sort=all) | | EXL4 | **67.5** | | 99.0 | 1946.4 | 11962.1 | 3800.2 | 18536.9 |
| 44 | | [At1g06990](http://atted.jp/cgi-bin/coex_Ath.cgi?gene=At1g06990&sort=all) | | hydrolase | **67.9** | | 89.4 | 7487.1 | 11050.0 | 7014.7 | 9186.9 |
| 45 | | [At1g74550](http://atted.jp/cgi-bin/coex_Ath.cgi?gene=At1g74550&sort=all) | | CYP98A9 | **67.9** | | 71.9 | 1043.8 | 5880.7 | 3444.9 | 5114.0 |
| 46 | | [At3g50580](http://atted.jp/cgi-bin/coex_Ath.cgi?gene=At3g50580&sort=all) | |  | **68.7** | | 74.1 | 17.7 | 6506.9 | 13918.3 | 2691.9 |
| 47 | | [At3g57620](http://atted.jp/cgi-bin/coex_Ath.cgi?gene=At3g57620&sort=all) | | oxidase | **69.0** | | 83.6 | 4004.4 | 2086.9 | 3035.0 | 3835.7 |
| 48 | | [At1g44224](http://atted.jp/cgi-bin/coex_Ath.cgi?gene=At1g44224&sort=all) | |  | **70.7** | | 57.5 | 5426.7 | 5120.1 | 7201.8 | 3200.3 |
| 49 | | 263927_s_at | |  | **71.1** | | 30.9 | 10343.7 | 9317.4 | 9422.9 | 13259.7 |
| 50 | | [At1g68875](http://atted.jp/cgi-bin/coex_Ath.cgi?gene=At1g68875&sort=all) | |  | **72.6** | | 114.0 | 6541.9 | 5401.3 | 11139.2 | 4551.0 |
| 51 | | [At5g07560](http://atted.jp/cgi-bin/coex_Ath.cgi?gene=At5g07560&sort=all) | | GRP20 | **72.8** | | 109.4 | 6711.7 | 5073.7 | 1436.4 | 743.6 |
| 52 | | [At1g22760](http://atted.jp/cgi-bin/coex_Ath.cgi?gene=At1g22760&sort=all) | | PAB3 | **73.3** | | 120.1 | 1768.3 | 1014.3 | 366.1 | 4090.3 |
| 53 | | [At2g39590](http://atted.jp/cgi-bin/coex_Ath.cgi?gene=At2g39590&sort=all) | | RPS15aC | **73.8** | | 73.0 | 1785.3 | 3684.6 | 4782.5 | 346.9 |
| 54 | | [At3g58290](http://atted.jp/cgi-bin/coex_Ath.cgi?gene=At3g58290&sort=all) | | MATH | **74.1** | | 97.4 | 1971.5 | 417.4 | 1634.9 | 16175.3 |
| 55 | | [At1g26710](http://atted.jp/cgi-bin/coex_Ath.cgi?gene=At1g26710&sort=all) | |  | **74.9** | | 109.5 | 4087.8 | 9319.3 | 9154.1 | 20591.4 |
| 56 | | [At1g13150](http://atted.jp/cgi-bin/coex_Ath.cgi?gene=At1g13150&sort=all) | | CYP86C4 | **75.0** | | 99.2 | 4500.3 | 2442.9 | 7847.2 | 3422.3 |
| 57 | | [At1g75930](http://atted.jp/cgi-bin/coex_Ath.cgi?gene=At1g75930&sort=all) | | EXL6 | **76.6** | | 112.9 | 4378.2 | 1870.8 | 4600.4 | 13419.8 |
| 58 | | [At1g22010](http://atted.jp/cgi-bin/coex_Ath.cgi?gene=At1g22010&sort=all) | |  | **76.7** | | 74.5 | 8720.3 | 4905.6 | 1090.1 | 6623.6 |
| 59 | | [At5g48210](http://atted.jp/cgi-bin/coex_Ath.cgi?gene=At5g48210&sort=all) | |  | **83.5** | | 123.8 | 1420.4 | 783.2 | 2808.3 | 12438.7 |
| 60 | | [At1g75890](http://atted.jp/cgi-bin/coex_Ath.cgi?gene=At1g75890&sort=all) | | EXL2 | **83.6** | | 74.6 | 10978.4 | 2522.7 | 1696.0 | 3145.0 |
| 61 | | [At1g01280](http://atted.jp/cgi-bin/coex_Ath.cgi?gene=At1g01280&sort=all) | | CYP703A2 | **83.8** | | 138.5 | 16.9 | 5317.2 | 2992.8 | 94.3 |
| 62 | | 247639_s_at | |  | **85.0** | | 125.0 | 5032.0 | 2167.6 | 14076.4 | 74.0 |
| 63 | | [At1g71770](http://atted.jp/cgi-bin/coex_Ath.cgi?gene=At1g71770&sort=all) | | PAB5 | **85.1** | | 129.2 | 1466.7 | 3467.4 | 2814.7 | 5462.7 |
| 64 | | [At1g66850](http://atted.jp/cgi-bin/coex_Ath.cgi?gene=At1g66850&sort=all) | | LTP | **85.8** | | 118.8 | 408.4 | 4229.0 | 7699.0 | 2042.0 |
| 65 | | [At5g07510](http://atted.jp/cgi-bin/coex_Ath.cgi?gene=At5g07510&sort=all) | | GRP14 | **86.4** | | 102.4 | 3750.6 | 9796.8 | 19338.4 | 948.4 |
| 66 | | [At5g16920](http://atted.jp/cgi-bin/coex_Ath.cgi?gene=At5g16920&sort=all) | |  | **86.7** | | 109.3 | 525.3 | 3651.6 | 3281.6 | 7095.0 |
| 67 | | [At3g23770](http://atted.jp/cgi-bin/coex_Ath.cgi?gene=At3g23770&sort=all) | | hydrolase | **88.0** | | 129.0 | 5.5 | 5234.6 | 9479.4 | 1918.6 |
| 68 | | [At3g59530](http://atted.jp/cgi-bin/coex_Ath.cgi?gene=At3g59530&sort=all) | | synthase | **90.2** | | 85.1 | 1958.5 | 3741.8 | 7825.7 | 6388.4 |
| 69 | | [At1g06260](http://atted.jp/cgi-bin/coex_Ath.cgi?gene=At1g06260&sort=all) | | cysteinease | **90.3** | | 135.4 | 3376.4 | 475.0 | 2857.5 | 3067.9 |
| 70 | | [At1g75920](http://atted.jp/cgi-bin/coex_Ath.cgi?gene=At1g75920&sort=all) | | EXL5 | **91.0** | | 113.9 | 6182.5 | 210.6 | 392.9 | 5492.4 |
| 71 | | 255609_s_at | |  | **91.4** | | 135.9 | 3117.7 | 5018.2 | 1632.1 | 2133.0 |
| 72 | | [At4g27910](http://atted.jp/cgi-bin/coex_Ath.cgi?gene=At4g27910&sort=all) | | SDG16 | **92.3** | | 54.1 | 430.7 | 5614.3 | 1314.4 | 19911.1 |
| 73 | | [At4g25950](http://atted.jp/cgi-bin/coex_Ath.cgi?gene=At4g25950&sort=all) | | VATG3 | **94.9** | | 159.6 | 5903.2 | 5065.0 | 4389.1 | 2486.3 |
| 74 | | [At4g28395](http://atted.jp/cgi-bin/coex_Ath.cgi?gene=At4g28395&sort=all) | | ATA7 | **96.1** | | 108.0 | 1339.6 | 4446.2 | 2350.6 | 4043.4 |
| 75 | | [At2g19070](http://atted.jp/cgi-bin/coex_Ath.cgi?gene=At2g19070&sort=all) | | SHT | **97.7** | | 134.5 | 453.8 | 4841.2 | 13099.9 | 1683.0 |
| 76 | | [263368_at](http://atted.jp/cgi-bin/coex_Ath.cgi?gene=263368_at&sort=all) | |  | **98.3** | | 118.8 | 2710.9 | 3246.8 | 1925.6 | 1050.5 |
| 77 | | [At1g23670](http://atted.jp/cgi-bin/coex_Ath.cgi?gene=At1g23670&sort=all) | |  | **99.9** | | 126.5 | 763.6 | 5531.5 | 2762.3 | 3943.9 |
| 78 | | [At1g47980](http://atted.jp/cgi-bin/coex_Ath.cgi?gene=At1g47980&sort=all) | |  | **101.9** | | 145.2 | 139.8 | 2931.1 | 4224.7 | 11549.4 |
| 79 | | [At4g28580](http://atted.jp/cgi-bin/coex_Ath.cgi?gene=At4g28580&sort=all) | | MRS2-6 | **101.9** | | 51.0 | 5309.6 | 3886.2 | 6603.7 | 8489.4 |
| 80 | | [At5g62320](http://atted.jp/cgi-bin/coex_Ath.cgi?gene=At5g62320&sort=all) | | MYB99 | **103.1** | | 101.0 | 1058.5 | 6106.8 | 3657.2 | 2715.4 |
| 81 | | [At4g12920](http://atted.jp/cgi-bin/coex_Ath.cgi?gene=At4g12920&sort=all) | | protease | **103.1** | | 107.8 | 170.6 | 8876.7 | 4172.0 | 12986.8 |
| 82 | | [At1g71160](http://atted.jp/cgi-bin/coex_Ath.cgi?gene=At1g71160&sort=all) | | KCS7 | **104.0** | | 148.8 | 1201.7 | 12240.9 | 2685.3 | 10329.1 |
| 83 | | [At5g59810](http://atted.jp/cgi-bin/coex_Ath.cgi?gene=At5g59810&sort=all) | | SBT5.4 | **105.0** | | 81.2 | 3548.9 | 1610.7 | 1416.7 | 1062.0 |
| 84 | | [At1g50310](http://atted.jp/cgi-bin/coex_Ath.cgi?gene=At1g50310&sort=all) | | STP9 | **107.1** | | 136.6 | 2386.7 | 6993.8 | 1407.0 | 7896.6 |
| 85 | | [At5g24820](http://atted.jp/cgi-bin/coex_Ath.cgi?gene=At5g24820&sort=all) | | protease | **107.9** | | 175.4 | 55.7 | 1773.8 | 728.4 | 3513.5 |
| 86 | | [At3g25050](http://atted.jp/cgi-bin/coex_Ath.cgi?gene=At3g25050&sort=all) | | XTH3 | **108.0** | | 163.7 | 1688.4 | 9422.9 | 601.0 | 16721.5 |
| 87 | | [At4g36350](http://atted.jp/cgi-bin/coex_Ath.cgi?gene=At4g36350&sort=all) | | PAP25 | **109.5** | | 53.8 | 7111.6 | 319.7 | 6232.1 | 3521.2 |
| 88 | | [At4g20050](http://atted.jp/cgi-bin/coex_Ath.cgi?gene=At4g20050&sort=all) | | QRT3 | **109.5** | | 176.4 | 17.4 | 4896.3 | 2566.8 | 2355.2 |
| 89 | | [At5g10420](http://atted.jp/cgi-bin/coex_Ath.cgi?gene=At5g10420&sort=all) | | transporter | **110.9** | | 98.8 | 4362.6 | 1331.2 | 462.6 | 18351.6 |
| 90 | | [At3g26140](http://atted.jp/cgi-bin/coex_Ath.cgi?gene=At3g26140&sort=all) | | cellulase | **114.1** | | 187.8 | 514.2 | 4776.4 | 1893.0 | 3324.1 |
| 91 | | [At5g25400](http://atted.jp/cgi-bin/coex_Ath.cgi?gene=At5g25400&sort=all) | | translocator | **114.2** | | 148.8 | 2397.3 | 4205.2 | 793.3 | 11249.7 |
| 92 | | [At2g33880](http://atted.jp/cgi-bin/coex_Ath.cgi?gene=At2g33880&sort=all) | | HB-3 | **114.9** | | 112.5 | 586.6 | 2765.1 | 6810.1 | 4460.7 |
| 93 | | [At3g42960](http://atted.jp/cgi-bin/coex_Ath.cgi?gene=At3g42960&sort=all) | | ATA1 | **115.4** | | 179.5 | 16.2 | 4050.0 | 1463.5 | 3688.3 |
| 94 | | [At3g50570](http://atted.jp/cgi-bin/coex_Ath.cgi?gene=At3g50570&sort=all) | | glycoprotein | **115.9** | | 50.8 | 1429.5 | 6597.7 | 11206.6 | 12214.3 |
| 95 | | [At5g17340](http://atted.jp/cgi-bin/coex_Ath.cgi?gene=At5g17340&sort=all) | |  | **116.0** | | 54.3 | 10456.8 | 3466.8 | 18482.0 | 3343.7 |
| 96 | | [At5g07540](http://atted.jp/cgi-bin/coex_Ath.cgi?gene=At5g07540&sort=all) | | GRP16 | **116.9** | | 165.6 | 598.3 | 1263.3 | 16815.9 | 3719.3 |
| 97 | | [At1g23570](http://atted.jp/cgi-bin/coex_Ath.cgi?gene=At1g23570&sort=all) | |  | **116.9** | | 159.2 | 12187.7 | 13686.0 | 16329.4 | 1721.6 |
| 98 | | [At5g03250](http://atted.jp/cgi-bin/coex_Ath.cgi?gene=At5g03250&sort=all) | | NPH3 | **120.0** | | 152.9 | 5759.9 | 1292.5 | 4977.0 | 1030.7 |
| 99 | | [At3g52810](http://atted.jp/cgi-bin/coex_Ath.cgi?gene=At3g52810&sort=all) | | PAP21 | **121.0** | | 168.2 | 9820.7 | 3361.2 | 2033.8 | 7372.9 |
| 100 | | [At3g11980](http://atted.jp/cgi-bin/coex_Ath.cgi?gene=At3g11980&sort=all) | | MS2 | **122.1** | | 166.1 | 97.1 | 3317.1 | 5526.7 | 4309.4 |
| 101 | | [At3g52160](http://atted.jp/cgi-bin/coex_Ath.cgi?gene=At3g52160&sort=all) | | KCS15 | **122.2** | | 177.4 | 593.1 | 1753.4 | 3051.0 | 16034.8 |
| 102 | | [At4g10440](http://atted.jp/cgi-bin/coex_Ath.cgi?gene=At4g10440&sort=all) | | dehydration-responsive | **122.3** | | 156.3 | 3052.3 | 7117.3 | 841.9 | 18923.7 |
| 103 | | [At5g07530](http://atted.jp/cgi-bin/coex_Ath.cgi?gene=At5g07530&sort=all) | | GRP17 | **123.0** | | 145.9 | 1080.1 | 19847.6 | 18207.3 | 1333.5 |
| 104 | | [At5g52160](http://atted.jp/cgi-bin/coex_Ath.cgi?gene=At5g52160&sort=all) | | LTP | **125.0** | | 187.1 | 1384.3 | 283.1 | 4298.0 | 6584.8 |
| 105 | | [At1g67990](http://atted.jp/cgi-bin/coex_Ath.cgi?gene=At1g67990&sort=all) | | TSM1 | **125.4** | | 185.1 | 5598.0 | 4055.4 | 4284.8 | 13578.2 |
| 106 | | [At2g17950](http://atted.jp/cgi-bin/coex_Ath.cgi?gene=At2g17950&sort=all) | | WUS | **126.4** | | 137.7 | 5370.0 | 5907.5 | 4518.5 | 19915.2 |
| 107 | | [At1g20150](http://atted.jp/cgi-bin/coex_Ath.cgi?gene=At1g20150&sort=all) | | subtilase | **126.6** | | 145.6 | 8281.7 | 3594.9 | 3660.0 | 7849.2 |
| 108 | | [At1g02813](http://atted.jp/cgi-bin/coex_Ath.cgi?gene=At1g02813&sort=all) | |  | **128.0** | | 167.2 | 2293.5 | 2115.1 | 14642.8 | 8804.9 |
| 109 | | [At5g41090](http://atted.jp/cgi-bin/coex_Ath.cgi?gene=At5g41090&sort=all) | | anac095 | **128.7** | | 57.3 | 7876.4 | 4547.6 | 2212.2 | 8646.8 |
| 110 | | [At1g61070](http://atted.jp/cgi-bin/coex_Ath.cgi?gene=At1g61070&sort=all) | | LCR66 | **128.9** | | 196.8 | 46.1 | 3147.2 | 15900.8 | 2025.8 |
| 111 | | [At1g73050](http://atted.jp/cgi-bin/coex_Ath.cgi?gene=At1g73050&sort=all) | | oxynitrilase | **129.4** | | 139.6 | 1108.2 | 208.1 | 1382.3 | 4404.3 |
| 112 | | [At4g29250](http://atted.jp/cgi-bin/coex_Ath.cgi?gene=At4g29250&sort=all) | | transferase | **129.6** | | 145.8 | 391.9 | 2542.3 | 1392.8 | 4168.7 |
| 113 | | [At3g52130](http://atted.jp/cgi-bin/coex_Ath.cgi?gene=At3g52130&sort=all) | | LTP | **131.2** | | 176.8 | 46.4 | 947.5 | 6145.9 | 2977.9 |
| 114 | | [At2g07560](http://atted.jp/cgi-bin/coex_Ath.cgi?gene=At2g07560&sort=all) | | AHA6 | **131.2** | | 209.1 | 2166.9 | 5722.1 | 1749.9 | 3356.8 |
| 115 | | [At4g02140](http://atted.jp/cgi-bin/coex_Ath.cgi?gene=At4g02140&sort=all) | |  | **131.4** | | 166.8 | 4977.9 | 1968.9 | 5244.4 | 2659.0 |
| 116 | | [At5g07550](http://atted.jp/cgi-bin/coex_Ath.cgi?gene=At5g07550&sort=all) | | GRP19 | **131.6** | | 177.6 | 4222.2 | 1797.5 | 19409.7 | 2344.2 |
| 117 | | [At1g04880](http://atted.jp/cgi-bin/coex_Ath.cgi?gene=At1g04880&sort=all) | | HMG1/2 | **132.2** | | 212.1 | 1311.0 | 2847.3 | 716.3 | 3676.8 |
| 118 | | [At5g17200](http://atted.jp/cgi-bin/coex_Ath.cgi?gene=At5g17200&sort=all) | | pectinase | **132.5** | | 118.4 | 838.5 | 567.8 | 6176.3 | 4271.3 |
| 119 | | [At1g30020](http://atted.jp/cgi-bin/coex_Ath.cgi?gene=At1g30020&sort=all) | |  | **134.0** | | 191.9 | 3080.0 | 3942.4 | 5276.6 | 5223.6 |
| 120 | | [At4g34850](http://atted.jp/cgi-bin/coex_Ath.cgi?gene=At4g34850&sort=all) | | synthase | **134.7** | | 167.6 | 42.9 | 5522.8 | 5525.1 | 214.0 |
| 121 | | 257400_s_at | |  | **135.8** | | 183.9 | 3395.0 | 3127.0 | 1987.1 | 6494.1 |
| 122 | | [At2g40250](http://atted.jp/cgi-bin/coex_Ath.cgi?gene=At2g40250&sort=all) | | hydrolase | **136.9** | | 122.5 | 2596.2 | 5496.9 | 1806.9 | 7546.1 |
| 123 | | [At2g42940](http://atted.jp/cgi-bin/coex_Ath.cgi?gene=At2g42940&sort=all) | | DNA-binding | **137.0** | | 137.2 | 2424.2 | 906.8 | 1659.2 | 3395.6 |
| 124 | | [At3g05960](http://atted.jp/cgi-bin/coex_Ath.cgi?gene=At3g05960&sort=all) | | STP6 | **137.4** | | 197.6 | 3819.6 | 2556.9 | 184.6 | 18515.5 |
| 125 | | [At1g79360](http://atted.jp/cgi-bin/coex_Ath.cgi?gene=At1g79360&sort=all) | | OCT2 | **137.5** | | 219.5 | 3426.6 | 1333.8 | 2455.4 | 19031.0 |
| 126 | | [At1g23240](http://atted.jp/cgi-bin/coex_Ath.cgi?gene=At1g23240&sort=all) | | caleosin | **137.9** | | 212.6 | 1632.0 | 213.8 | 1136.4 | 7194.8 |
| 127 | | [At5g07230](http://atted.jp/cgi-bin/coex_Ath.cgi?gene=At5g07230&sort=all) | | LTP | **138.1** | | 178.3 | 210.4 | 1201.7 | 6023.6 | 1024.7 |
| 128 | | [At1g30350](http://atted.jp/cgi-bin/coex_Ath.cgi?gene=At1g30350&sort=all) | | lyase | **138.2** | | 184.5 | 6929.6 | 4187.0 | 390.3 | 11883.2 |
| 129 | | [At3g13220](http://atted.jp/cgi-bin/coex_Ath.cgi?gene=At3g13220&sort=all) | | WBC27 | **139.4** | | 172.5 | 80.4 | 4612.0 | 4176.5 | 1614.6 |
| 130 | | [At1g62940](http://atted.jp/cgi-bin/coex_Ath.cgi?gene=At1g62940&sort=all) | | ACOS5 | **142.3** | | 147.0 | 64.3 | 1319.1 | 1135.0 | 1271.5 |
| 131 | | [At2g31210](http://atted.jp/cgi-bin/coex_Ath.cgi?gene=At2g31210&sort=all) | | bHLH | **143.0** | | 93.5 | 7271.0 | 12238.0 | 6524.8 | 3240.3 |
| 132 | | [At5g61605](http://atted.jp/cgi-bin/coex_Ath.cgi?gene=At5g61605&sort=all) | |  | **143.2** | | 200.0 | 10790.3 | 1882.4 | 1527.7 | 9876.9 |
| 133 | | [At1g23420](http://atted.jp/cgi-bin/coex_Ath.cgi?gene=At1g23420&sort=all) | | INO | **143.4** | | 175.0 | 2262.7 | 5217.7 | 4804.4 | 7890.5 |
| 134 | | [At1g28430](http://atted.jp/cgi-bin/coex_Ath.cgi?gene=At1g28430&sort=all) | | CYP705A24 | **143.5** | | 205.5 | 1282.2 | 3252.1 | 8370.2 | 7076.9 |
| 135 | | [At5g49070](http://atted.jp/cgi-bin/coex_Ath.cgi?gene=At5g49070&sort=all) | | KCS21 | **143.9** | | 184.8 | 1769.3 | 2916.5 | 1620.7 | 20643.2 |
| 136 | | [At4g10950](http://atted.jp/cgi-bin/coex_Ath.cgi?gene=At4g10950&sort=all) | | hydrolase | **146.1** | | 115.8 | 2072.4 | 6521.8 | 13710.2 | 2509.8 |
| 137 | | 257862_s_at | |  | **146.2** | | 231.9 | 2653.4 | 18649.9 | 5956.1 | 13001.2 |
| 138 | | [At4g28680](http://atted.jp/cgi-bin/coex_Ath.cgi?gene=At4g28680&sort=all) | | TYRDC1 | **150.3** | | 356.8 | 838.9 | 903.6 | 2305.5 | 9929.7 |
| 139 | | [At4g14080](http://atted.jp/cgi-bin/coex_Ath.cgi?gene=At4g14080&sort=all) | | MEE48 | **152.4** | | 195.7 | 25.8 | 3044.1 | 4498.1 | 7359.7 |
| 140 | | [At3g23130](http://atted.jp/cgi-bin/coex_Ath.cgi?gene=At3g23130&sort=all) | | SUP | **153.6** | | 134.6 | 3998.4 | 608.1 | 3291.9 | 2771.7 |
| 141 | | [At4g20420](http://atted.jp/cgi-bin/coex_Ath.cgi?gene=At4g20420&sort=all) | | tapetum-specific | **153.6** | | 134.8 | 54.0 | 1715.9 | 1623.6 | 18510.4 |
| 142 | | [At3g28470](http://atted.jp/cgi-bin/coex_Ath.cgi?gene=At3g28470&sort=all) | | TDF1 | **154.6** | | 180.7 | 7634.0 | 9276.0 | 2802.8 | 16131.0 |
| 143 | | [At2g32890](http://atted.jp/cgi-bin/coex_Ath.cgi?gene=At2g32890&sort=all) | | RALFL17 | **155.0** | | 202.6 | 1072.5 | 18409.7 | 8050.7 | 11200.8 |
| 144 | | [At4g29980](http://atted.jp/cgi-bin/coex_Ath.cgi?gene=At4g29980&sort=all) | |  | **155.2** | | 207.0 | 36.2 | 3253.5 | 1912.2 | 14795.7 |
| 145 | | [At5g53510](http://atted.jp/cgi-bin/coex_Ath.cgi?gene=At5g53510&sort=all) | | OPT9 | **156.2** | | 279.1 | 5524.1 | 6743.4 | 336.6 | 1180.0 |
| 146 | | [At5g13380](http://atted.jp/cgi-bin/coex_Ath.cgi?gene=At5g13380&sort=all) | | GH3 | **156.5** | | 229.8 | 4191.8 | 5237.0 | 3160.5 | 20842.6 |
| 147 | | [At1g23650](http://atted.jp/cgi-bin/coex_Ath.cgi?gene=At1g23650&sort=all) | |  | **156.6** | | 136.0 | 14017.0 | 3612.9 | 3749.1 | 5670.3 |
| 148 | | [At4g28700](http://atted.jp/cgi-bin/coex_Ath.cgi?gene=At4g28700&sort=all) | | AMT1;4 | **156.8** | | 101.3 | 1241.6 | 7286.1 | 7603.2 | 3055.1 |
| 149 | | [At5g59040](http://atted.jp/cgi-bin/coex_Ath.cgi?gene=At5g59040&sort=all) | | COPT3 | **157.3** | | 136.6 | 4003.7 | 14.8 | 316.9 | 7452.2 |
| 150 | | [At3g42850](http://atted.jp/cgi-bin/coex_Ath.cgi?gene=At3g42850&sort=all) | | galactokinase | **157.7** | | 129.2 | 4125.9 | 11236.1 | 10692.5 | 3287.1 |
| 151 | | [At1g23580](http://atted.jp/cgi-bin/coex_Ath.cgi?gene=At1g23580&sort=all) | |  | **158.4** | | 169.9 | 2404.6 | 6872.3 | 6976.7 | 2887.1 |
| 152 | | [At4g30040](http://atted.jp/cgi-bin/coex_Ath.cgi?gene=At4g30040&sort=all) | | protease | **158.7** | | 198.0 | 68.9 | 2597.8 | 3811.0 | 19849.2 |
| 153 | | [At2g03850](http://atted.jp/cgi-bin/coex_Ath.cgi?gene=At2g03850&sort=all) | | LEA | **160.1** | | 200.8 | 7591.2 | 3550.8 | 1855.8 | 1687.5 |
| 154 | | [At5g46795](http://atted.jp/cgi-bin/coex_Ath.cgi?gene=At5g46795&sort=all) | | MSP2 | **160.1** | | 177.5 | 3654.0 | 5492.4 | 2382.4 | 6362.4 |
| 155 | | [At3g23840](http://atted.jp/cgi-bin/coex_Ath.cgi?gene=At3g23840&sort=all) | | transferase | **162.1** | | 130.1 | 7890.6 | 7833.9 | 19540.4 | 1044.8 |
| 156 | | [At2g23800](http://atted.jp/cgi-bin/coex_Ath.cgi?gene=At2g23800&sort=all) | | GGPS2 | **162.5** | | 221.3 | 5706.6 | 4128.4 | 2736.3 | 13307.2 |
| 157 | | [At1g18520](http://atted.jp/cgi-bin/coex_Ath.cgi?gene=At1g18520&sort=all) | | TET11 | **163.5** | | 213.9 | 868.7 | 2982.1 | 4491.6 | 6392.0 |
| 158 | | [At5g21150](http://atted.jp/cgi-bin/coex_Ath.cgi?gene=At5g21150&sort=all) | | AGO9 | **165.9** | | 348.5 | 153.1 | 6330.2 | 509.0 | 2487.4 |
| 159 | | 246471_s_at | |  | **167.2** | | 459.2 | 6981.7 | 4656.6 | 5783.5 | 446.8 |
| 160 | | [At1g24400](http://atted.jp/cgi-bin/coex_Ath.cgi?gene=At1g24400&sort=all) | | LHT2 | **168.2** | | 140.0 | 19203.2 | 10282.1 | 755.5 | 6442.7 |
| 161 | | [At5g36150](http://atted.jp/cgi-bin/coex_Ath.cgi?gene=At5g36150&sort=all) | | PEN3 | **169.2** | | 115.4 | 1760.3 | 1388.3 | 4155.9 | 3441.8 |
| 162 | | 255729_at | |  | **169.3** | | 189.9 | 9153.6 | 18968.4 | 2467.5 | 21442.9 |
| 163 | | [At1g03390](http://atted.jp/cgi-bin/coex_Ath.cgi?gene=At1g03390&sort=all) | | transferase | **171.4** | | 261.1 | 215.2 | 913.8 | 3118.6 | 4976.9 |
| 164 | | 259905_s_at | |  | **172.6** | | 143.0 | 2840.8 | 3976.0 | 4794.3 | 13927.7 |
| 165 | | [At1g69500](http://atted.jp/cgi-bin/coex_Ath.cgi?gene=At1g69500&sort=all) | | CYP704B1 | **172.7** | | 267.2 | 28.7 | 4378.6 | 13461.4 | 7853.1 |
| 166 | | [At3g04620](http://atted.jp/cgi-bin/coex_Ath.cgi?gene=At3g04620&sort=all) | | nucleic acid binding | **173.0** | | 135.8 | 6039.4 | 11343.8 | 10063.1 | 2422.3 |
| 167 | | [At5g40940](http://atted.jp/cgi-bin/coex_Ath.cgi?gene=At5g40940&sort=all) | | FLA20 | **173.1** | | 230.1 | 109.1 | 9212.5 | 4987.4 | 22148.5 |
| 168 | | [At5g25950](http://atted.jp/cgi-bin/coex_Ath.cgi?gene=At5g25950&sort=all) | |  | **174.2** | | 285.0 | 2509.6 | 18260.7 | 18416.0 | 7387.0 |
| 169 | | [At1g26610](http://atted.jp/cgi-bin/coex_Ath.cgi?gene=At1g26610&sort=all) | | zinc finger | **175.3** | | 210.7 | 1620.8 | 2213.0 | 2129.3 | 17173.3 |
| 170 | | [At3g21970](http://atted.jp/cgi-bin/coex_Ath.cgi?gene=At3g21970&sort=all) | | kinase | **176.6** | | 274.2 | 686.6 | 2568.0 | 875.2 | 1944.5 |
| 171 | | [At1g79800](http://atted.jp/cgi-bin/coex_Ath.cgi?gene=At1g79800&sort=all) | | plastocyanin-like | **177.4** | | 71.9 | 1708.0 | 4984.2 | 1314.2 | 10708.8 |
| 172 | | [At1g15460](http://atted.jp/cgi-bin/coex_Ath.cgi?gene=At1g15460&sort=all) | | BOR4 | **178.5** | | 129.6 | 4366.4 | 3972.0 | 3920.5 | 11630.6 |
| 173 | | [At5g14980](http://atted.jp/cgi-bin/coex_Ath.cgi?gene=At5g14980&sort=all) | | thioesterase | **178.8** | | 194.0 | 6295.2 | 4131.3 | 5513.7 | 3853.2 |
| 174 | | [At3g18360](http://atted.jp/cgi-bin/coex_Ath.cgi?gene=At3g18360&sort=all) | | VQ motif | **180.0** | | 215.6 | 932.2 | 2448.0 | 1268.7 | 6943.7 |
| 175 | | [At1g78160](http://atted.jp/cgi-bin/coex_Ath.cgi?gene=At1g78160&sort=all) | | APUM7 | **184.2** | | 230.0 | 3196.4 | 1723.5 | 83.3 | 2991.4 |
| 176 | | [At5g44300](http://atted.jp/cgi-bin/coex_Ath.cgi?gene=At5g44300&sort=all) | | dormancy/auxin associated | **185.7** | | 285.5 | 656.8 | 5129.5 | 2937.3 | 1100.4 |
| 177 | | [At3g07450](http://atted.jp/cgi-bin/coex_Ath.cgi?gene=At3g07450&sort=all) | | LTP | **185.8** | | 253.7 | 176.3 | 217.4 | 2431.5 | 9426.6 |
| 178 | | [At5g62080](http://atted.jp/cgi-bin/coex_Ath.cgi?gene=At5g62080&sort=all) | | LTP | **186.4** | | 158.8 | 173.3 | 1799.5 | 12921.5 | 8335.0 |
| 179 | | [At1g07795](http://atted.jp/cgi-bin/coex_Ath.cgi?gene=At1g07795&sort=all) | |  | **186.5** | | 178.1 | 910.9 | 79.3 | 1938.5 | 8554.3 |
| 180 | | [At2g28355](http://atted.jp/cgi-bin/coex_Ath.cgi?gene=At2g28355&sort=all) | | LCR5 | **187.3** | | 262.5 | 11872.9 | 3684.8 | 5320.0 | 6409.0 |
| 181 | | [At3g51490](http://atted.jp/cgi-bin/coex_Ath.cgi?gene=At3g51490&sort=all) | | TMT3 | **187.5** | | 214.2 | 605.0 | 877.7 | 314.8 | 12858.4 |
| 182 | | [At3g17630](http://atted.jp/cgi-bin/coex_Ath.cgi?gene=At3g17630&sort=all) | | CHX19 | **187.8** | | 226.8 | 12578.0 | 2241.0 | 6957.4 | 711.9 |
| 183 | | [At5g60090](http://atted.jp/cgi-bin/coex_Ath.cgi?gene=At5g60090&sort=all) | | kinase | **188.4** | | 245.4 | 895.7 | 2466.5 | 1076.7 | 6740.5 |
| 184 | | [At5g16960](http://atted.jp/cgi-bin/coex_Ath.cgi?gene=At5g16960&sort=all) | | oxidoreductase | **192.2** | | 207.7 | 6769.7 | 3019.2 | 3868.5 | 2041.4 |
| 185 | | [At1g04670](http://atted.jp/cgi-bin/coex_Ath.cgi?gene=At1g04670&sort=all) | |  | **193.5** | | 302.7 | 5220.2 | 6593.7 | 2821.7 | 9171.4 |
| 186 | | [At1g61700](http://atted.jp/cgi-bin/coex_Ath.cgi?gene=At1g61700&sort=all) | | RPB10 | **194.3** | | 225.6 | 6505.5 | 1405.4 | 412.2 | 16788.6 |
| 187 | | [At3g46770](http://atted.jp/cgi-bin/coex_Ath.cgi?gene=At3g46770&sort=all) | | transcription | **194.8** | | 385.8 | 2855.4 | 2814.8 | 1095.8 | 11523.0 |
| 188 | | [At1g54560](http://atted.jp/cgi-bin/coex_Ath.cgi?gene=At1g54560&sort=all) | | XIE | **195.3** | | 296.5 | 2324.0 | 401.3 | 1625.4 | 16208.1 |
| 189 | | [At4g27980](http://atted.jp/cgi-bin/coex_Ath.cgi?gene=At4g27980&sort=all) | |  | **196.1** | | 249.1 | 3653.4 | 5236.9 | 4315.2 | 7413.9 |
| 190 | | [At5g52340](http://atted.jp/cgi-bin/coex_Ath.cgi?gene=At5g52340&sort=all) | | EXO70A2 | **196.8** | | 241.9 | 6458.8 | 1798.6 | 5402.4 | 875.0 |
| 191 | | [At2g29790](http://atted.jp/cgi-bin/coex_Ath.cgi?gene=At2g29790&sort=all) | |  | **196.8** | | 295.8 | 14855.6 | 3902.4 | 6539.9 | 10264.6 |
| 192 | | [At5g57240](http://atted.jp/cgi-bin/coex_Ath.cgi?gene=At5g57240&sort=all) | | ORP4C | **197.0** | | 307.2 | 6864.3 | 5565.9 | 13631.3 | 19798.4 |
| 193 | | [At4g18960](http://atted.jp/cgi-bin/coex_Ath.cgi?gene=At4g18960&sort=all) | | AG | **197.2** | | 392.6 | 272.0 | 10590.5 | 6005.9 | 3319.7 |
| 194 | | [At2g18420](http://atted.jp/cgi-bin/coex_Ath.cgi?gene=At2g18420&sort=all) | |  | **198.1** | | 260.1 | 3795.4 | 5435.5 | 8658.7 | 642.4 |
| 195 | | [At3g08560](http://atted.jp/cgi-bin/coex_Ath.cgi?gene=At3g08560&sort=all) | | VHA-E2 | **198.3** | | 270.5 | 3398.7 | 1832.4 | 4199.8 | 3675.9 |
| 196 | | [At5g28470](http://atted.jp/cgi-bin/coex_Ath.cgi?gene=At5g28470&sort=all) | | transporter | **199.2** | | 243.1 | 2809.6 | 444.7 | 325.0 | 18564.7 |
| 197 | | [At3g26125](http://atted.jp/cgi-bin/coex_Ath.cgi?gene=At3g26125&sort=all) | | CYP86C2 | **199.8** | | 274.2 | 14076.7 | 9353.8 | 5795.1 | 20651.8 |
| 198 | | 266675_s_at | |  | **200.6** | | 188.9 | 745.5 | 2511.8 | 5291.4 | 7952.1 |
| 199 | | [At1g02050](http://atted.jp/cgi-bin/coex_Ath.cgi?gene=At1g02050&sort=all) | | synthase | **200.8** | | 254.2 | 38.9 | 2577.1 | 8732.5 | 2314.6 |
| 200 | | [At1g23520](http://atted.jp/cgi-bin/coex_Ath.cgi?gene=At1g23520&sort=all) | |  | **204.2** | | 254.9 | 5133.2 | 2714.5 | 2321.0 | 1260.6 |
| 201 | | [At4g39010](http://atted.jp/cgi-bin/coex_Ath.cgi?gene=At4g39010&sort=all) | | GH9B18 | **204.7** | | 254.9 | 501.8 | 3011.9 | 7381.9 | 3514.5 |
| 202 | | [At1g23690](http://atted.jp/cgi-bin/coex_Ath.cgi?gene=At1g23690&sort=all) | |  | **204.8** | | 274.3 | 500.8 | 6096.4 | 1394.9 | 4346.2 |
| 203 | | [At5g10800](http://atted.jp/cgi-bin/coex_Ath.cgi?gene=At5g10800&sort=all) | | RRM | **205.2** | | 248.8 | 6585.0 | 3576.1 | 5259.8 | 725.0 |
| 204 | | [At1g03710](http://atted.jp/cgi-bin/coex_Ath.cgi?gene=At1g03710&sort=all) | |  | **206.0** | | 519.1 | 2650.4 | 3996.0 | 4258.8 | 5679.6 |
| 205 | | [At1g23590](http://atted.jp/cgi-bin/coex_Ath.cgi?gene=At1g23590&sort=all) | |  | **211.1** | | 309.3 | 1888.1 | 1624.2 | 1314.0 | 3373.0 |
| 206 | | [At2g13680](http://atted.jp/cgi-bin/coex_Ath.cgi?gene=At2g13680&sort=all) | | CALS5 | **211.6** | | 297.4 | 549.7 | 4820.4 | 4073.6 | 4821.5 |
| 207 | | [At1g11920](http://atted.jp/cgi-bin/coex_Ath.cgi?gene=At1g11920&sort=all) | | lyase | **212.4** | | 235.2 | 1708.7 | 451.0 | 2828.3 | 1433.4 |
| 208 | | [At1g06450](http://atted.jp/cgi-bin/coex_Ath.cgi?gene=At1g06450&sort=all) | | transcription | **213.3** | | 178.8 | 6634.3 | 15683.5 | 3040.4 | 21285.8 |
| 209 | | [At5g17480](http://atted.jp/cgi-bin/coex_Ath.cgi?gene=At5g17480&sort=all) | | APC1 | **213.7** | | 325.8 | 13176.0 | 9480.8 | 7778.8 | 4283.3 |
| 210 | | [At2g33690](http://atted.jp/cgi-bin/coex_Ath.cgi?gene=At2g33690&sort=all) | | LEA | **216.0** | | 330.5 | 5403.4 | 4569.5 | 9175.2 | 508.5 |
| 211 | | [At2g27880](http://atted.jp/cgi-bin/coex_Ath.cgi?gene=At2g27880&sort=all) | | AGO5 | **217.2** | | 545.3 | 998.2 | 3120.6 | 4923.9 | 2986.1 |
| 212 | | [At5g23970](http://atted.jp/cgi-bin/coex_Ath.cgi?gene=At5g23970&sort=all) | | transferase | **218.1** | | 502.9 | 4822.9 | 4874.2 | 784.3 | 4564.1 |
| 213 | | [At4g37900](http://atted.jp/cgi-bin/coex_Ath.cgi?gene=At4g37900&sort=all) | | glycine-rich | **218.8** | | 215.2 | 8995.1 | 14489.3 | 10007.7 | 10640.8 |
| 214 | | [At1g72290](http://atted.jp/cgi-bin/coex_Ath.cgi?gene=At1g72290&sort=all) | | Kunitz | **221.1** | | 345.4 | 1722.1 | 2676.7 | 6068.6 | 9787.4 |
| 215 | | [At5g60080](http://atted.jp/cgi-bin/coex_Ath.cgi?gene=At5g60080&sort=all) | | kinase | **221.8** | | 224.7 | 1463.2 | 385.5 | 8126.9 | 2999.9 |
| 216 | | [At2g39820](http://atted.jp/cgi-bin/coex_Ath.cgi?gene=At2g39820&sort=all) | | translation | **222.6** | | 357.9 | 1227.5 | 571.7 | 1264.1 | 4958.5 |
| 217 | | [At5g61110](http://atted.jp/cgi-bin/coex_Ath.cgi?gene=At5g61110&sort=all) | | protein binding | **223.4** | | 251.3 | 1243.9 | 4487.3 | 2123.6 | 6332.9 |
| 218 | | [At5g17830](http://atted.jp/cgi-bin/coex_Ath.cgi?gene=At5g17830&sort=all) | |  | **226.6** | | 187.2 | 14242.5 | 8538.9 | 17506.8 | 8789.8 |
| 219 | | [At4g23660](http://atted.jp/cgi-bin/coex_Ath.cgi?gene=At4g23660&sort=all) | | PPT1 | **228.7** | | 138.5 | 5413.9 | 7399.3 | 18426.1 | 12553.9 |
| 220 | | [At1g61630](http://atted.jp/cgi-bin/coex_Ath.cgi?gene=At1g61630&sort=all) | | ENT7 | **228.8** | | 194.8 | 7645.3 | 4001.9 | 2385.7 | 7694.2 |
| 221 | | [At1g08730](http://atted.jp/cgi-bin/coex_Ath.cgi?gene=At1g08730&sort=all) | | XIC | **229.8** | | 322.4 | 2691.5 | 1342.9 | 1059.2 | 18594.4 |
| 222 | | [At1g21540](http://atted.jp/cgi-bin/coex_Ath.cgi?gene=At1g21540&sort=all) | | AMP-binding | **231.9** | | 447.3 | 6443.3 | 16336.2 | 2230.2 | 2135.1 |
| 223 | | [At1g23700](http://atted.jp/cgi-bin/coex_Ath.cgi?gene=At1g23700&sort=all) | | kinase | **232.2** | | 195.8 | 2929.0 | 4970.1 | 35.9 | 21086.2 |
| 224 | | [At3g13662](http://atted.jp/cgi-bin/coex_Ath.cgi?gene=At3g13662&sort=all) | |  | **236.1** | | 743.0 | 2412.8 | 1673.4 | 5410.1 | 3834.7 |
| 225 | | [At2g42990](http://atted.jp/cgi-bin/coex_Ath.cgi?gene=At2g42990&sort=all) | | hydrolase | **237.4** | | 545.2 | 49.4 | 294.5 | 12024.9 | 18153.2 |
| 226 | | [At5g12270](http://atted.jp/cgi-bin/coex_Ath.cgi?gene=At5g12270&sort=all) | | oxygenase | **237.4** | | 312.4 | 16609.8 | 1856.9 | 462.7 | 607.3 |
| 227 | | [At5g11400](http://atted.jp/cgi-bin/coex_Ath.cgi?gene=At5g11400&sort=all) | | kinase | **238.1** | | 351.8 | 2938.5 | 4499.9 | 2698.4 | 3975.7 |
| 228 | | [At4g01470](http://atted.jp/cgi-bin/coex_Ath.cgi?gene=At4g01470&sort=all) | | TIP1;3 | **239.4** | | 349.6 | 871.3 | 61.5 | 770.5 | 8049.8 |
| 229 | | [At1g80970](http://atted.jp/cgi-bin/coex_Ath.cgi?gene=At1g80970&sort=all) | | XH | **240.2** | | 251.3 | 2280.3 | 6396.6 | 2245.2 | 8965.2 |
| 230 | | 257558_s_at | |  | **240.4** | | 326.2 | 6934.1 | 16929.0 | 6076.8 | 12692.3 |
| 231 | | [At1g25330](http://atted.jp/cgi-bin/coex_Ath.cgi?gene=At1g25330&sort=all) | | bHLH | **240.6** | | 380.6 | 12831.5 | 6212.3 | 3913.3 | 15986.0 |
| 232 | | [At4g13230](http://atted.jp/cgi-bin/coex_Ath.cgi?gene=At4g13230&sort=all) | | LEA | **241.1** | | 353.7 | 10838.0 | 4263.4 | 3367.3 | 1589.2 |
| 233 | | [At2g17370](http://atted.jp/cgi-bin/coex_Ath.cgi?gene=At2g17370&sort=all) | | HMG2 | **242.5** | | 365.4 | 10414.3 | 2331.4 | 11970.3 | 8822.6 |
| 234 | | [At1g22090](http://atted.jp/cgi-bin/coex_Ath.cgi?gene=At1g22090&sort=all) | | emb2204 | **243.6** | | 73.2 | 7624.0 | 877.6 | 6683.6 | 2903.5 |
| 235 | | [At4g11030](http://atted.jp/cgi-bin/coex_Ath.cgi?gene=At4g11030&sort=all) | | synthetase | **243.7** | | 330.9 | 9438.7 | 8238.1 | 5570.9 | 3116.5 |
| 236 | | [At1g44970](http://atted.jp/cgi-bin/coex_Ath.cgi?gene=At1g44970&sort=all) | | peroxidase | **243.9** | | 314.4 | 3895.8 | 7726.7 | 16424.2 | 5835.4 |
| 237 | | [At3g06100](http://atted.jp/cgi-bin/coex_Ath.cgi?gene=At3g06100&sort=all) | | NIP7;1 | **244.5** | | 310.4 | 5.2 | 1510.1 | 435.6 | 696.4 |
[truncated: 610,887 more chars]
